# Cooperative Aldehyde Chemistry Maps an Orthogonal Lysine Reactivity Landscape

**DOI:** 10.1021/jacs.6c01030

**Published:** 2026-04-02

**Authors:** Ana Villalobos Galindo, Pinki Sihag, John M. Talbott, Monika Raj

**Affiliations:** Department of Chemistry, 1371 Emory University, Atlanta, Georgia 30322, United States

## Abstract

Reactive aldehyde metabolites are commonly viewed as drivers of nonspecific protein damage and stochastic cross-linking. Here, we show that cooperative aldehyde chemistry can generate multicomponent, mass-consistent electrophilic intermediates in water with strong lysine bias and site selectivity. Specifically, malondialdehyde (MDA) couples with monoaldehydes (e.g., acetaldehyde and benzaldehyde) to form a cooperative intermediate that channels reactivity toward lysine, yielding chemically stable dihydropyridine (DHP) adducts under aqueous conditions. Across peptides, purified proteins, and complex lysates, this pathway produces nonrandom, lysine-selective labeling. Comparison with NHS-ester chemoproteomic data sets suggests a distinct selectivity regime: whereas NHS acylation broadly tracks nucleophile accessibility with weak context dependence, cooperative MDA-monoaldehyde chemistry preferentially labels lysines in acidic microenvironments, consistent with an electrostatically influenced association-and-capture model that promotes productive cyclization to stable DHP adducts. Finally, electronic tuning of the DHP scaffold affords red-shifted emission compatible with live-cell imaging. Together, these results establish a tunable cooperative aldehyde platform that expands selective lysine bioconjugation chemistry and enables proteome-scale mapping of lysine microenvironment reactivity not captured by conventional acylating reagents.

## Graphical abstract

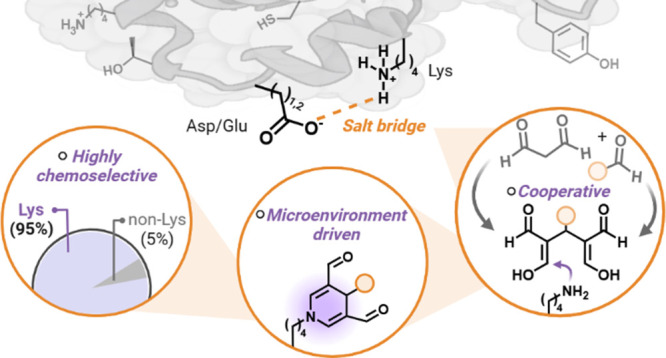

## Introduction

Reactive aldehyde metabolites generated during metabolism, lipid peroxidation, and environmental exposure covalently modify proteins and reshape the proteome.^1–5^ Classic work has largely emphasized single-metabolite chemistry, in which aldehydes such as malondialdehyde (MDA) or acetaldehyde react with multiple nucleophilic residues to produce heterogeneous mixtures of carbonyl adducts, cross-links, and aggregates.^3,6–9^ This chemical diversity has complicated efforts to understand how aldehyde burden translates into selective protein remodeling in complex biological settings.

A complementary and less systematically explored reactivity mode arises when aldehydes act cooperatively, assembling multicomponent electrophiles that access reaction pathways unavailable to either aldehyde alone.^10–14^ Cooperative malondialdehyde-acetaldehyde (MAA) and related dihydropyridine (DHP)-type products have been detected in vivo under combined aldehyde burdens (including smoke+alcohol exposure models) and are widely used as stable immunochemical epitopes in toxicology and inflammation research.^13,14^ Yet despite their biological prevalence, the molecular rules that govern where and how such cooperative electrophiles engage proteins remain poorly defined.

A key limitation is that current chemical proteomic tools for lysine mapping, including NHS esters, rely predominantly on nucleophilicity and therefore preferentially label the deprotonated fraction of the lysine proteome (Figure 1a).^15,16^ As a result, many lysine sites constrained by salt bridges and electrostatic interactions may be undersampled to systematic chemical profiling. Deconvoluting the reactivity of these electrostatically gated sites is important not only for understanding aldehyde-associated toxicity, but also for expanding chemoproteomic coverage into regions of lysine reactivity space that standard acylation chemistry under-samples. Defining these rules is essential to move the field from describing stochastic damage to mapping microenvironment-governed chemical events.

**Figure 1:**
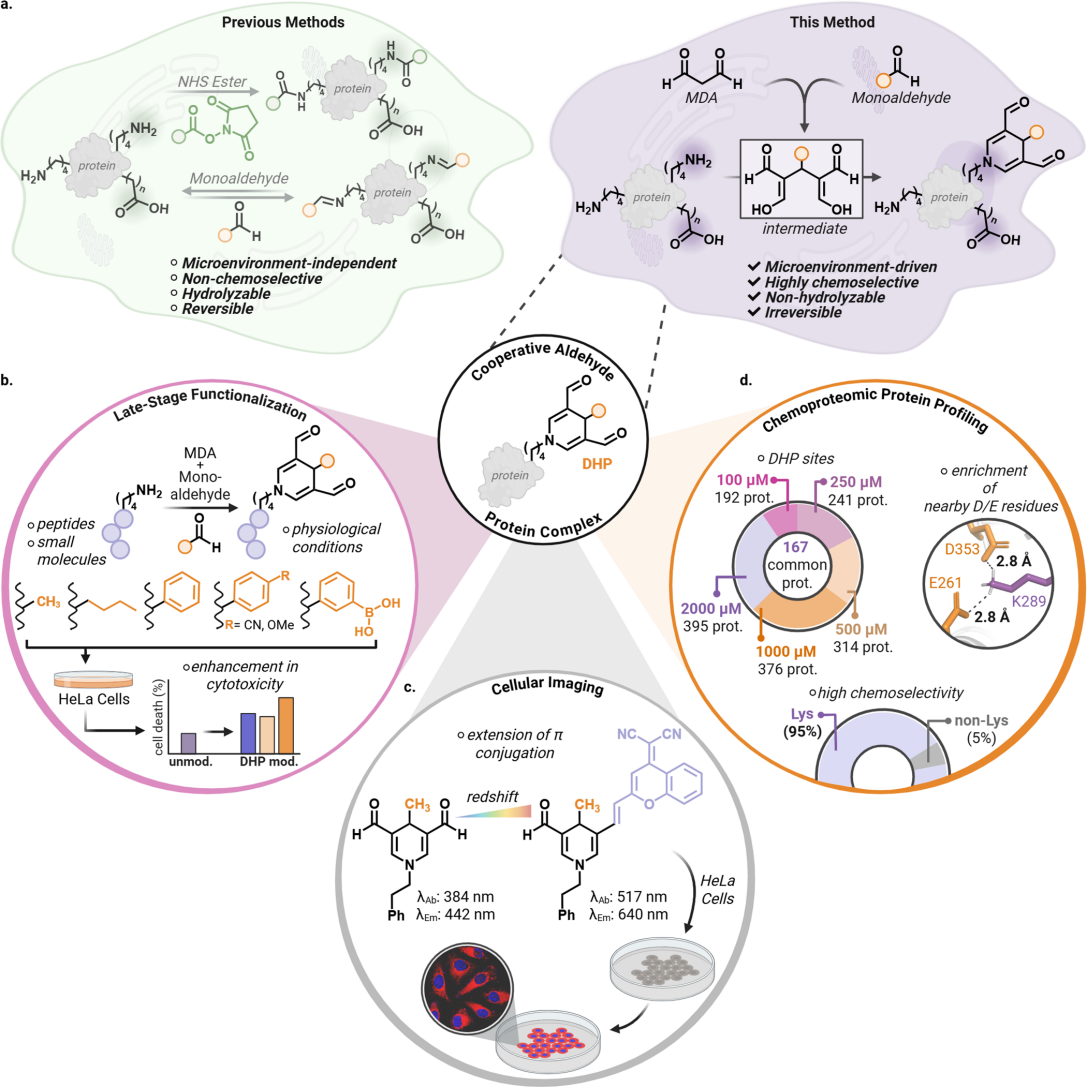
Cooperative metabolite strategy for targeted peptide and protein modification. (a) Prior lysine-labeling approaches based on activated esters or single aldehydes often exhibit broad nucleophile reactivity, hydrolytic instability, and/or reversible adduct formation, with limited dependence on local microenvironment. Here we show that reactive aldehyde metabolites, dicarbonyls such as malondialdehyde (MDA) and monoaldehydes (acetaldehyde and benzaldehyde) can act cooperatively to generate electrophilic intermediates that drive lysine-selective, irreversible modification of peptides and proteins. Labeling is strongly influenced by local residue context, with enrichment at lysines embedded in acidic microenvironments. (b) Late-stage functionalization of peptides and small molecules using MDA-monoaldehyde complexes under physiological conditions enables chemoselective lysine bioconjugation, yielding stable DHP adducts that can measurably alter peptide properties in cells under the conditions tested as compared to unmodified peptides. (c) Structural variation in the DHP core allows for fine-tuning of the photophysical properties, resulting in bright, red-shifted emission suitable for live-cell imaging with minimal cytotoxicity under the conditions tested. (d) Dose-dependent chemoproteomic profiling with an MDA-benzaldehyde complex identifies 167 unique proteins reproducibly labeled across concentrations. In comparison to NHS-ester acylation (typically exhibiting broader nucleophile reactivity and weaker context signatures), cooperative MDA-monoaldehyde chemistry enriches lysine labeling in acidic microenvironments, consistent with an electrostatic association-and-capture model that promotes productive cyclization into stable DHP adducts. MDA-monoaldehyde chemistry is strongly lysine-selective, with 95% lysine labeling and minimal nonlysine modification (5%). Created in BioRender. Villalobos, A. (2026) https://BioRender.com/lbznvad.

Here, we map the site selectivity of cooperative MDA-monoaldehyde chemistry using an integrated chemical and proteomic workflow. We show that MDA reacts not only with acetaldehyde but also with aromatic monoaldehydes, including benzaldehyde, to form DHP adducts under aqueous conditions (Figure 1a). Across peptide and protein substrates, this cooperative pathway displays a pronounced lysine preference and enables systematic identification of favored modification sites in peptides, intact proteins, and complex lysates. Mechanistically, the cooperative system proceeds through a cascade addition-cyclization sequence that yields chemically stable, homogeneous DHP conjugates suitable for bioconjugation and reactivity profiling, in contrast to single-aldehyde reactions that often produce heterogeneous mixtures of labile and/or reversible adducts.^17–19^

A central advance of this work is to quantify how cooperative aldehyde labeling is shaped by local electrostatics, rather than the steric/nucleophilicity logic that dominates standard acylating reagents. Comparison with an NHS-ester probe reveals an orthogonal selectivity regime: whereas NHS-ester chemistry shows weak microenvironment dependence, cooperative MDA-monoaldehyde labeling enriches lysines embedded in acidic microenvironments and exhibits a distinct motif signature. Proteome-scale profiling further reveals nonrandom, site-resolved DHP labeling distributed across specific microenvironmental classes, supporting the view that local electrostatics, not simple solvent exposure, can gate cooperative metabolite reactivity at lysine.

Beyond mapping selectivity, the DHP scaffold provides a tunable chemical platform (Figure 1b–d). Electronic modulation of the DHP core enables red-shifted emission compatible with live-cell imaging, extending cooperative aldehyde chemistry from mechanistic insight into functional probe design (Figure 1c).^20–22^ Finally, we demonstrate applications in late-stage peptide diversification, selective protein labeling, and chemoproteomic mapping of lysine microenvironment reactivity (Figure 1b–d). Together, these studies position cooperative MDA-monoaldehyde chemistry as a complementary approach for lysine labeling and for mapping electrostatically enriched proteome microenvironments that are poorly captured by conventional acylating reagents, potentially providing a route to deconvolute selective protein remodeling events associated with cooperative aldehyde burdens.

## Results and Discussion

### Design and Optimization of a Cooperative MDA-Monoaldehyde Labeling Reaction

To evaluate cooperative MDA-monoaldehyde chemistry as a lysine-labeling strategy, we began with a model peptide bearing a single lysine residue, Ac-NKF (1a). Reaction parameters were systematically optimized by varying the MDA:acetaldehyde ratio under acidic and near-physiological aqueous conditions (Figure 2a; MDA synthesis, Supplementary Figure 1). Under acidic conditions (sodium acetate buffer, pH 4), 1a was quantitatively converted to the DHP adduct 2a using 2 equiv of MDA and 3 equiv of acetaldehyde (Figure 2a, Entry 1, Supplementary Figure 2).

**Figure 2:**
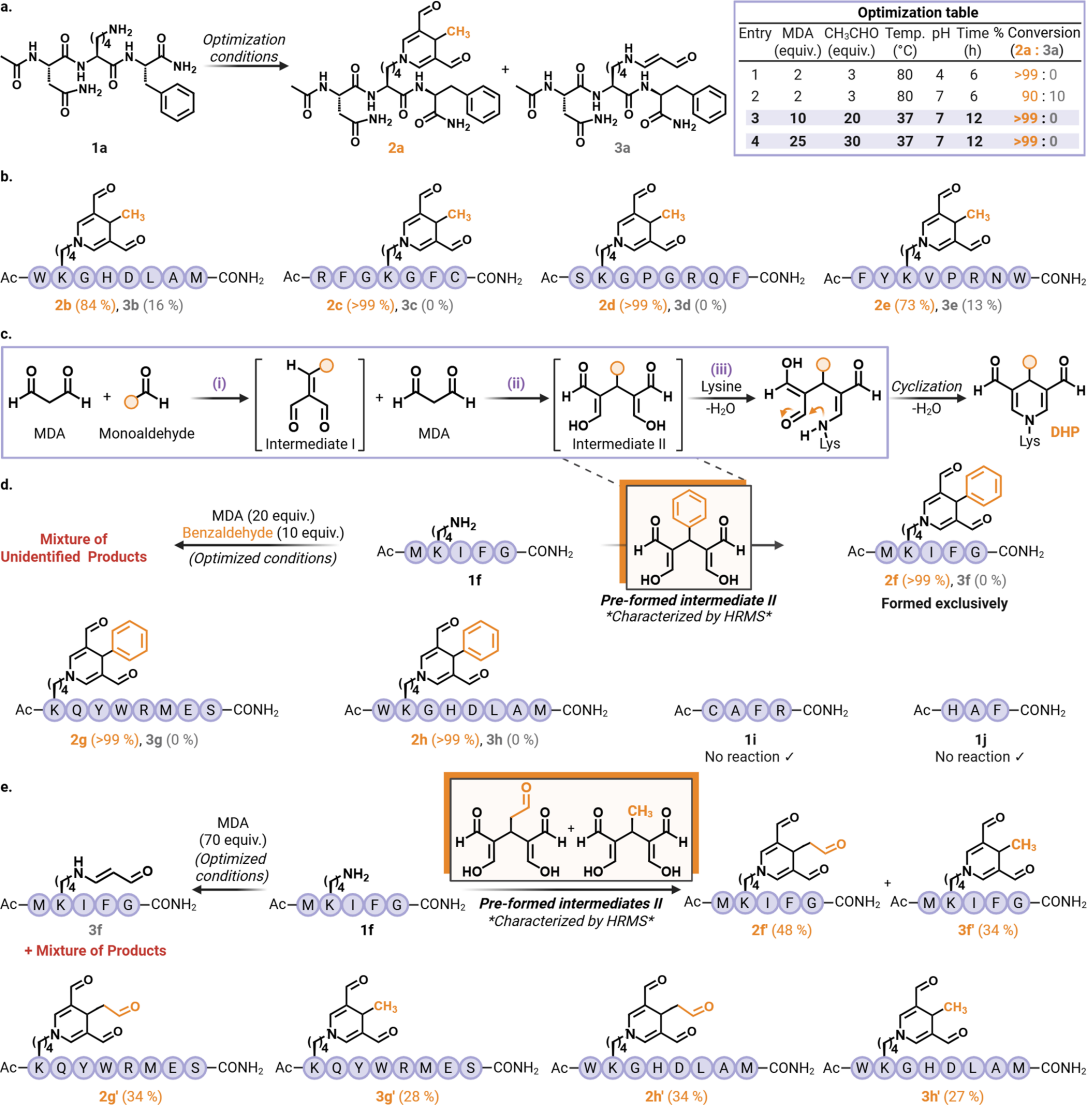
MDA-monoaldehyde reaction optimization and substrate scope of cooperative complexes in peptide modification. (a) Optimization of cooperative MDA-monoaldehyde reaction using model peptide 1a (Ac-NKF), yielding DHP adduct 2a and minor single-addition product 3a. Optimized conditions: 10 equiv of MDA and 20 equiv of acetaldehyde in sodium phosphate buffer pH 7 at 37 °C for 12 h. (b) Chemoselectivity analysis across lysine-containing peptides 1b–1e bearing additional reactive residues (Trp, His, Asp, Met, Arg, Cys, Tyr, Asn, Ser, Gln). Modification occurred exclusively at lysine, affording DHP adducts 2b–2e without detectable off-target reactions. (c) Proposed three-step cascade mechanism of cooperative complex formation: initial nucleophilic addition, generation of reactive intermediate II (*characterized by HRMS), and intramolecular cyclization to the stable DHP product. (d) Substrate scope of the preformed MDA-benzaldehyde intermediate II (*characterized by HRMS) demonstrating efficient and selective DHP formation (2f–2h) with no modification of Cys- or His-containing control peptides 1i and 1j. (e) Reactivity of self-cooperative MDA-MDA complexes (intermediate II, *characterized by HRMS), yielding corresponding DHP products 2f’–2h’ and 3f’–3h’, illustrating the structural versatility of cooperative aldehyde chemistry. Created in BioRender. Villalobos, A. (2026) https://BioRender.com/ycoovlv.

We next evaluated the reaction in sodium phosphate buffer (pH 7) to assess compatibility with protein labeling. Maintaining the same acetaldehyde:MDA ratio (1.5:1) afforded efficient formation of 2a (∼90% conversion), together with a minor side product (∼10%) corresponding to a single MDA addition to lysine (3a) (Figure 2a, Entry 2, Supplementary Figure 2). The appearance of 3a under these conditions is consistent with incomplete channeling of MDA into the cooperative pathway, leaving a fraction of free MDA available for noncooperative lysine modification.

To suppress competing single-addition chemistry and drive the reaction toward DHP formation, we reduced the acetaldehyde:MDA ratio to 1.2:1 and increased the overall aldehyde equivalents relative to peptide (10 or 25 equiv MDA, 20 or 30 equiv acetaldehyde). Under these conditions, the reaction proceeded to quantitative conversion (>99%), affording DHP product 2a as the sole observable species by LC-MS (Figure 2a, Entries 3–4, Supplementary Figure 2). Notably, high conversion to 2a was maintained even when the reaction time was shortened to 6 h, indicating that DHP formation is robust rather than narrowly tuned. These optimized parameters were used for subsequent peptide, protein, and proteome-scale studies unless noted otherwise.

### Chemoselectivity Studies

To evaluate residue selectivity, we applied the optimized MDA-acetaldehyde conditions to a panel of lysine-containing peptides that also incorporate potentially competing nucleophiles: Ac-WKGHDLAM (1b), Ac-RFGKGFC (1c), Ac-SKGPGRQF (1d), and Ac-FYKVPRNW (1e), which collectively present Trp, His, Asp, Met, Arg, Cys, Tyr, Asn, Ser, and Gln side chains alongside lysine (Figure 2b). Across all sequences (1b–1e), DHP formation was observed exclusively at lysine, affording DHP-labeled peptides 2b–2e. Minor byproducts consistent with single MDA addition to lysine (e.g., 3b and 3e) were detected in select cases, but no modification of other nucleophilic residues was observed under these conditions (Figure 2b, Supplementary Figure 3). This lysine-biased outcome contrasts with the broader residue reactivity typically associated with single carbonyl species, which often generate heterogeneous adduct mixtures across multiple amino acids.^4–7^

### Mechanistic Framework and Detection of the Cooperative Intermediate

The selective formation of DHP products is consistent with a cooperative, multistep assembly mechanism in which aldehyde-aldehyde reactions generate reactive intermediates that subsequently engage lysine. We propose a three-step cascade comprising: (i) MDA addition to the monoaldehyde to form Intermediate I, (ii) recruitment of a second MDA equivalent to generate the cooperative Intermediate II, and (iii) lysine capture and cyclization to DHP (Figure 2c).^17,18^ Intermediate II was intercepted by mass spectrometry under precomplexation conditions, consistent with formation of a cooperative intermediate that is competent for downstream lysine capture (Supplementary Figure 4). MDA alone or monoaldehyde alone did not yield the signature DHP adduct under matched conditions, and mixing the components without a preformation step (or reversing the order-of-addition) decreased DHP labeling. Together with the stoichiometry dependence observed during optimization (Figure 2a), these results highlight how aldehyde ratio and order-of-addition govern productive channeling into DHP formation while minimizing noncooperative single-addition chemistry.

### Substrate Scope of Cooperative MDA-Monoaldehyde Complexes

To assess generality beyond acetaldehyde, we surveyed monoaldehydes capable of generating structurally distinct DHP-lysine adducts. As a representative aromatic substrate, we selected benzaldehyde, which is encountered in environmental exposures, including flavored e-cigarette aerosols.^23–26^ When benzaldehyde was reacted directly with the model peptide Ac-MKIFG (1f) under the one-pot conditions optimized for acetaldehyde, a mixture of products was observed (Figure 2d, Supplementary Figure 4), consistent with reduced efficiency and competing pathways likely arising from steric and/or electronic differences in the aromatic aldehyde partner.

To enable controlled DHP formation, we therefore adopted a two-step protocol in which the MDA-benzaldehyde cooperative species was preassembled prior to peptide addition as confirmed by MS (Intermediate II, Supplementary Figure 4). The preassembled Intermediate II was purified by HPLC and lyophilized. The dry powder is stable at −80 °C for at least 2–3 months and was reconstituted in buffer immediately before use. Under these conditions, peptide 1f underwent clean, quantitative conversion to the desired DHP adduct 2f with no detectable side products (Figure 2d, Supplementary Figure 4). We use precomplexation to generate a reproducible reagent state for controlled selectivity mapping, without implying that an identical preassembled intermediate is required in vivo. This precomplexation strategy was extended to additional peptide substrates of varying length and sequence (1g and 1b), which likewise afforded efficient lysine-selective labeling (>99%) to give 2g and 2h (Figure 2d, Supplementary Figure 4). To further challenge residue selectivity, we examined peptides lacking lysine but containing nucleophiles commonly modified by electrophiles. Under identical conditions, peptides containing only Cys (1i) or His (1j) showed no detectable modification, supporting a strong preference of the preformed intermediate II for lysine and its conversion into a single, homogeneous DHP product (Figure 2d, Supplementary Figure 5). Notably, the core mechanistic hallmarks, Intermediate II formation by MS and selective channeling into a single DHP lysine adduct, were conserved across acetaldehyde and benzaldehyde partners under the corresponding optimized conditions (Supplementary Figures 4 and 5).

### Self-Cooperative MDA-MDA Reactivity

We next tested whether MDA can serve as both partners in a self-cooperative pathway, generating an MDA-MDA cooperative species capable of DHP formation (Figure 2e). As expected, when MDA was incubated with lysine-containing peptide in the absence of precomplexation, the dominant product corresponded to a simple MDA-lysine Schiff-base-type adduct (3f). In contrast, preassembling the MDA-MDA cooperative species as detected by MS prior to peptide addition enabled formation of the DHP adduct (2f’), demonstrating that MDA can engage in self-cooperative reactivity to access the DHP scaffold (Figure 2e, Supplementary Figure 6).^9,27–36^ In these experiments, we also observed an MDA-acetaldehyde-type DHP product (3f’), consistent with prior reports of acetaldehyde formation from MDA under certain conditions.^17,37^ This observation underscores the chemical adaptability of aldehyde cooperativity and motivates careful control of assembly conditions when delineating cooperative pathways. Extending the preformed MDA-MDA protocol to longer peptide substrates likewise afforded the corresponding DHP products (2g’, 2h’ and 3g’, 3h’, Figure 2e, Supplementary Figures 6 and 7). Collectively, these studies establish that cooperative aldehyde assembly exhibits broad scope across multiple monoaldehyde partners including self-cooperative MDA yielding chemically stable, lysine-selective DHP conjugates suitable for subsequent protein- and proteome-scale interrogation.

### Substrate Scope of Monoaldehydes in Selective Modification

To determine the substrate scope across multiple monoaldehyde partners for cooperative MDA-monoaldehyde chemistry, we next evaluated its utility for late-stage functionalization (LSF) of peptide scaffolds as a route to rapidly diversify structure and physicochemical properties. We selected Ac-QPK (4a), a lysine-containing cytotoxic peptide, as a model substrate and subjected it to cooperative labeling with a panel of aliphatic and aromatic monoaldehyde partners, including acetaldehyde, valeraldehyde, benzaldehyde, 4-cyanobenzaldehyde, 4-methoxybenzaldehyde, and 3-boronic acid benzaldehyde (Figure 3a).^38^ These reactions afforded a small library of DHP-conjugated peptides (4b–4g) bearing electronically and sterically varied substituents on the DHP core (Figure 3a, Supplementary Figure 8).

**Figure 3:**
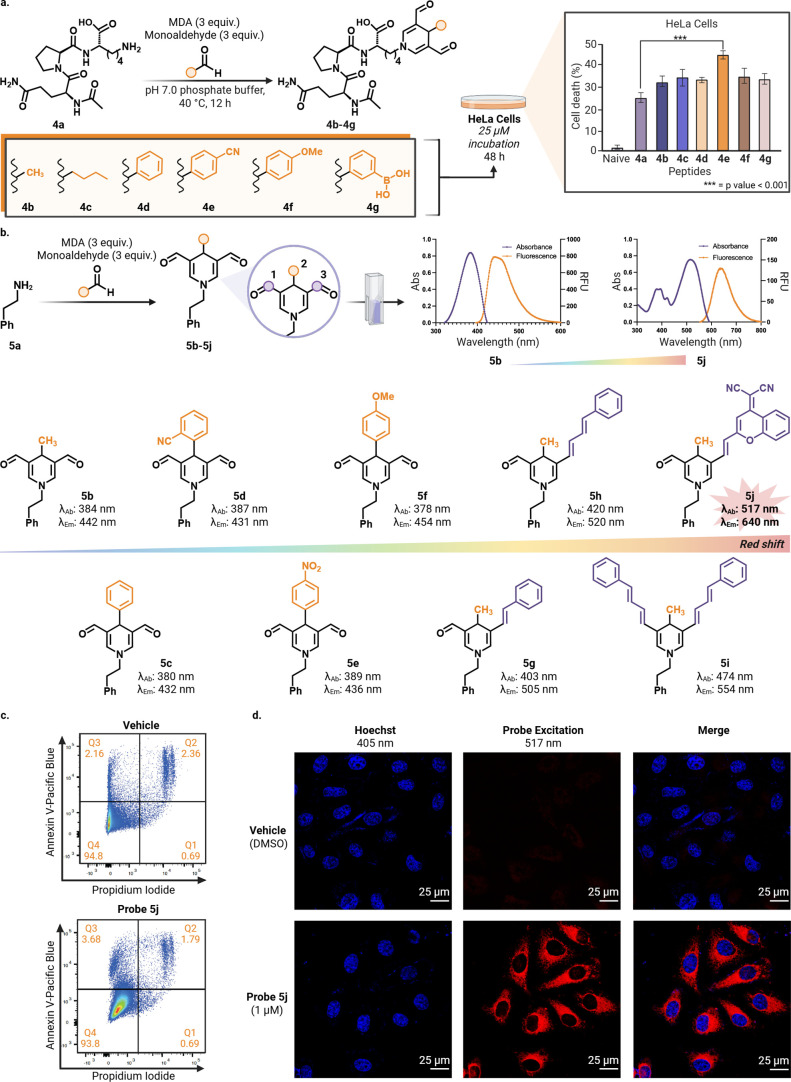
Late-stage functionalization of peptides and tuning photophysical properties of DHP for live cell imaging. (a) Late-stage functionalization of peptide Ac-QPK (4a) using cooperative MDA-monoaldehyde complexes yields DHP-modified analogs (4b–4g). Cell-based viability assays following 48 h incubation in HeLa cells show increased apparent cytotoxicity of DHP-modified variants relative to unmodified 4a. Statistical significance was evaluated by one-way ANOVA with Dunnett’s multiple-comparison test versus 4a, experiments were performed in triplicate and error bars represent mean ± SD, *** = *p* < 0.001. (b) Investigating the fluorescent properties of DHP (5b) by functionalizing position 1 and/or 3 (purple) or position 2 (orange) (5b–5j). Fluorescence and emission spectra of fluorophores 5b and 5j. Functionalization at position 3 with dicyanomethylene-4H-pyran (5j) resulted in a significant red shift (by 200 nm) in fluorescence. (c) Flow cytometry data showed negligible decrease in cell viability with exposure to 10 μM of 5j for 24 h. (d) Fluorescent microscopy of HeLa cells dosed with 1 μM of 5j and stained with Hoechst 33342, demonstrates the compatibility of DHP fluorophores for live-cell imaging. Vehicle cells were dosed with DMSO and stained with only Hoechst. Scale bar = 25 μm. Imaging repeated with separate cell passages with consistent imaging results. Created in BioRender. Villalobos, A. (2026) https://BioRender.com/ws3odre.

To gauge functional consequences of these modifications under a standardized condition, we compared the cytotoxicity of parent peptide 4a and DHP-modified analogs 4b–4g in HeLa cells (25 μM, 48 h, Figure 3a, Supplementary Figure 9). Under these conditions, all DHP variants exhibited increased apparent cytotoxicity relative to 4a, with the 4-cyano-substituted analogue 4e showing the largest effect. Because these measurements were performed at a single concentration and time point, we interpret these data as evidence that cooperative aldehyde modification can measurably alter peptide behavior, while full potency ranking and mechanism (e.g., uptake, stability, aggregation, or target engagement) will require further investigation. Collectively, these results illustrate that cooperative MDA-monoaldehyde chemistry exhibits broad substrate scope across multiple monoaldehyde partners and can serve as a practical LSF handle to tune peptide properties through modular installation of DHP substituents.

### Exploiting the Chemical Versatility and Tunable Photophysics of the DHP Scaffold

Beyond mapping site selectivity, we were intrigued by the conjugated architecture of the DHP framework and asked whether it could serve as a versatile platform for modular scaffold design. To test how substituent electronics are transmitted through the DHP π-system, we systematically varied the aldehyde building blocks used in DHP assembly. The rigid, partially conjugated DHP core exhibits intrinsic fluorescence, providing a starting point for rational red-shifting and brightness optimization toward live-cell imaging-compatible wavelengths. As a baseline, the MDA-acetaldehyde DHP adduct 5b emitted in the blue region (λ_abs_ = 384 nm, λ_em_ = 442 nm, Figure 3b, Supplementary Figures 10 and 11). Substitution at the 2-position via aromatic aldehydes (5c–5f, benzaldehyde, 2-cyano-, 4-nitro-, and 4-methoxybenzaldehyde derivatives) produced only modest spectral perturbations relative to 5b (Figure 3b, Supplementary Figures 10 and 11), consistent with limited electronic coupling between the position 2-substituent and the DHP core. We therefore targeted positions 1 and/or 3 to more directly extend π-conjugation. Installation of aromatic or styryl substituents produced pronounced bathochromic shifts (e.g., 5g: λ_abs_ = 403 nm, λ_em_ = 505 nm, 5h: λ_abs_ = 420 nm, λ_em_ = 520 nm, Figure 3b, Supplementary Figures 10 and 11).

Dual styryl substitution at both positions 1 and 3 (5i) further increased emission intensity and shifted the spectrum to longer wavelength (λ_abs_ = 474 nm, λ_em_ = 554 nm, Figure 3b, Supplementary Figures 10 and 11). To access deeper red emission, we introduced a dicyanomethylene-4H-pyran acceptor at position 3 (5j), yielding a further red-shift (λ_em_ = 640 nm). Although 5j displays a modest quantum yield (Φ = 0.14), it exhibits a large Stokes shift (133 nm), which reduces excitation/emission cross-talk and is advantageous for signal isolation in complex biological settings. Consistent with this profile, HeLa cells incubated with 5j (10 μM, 24 h, DMSO vehicle) displayed robust intracellular fluorescence, confirming that these electronically tuned scaffolds are compatible with complex biological environments (Figure 3c,d, Supplementary Figures 12–14). Collectively, these results establish the DHP core as an electronically addressable scaffold that can be tuned for diverse applications beyond bioconjugation.

### Cooperative Metabolite Complexes for Selective Modification of Proteins

We next evaluated whether cooperative MDA-monoaldehyde complexes can selectively modify lysine residues on folded proteins. As an initial benchmark, we used lysozyme chicken, which contains six lysine residues. Treatment with a preformed MDA-benzaldehyde complex led to dose-dependent labeling over 20 h (Figure 4a, Supplementary Figure 15). At 20 equiv (relative to protein), ∼76% of the protein population was converted to DHP-modified lysozyme, with up to two lysines modified. Increasing the complex to 30 equiv increased conversion to ∼87% and produced up to three modifications, whereas 50 equiv afforded >99% conversion with higher modification stoichiometry (up to six labeled lysines detected under these forcing conditions). To test whether complex formation is required for lysine modification, we performed control reactions in which lysozyme was treated with MDA alone or benzaldehyde alone, under these conditions, no detectable modification of amino acid side chains was observed (Supplementary Figure 15). Moreover, simply mixing MDA and benzaldehyde with protein under either neutral or acidic conditions resulted in little to no protein DHP labeling (Supplementary Figure 15). Kinetic and mechanistic controls argue against a simple stepwise Schiff-base pathway under the conditions tested. Attempted labeling of lysozyme by sequential addition of MDA followed by monoaldehyde yielded only trace modification (Supplementary Figure 15). In contrast, incubation with the preassembled cooperative complex (characterized by MS, Supplementary Figure 15) resulted in quantitative conversion to the DHP adduct. Together, these controls indicate that, under the conditions tested, preassembly of the cooperative complex is required to achieve efficient and selective lysine modification and formation of the stable DHP adduct. A comparable dose-dependent labeling profile was observed using a preformed MDA-MDA complex (Figure 4b, characterized by MS, Supplementary Figure 15). The higher equivalents of MDA-MDA complex afforded >99% conversion with up to three lysine modifications.

**Figure 4:**
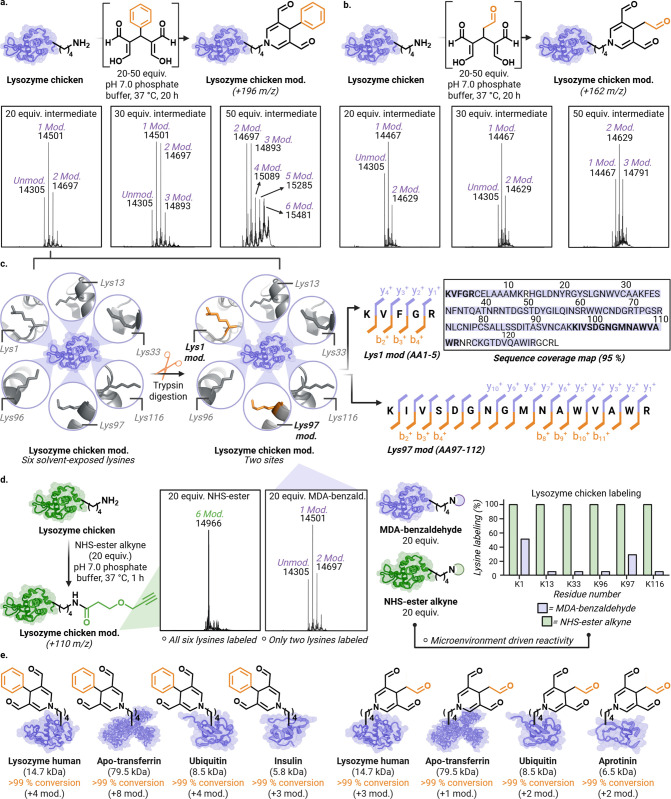
Chemoselective modification of proteins using cooperative metabolite complexes. (a) Optimization of protein labeling using a preformed MDA-benzaldehyde complex with lysozyme chicken. Increasing equivalents of the MDA-benzaldehyde intermediate (20–50 equiv) resulted in an increase in number of lysine modifications, with up to six distinct DHP adducts observed by intact protein mass spectrometry. (b) Optimization of protein labeling using preformed MDA-MDA complex showed dose-dependent increase in the modification of lysine to DHP. (c) MS/MS analysis of DHP-modified lysozyme chicken obtained by 20 equiv of MDA-benzaldehyde intermediate revealed site-specific modification exclusively at Lys1 and Lys97, while other solvent-exposed lysines remained unreacted. (d) Under matched conditions, an NHS-ester probe labeled lysozyme broadly, modifying all lysine residues with no evident regioselectivity. In contrast, cooperative MDA labeling remained site-focused and appears to be dictated by local microenvironmental features. Together, these results underscore the complementary nature of the two chemistries. (e) Application of the method to a panel of structurally and functionally diverse proteins revealed broad substrate compatibility. In all cases, cooperative metabolite complexes (MDA-benzaldehyde or MDA-MDA) achieved >99% lysine selective conversions. Created in BioRender. Villalobos, A. (2026) https://BioRender.com/jg6jjqg.

Notably, under the protein-labeling conditions examined, we did not detect Schiff-base-type byproducts or heterogeneous mixed-adduct distributions by intact-protein MS, consistent with efficient channeling into DHP formation when the cooperative species is preassembled. We next assessed site selectivity by generating DHP-modified lysozyme using 20 equiv of the MDA-benzaldehyde complex, followed by proteolysis and LC-MS/MS analysis. Labeling was consistently concentrated at two sites, Lys1 and Lys97, while the other solvent-exposed lysines and competing nucleophiles were not detectably modified under these conditions (Figure 4c, Supplementary Figure 15). In direct contrast, an amine-reactive NHS-ester probe modified essentially all lysines in lysozyme chicken under comparable conditions (Figure 4d, Supplementary Figure 16). Together, these results highlight the complementary nature of the two chemistries: whereas NHS esters broadly acylate accessible amines, the cooperative MDA platform delivers focused, reproducible lysine targeting that appears to be dictated by local microenvironmental features (e.g., electrostatics and geometric positioning) rather than solvent accessibility alone.

To assess generality, we extended labeling to a panel of structurally and functionally distinct proteins spanning a broad molecular-weight range, including lysozyme human, apo-transferrin, ubiquitin, insulin, and aprotinin (5.8–79.5 kDa, Figure 4e, Supplementary Figures 17 and 18). Across this panel, both MDA-benzaldehyde and MDA-MDA cooperative complexes afforded efficient formation of stable DHP conjugates by intact-protein analysis. Collectively, these results establish cooperative aldehyde assembly as a general strategy for lysine-selective protein modification with chemically stable DHP adducts, and motivate its application to map microenvironment-gated lysine reactivity and benchmarking orthogonality relative to conventional acylating probes.

### Chemoproteomic Profiling Reveals Electrostatic Enrichment and Orthogonality to NHS Reactivity

To define proteome-wide determinants of cooperative aldehyde reactivity, we performed activity-based protein profiling (ABPP) in T-47D human breast cancer cell lysates using a preformed MDA-benzaldehyde cooperative complex (Figure 5a, Supplementary Figure 19). Lysates were incubated with the complex (100–2000 μM, 20 h), followed by tryptic digestion and LC-MS/MS analysis. To ensure that the observed trends did not reflect slow, accumulative side chemistry or loss of protein integrity during the 20 h incubation, we validated the lysate stability. Analysis of the SDS-PAGE pattern before and after the reaction showed no gross degradation or aggregation of the proteins (Supplementary Figure 19). Additionally, the pH remained stable throughout the incubation period, confirming that the buffering capacity of the system was maintained upon addition of the cooperative complex. Importantly, the same residue selectivity and site-context trends were preserved at shorter incubation times (8 h, 500 μM), arguing against time-dependent secondary chemistry as the source of the observed selectivity.

**Figure 5:**
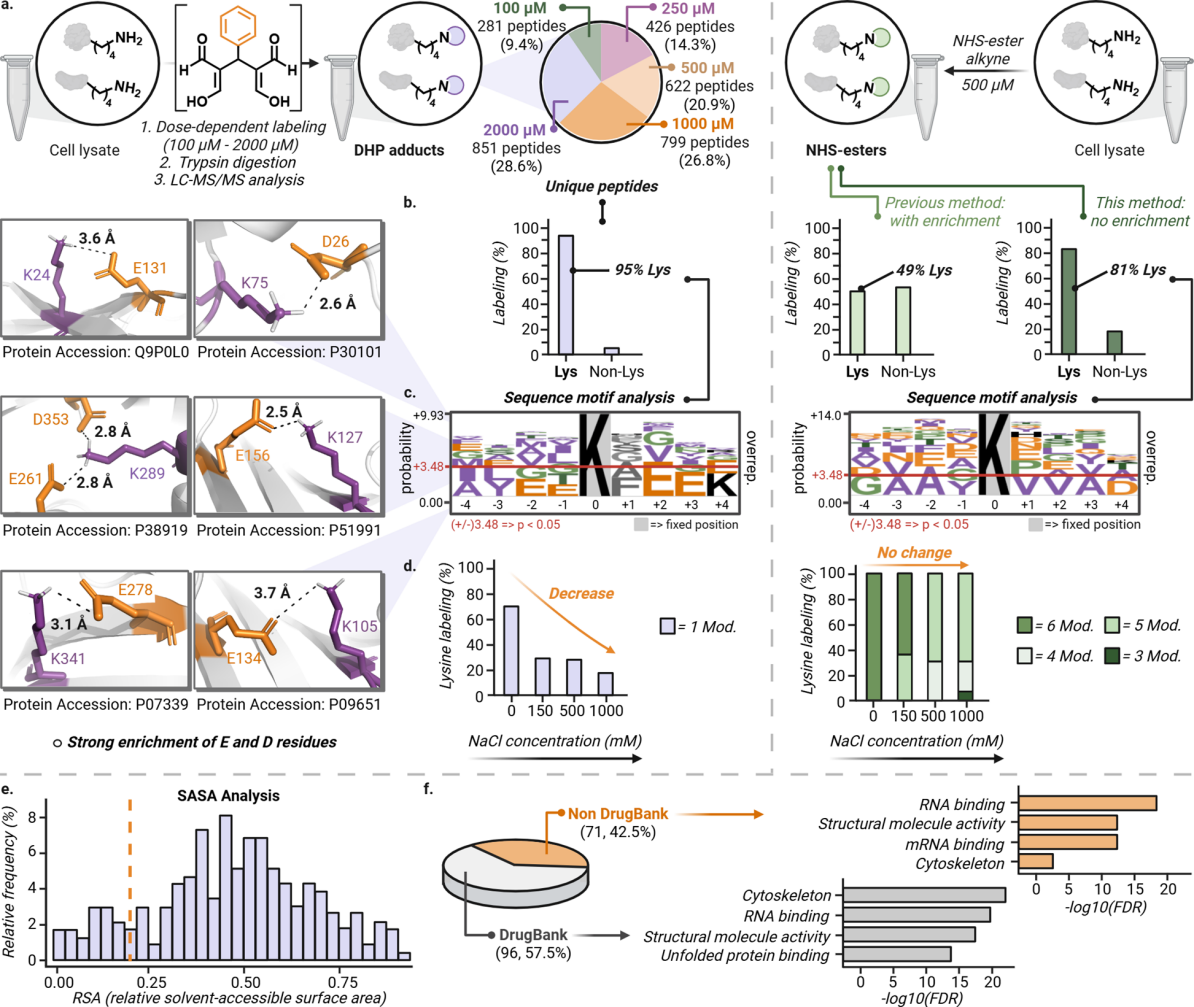
Chemoproteomic profiling of proteome toward the cooperative MDA–benzaldehyde complex compared with a canonical lysine-reactive NHS ester. (a) ABPP workflow in T-47D cell lysates treated with the preformed MDA-benzaldehyde complex (100–2000 μM) or an NHS-ester alkyne (500 μM), followed by side-by-side trypsin digestion and LC–MS/MS analysis. Venn diagrams summarize unique modified peptides across concentrations. (b) Chemoselectivity studies show strong lysine selectivity for cooperative DHP labeling (>95% Lys, negligible off-targets), whereas NHS-ester show broader residue coverage (∼81% Lys, ∼19% non-Lys). (c) Motif analysis of modified lysines reveals that acidic microenvironment enrichment is a unique feature of cooperative DHP labeling, which is not observed in the matched NHS-ester data. (d) Increasing ionic strength (0–1 M NaCl) suppresses aldehyde cooperative labeling of lysozyme chicken lysines (∼67% conversion to <20%), consistent with electrostatic gating, while NHS-ester labeling is only modestly affected under the same conditions. (e) RSA/SASA analysis shows labeled lysines span a broad accessibility range, including many with RSA < 0.25, indicating DHP labeling is not limited to maximally exposed sites. (f) Of the 167-protein cohort, 96 (57.5%) are DrugBank-annotated whereas 71 proteins (42.5%) are non-DrugBank, GO analysis of the detectable landscape highlights enrichment in cytoskeletal and RNA-binding protein classes in both subsets, with particularly strong RNA/mRNA-binding representation among non-DrugBank proteins. The data utilized for analysis of Figure (5a–f) for MDA-benzaldehyde and NHS-ester were acquired with *n* = 2 biological independent samples. Quantitative analysis of biological replicates of MDA-benzaldehyde data (*n* = 2) confirmed high reproducibility (Pearson *r* = 0.91–0.95) and low variance (standard deviation SD < 0.2) across modified lysine sites (Supplementary Figure 19). The workflow demonstrated high technical stability and quantitative reproducibility, supporting the use of this cohort for downstream selectivity and motif analysis. Excel sheet of analysis is included as Supplementary Data 1–7. Source data are available as a Source Data file. Created in BioRender. Raj, M. (2026) https://BioRender.com/id33qrw.

Labeling expanded in a concentration-dependent manner, yielding a high-confidence set of ∼850 peptides and ∼400 proteins (Figure 5a, Supplementary Figure 19). By performing proteome-wide profiling without affinity enrichment, we minimize potential capture-tag biases but restrict our mapping to the detectable proteome landscape accessible by nonenriched LC-MS/MS, such that observed trends predominantly reflect intrinsic chemical selectivity rather than bulk protein abundance. A core cohort of 245 peptides and 167 proteins were reproducibly labeled across all concentrations (Supplementary Figure 19) and within this cohort 98 proteins were modified at a single lysine residue (Supplementary Figure 19), consistent with strong site selectivity shaped by local microenvironment features rather than bulk lysine abundance.

We next asked how many residues are prone to any kind of modification in the cooperative aldehyde platform. To minimize residue-assignment bias, we performed an unrestricted open-modification search allowing the DHP Δ*m* (+196 Da) on any residue, followed by localization scoring. Using the same search framework, we also explicitly screened for mass shifts consistent with plausible reaction intermediates including monoaldehyde adducts (+54 and +88 Da, corresponding to MDA single-addition and benzaldehyde addition) and Intermediate I (+160 Da) but none were detected above background (See Supplementary Data 1–6). To control for false positives arising from unrestricted searches, we applied stringent PSM-level filters (*q*-value ≤ 0.01, Rank = 1, Search Engine Rank = 1, isolation interference ≤30%) prior to chemoselectivity analysis. After filtering, >95% of confidently localized modification sites occurred on lysine residues, with only <5% distributed across other amino acids (Figure 5b). While a flexible model peptide with a free N-terminus showed high conversion to the DHP adduct, we observed no N-terminal modification across complex lysates. This suggests that in the context of folded proteins, the N-terminus lacks the specific electrostatic gating such as the proximal acidic microenvironments identified in our motif analysis required to promote productive DHP assembly (Supplementary Figure 19). To further validate our findings, we performed a head-to-head comparison using a canonical NHS-ester alkyne probe (500 μM) in T-47D cell lysates. Our implementation showed approximately 19% of modifications localized to nonlysine residues (Figure 5a,b, Supplementary Data 7). While this represents higher lysine reactivity than some previous reports (51% nonlysine),^15^ the sequence motif analysis remains the defining differentiator. Both the reported data sets and our matched NHS-ester experiment showed no preference for charged residues, instead displaying a consistent enrichment of small residues like Ala, Val, and Gly (Figure 5c, Supporting Information Figure 19). This confirms that NHS-ester reactivity is relatively independent of a specific chemical microenvironment, whereas the DHP platform uniquely leverages nearby Asp/Glu residues to achieve high-fidelity lysine labeling even at higher concentrations (up to 2 mM). Together, these data are consistent with a mechanistic distinction between cooperative DHP formation and classical acylation. For the cooperative MDA platform, proximal Asp/Glu residues may stabilize and position the lysine ε-ammonium through salt-bridge/hydrogen-bond networks while creating a localized electrostatic environment that promotes productive association of the cooperative aldehyde complex (Figure 5c). Following association, covalent engagement and rapid cyclization would commit the pathway to the chemically stable DHP adduct. In contrast, NHS esters do not require a preorganized electrostatic microenvironment and broadly acylate accessible nucleophiles, resulting in comparatively weaker dependence on local acidic partners. We directly tested the role of electrostatics by modulating ionic strength in a controlled protein system (Figure 5d, Supplementary Figure 20). NaCl was introduced only during the protein-labeling step after preforming the cooperative Intermediate II. With lysozyme chicken, increasing NaCl (0, 150 mM, 500 mM, 1 M) strongly attenuated DHP labeling in a dose-dependent manner (from ∼67% conversion to <20%), consistent with electrostatic screening disrupting a productive association/orientation step (Figure 5d). Under the same salt challenge, an NHS-ester probe continued to modify lysozyme lysines, with only a modest reduction in the number of modified Lys sites (Figure 5d). To further support this electrostatic gating model, we performed lysozyme chicken modification experiments with both a nonionic osmolyte (sucrose) and an alternative ionic salt (Na_2_SO_4_). We observed no significant change in lysine reactivity in the presence of sucrose, whereas Na_2_SO_4_ led to a dose-dependent reduction in modification (Supplementary Figure 20). Rather than a narrow intrinsic p*K*_a_ sweet spot, site reactivity appears to be governed by the local electrostatic neighborhood, where lysines with high apparent p*K*_a_ (>10) are primed for modification by stabilizing acidic microenvironments. These results support the view that cooperative MDA chemistry is more strongly gated by local electrostatic microenvironments than NHS acylation. A key practical consequence of this cooperative mechanism is adduct stability. Whereas Schiff-base adducts are readily reversible upon challenge with competing nucleophiles, the fused DHP product is chemically locked. Accordingly, DHP-labeled proteins remained intact upon challenge with glutathione and hydroxylamine at 37 °C (Supplementary Figure 21). This stability enables durable, noncanonical lysine modification for proteomic tracking and mechanistic interrogation of selective protein labeling under cooperative aldehyde chemistry. To further assess structural determinants, we examined whether DHP formation is governed primarily by solvent accessibility. Mapping DHP-modified lysines onto available structures and quantifying relative solvent-accessible surface area (RSA/SASA) revealed a broad distribution of accessibilities (Figure 5e). Notably, a subset of labeled lysines fell below RSA < 0.25 (Figure 5e), indicating that cooperative labeling is not restricted to maximally exposed sites. These observations are consistent with microenvironment-assisted association and capture, in line with the acidic enrichment and ionic-strength sensitivity described above. Finally, we asked whether the proteome space engaged by cooperative MDA chemistry overlaps with known drug-target space. Within the 167-protein core cohort, 96 proteins (57.5%) are annotated in DrugBank, whereas 71 proteins (42.5%) are non-DrugBank (Figure 5f). Functional analysis of the detectable landscape highlighted cytoskeletal/structural functions and RNA-binding activities across both subsets, with the non-DrugBank fraction showing particularly strong representation of RNA/mRNA binding and structural molecule activity (Figure 5f, Supplementary Figure 22). Thus, cooperative MDA labeling captures a substantial fraction of established drug-target space while also extending into protein classes that are less represented in current drug databases. Collectively, these analyses indicate that cooperative MDA chemistry reports an orthogonal, microenvironment-gated lysine reactivity landscape that is not reducible to solvent exposure alone and spans both canonical and underexplored target space. This selectivity profile complements NHS acylation and supports the use of cooperative aldehyde chemistry for prioritizing functionally privileged lysines and developing more site-selective covalent probes.

## Conclusion

This study defines a cooperative aldehyde reactivity mode in which malondialdehyde (MDA) and monoaldehydes assemble a distinct cooperative intermediate that enables lysine-biased protein modification under aqueous conditions. In contrast to single-aldehyde reactions that often yield heterogeneous or reversible carbonyl adducts, the cooperative pathway channels reactivity into chemically stable dihydropyridine (DHP) conjugates and supports site-resolved mapping across peptides, proteins, and complex lysates. Electronic tuning of the DHP scaffold further enables modular control of photophysical properties, providing red-shifted emissive variants compatible with live-cell imaging contexts.

At the proteome scale, cooperative labeling is nonrandom and converges on a structurally and chemically defined subset of lysines. Sequence and structural analyses reveal enrichment of proximal acidic residues and electrostatically polarized microenvironments near modified sites, consistent with an electrostatically influenced association step contributing to site selectivity. Importantly, solvent accessibility alone does not explain reactivity: RSA/SASA mapping shows that DHP formation spans a broad accessibility range and includes partially occluded lysines (RSA < 0.25), indicating that cooperative labeling is not restricted to maximally exposed ε-amines. Benchmarking against an NHS-ester probe in a controlled protein system, together with comparison to published NHS-ester chemoproteomic data sets, places cooperative aldehyde chemistry in a distinct region of lysine reactivity space that preferentially engages microenvironment-gated lysines.

Finally, proteome-scale annotation indicates that cooperative labeling engages both established and less represented target space. Within the reproducibly labeled core cohort, around half of the proteins are DrugBank-annotated, while another fraction falls outside DrugBank, with enrichment across RNA-binding and structural functions. Together, these results establish cooperative aldehyde chemistry as a tunable platform for interrogating lysine microenvironments and expanding chemoselective lysine labeling beyond accessibility-driven acylation. By linking electrostatic and structural features to cooperative DHP formation, this work provides a practical framework for future studies of metabolite-derived electrophiles and for developing site-selective lysine-targeting probes in complex biological settings.

## Supplementary Material

















## Data Availability

All data supporting the findings of this study are available within the Supporting Information. The mass spectrometry proteomics data generated in this study have been deposited to the ProteomeXchange Consortium via the PRIDE partner repository with the data set identifier PXD067614. Source data are provided as a Source Data file. Supplementary data are provided with this paper.

## Notes

Supplemental Data 1: Peptide Counts for MDA-Benzaldehyde Complex Modification (XLSX)
Supplemental Data 2: Peptide Spectrum Matches (PSMs) for MDA-Benzaldehyde Complex Modification (XLSX)
Supplemental Data 3: Peptide Spectrum Matches (PSMs) for Non-Lysine MDA-Benzaldehyde Complex Modification (XLSX)
Supplemental Data 4: Peptide Spectrum Matches (PSMs) for Intermediate I Side Product (XLSX)
Supplemental Data 5: Peptide Spectrum Matches (PSMs) for Benzaldehyde Addition Side Product (XLSX)
Supplemental Data 6: Peptide Spectrum Matches (PSMs) for MDA Single Addition Side Product (XLSX)
Supplemental Data 7: Peptide Spectrum Matches (PSMs) for NHS Ester Alkyne Modification (XLSX)
Synthesis of the probes, optimization of the reaction conditions, procedure of modification on peptides, intact proteins, cell lysates, chemoselectivity studies, cytotoxicity studies, fluorescence studies, chemoproteomic profiling, and product characterization by NMR, HPLC, LC-MS, MS/MS, and HRMS (PDF)

## Abbreviations

LSF: late-stage functionalization
MAA: malondialdehyde-acetaldehyde
DHP: dihydropyridine
MDA: malondialdehyde
FDR: false discovery rate
