# Supplementary material for: Cooperative Aldehyde Chemistry Maps an Orthogonal Lysine Reactivity Landscape
Source: J Am Chem Soc. 2026 Apr 2;148(14):15125–37. doi: 10.1021/jacs.6c01030 (PMC13088235; doi:10.1021/jacs.6c01030)
Supplement: Supplementary file 8 [file ja6c01030_si_008.pdf]

Supporting Information

Cooperative Aldehyde Chemistry Maps an Orthogonal Lysine Reactivity Landscape

Ana Villalobos Galindo[1#], Pinki Sihag[1#], John M. Talbott[1], Monika Raj[*1]

[1]Department of Chemistry, Emory University, Atlanta, GA, United States, 30322

#These authors contributed equally

Table of Contents

General..2

Supplementary Figure 1. Synthesis of Malondialdehyde (MDA).[2]..4

Experimental..5

Supplementary Figure 2. Reaction Optimization. 5

Supplementary Figure 3. Substrate Scope 10

Supplementary Figure 4. Substrate Scope of MDA-Benzaldehyde Cooperative Complexes....................19

Supplementary Figure 5. Chemoselectivity Studies of MDA-monoaldehyde Cooperative Complexes.....29

Supplementary Figure 6. Substrate Scope of MDA-MDA Cooperative Complexes. 33

Supplementary Figure 7. Chemoselectivity Studies of MDA-monoaldehyde Cooperative Complexes.....45

Supplementary Figure 8. Late-Stage Functionalization of Peptides. 49

Supplementary Figure 9. Flow Cytometry of Ac-QPK-COOH Analogs (4a-4g)..........................56

Supplementary Figure 10. Synthesis of DHP analogs with enhanced conjugation.........................58

Supplementary Figure 11. Absorbance and Emission Spectra of DHP analogs...............................73

Supplementary Figure 12. Quantum Yield of 5b and 5j 76

Supplementary Figure 13. Cytotoxicity of 5j. 77

Supplementary Figure 14. Cellular Imaging of 5j in HeLa cells...78

Supplementary Figure 15. Selective Modification of Proteins Reaction Optimization. 79

Supplementary Figure 16. Lysozyme chicken NHS-ester alkyne. 103

Supplementary Figure 17. Proteins Substrate Scope MDA-Benzaldehyde Complex.......................106

Supplementary Figure 18. Proteins Substrate Scope MDA-MDA Complex. 116

Supplementary Figure 19. Proteomic Analysis. 126

Supplementary Figure 20. Ionic and Non-ionic Strength Studies on Lysozyme Chicken. 136

Supplemental Figure 21: Stability Studies 157

Supplemental Figure 22. GO MF of 167 Protein Cohort and Protein Abundance Distribution...............161

General.

1. General. All commercial materials (Sigma-Aldrich, TCI America, Oakwood Chemical) were used without further purification. All solvents were reagent or HPLC (Fisher) grade. All reactions were performed under air in glass dram vials. Yields refer to chromatographically pure compounds; percent yields were obtained by comparing HPLC peak areas of products and starting materials. HPLC and MS were used to monitor reaction progress, and product elucidation was done using HRMS and NMR.

2. Materials. Fmoc-amino acids, Rink amide resin, 3-[bis(dimethylamino)methyliumyl]-3H-benzotriazol-1-oxide hexafluorophosphate (HBTU), 1-hydroxy-7-azabenzotriazole (HOAt), N,N′-iisopropylcarbodiimide (DIC) was obtained from CreoSalus (Louisville, Kentucky) and N,N-diisopropylethylamine (DIPEA) was obtained from TCI America. Piperidine, trifluoroacetic acid (TFA), were obtained from Alfa Aesar (Ward Hill, Massachusetts). N,N-dimethylformamide (DMF), dichloromethane (DCM), methanol (MeOH), and acetonitrile (ACN) were obtained from VWR (100 Matsonford Road Radnor, Pennsylvania). All small molecules were obtained from commercial vendors. Commercially available proteins: insulin, lysozyme from chicken egg white, lysozyme from human, ubiquitin, apo-transferrin, and aprotinin were obtained from Sigma.

3. Purification. HPLC: Purification of peptide starting materials was performed using high performance liquid chromatography (HPLC) on an Agilent 1100 series HPLC equipped with a C-18 reverse phase column with a particle size of 5 µm. All separations involved a mobile phase of water (solvent A) and acetonitrile (solvent B). The HPLC method used a linear gradient of 0-80% solvent B over 30 minutes at ambient temperature with a flow rate of 1 mL/min. The eluent was monitored by absorbance at 220 nm.

4. Instrumentation and sample analysis. NMR. ^{1}H and ^{13}C spectra were acquired at 25 °C using an Agilent DD2 (400 or 600 MHz) spectrometer with a 3-mm He triple resonance (HCN) cryoprobe. All ^{1}H NMR chemical shifts (δ) were referenced relative to the residual DMSO-d_6 peak at 2.50 ppm or $CDCl_3$ at 7.26 ppm. ^{13}C NMR chemical shifts were referenced to DMSO-d_6 at 39.52 ppm or $CDCl_3$ at 77.16 ppm. ^{13}C NMR spectra were proton decoupled. NMR spectral data are reported as chemical shift (multiplicity, coupling constants (J), integration). Multiplicity is reported as follows: singlet (s), broad singlet (br s), doublet (d), doublet of doublets (dd), doublet of triplets (td), triplet (t) and multiplet (m). Coupling constant (J) in hertz (Hz).

4a. Analytical HPLC. Analytical HPLC chromatography (HPLC) was performed on an Agilent 1100 series HPLC equipped with a 4.6 x 150 mm RediSep Prep C_{18} Aq, 100 Å, 5 µm column. The reaction was monitored by analytical reverse phase HPLC using a gradient of water versus acetonitrile in linear gradients with a constant flow rate of 1 mL/min. Separations involved a mobile phase of 0.1% formic acid in water (solvent A) and 0.1 % formic acid in acetonitrile (solvent B) or mobile phase of water (solvent A) and acetonitrile (solvent B). The eluent was monitored with a detection wavelength of 220 nm.

HPLC Method A: Gradient: 0 to 80 % B (0.1% formic acid in ACN) in 30 min; 80-100 % B in 31-35 min at a flow rate of 1 mL/min.

HPLC Method B: Gradient: 0 to 30 % B (0.1% formic acid in ACN) in 30 min; 30-100 % B in 31-35 min at a flow rate of 0.5 mL/min.

4b. LC/MS. High resolution LC-MS conditions for all purified peptides: Analyses were performed on an ultraperformance LC system (ACQUITY, Waters Corp., USA) coupled with a quadrupole 3 time-of-flight mass spectrometer (Q-ToF Premier, Waters) with electrospray ionization (ESI) in positive mode using Mass lynx software (V4.1) or high-performance LC system (Agilent, 1100 series) coupled with triple quadrupole.

LC-MS (Agilent technologies 6460) with electrospray ionization (ESI) in positive mode using Agilent mass hunter (10.0). Unless otherwise mentioned a sample was injected either onto a C4 column

(Phenomenex Aeris™ 3.6 μm WIDEPORE C_4 200 Å, LC Column 50 x 2.1 mm) with a 400 μL/min flow rate of mobile phase of solution A (90 % H_2O, 10 % acetonitrile and 0.1 % formic acid (FA)) and solution B (95 % acetonitrile, 5 % H_2O, and 0.1 % formic acid) beginning gradient- Time- 0 min 10 % B; 5 min 28 % B; 20 min 38 % B; 22 min 90 % B; C18 column (ACQUITY UPLC BEH 1.7 μm 1x 50 mm) with a 200 μL/min flow rate of mobile phase of solution A (90 % H_2O, 10 % acetonitrile and 0.1 % formic acid) and solution B (90 % acetonitrile, 10 % H_2O, and 0.1 % formic acid) beginning gradient- Time- 1 min 0% B; 1-10 min 100% B for chromatography analysis (or) directly injected with mobile phase 90 % H_2O: 10 % ACN, 0.1% formic acid at 400 μL/min flow rate in ESI positive mode.

4c. HRMS. High resolution MS data were acquired on Thermo Exactive Plus using a heated electrospray source. The solution was infused at a rate of 10-25 μL/min/electrospray using 3.3 KV. The typical settings were Capillary temp 320 °C. S-lens RF level was between 30-80 with an AGC setting of 1 E6. The maximum injection time as set to 50 ms. Spectra were taken at 140,000 resolutions at *m/z* 200 using Tune software and analyze with Thermo's Freestyle software.

4d. MS/MS Analysis. Peptide mapping was performed on ~100 μg of the modified protein after trypsin digestion using the SMART Digest™ Trypsin Kit by Thermo Scientific. The digested sample was analyzed using LC-MS (Agilent AdvanceBio Peptide Plus 2.1 x 250 mm, 2.7 μm, pore size 100 Å) using the following method: 3-40% solvent B (0.1% formic acid in MeCN) over 15 min; then 40-90% solvent B over 15-18 min; then 90% solvent B over 18-20 min, flow rate: 0.4 ml/min. Digested peptides were analyzed using Agilent Bioconfirm (v 10.0) for MS/MS analysis.

4e. Fluorimeter. Fluorescence spectroscopy was measured with an Agilent Cary Eclipse Fluorescence Spectrophotometer. Excitation and emission slits were set to 5 cm unless otherwise stated. The photomultiplier tube (PMT) detector voltage was set to 600 V.

4f. Microscopy. Live cell confocal Imaging was performed on a STELLARIS 8 Leica DMi8 microscope® (63x objective) with fast lifetime contrast (FALCON) module. Samples were excited using a 40 MHz pulsed white light laser tuned to 517 nm. Emitted photons were detected using HyD® X (GaAsP hybrid photocathode).

5. Cell Culture Technique. Cells were maintained at 37 °C and 5% CO_2. HeLa cells were obtained from the Salaita lab in the department of chemistry at Emory University. T-47D cells were obtained from the Spangle lab at Winship Cancer Institute at Emory University. HeLa and T-47D cells were cultured with RPMI 1640 supplemented with 10% (V/V) fetal bovine serum (FBS) and 1% (V/V) penicillin/streptomycin (100 μg/mL).

6. Fmoc Solid-Phase Peptide Synthesis (Fmoc-SPPS).[1] Peptides were synthesized manually on a 0.25 mmol scale using Rink amide resin. Resin was swollen with DCM for 1 h at room temperature. Fmoc was deprotected using 20% piperidine–DMF for 5 min to obtain a deprotected peptide-resin. First, Fmoc protected amino acid (1.25 mmol/5 equiv.) was coupled using HOAt (1.25 mmol/5 equiv.) and DIC (1.25 mm/5 equiv.) in DMF for 15 min at room temperature. Fmoc-protected amino acids (0.75 mmol/3 equiv.) were sequentially coupled on the resin using HBTU (0.75 mmol/3 equiv.) and DIEA (1.5 mmol/6 equiv.) in DMF for 5 min at room temperature. Peptides were synthesized using standard protocols. Peptides were cleaved from the resin using a cocktail of 95:5, trifluoroacetic acid:water for 2 h. The resin was removed by filtration and the resulting solution was concentrated via air flow. The residue was washed with cold ether (3 x 10 mL), precipitating a white solid. Ether was removed via vacuum overnight. The white solid was diluted with ACN/H_2O mixture. The resulting solution was purified by HPLC.

Supplementary Figure 1. Synthesis of Malondialdehyde (MDA).[2]

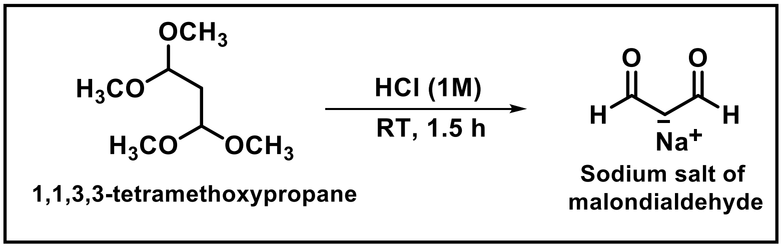

In an oven-dried 250 mL round-bottom flask with stir bar, 15 mL of 1,1,3,3-tetramethoxypropane is added then 8 mL of 1 M HCl solution in water at room temperature. The reaction mixture was stirred for 90 minutes till the two liquid phases became miscible and colorless liquid turned pale yellow. Then the pH of the solution was adjusted to ~7-8 with NaOH solution (5 M in water) to limit polymerization. The intensity of the solution color is dependent on the MDA polymerization. MDA was precipitated by cooling the solution to 0 °C, in 150 mL of acetone for 1 hour. Solid MDA salt was separated *via* vacuum filtration.

^{1}H NMR (400 MHz, D_2O): δ 8.67 – 8.58 (m, 2H), 5.35 – 5.24 (m, 1H).

^{1}H NMR (400 MHz, $CDCl_3$) of **MDA sodium salt**

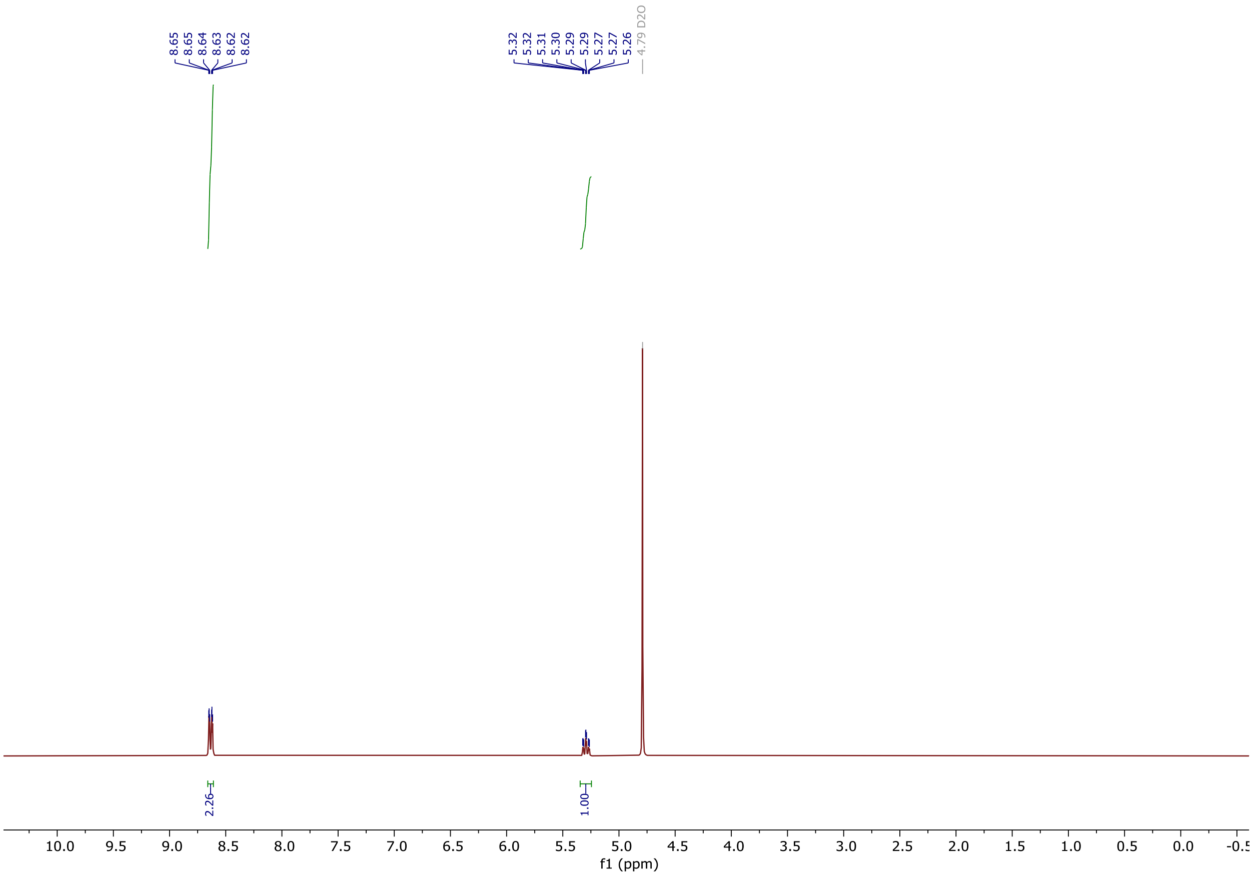

Experimental.

Supplementary Figure 2. Reaction Optimization.

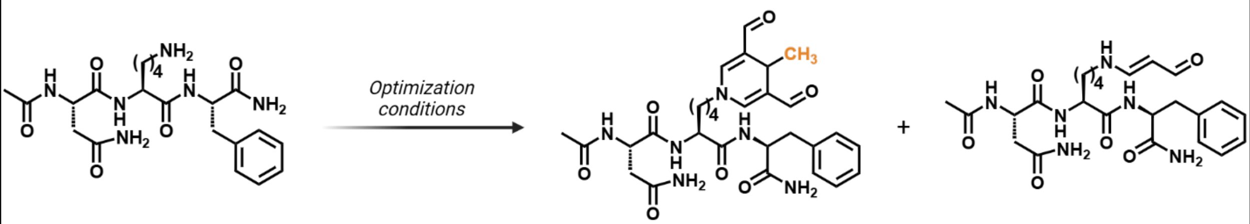

In a one-dram vial, peptide **1a** (1.0 mg, 0.002 mmol, 1 equiv.) was dissolved in 400 µL of sodium acetate buffer (100 mM, pH 4) or sodium phosphate buffer (100 mM, pH 7) followed by the addition of MDA sodium salt (2-25 equiv.) and acetaldehyde (CH_3CHO) (3-30 equiv.) and left to stir at 37 °C or 80 °C for 6-12 hours. Subsequently, the reaction mixture was injected into the HPLC for determining the % conversion of peptide **1a** to the labeled peptides **2a** or **3a** and their mass confirmed with LC-MS. HPLC analysis was carried out utilizing **HPLC Method A** at detection wavelength 220 nm. Refer to optimization table below for reaction conditions. The masses of the products were confirmed with LC-MS.

Optimization table

Entry	MDA (equiv.)	CH_3CHO (equiv.)	Temp. (°C)	pH	Time (h)	% Conversion (2a : 3a)
1	2	3	80	4	6	>99 : 0
2	2	3	80	7	6	90 : 10
**3**	**10**	**20**	**40**	**7**	**12**	**>99 : 0**
**4**	**25**	**30**	**40**	**7**	**12**	**>99 : 0**

Ac-NKF-CONH$_2$ ($C_{21}H_{32}N_6O_5$) peptide 1a. LCMS: *m/z* 449.2507 (calcd $[M+H]^+$ = 449.2507), *m/z* 471.2325 (calcd $[M+Na]^+$ = 471.2326) (HPLC analysis at 220 nm). Retention time in HPLC: 5.4 min.

Ac-NKF-CONH$_2$ ($C_{29}H_{38}N_6O_7$) labeled product 2a. LCMS: *m/z* 605.2697 (calcd $[M+Na]^+$ = 605.2694) (HPLC analysis at 220 nm). Retention time in HPLC: 12.0-12.1 min.

Ac-NKF-CONH$_2$ ($C_{24}H_{34}N_6O_6$) side product 3a. LCMS: *m/z* 525.2421 (calcd $[M+Na]^+$ = 525.2432) (HPLC analysis at 220 nm). Retention time in HPLC: 9.7 min.

HRMS Trace of Peak at 5.4 min

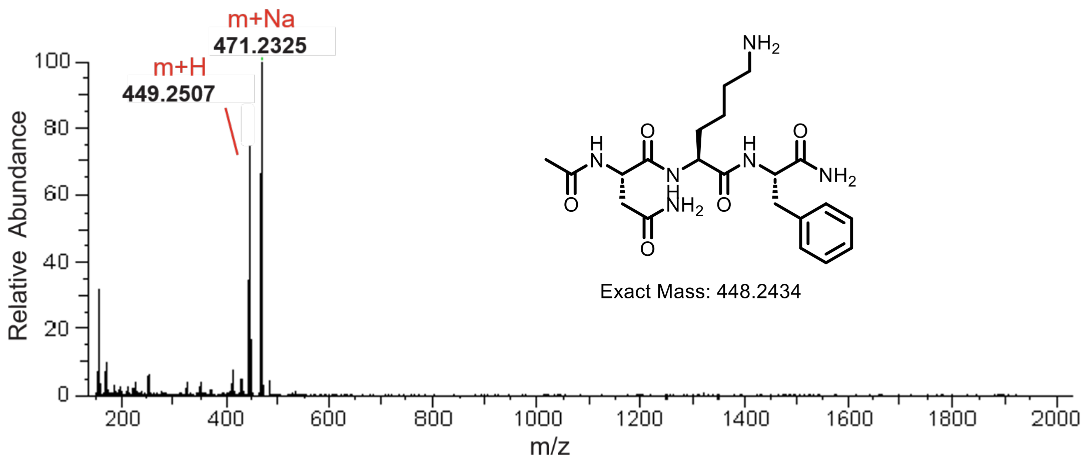

HRMS Trace of Peak at 12.1 min

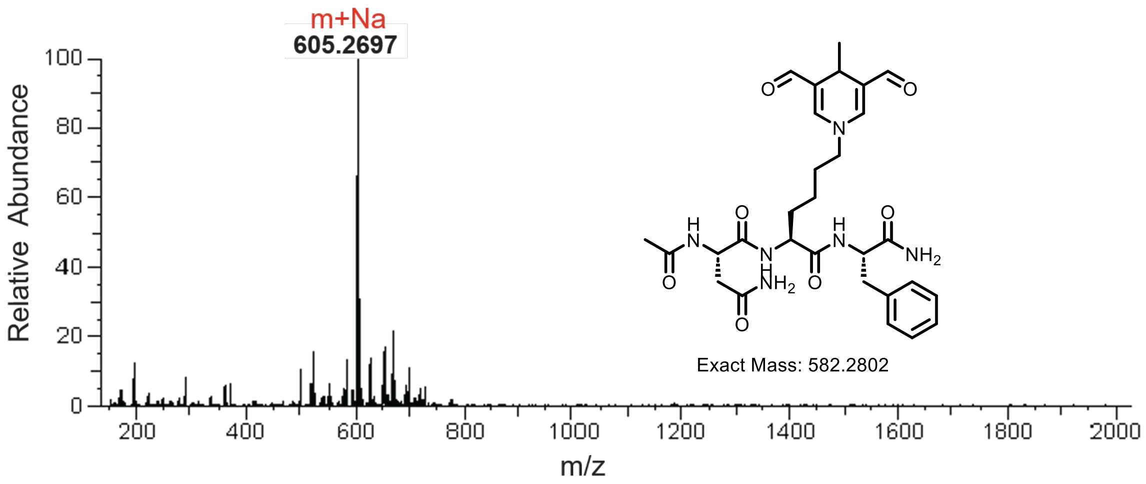

HRMS Trace Peak at 9.7 min

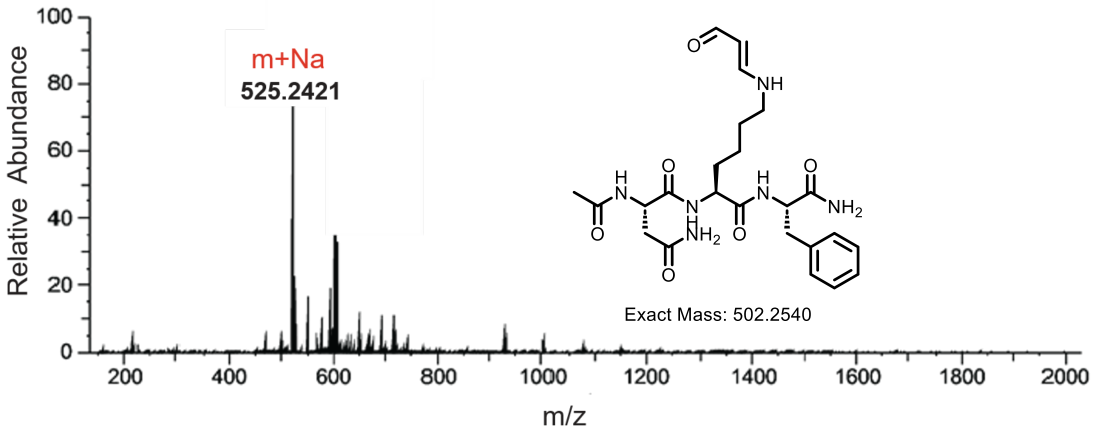

HPLC of Ac-NKF-CONH$_2$ Starting Peptide at 220 nm

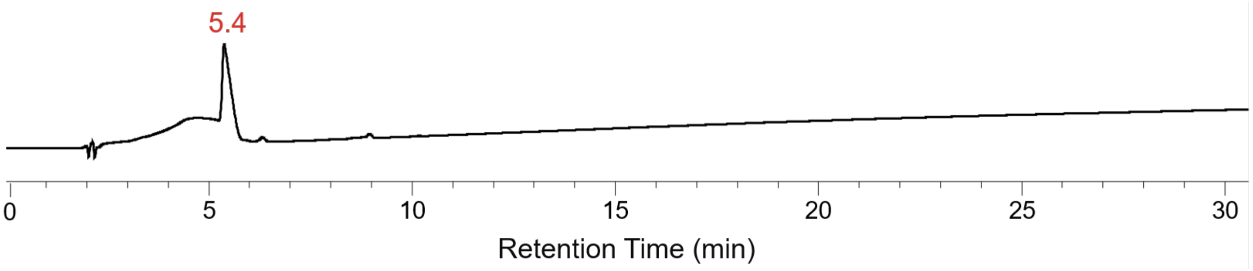

Entry 1.

HPLC Trace of Ac-NKF-CONH$_2$ Reaction Mixture at 220 nm

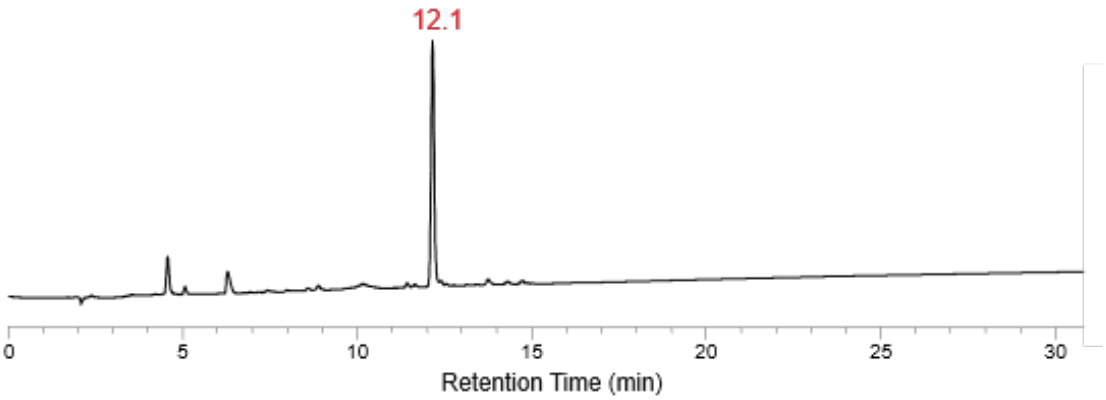

Entry 2.

HPLC Trace of Ac-NKF-CONH$_2$ Reaction Mixture at 220 nm

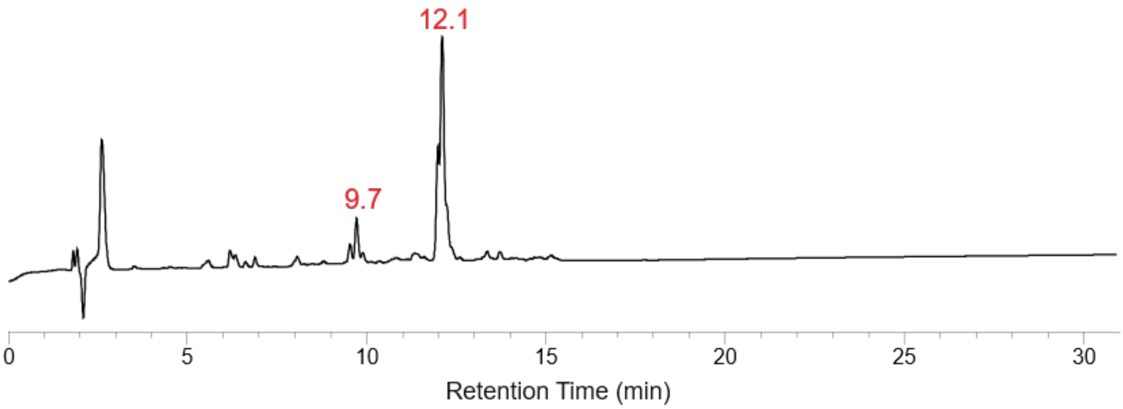

Entry 3.

HPLC Trace of Ac-NKF-CONH₂ Reaction Mixture at 220 nm

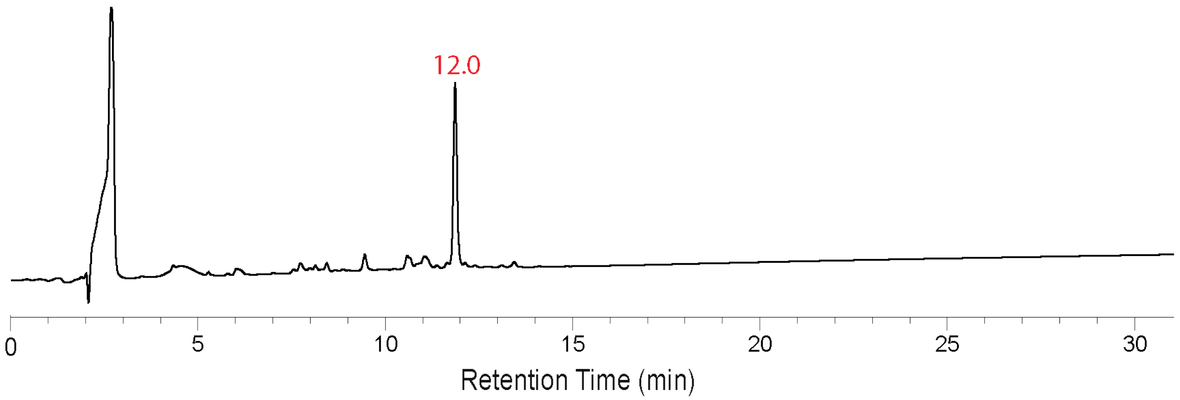

Entry 4.

HPLC Trace of Ac-NKF-CONH₂ Reaction Mixture at 220 nm

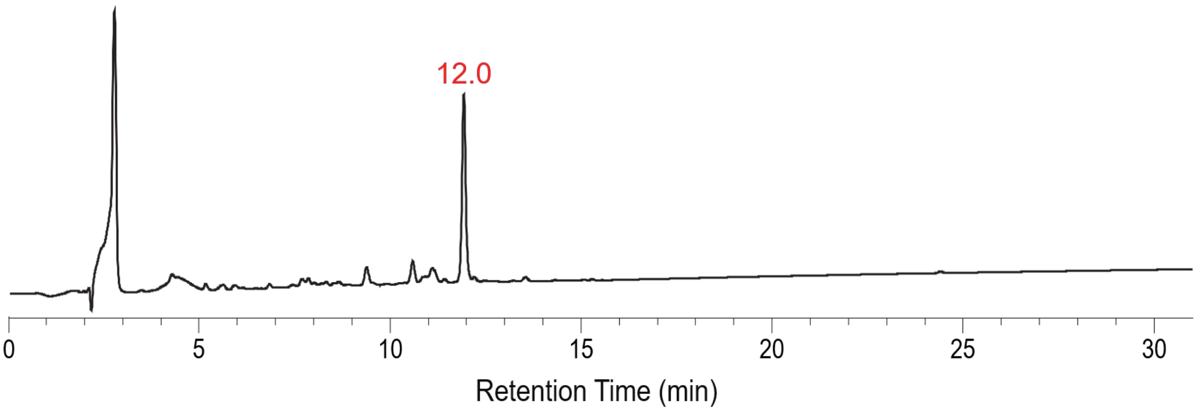

Free N-Terminus.

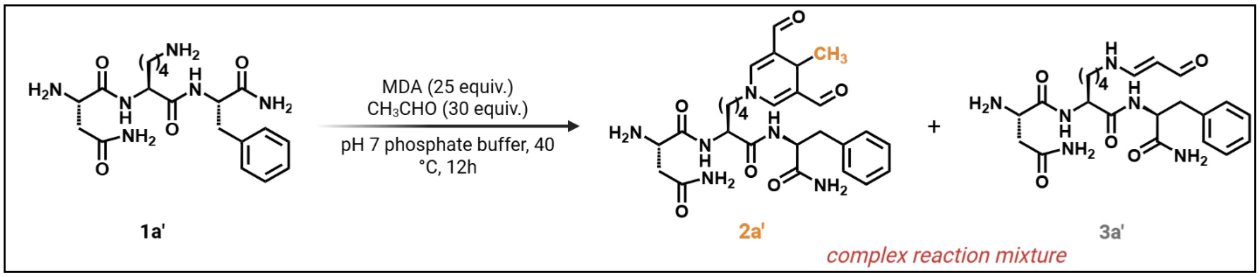

In a one-dram vial, peptide **1a'** (1.0 mg, 0.003 mmol, 1 equiv.) was dissolved in 400 µL of sodium phosphate buffer (100 mM, pH 7) followed by the addition of MDA sodium salt (25 equiv.) and acetaldehyde (CH_3CHO) (30 equiv.) and left to stir at 37 °C for 12 hours. Subsequently, the reaction mixture was injected into the HPLC for determining the % conversion of peptide **1a'** to the labeled peptides **2a'** or **3a'**. HPLC analysis was carried out utilizing **HPLC Method A** at detection wavelength 220 nm. The result was a complex reaction mixture; no products were cleanly identified.

H_2N-NKF-$CONH_2$ ($C_{19}H_{30}N_6O_4$) peptide 1a'. LCMS: *m/z* 407.2400 (calcd $[M+H]^+$ = 407.2401), *m/z* 429.2218 (calcd $[M+Na]^+$ = 429.2226) (HPLC analysis at 220 nm). Retention time in HPLC: 4.3 min.

HPLC of H_2N-NKF-$CONH_2$ Starting Peptide at 220 nm

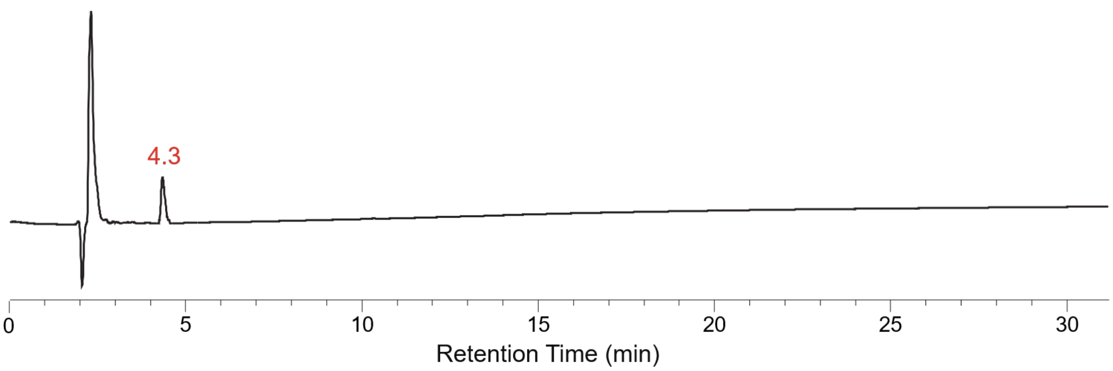

HRMS Trace of Peak at 4.3 min

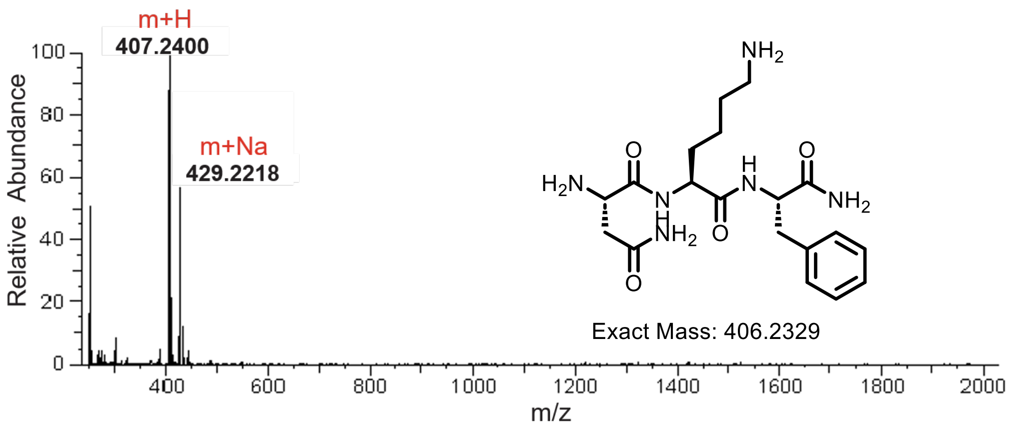

HPLC Trace of H_2N-NKF-$CONH_2$ Reaction Mixture at 220 nm

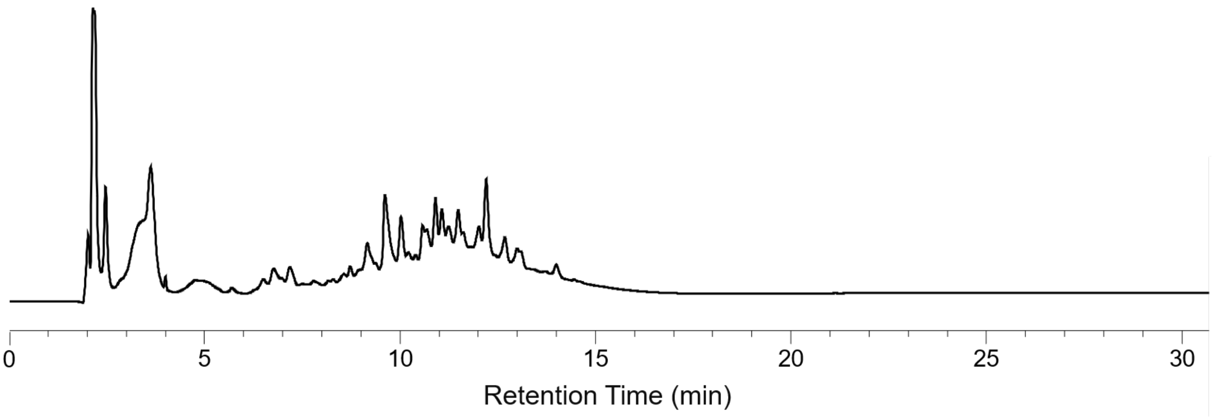

Supplementary Figure 3. Substrate Scope

Peptide 1b.

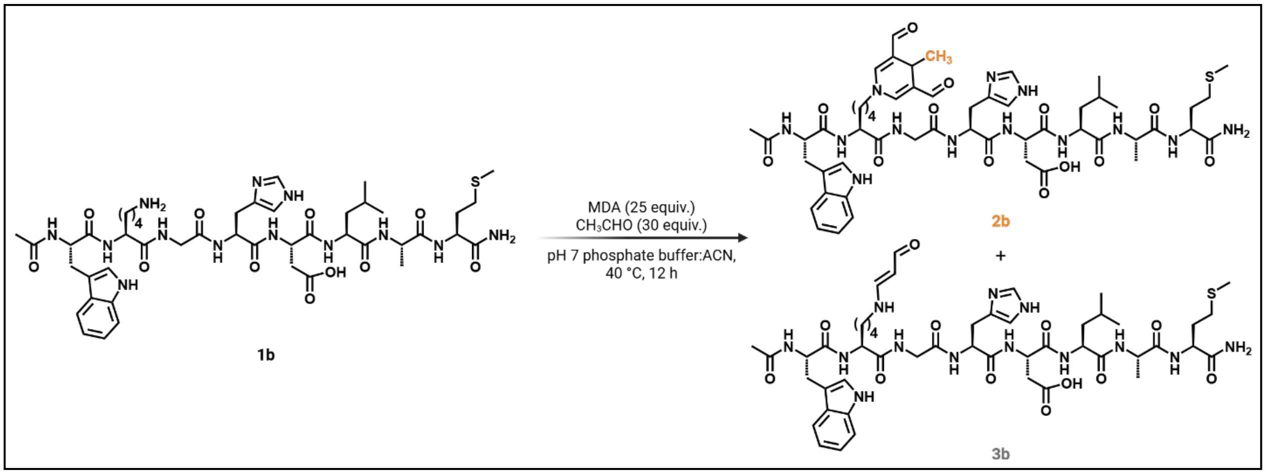

In a one-dram vial, peptide **1b** (Ac-WKGHDLAM-CONH$_2$) (1.0 mg, 0.001 mmol) was dissolved in 300 µL of sodium phosphate buffer (100 mM, pH 7) and 100 µL of acetonitrile followed by the addition of MDA (25 equiv.) and CH_3CHO (30 equiv.) and left to stir at 37 °C for 12 hours. Subsequently, the reaction mixture was injected into the HPLC for determining the % conversion of peptide **1b** to the labeled peptides **2b** or **3b** and their mass confirmed with LC-MS. HPLC analysis was carried out utilizing **HPLC Method A** at detection wavelength 220 nm. The masses of the products were confirmed with LC-MS and compiled below. The conversion was determined to be 84% of **2b** and 16% of **3b**.

Ac-WKGHDLAM-CONH$_2$ ($C_{45}H_{67}N_{13}O_{11}S$) peptide 1b. LCMS: *m/z* 998.4881 (calcd $[M+H]^+$ = 998.4876) *m/z* 499.7474 (calcd $[M+2H/2]^+$ = 499.7475) (HPLC analysis at 220 nm). Retention time in HPLC: 7.8 min

Reaction Mixture:

Ac-WKGHDLAM-CONH$_2$ ($C_{53}H_{73}N_{13}O_{13}S$) labeled product 2b. LCMS: 1132.5236 (calcd $[M+H]^+$ = 1132.5244) (HPLC analysis at 220 nm). Retention time in HPLC: 13.2 min. (84%)

Ac-WKGHDLAM-CONH$_2$ ($C_{48}H_{69}N_{13}O_{12}S$) side product 3b. LCMS: 1052.4979 (calcd $[M+H]^+$ = 1052.4982) (HPLC analysis at 220 nm). Retention time in HPLC: 12.2 min. (16%)

HPLC of Ac-WKGHDLAM-CONH$_2$ Starting Peptide at 220 nm

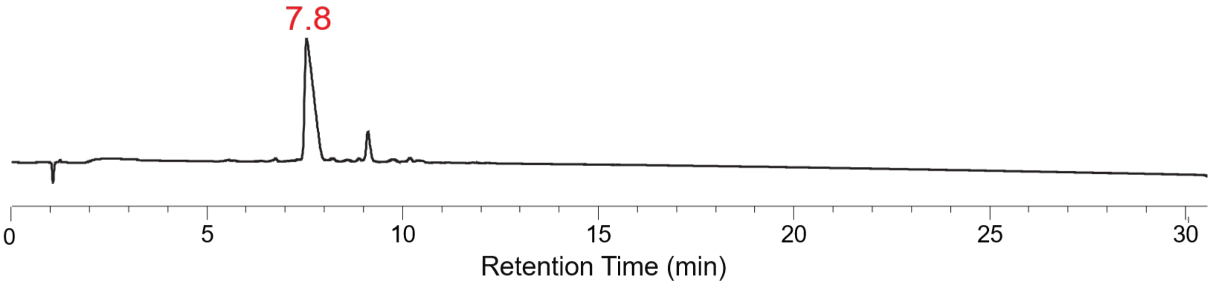

HRMS Trace of Peak at 7.8 min

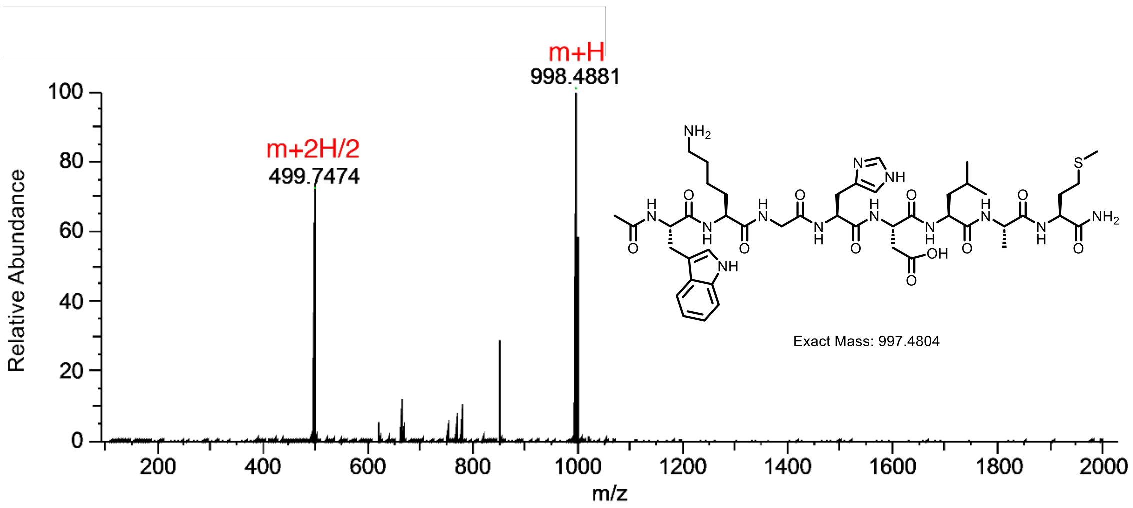

HPLC Trace of Ac-WKGHDLAM-CONH₂ Reaction Mixture at 220 nm

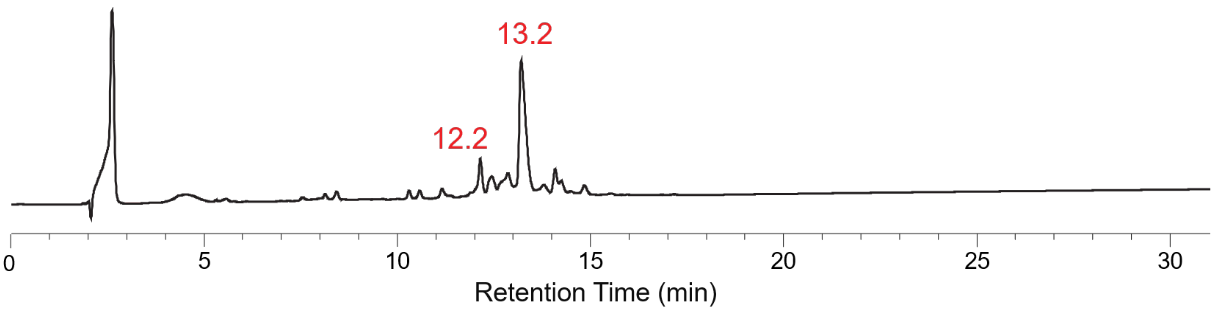

HRMS Trace of Peak at 12.2 min

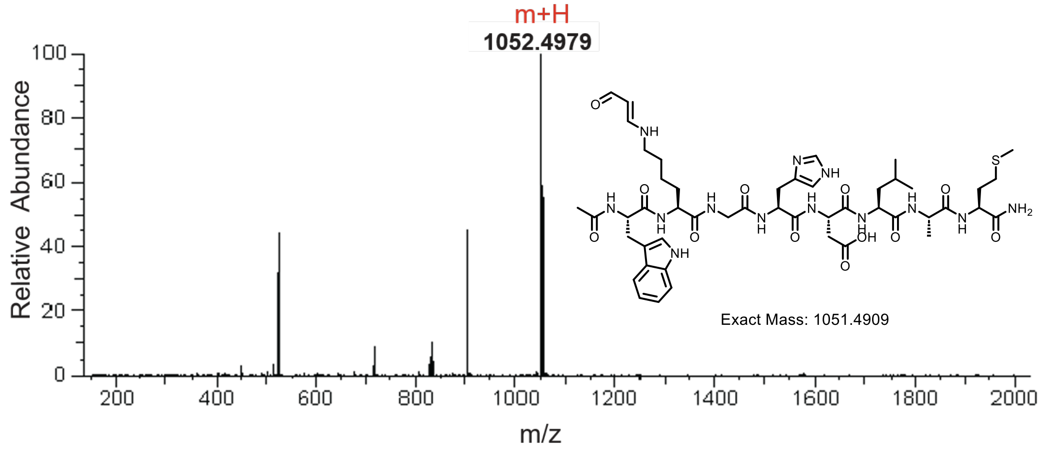

HRMS Trace of Peak at 13.2 min

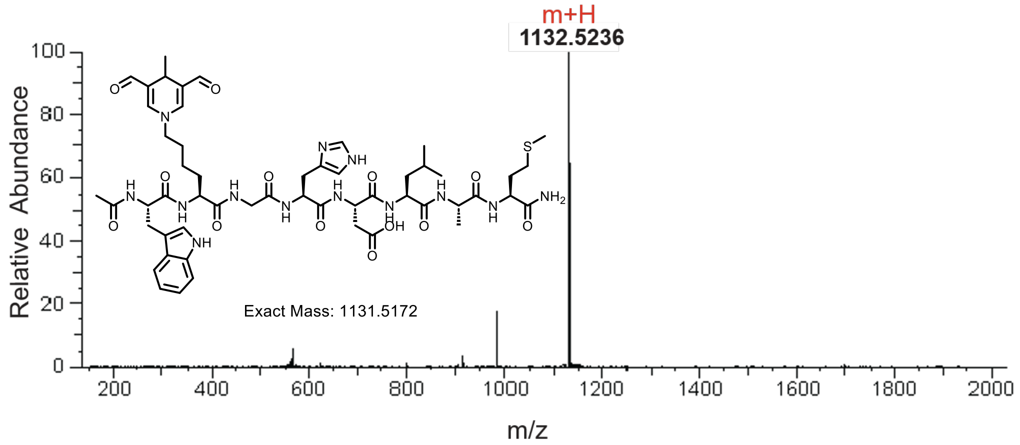

Peptide 1c.

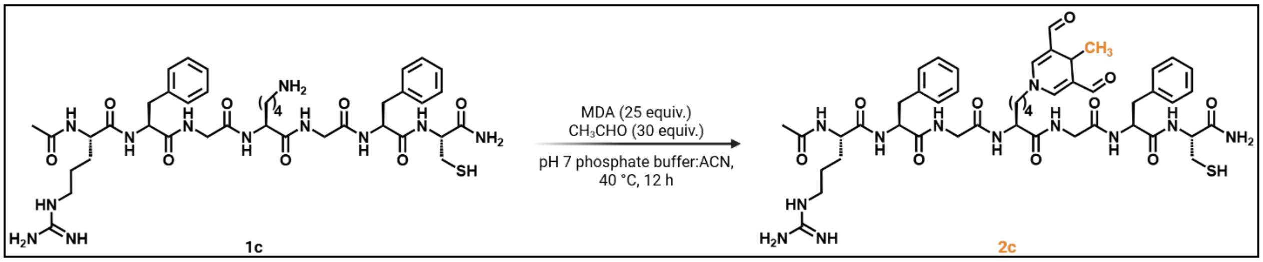

In a one-dram vial, peptide **1c** (Ac-RFGKGFC-CONH$_2$) (1.0 mg, 0.001 mmol) was dissolved in 300 µL of sodium phosphate buffer (100 mM, pH 7) and 100 µL of acetonitrile followed by the addition of MDA sodium salt (25 equiv.) and CH_3CHO (30 equiv.) and left to stir at 37 °C for 12 hours. Subsequently, the reaction mixture was injected into the HPLC for determining the % conversion of peptide **1c** to the labeled peptide **2c** and its mass confirmed with LC-MS. HPLC analysis was carried out utilizing **HPLC Method A** at detection wavelength 220 nm. The mass of the product was confirmed with LC-MS and compiled below. The conversion was determined to be >95% of **2c**.

Ac-RFGKGFC-CONH$_2$ ($C_{39}H_{58}N_{12}O_8S$) peptide 1c. LCMS: *m/z* 855.4291 (calcd [M+H]+ = 855.4294) *m/z* 428.2180 (calcd $[M+2H/2]^+$ = 428.2184) (HPLC analysis at 220 nm). Retention time in HPLC: 8.5 min

Reaction Mixture:

Ac-RFGKGFC-CONH$_2$ ($C_{47}H_{64}N_{12}O_{10}S$) labeled product 2c. LCMS: *m/z* 989.4667 (calcd $[M+H]^+$ = 989.4662) (HPLC analysis at 220 nm). Retention time in HPLC: 12.5-12.8 min. (>95%)

HPLC of Ac-RFGKGFC-CONH$_2$ Starting Peptide at 220 nm

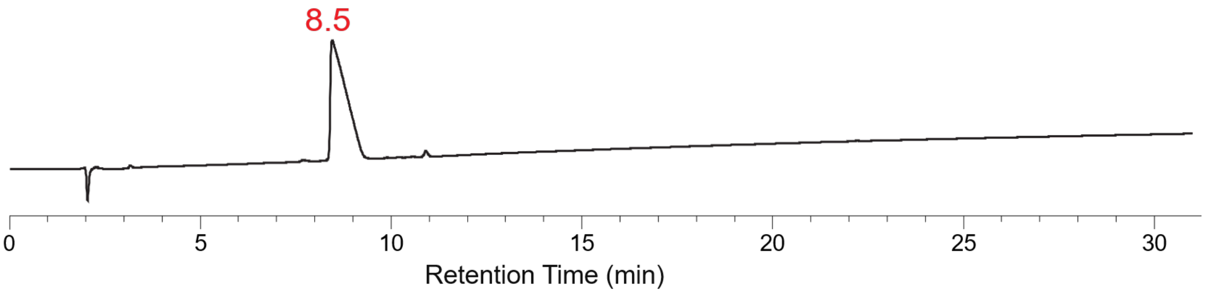

HRMS Trace of Peak at 8.5 min

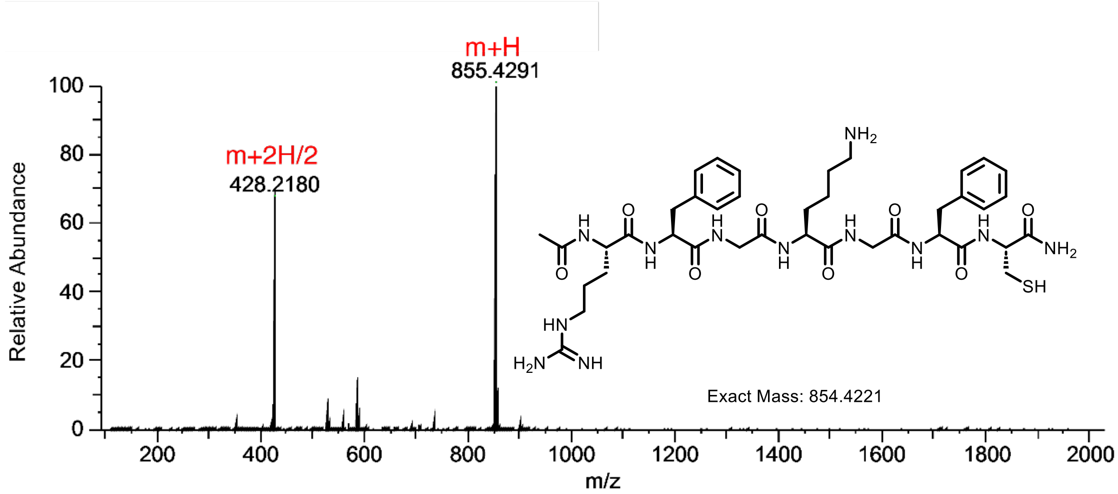

HPLC Trace of Ac-RFGKGFC-CONH₂ Reaction Mixture at 220 nm

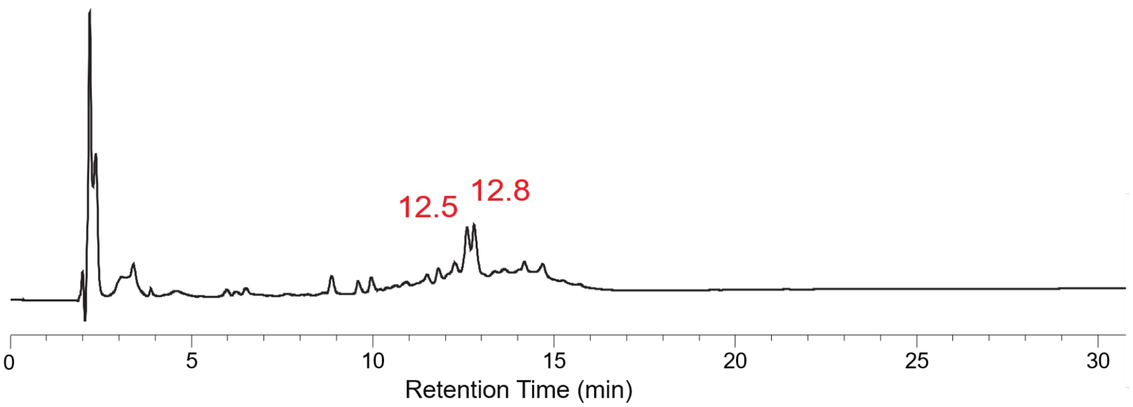

HRMS Trace of Peak at 12.5-12.8 min

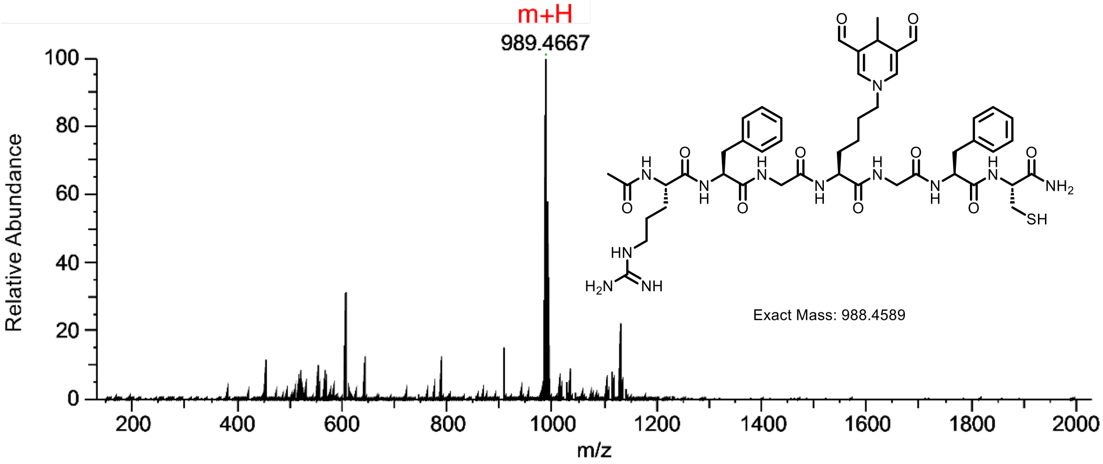

Peptide 1d.

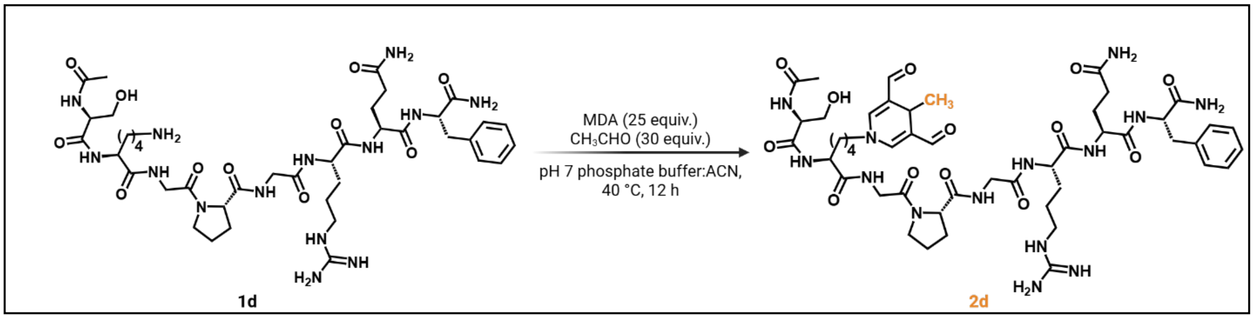

In a one-dram vial, peptide **1d** (Ac-SKGPGRQF-$CONH_2$) (1.0 mg, 0.001 mmol) was dissolved in 300 µL of sodium phosphate buffer (100 mM, pH 7) and 100 µL of acetonitrile followed by the addition of MDA sodium salt (25 equiv.) and CH_3CHO (30 equiv.) and left to stir at 37 °C for 12 hours. Subsequently, the reaction mixture was injected into the HPLC for determining the % conversion of peptide **1d** to the labeled peptide **2d** and its mass confirmed with LC-MS. HPLC analysis was carried out utilizing **HPLC Method A** at detection wavelength 220 nm. The mass of the product was confirmed via LC-MS. The conversion was determined to be >95% of **2d**.

Ac-SKGPGRQF-$CONH_2$ ($C_{40}H_{64}N_{14}O_{11}$) peptide 1d. LCMS: *m/z* 917.4953 (calcd $[M+H]^+$ = 917.4952) *m/z* 459.2511 (calcd $[M+2H/2]^+$ = 459.2513) (HPLC analysis at 220 nm). Retention time in HPLC: 6.8 min

Reaction Mixture:

Ac-SKGPGRQF-$CONH_2$ ($C_{48}H_{70}N_{14}O_{13}$) labeled product 2d. LCMS: *m/z* 1051.5326 (calcd $[M+H]^+$ = 1051.5320), (HPLC analysis at 220 nm). Retention time in HPLC: 10.1 min (>95%)

HPLC of Ac-SKGPGRQF-$CONH_2$ Starting Peptide at 220 nm

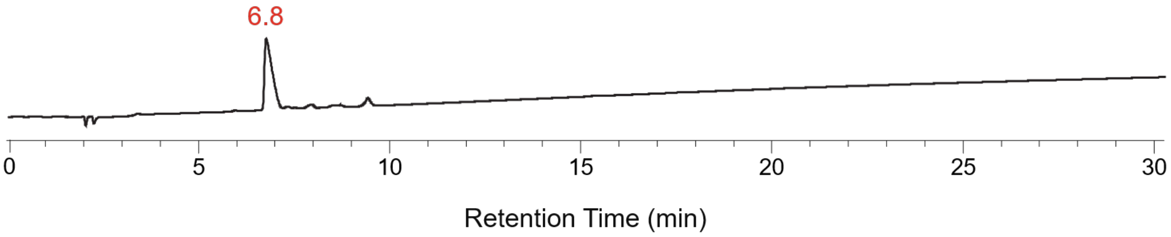

HRMS Trace of Peak at 6.8 min

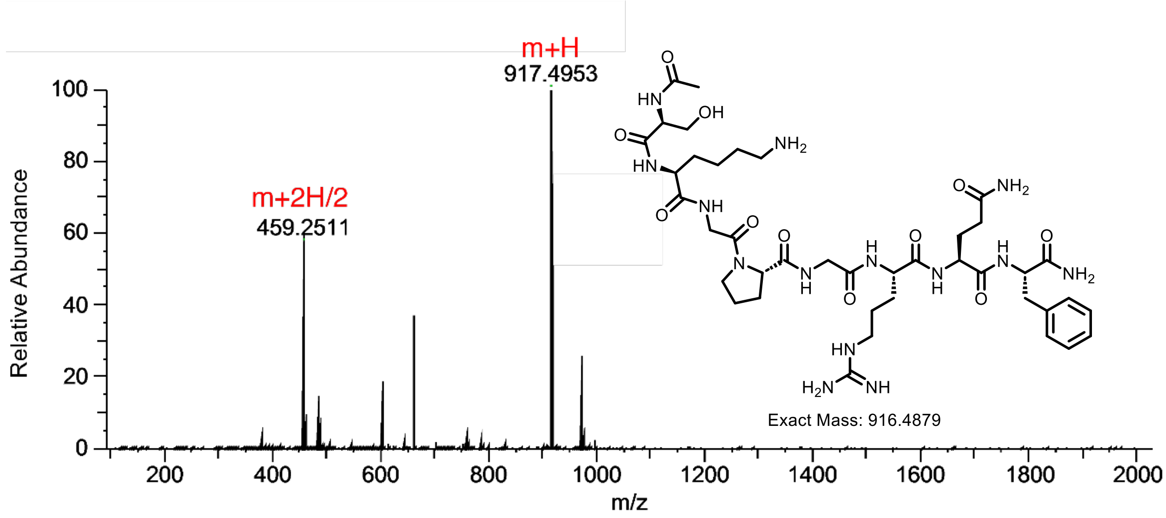

HPLC Trace of Ac-SKGPGRQF-CONH$_2$ Reaction Mixture at 220 nm

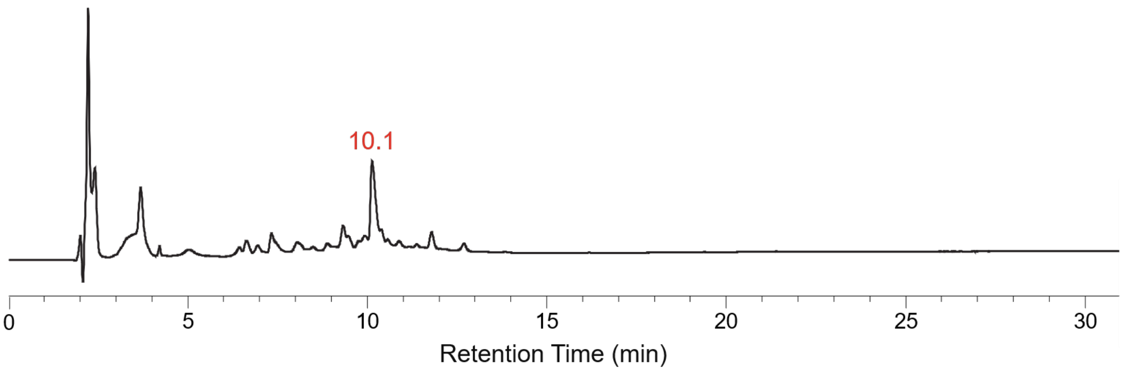

HRMS Trace of Peak at 10.1 min

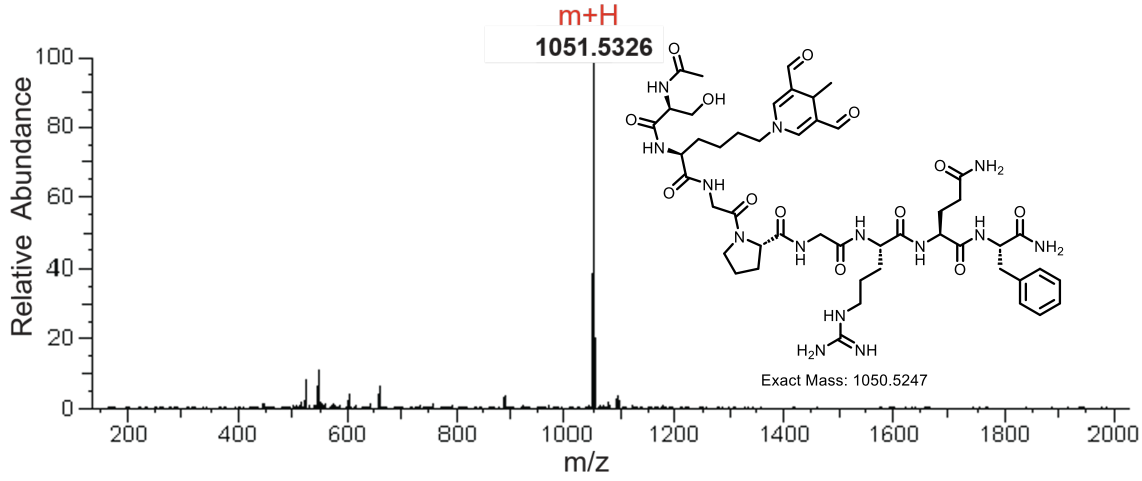

Peptide 1e.

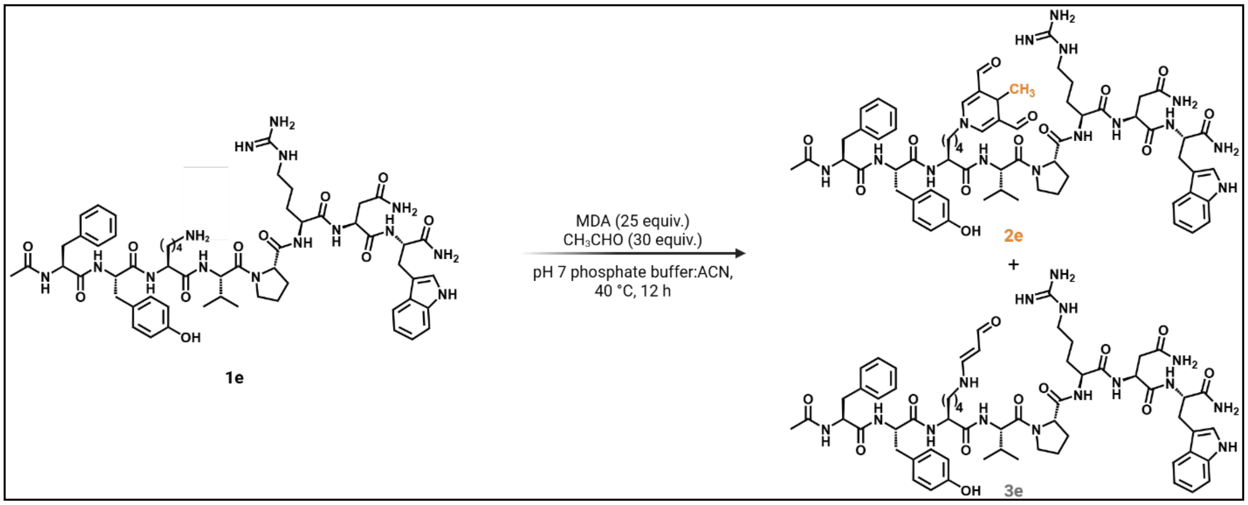

In a one-dram vial, peptide **1e** (Ac-FYKVPRNW-CONH$_2$) (1.0 mg, 0.0009 mmol) was dissolved in 300 µL of sodium phosphate buffer (100 mM, pH 7) and 100 µL of acetonitrile followed by the addition of MDA sodium salt (25 equiv.) and CH_3CHO (30 equiv.) and left to stir at 37 °C for 12 hours. Subsequently, the reaction mixture was injected into the HPLC for determining the % conversion of peptide **1e** to the labeled peptides **2e** or **3e** and their mass confirmed with LC-MS. HPLC analysis was carried out utilizing **HPLC Method A** at detection wavelength 220 nm. The masses of the products were confirmed with LC-MS and compiled below. The conversion was determined to be 73% of **2e** and 13% of **3e**.

Ac-FYKVPRNW-CONH$_2$ ($C_{57}H_{79}N_{15}O_{11}$) peptide 1e. LCMS: *m/z* 1150.6161 (calcd $[M+H]^+$ = 1150.6156), *m/z* 575.8115 (calcd $[M+2H/2]^+$ = 575.8120), (HPLC analysis at 220 nm). Retention time in HPLC: 12.1 min

Reaction Mixture:

Ac-FYKVPRNW-CONH$_2$ ($C_{57}H_{79}N_{15}O_{11}$) peptide 1e. LCMS: *m/z* 1150.6163 (calcd $[M+H]^+$ = 1150.6156), *m/z* 575.8117 (calcd $[M+2H/2]^+$ = 575.8120), (HPLC analysis at 220 nm). Retention time in HPLC: 11.9 min. (14%)

Ac-FYKVPRNW-CONH$_2$ ($C_{65}H_{85}N_{15}O_{13}$) labeled product 2e. LCMS: *m/z* 1284.6532 (calcd [M+H]+ = 1284.6524) (HPLC analysis at 220 nm). Retention time in HPLC: 14.4 min. (73%)

Ac-FYKVPRNW-CONH$_2$ ($C_{60}H_{81}N_{15}O_{12}$) side product 3e. LCMS: 1204.6262 (calcd $[M+H]^+$ = 1204.6262), *m/z* 602.8167 (calcd $[M+2H/2]^+$ = 602.8168) (HPLC analysis at 220 nm). Retention time in HPLC: 13.4 min. (13%)

HPLC of Ac-FYKVPRNW-CONH₂ Starting Peptide at 220 nm

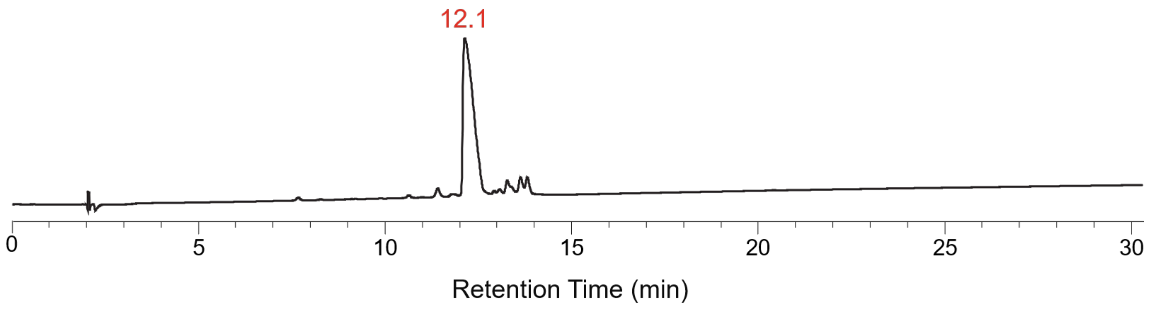

HRMS Trace of Peak at 12.1 min

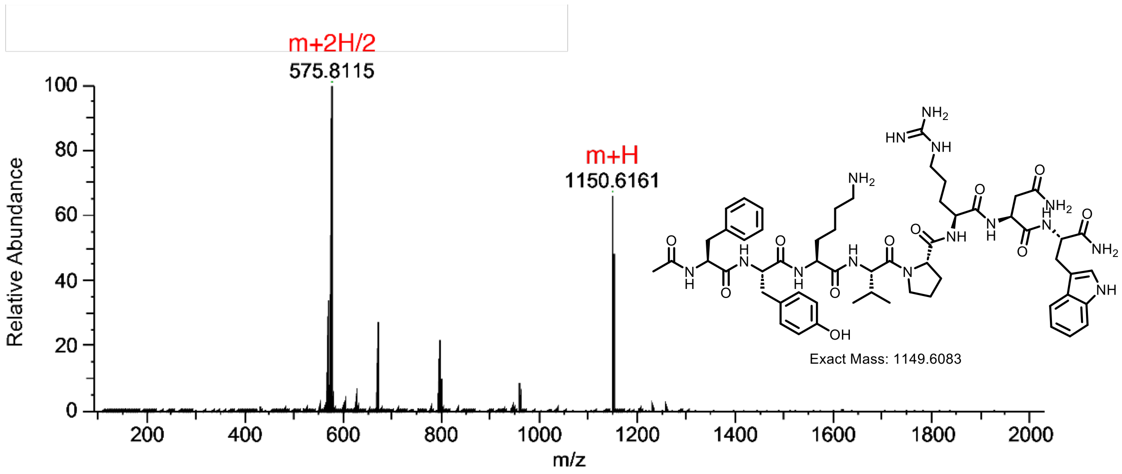

HPLC Trace of Ac-FYKVPRNW-CONH₂ Reaction Mixture at 220 nm

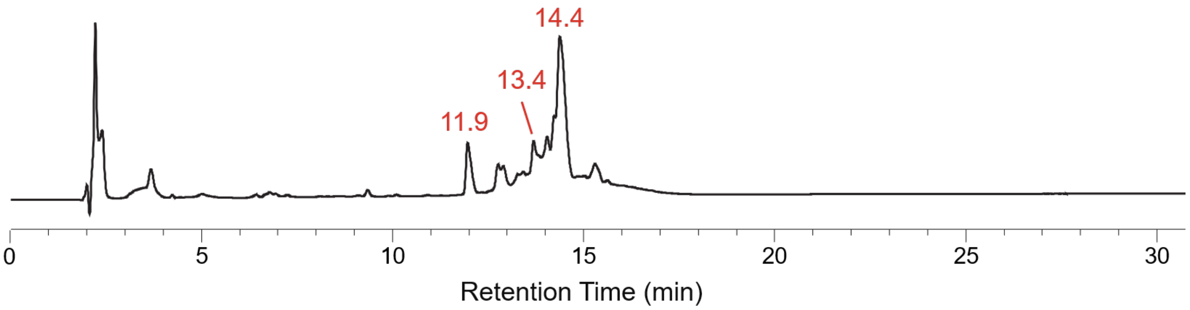

HRMS Trace of Peak at 11.9 min

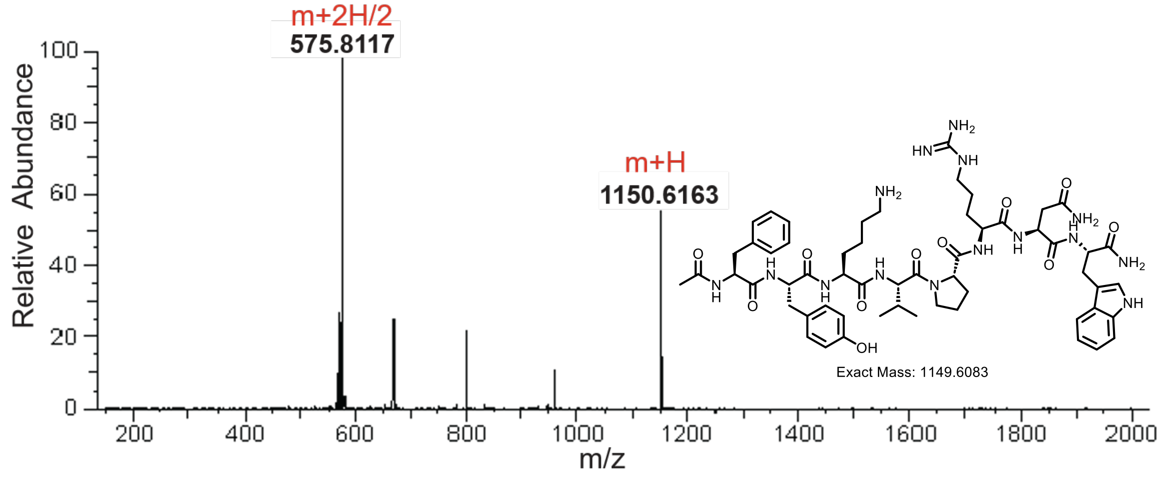

HRMS Trace of Peak at 13.4 min

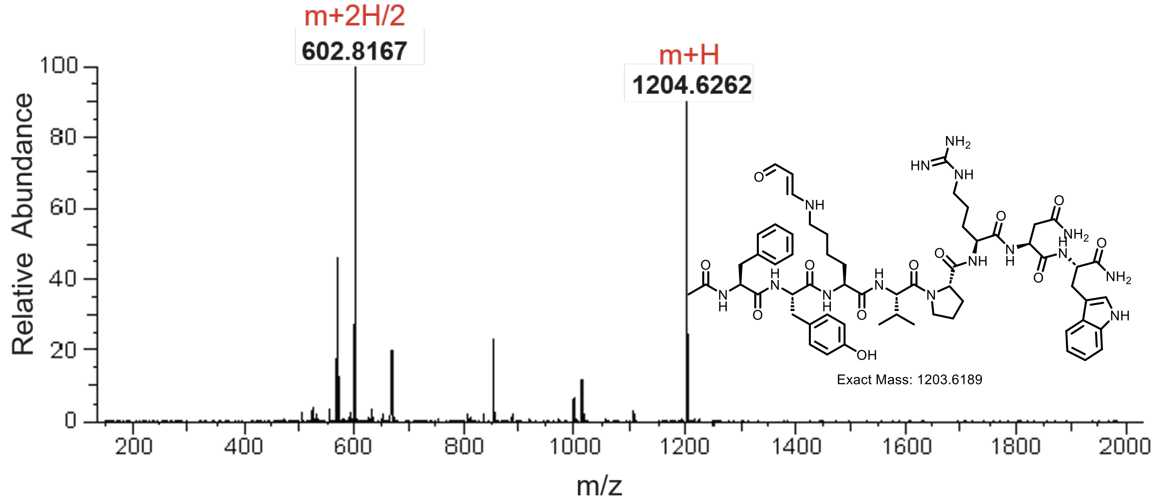

HRMS Trace of Peak at 14.4 min

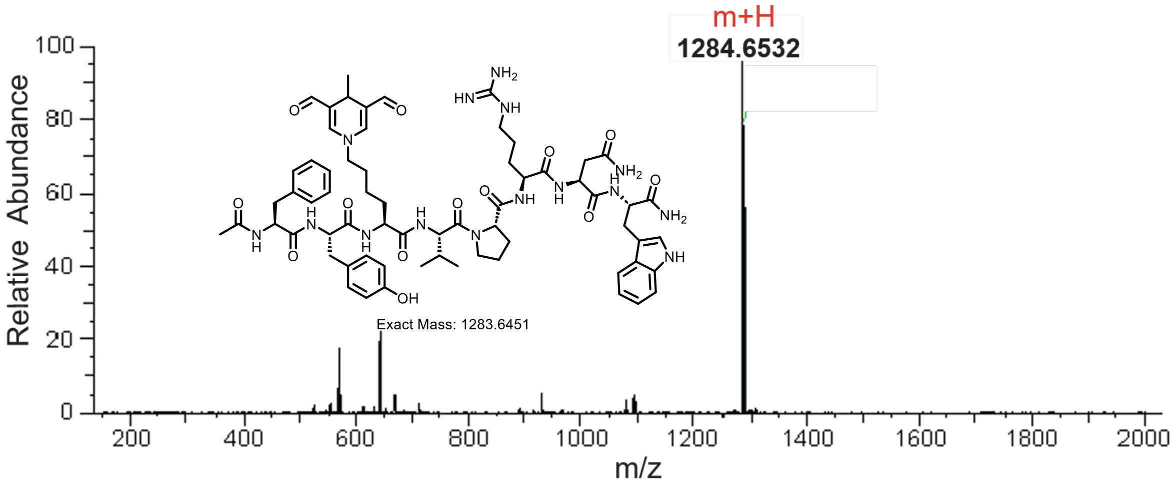

Supplementary Figure 4. Substrate Scope of MDA-Benzaldehyde Cooperative Complexes.

Peptide 1f (optimized conditions)

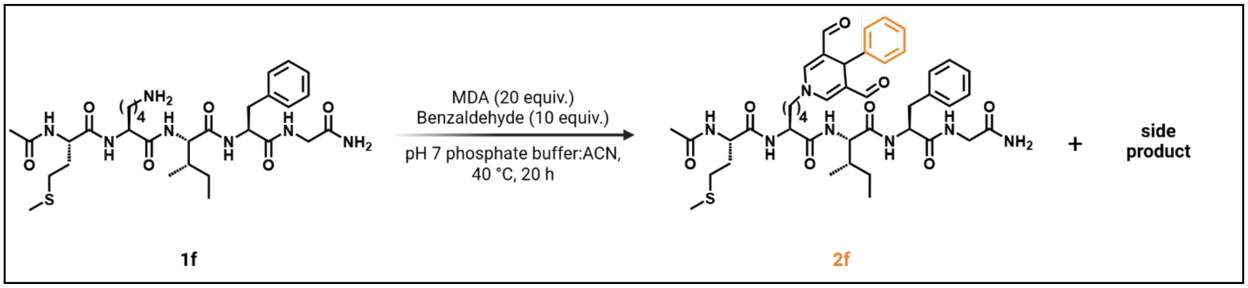

In a one-dram vial, peptide **1f** (Ac-MKIFG-CONH$_2$) (1.0 mg, 0.002 mmol) was dissolved in 300 µL of sodium phosphate buffer (100 mM, pH 7) and 100 µL of acetonitrile followed by the addition of MDA (20 equiv.) and benzaldehyde (10 equiv.) and left to stir at 37 °C for 12 hours. Subsequently, the reaction mixture was injected into the HPLC for determining the % conversion of peptide **1f** to the labeled peptide **2f** and its mass confirmed with LC-MS. HPLC analysis was carried out utilizing **HPLC Method A** at detection wavelength 220 nm. The masses of the products were confirmed with LC-MS and compiled below. The conversion was determined to be 52% of **2f** and 48% of unconfirmed side product.

Ac-MKIFG-CONH$_2$ ($C_{30}H_{49}N_7O_6S$) peptide 1f. LCMS: *m/z* 636.3527 (calcd $[M+H]^+$ = 636.3538) (HPLC analysis at 220 nm). Retention time in HPLC: 11.2 min

Reaction Mixture:

Ac-MKIFG-CONH$_2$ ($C_{43}H_{57}N_7O_8S$) labeled product 2f. LCMS: *m/z* 832.4064 (calcd $[M+H]^+$ = 832.4062), *m/z* 854.3873 (calcd $[M+Na]^+$ = 854.3882) (HPLC analysis at 220 nm). Retention time in HPLC: 15.7 min. (52%)

Ac-MKIFG-CONH$_2$ side product (unknown). LCMS: *m/z* 804.5490 (HPLC analysis at 220 nm). Retention time in HPLC: 14.3 min. (48%)

HPLC of Ac-MKIFG-CONH$_2$ Starting Peptide at 220 nm

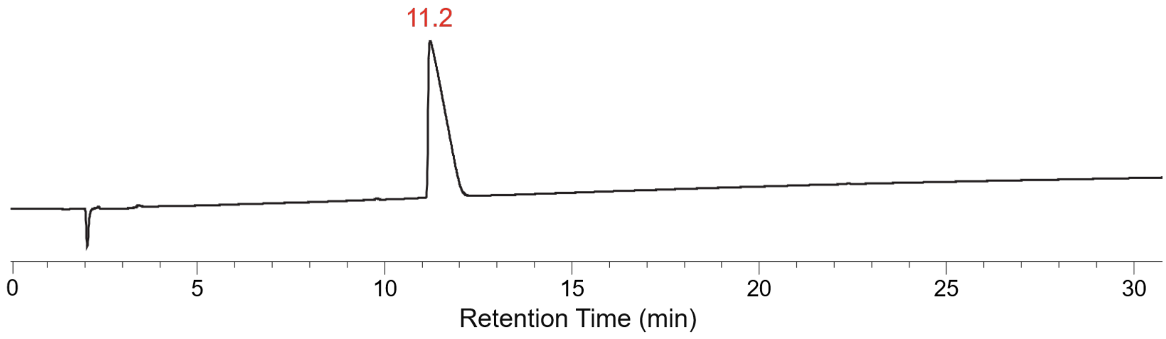

HRMS Trace of Peak at 11.2 min

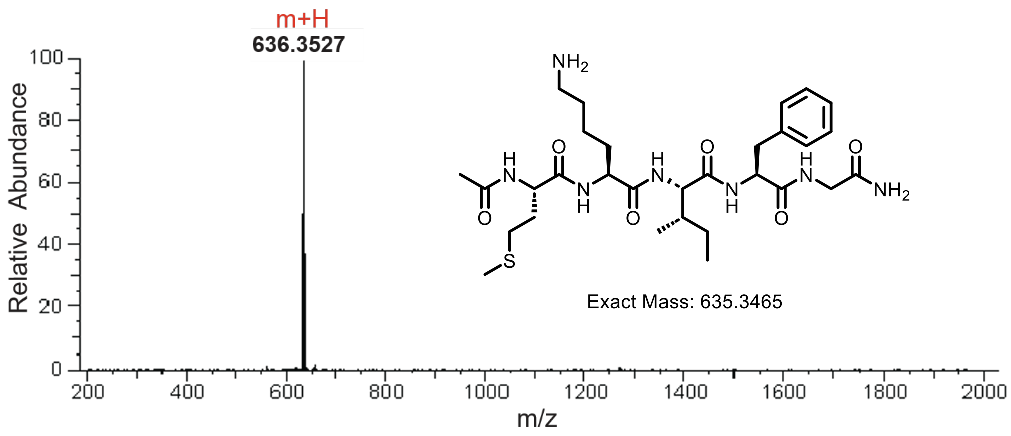

HPLC of Ac-MKIFG-CONH₂ Reaction Mixture at 220 nm

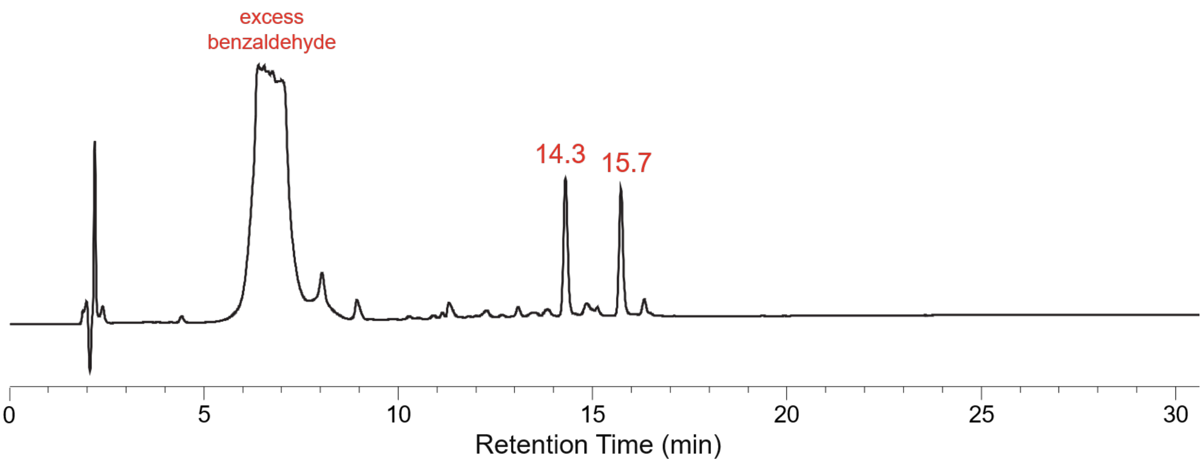

HRMS Trace of Peak at 14.3 min

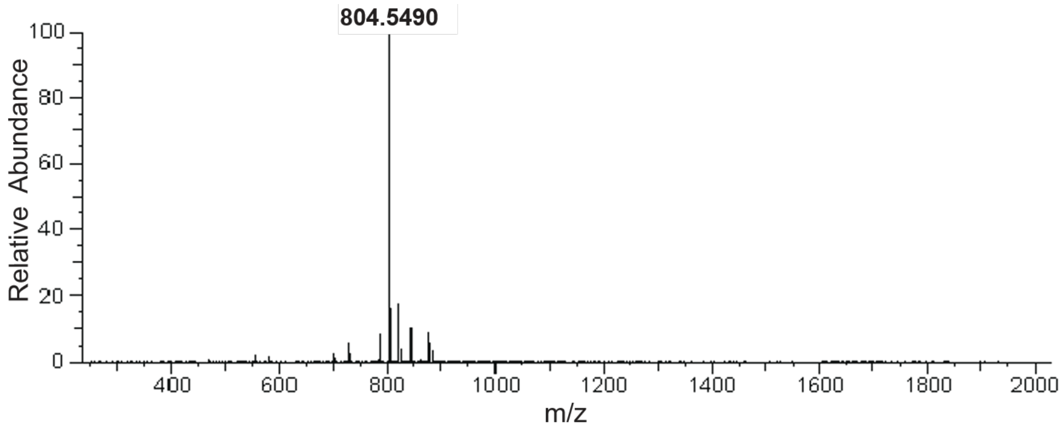

HRMS Trace of Peak at 15.7 min

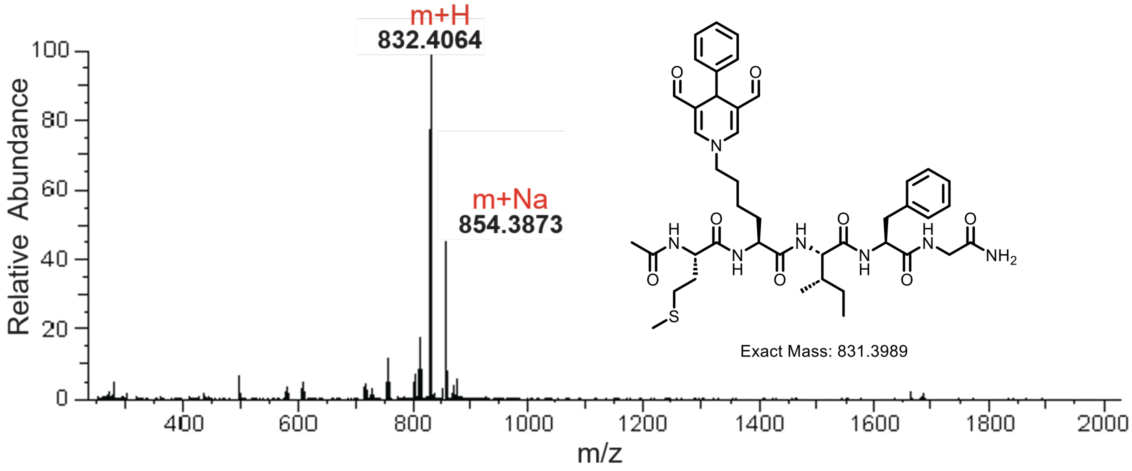

Pre-formed Intermediate Synthesis (MDA-benzaldehyde).

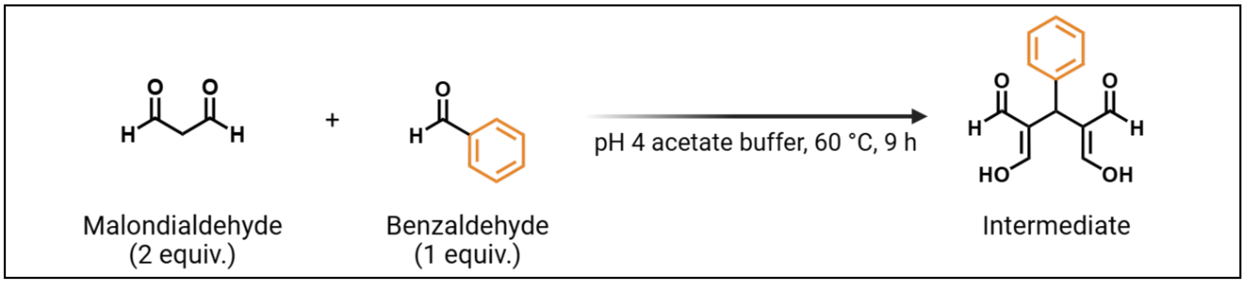

In a round bottom flask MDA (240 mg, 3.33 mmol) was dissolved in pH 4 acetate buffer (5 mL). Then, benzaldehyde (177 mg, 1.67 mmol) was added, and the reaction was left to stir at 60 °C for 9 hours. Reaction completion was determined when benzaldehyde was consumed. After completion, the excess solvent was removed via a speedvac vacuum concentrator. Subsequently, the reaction mixture was redissolved in 1:1 H_2O:ACN and injected into the HPLC for purification. HPLC analysis was carried out utilizing **HPLC Method A** at detection wavelength 220 nm. The mass of the product was confirmed with LC-MS and compiled below. The conversion was determined to be >95% of the intermediate.

MDA-Benzaldehyde Intermediate ($C_{13}H_{12}O_4$). LCMS: *m/z* 233.0808 (calcd $[M+H]^+$ = 233.0808) (HPLC analysis at 220 nm). Retention time in HPLC: 5.9 min

HPLC of Purified MDA-Benzaldehyde Intermediate at 220 nm

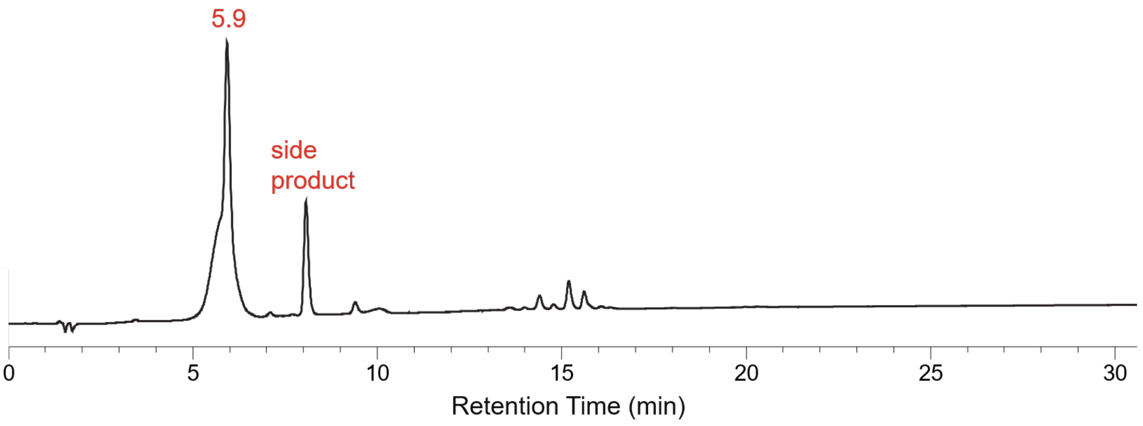

HRMS Trace of peak at 5.9 min

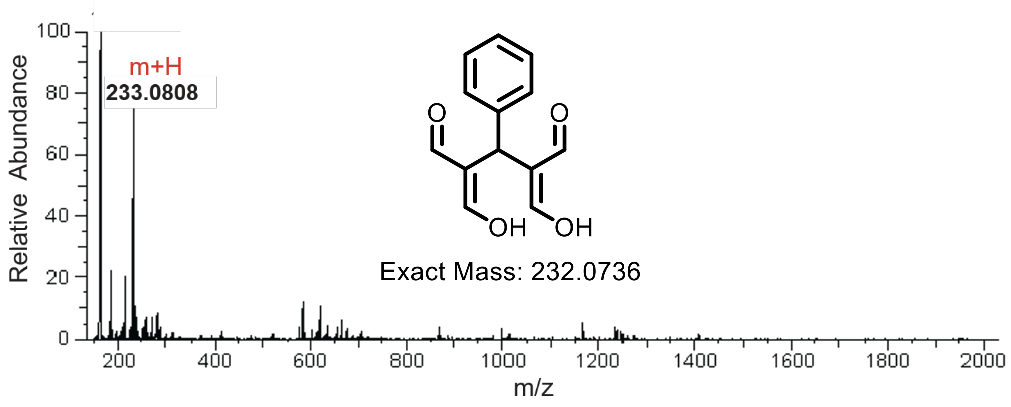

Pre-formed Intermediate Synthesis (MDA-MDA and MDA-acetaldehyde).

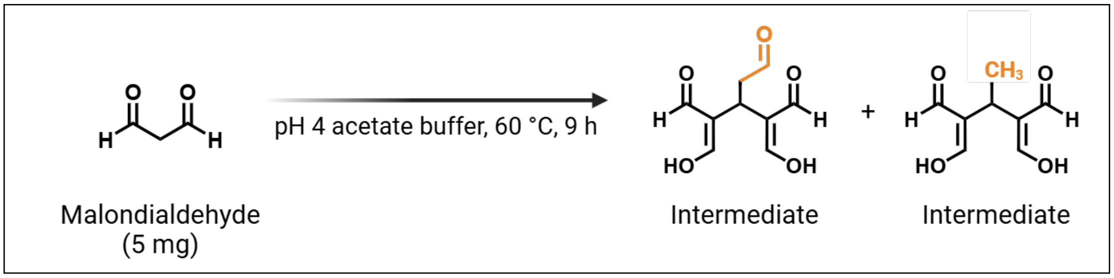

In a one-dram vial, MDA (5 mg), was dissolved in pH 4 acetate buffer (500 µL), and the reaction was left to stir at 60 °C for 9 hours. After which, the mixture was stored in dry ice and immediately injected into the HRMS for characterization.

MDA-MDA Intermediate ($C_9H_{10}O_5$). LCMS: *m/z* 199.0570 (calcd $[M+H]^+$ = 199.0601), *m/z* 237.0724 (calcd $[M+K]^+$ = 237.0160).

MDA-acetaldehyde Intermediate ($C_8H_{12}O_5$). LCMS: *m/z* 189.0540 (calcd $[M+H_2O]^+$ = 189.0757).

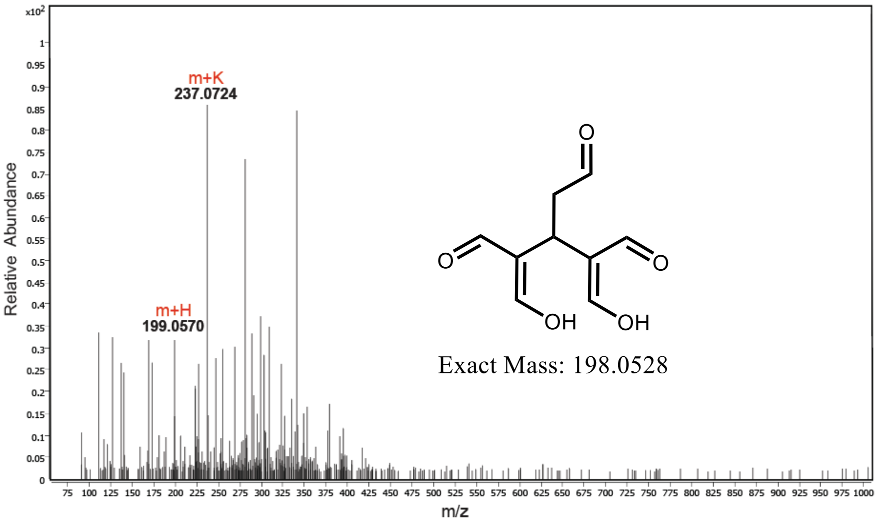

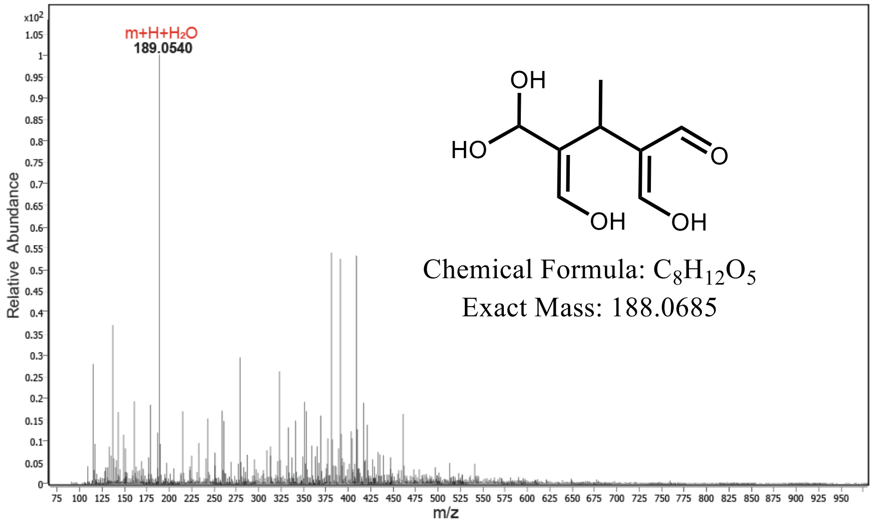

Peptide 1f. (pre-formed intermediate)

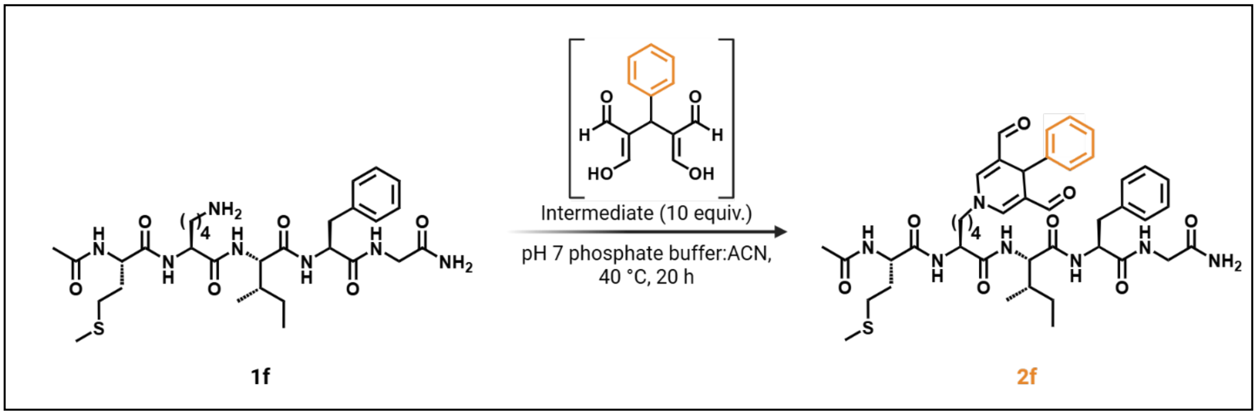

In a one-dram vial, peptide **1f** (Ac-MKIFG-CONH$_2$) (1.0 mg, 0.002 mmol) was dissolved in 300 µL of sodium phosphate buffer (100 mM, pH 7) and 100 µL of acetonitrile followed by the addition of pre-formed benzaldehyde intermediate (10 equiv.) and left to stir at 37 °C for 20 hours. Subsequently, the reaction mixture was injected into the HPLC for determining the % conversion of peptide **1f** to the

labeled peptide **2f** and its mass confirmed with LC-MS. HPLC analysis was carried out utilizing **HPLC Method A** at detection wavelength 220 nm. The mass of the product was confirmed with LC-MS and compiled below. The conversion was determined to be >95% of **2f.**

Ac-MKIFG-CONH$_2$ ($C_{30}H_{49}N_7O_6S$) peptide 1f. LCMS: *m/z* 636.3527 (calcd $[M+H]^+$ = 636.3538) (HPLC analysis at 220 nm). Retention time in HPLC: 11.2 min

Reaction Mixture:

Ac-MKIFG-CONH$_2$ ($C_{43}H_{57}N_7O_8S$) labeled product 2f. LCMS: *m/z* 832.4064 (calcd $[M+H]^+$ = 832.4062), *m/z* 854.3875 (calcd $[M+Na]^+$ = 854.3882) (HPLC analysis at 220 nm). Retention time in HPLC: 15.3 min. (>95%)

HPLC of Ac-MKIFG-CONH$_2$ Starting Peptide at 220 nm

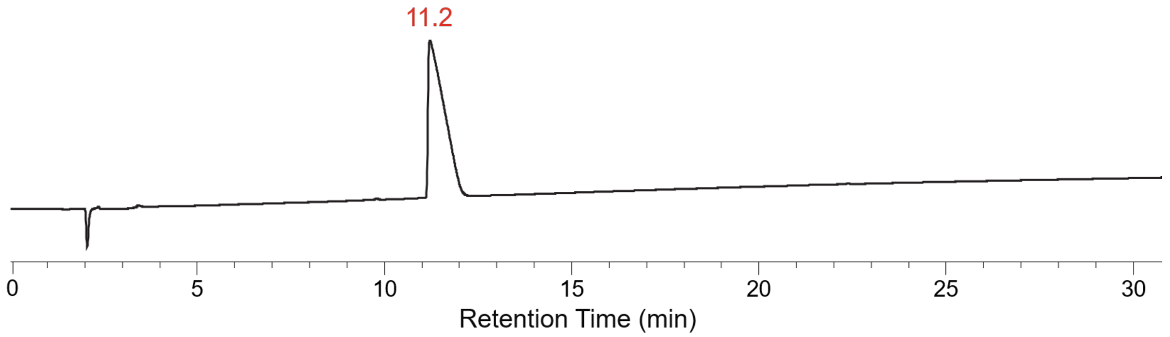

HRMS Trace of Peak at 11.2 min

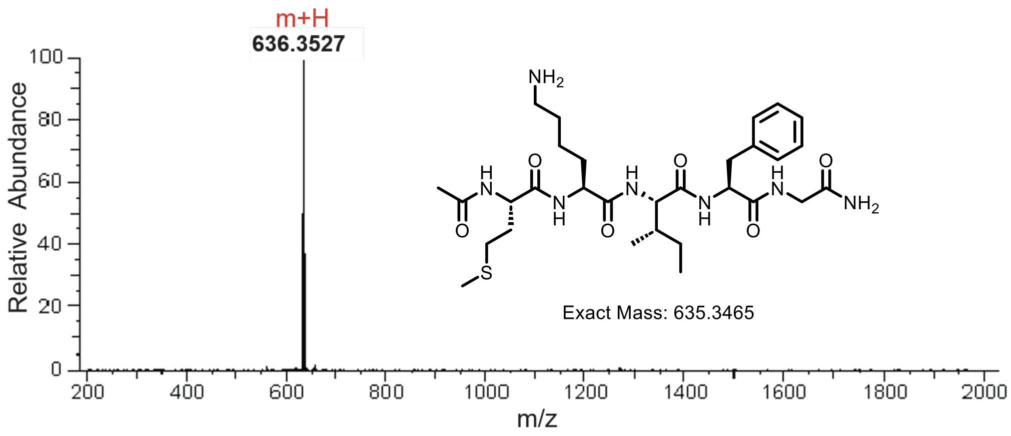

HPLC of Ac-MKIFG-CONH$_2$ Reaction Mixture at 220 nm

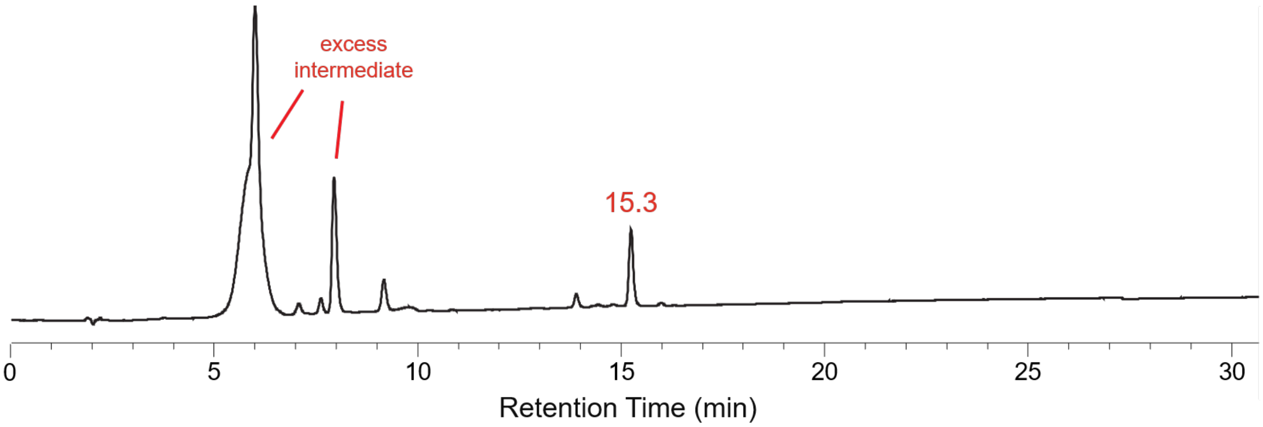

HRMS Trace of Peak at 15.3 min

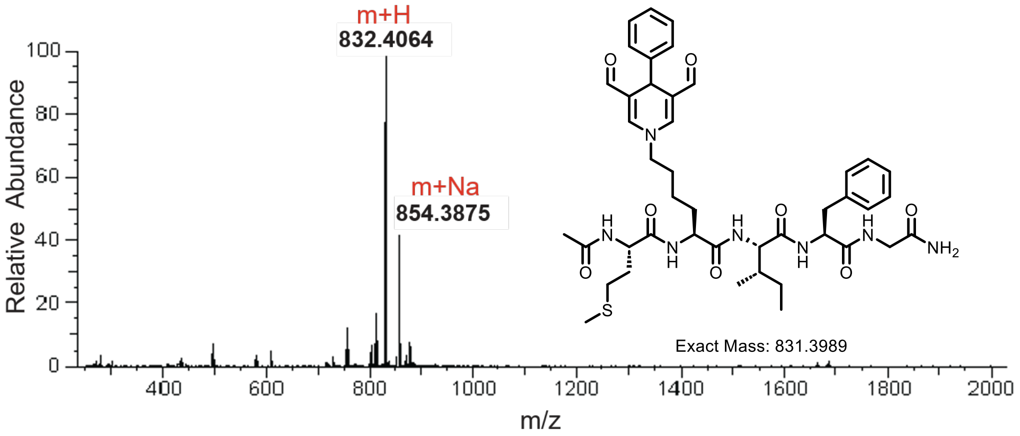

Peptide 1g.

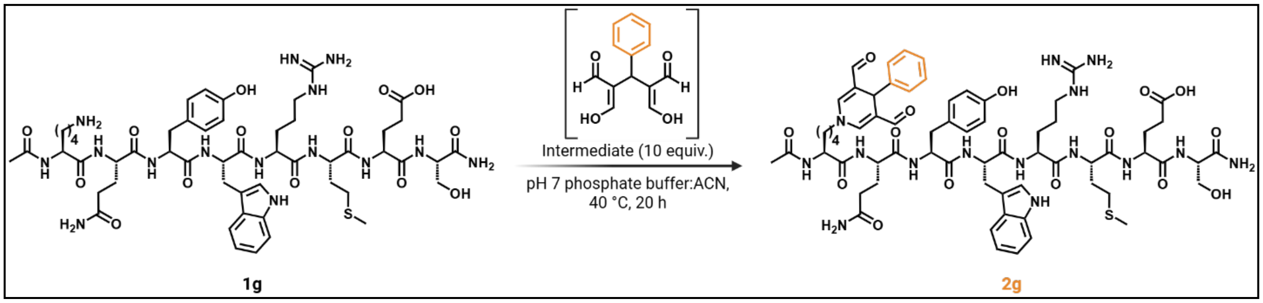

In a one-dram vial, peptide **1g** (Ac-KQYWRMES-CONH$_2$) (1.0 mg, 0.0009 mmol) was dissolved in 300 µL of sodium phosphate buffer (100 mM, pH 7) and 100 µL of acetonitrile followed by the addition of pre-formed benzaldehyde intermediate (10 equiv.) and left to stir at 37 °C for 20 hours. Subsequently, the reaction mixture was injected into the HPLC for determining the % conversion of peptide **1g** to the labeled peptide **2g** and its mass confirmed with LC-MS. HPLC analysis was carried out utilizing **HPLC Method A** at detection wavelength 220 nm. The mass of the product was confirmed with LC-MS and compiled below. The conversion was determined to be >95% of **2g.**

Ac-KQYWRMES-CONH$_2$ ($C_{52}H_{77}N_{15}O_{14}S$) peptide 1g. LCMS: *m/z* 1168.5573 (calcd $[M+H]^+$ = 1168.5568), *m/z* 584.7820 (calcd $[M+2H/2]^+$ = 584.7821) (HPLC analysis at 220 nm). Retention time in HPLC: 9.6 min

Reaction Mixture:

Ac-KQYWRMES-CONH$_2$ ($C_{65}H_{85}N_{15}O_{16}S$) labeled product 2g. LCMS: *m/z* 1364.6067 (calcd $[M+H]^+$ = 1364.6092) (HPLC analysis at 220 nm). Retention time in HPLC: 13.6 min (>95%)

HPLC of Ac-KQYWRMES-CONH$_2$ Starting Peptide at 220 nm

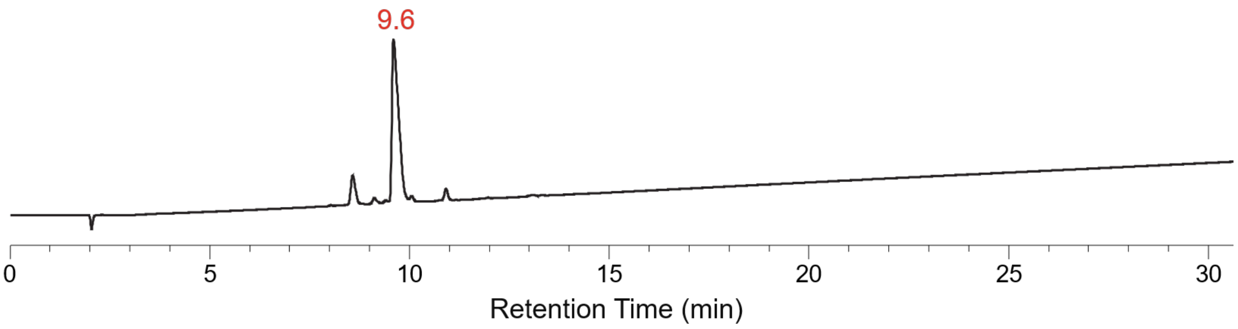

HRMS Trace of Peak at 9.6 min

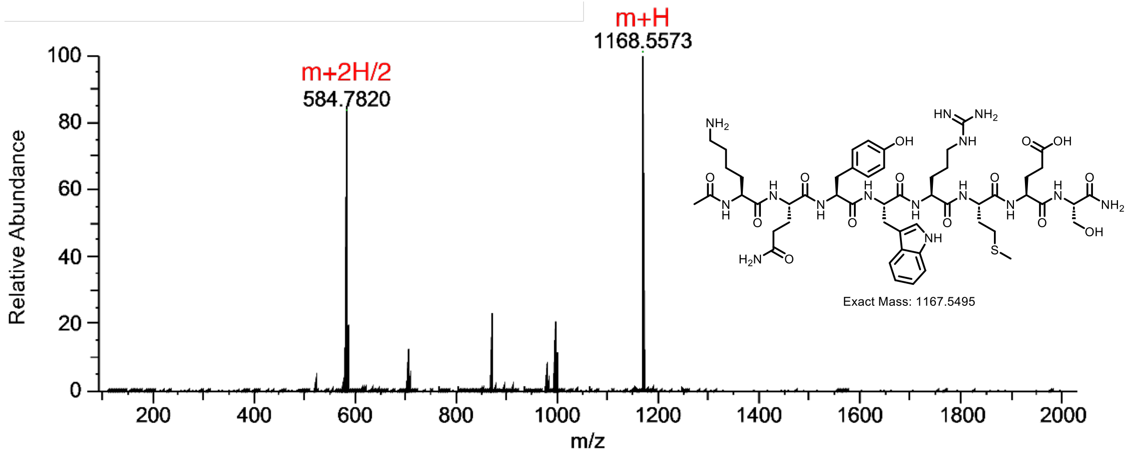

HPLC of Ac-KQYWRMES-CONH$_2$ Reaction Mixture at 220 nm

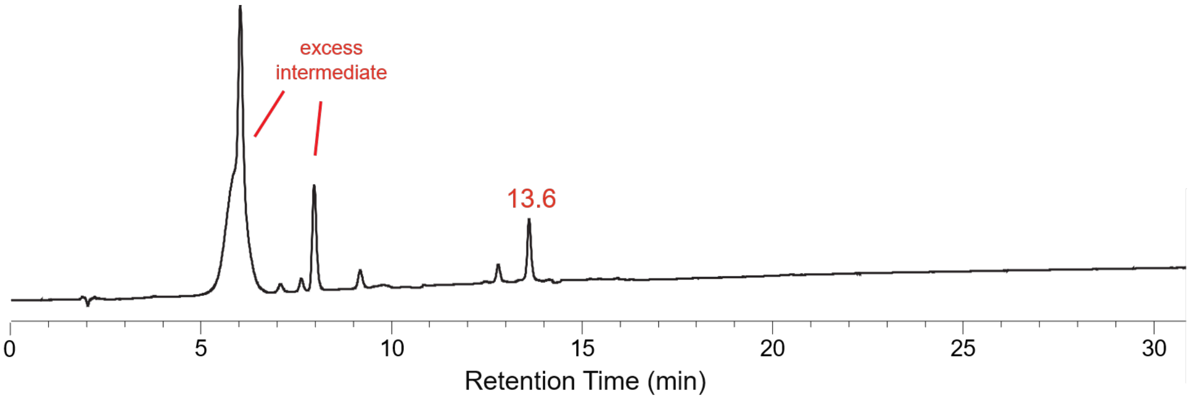

HRMS Trace of Peak at 13.6 min

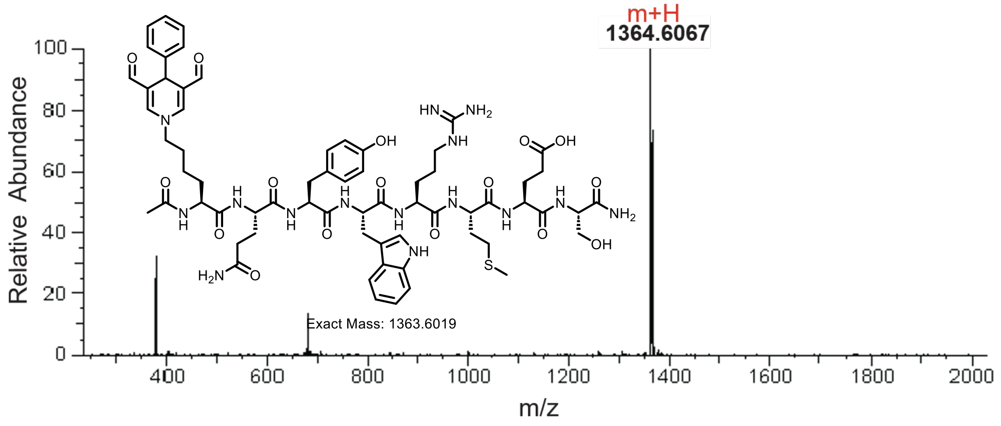

Peptide 1b.

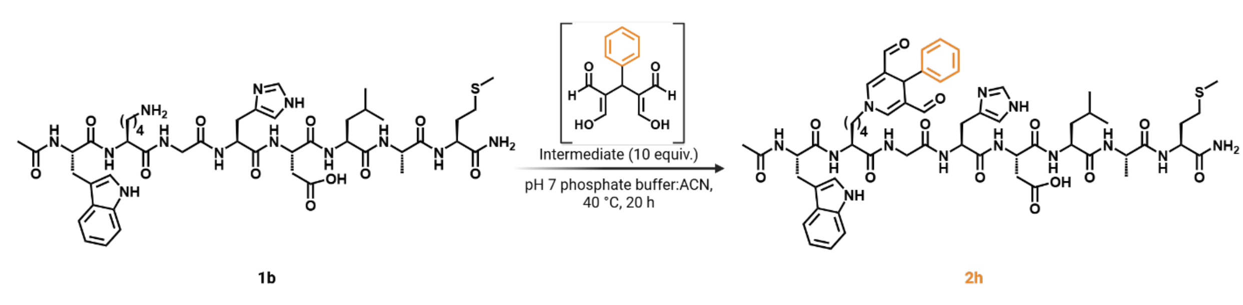

In a one-dram vial, peptide **1b** (Ac-WKGHDLAM-$CONH_2$) (1.0 mg, 0.001 mmol) was dissolved in 300 μL of sodium phosphate buffer (100 mM, pH 7) and 100 μL of acetonitrile followed by the addition of pre-formed benzaldehyde intermediate (10 equiv.) and left to stir at 37 °C for 20 hours. Subsequently, the reaction mixture was injected into the HPLC for determining the % conversion of peptide **1b** to the labeled peptide **2h** and its mass confirmed with LC-MS. HPLC analysis was carried out utilizing **HPLC Method A** at detection wavelength 220 nm. The mass of the product was confirmed with LC-MS. The conversion was determined to be >95% of **2h**.

Ac-WKGHDLAM-$CONH_2$ ($C_{45}H_{67}N_{13}O_{11}S$) peptide 1b. LCMS: *m/z* 998.4881 (calcd $[M+H]^+$ = 998.4876) *m/z* 499.7474 (calcd $[M+2H/2]^+$ = 499.7475) (HPLC analysis at 220 nm). Retention time in HPLC: 7.8 min

Reaction Mixture:

Ac-WKGHDLAM-$CONH_2$ ($C_{58}H_{75}N_{13}O_{13}S$) labeled product 2h. LCMS: 1194.5386 (calcd $[M+H]^+$ = 1194.5401) (HPLC analysis at 220 nm). Retention time in HPLC: 13.9 min. (>95%)

HPLC of Ac-WKGHDLAM-CONH$_2$ Starting Peptide at 220 nm

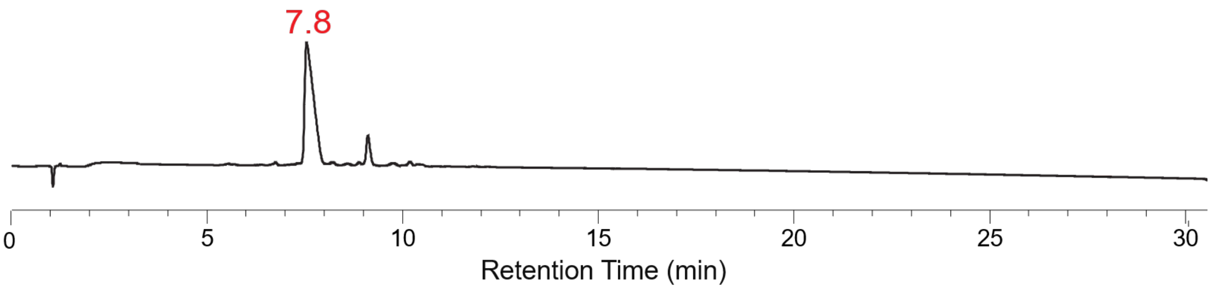

HRMS Trace of Peak at 7.8 min

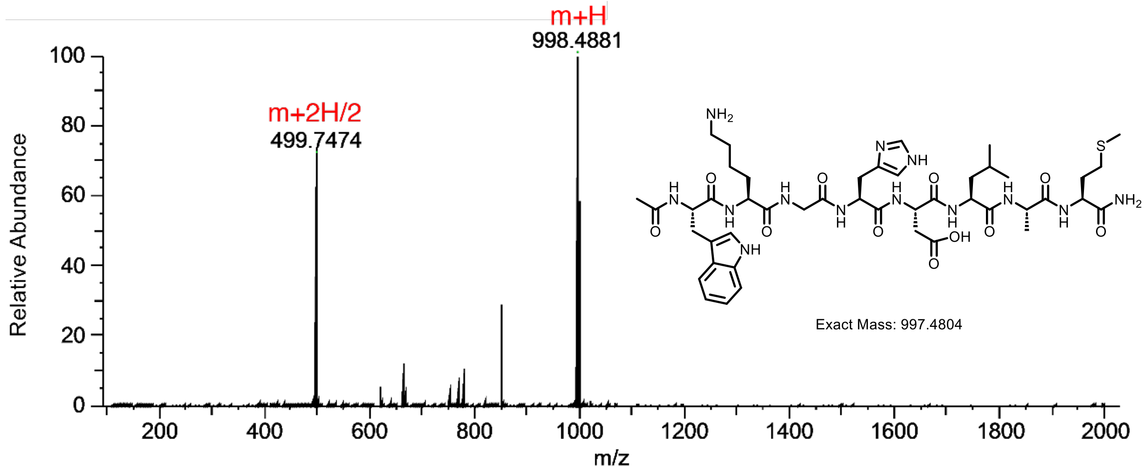

HPLC Trace of Ac-WKGHDLAM-CONH$_2$ Reaction Mixture at 220 nm

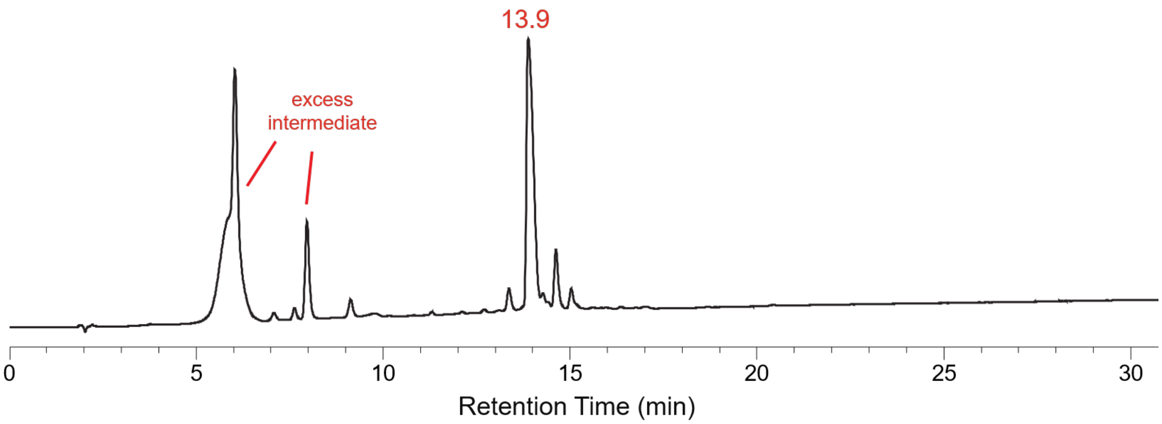

HRMS Trace of Peak at 13.9 min

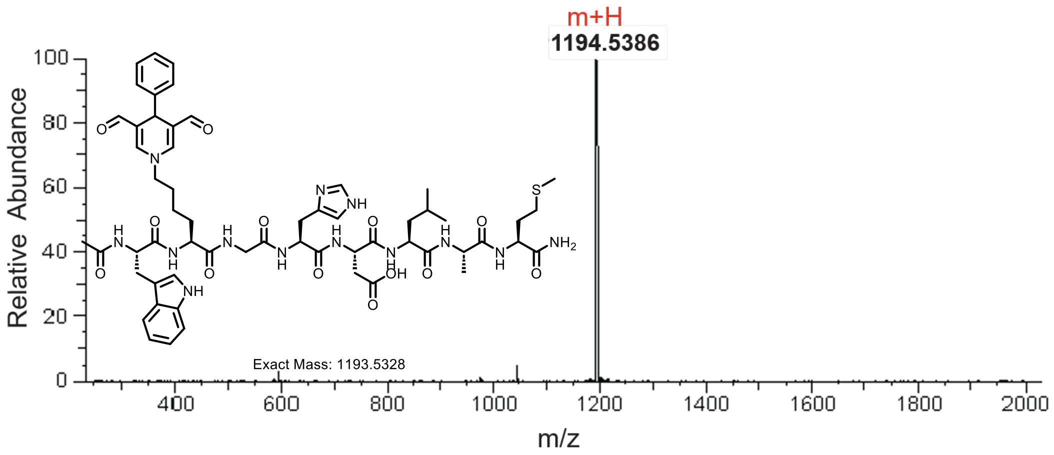

Supplementary Figure 5. Chemoselectivity Studies of MDA-monoaldehyde Cooperative Complexes.

Peptide 1i. MDA-Benzaldehyde Complex

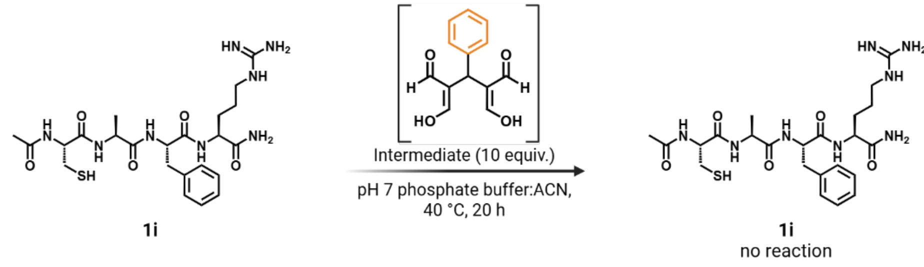

In a one-dram vial, peptide **1i** (Ac-CAFR-CONH$_2$) (1.0 mg, 0.002 mmol) was dissolved in 300 μL of sodium phosphate buffer (100 mM, pH 7) and 100 μL of acetonitrile followed by the addition of pre-formed benzaldehyde intermediate (10 equiv.) and left to stir at 37 °C for 20 hours. Subsequently, the reaction mixture was injected into the HPLC for determining the % conversion of peptide **1i** and its mass confirmed with LC-MS. HPLC analysis was carried out utilizing **HPLC Method A** at detection wavelength 220 nm. The mass of the product was confirmed with LC-MS and compiled below.

Ac-CAFR-CONH$_2$ ($C_{23}H_{35}N_7O_6S$) peptide 1i. LCMS: *m/z* 537.2595 (calcd $[M+H]^+$ = 537.2602) (HPLC analysis at 220 nm). Retention time in HPLC: 7.5 min

Ac-CAFR-CONH$_2$ ($C_{46}H_{70}N_{16}O_{10}S_2$) peptide 1i (disulfide). LCMS: *m/z* 1071.4971 (calcd $[M+H]^+$ = 1071.4975), *m/z* 536.2528 (calcd $[M+2H/2]^+$ = 536.2524) (HPLC analysis at 220 nm). Retention time in HPLC: 10.1 min

Reaction Mixture:

Ac-CAFR-CONH$_2$ ($C_{46}H_{70}N_{16}O_{10}S_2$) peptide 1i (disulfide). LCMS: *m/z* 1071.4971 (calcd $[M+H]^+$ = 1071.4975), *m/z* 536.2526 (calcd $[M+2H/2]^+$ = 536.2524) (HPLC analysis at 220 nm). Retention time in HPLC: 10.0 min

HPLC of Ac-CAFR-CONH$_2$ Starting Peptide at 220 nm

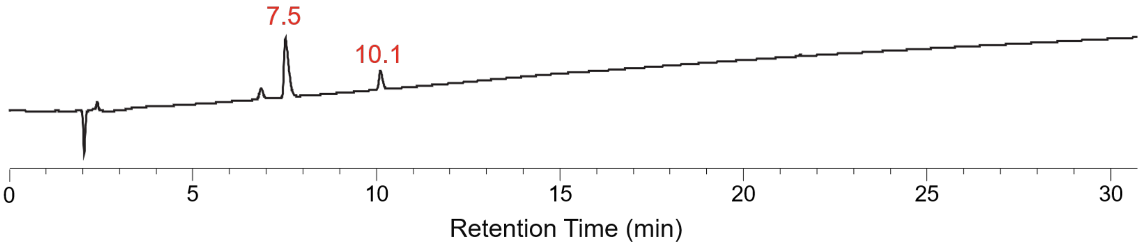

HRMS Trace of Peak at 7.5 min

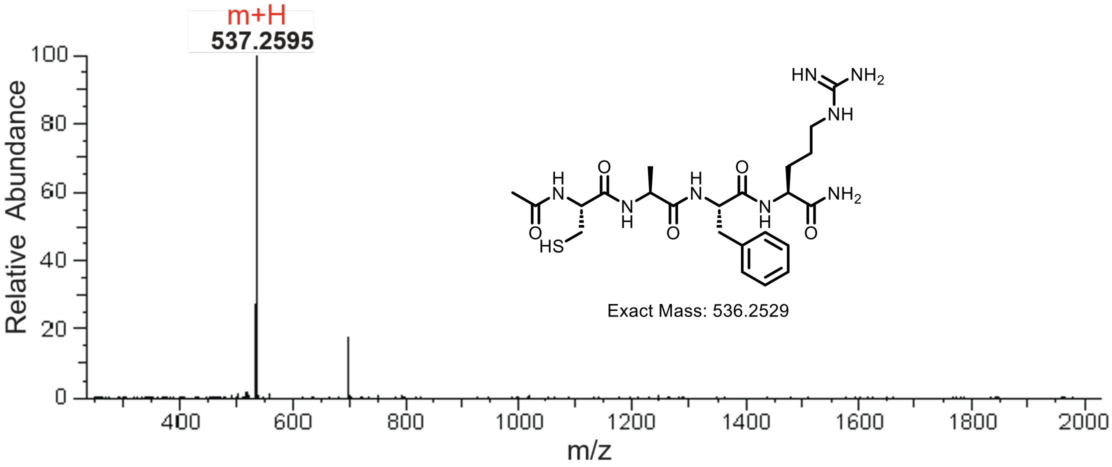

HRMS Trace of Peak at 10.1 min

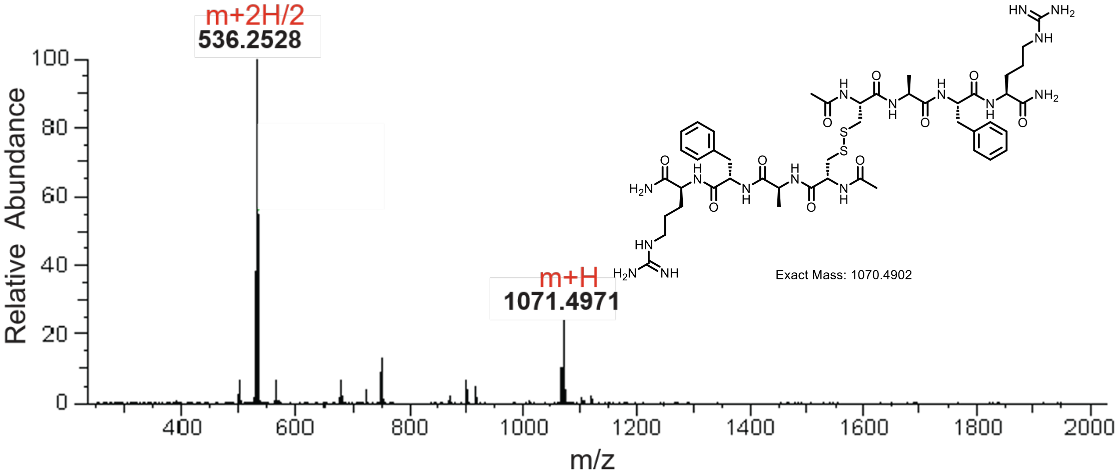

HPLC of Ac-CAFR-CONH$_2$ Reaction Mixture at 220 nm

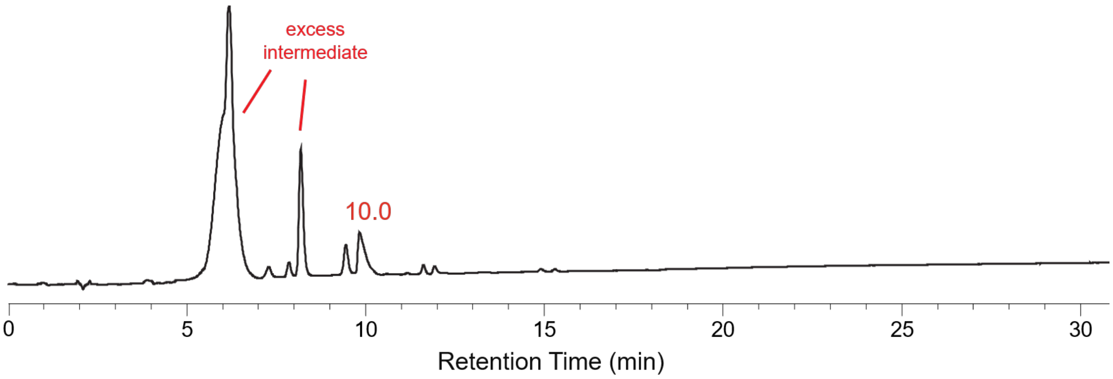

HRMS Trace of Peak at 10.0 min

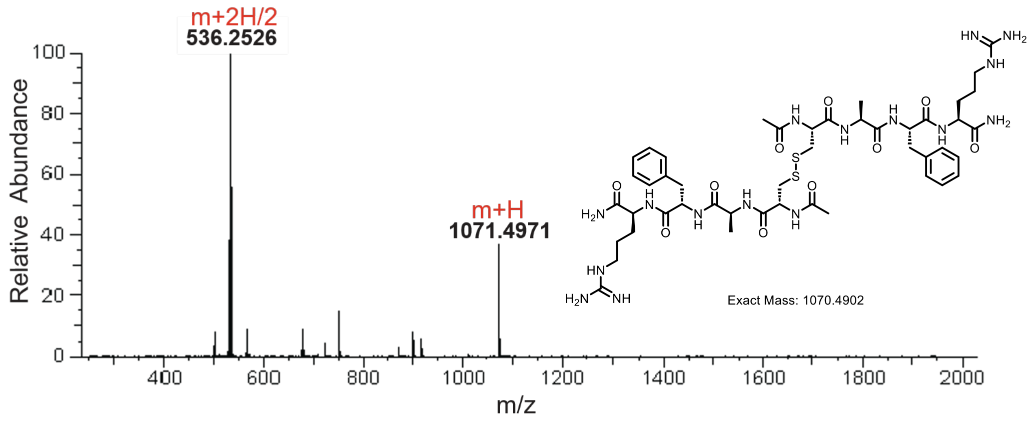

Peptide 1j. MDA-Benzaldehyde Complex

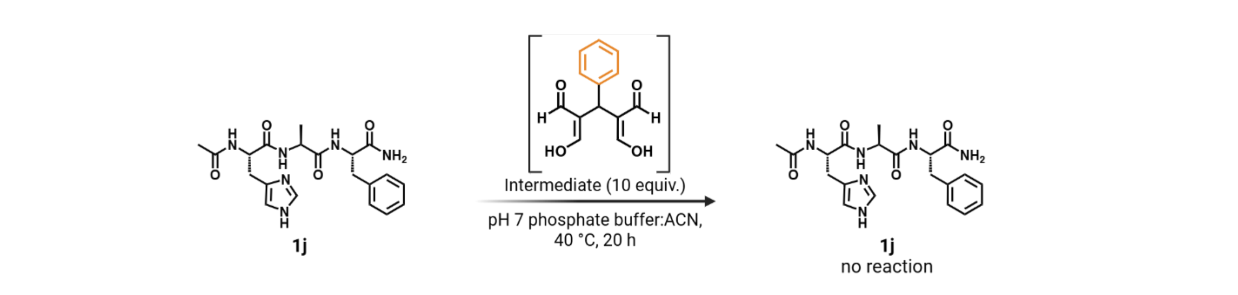

In a one-dram vial, peptide **1j** (Ac-HAF-CONH$_2$) (1.0 mg, 0.002 mmol) was dissolved in 300 µL of sodium phosphate buffer (100 mM, pH 7) and 100 µL of acetonitrile followed by the addition of pre-formed benzaldehyde intermediate (10 equiv.) and left to stir at 37 °C for 20 hours. Subsequently, the reaction mixture was injected into the HPLC for determining the % conversion of peptide **1j** and its mass confirmed with LC-MS. HPLC analysis was carried out utilizing **HPLC Method A** at detection wavelength 220 nm. The mass of the product was confirmed with LC-MS and compiled below.

Ac-HAF-CONH$_2$ ($C_{20}H_{26}N_6O_4$) peptide 1j. LCMS: *m/z* 415.2085 (calcd $[M+H]^+$ = 415.2088) (HPLC analysis at 220 nm). Retention time in HPLC: 5.4 min

Reaction Mixture:

Ac-HAF-CONH$_2$ ($C_{20}H_{26}N_6O_4$) peptide 1j. LCMS: *m/z* 415.2094 (calcd $[M+H]^+$ = 415.2088), *m/z* 437.1910 (calcd [M+Na] = 437.1908) (HPLC analysis at 220 nm). Retention time in HPLC: 5.3 min

HPLC of Ac-HAF-CONH$_2$ Starting Peptide at 220 nm

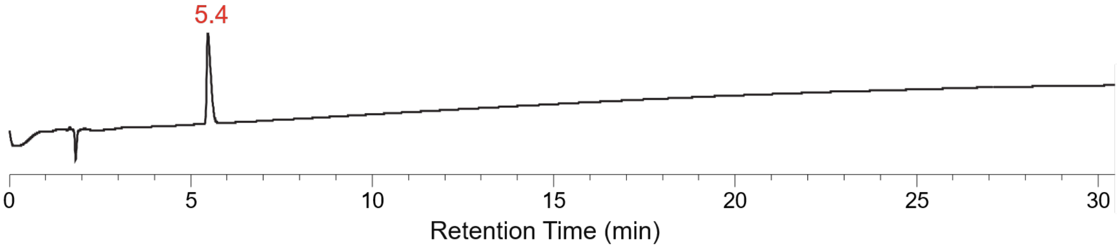

HRMS Trace of Peak at 5.4 min

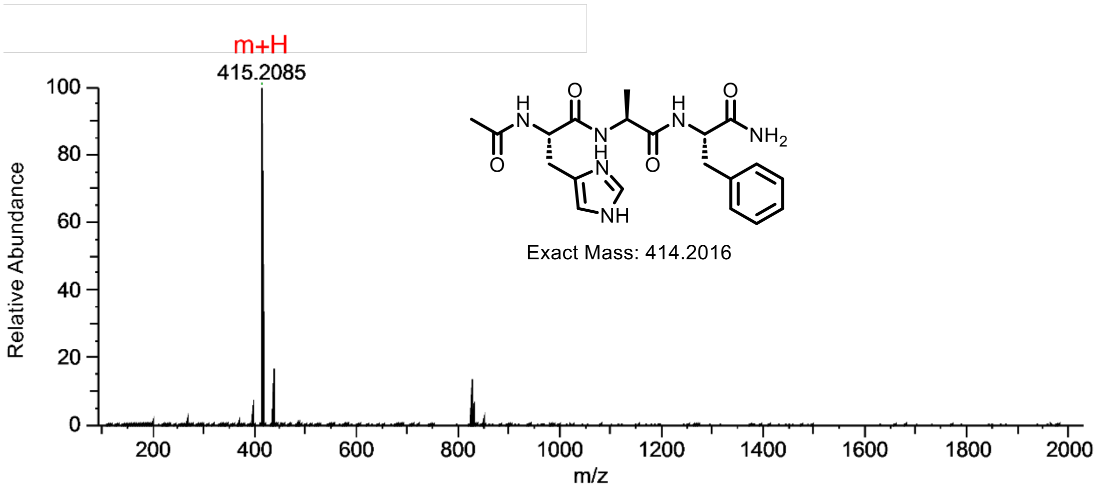

HPLC of Ac-HAF-CONH$_2$ Reaction Mixture at 220 nm

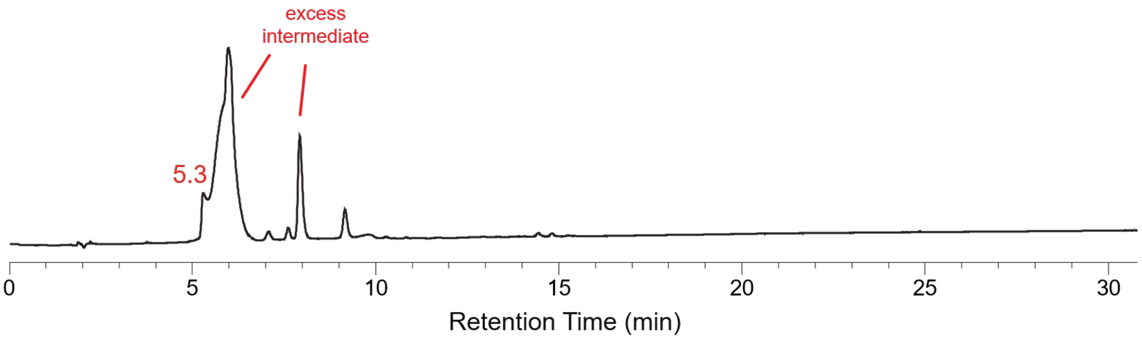

HRMS Trace of Peak at 5.3 min

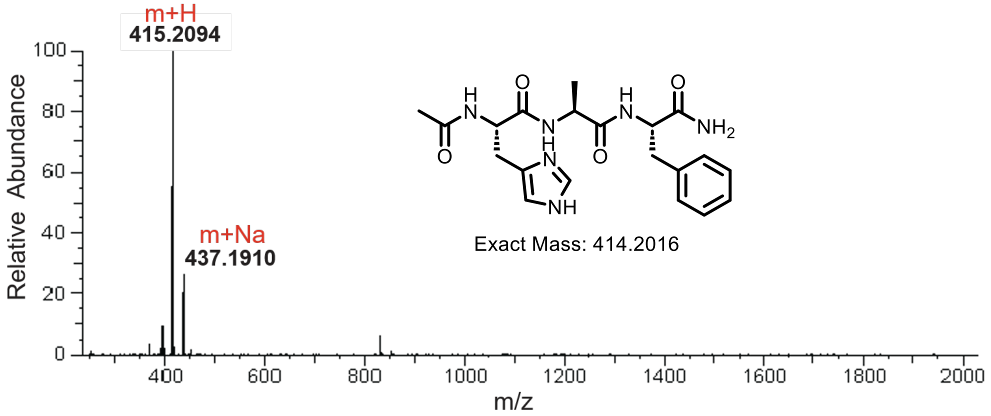

Supplementary Figure 6. Substrate Scope of MDA-MDA Cooperative Complexes.

Peptide 1f. (optimized conditions)

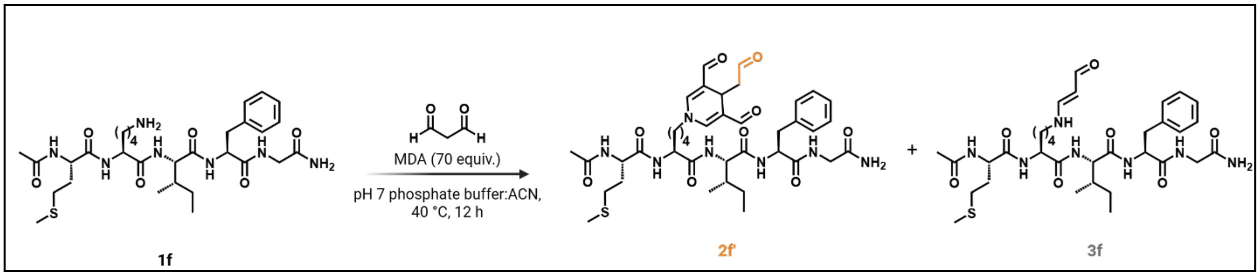

In a one-dram vial, peptide **1f** (Ac-MKIFG-CONH$_2$) (1.0 mg, 0.002 mmol) was dissolved in 300 µL of sodium phosphate buffer (100 mM, pH 7) and 100 µL of acetonitrile followed by the addition of MDA sodium salt (70 equiv.) and left to stir at 37 °C for 12 hours. Subsequently, the reaction mixture was injected into the HPLC for determining the % conversion of peptide **1f** to the labeled peptides **2f'** or **3f** and their mass confirmed with LC-MS. HPLC analysis was carried out utilizing **HPLC Method A** at detection wavelength 220 nm. The masses of the products were confirmed with LC-MS and compiled below. The conversion was determined to be 15% of **1f** 42% of **2f'**, and 43% **3f**.

Ac-MKIFG-CONH$_2$ ($C_{30}H_{49}N_7O_6S$) peptide 1f. LCMS: *m/z* 636.3527 (calcd $[M+H]^+$ = 636.3538) (HPLC analysis at 220 nm). Retention time in HPLC: 11.2 min

Reaction Mixture:

Ac-MKIFG-CONH$_2$ ($C_{30}H_{49}N_7O_6S$) peptide 1f. LCMS: *m/z* 636.3533 (calcd $[M+H]^+$ = 636.3538) (HPLC analysis at 220 nm). Retention time in HPLC: 11.2 min. (15%)

Ac-MKIFG-CONH$_2$ ($C_{39}H_{55}N_7O_9S$) labeled product 2f'. LCMS: *m/z* 798.3849 (calcd $[M+H]^+$ = 798.3855) (HPLC analysis at 220 nm). Retention time in HPLC: 12.1 min. (42%)

Ac-MKIFG-CONH$_2$ ($C_{33}H_{51}N_7O_7S$) side product 3f. LCMS: *m/z* 690.3641 (calcd $[M+H]^+$ = 690.3643) (HPLC analysis at 220 nm). Retention time in HPLC: 13.0 min. (43%)

HPLC of Ac-MKIFG-CONH₂ Starting Peptide at 220 nm

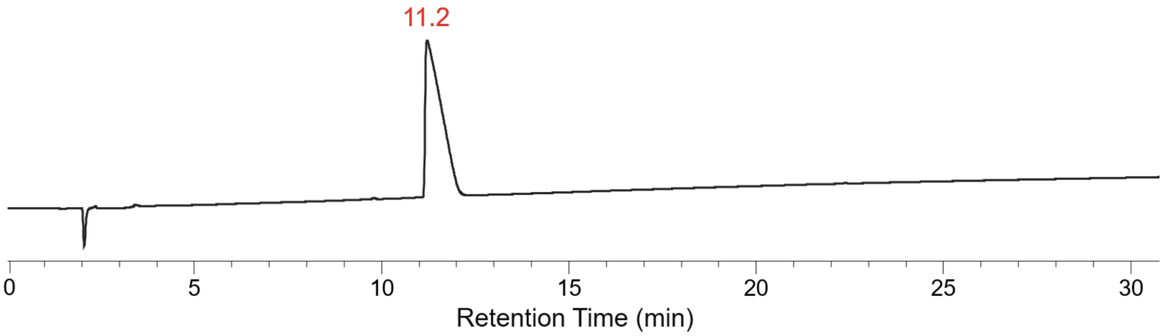

HRMS Trace of Peak at 11.2 min

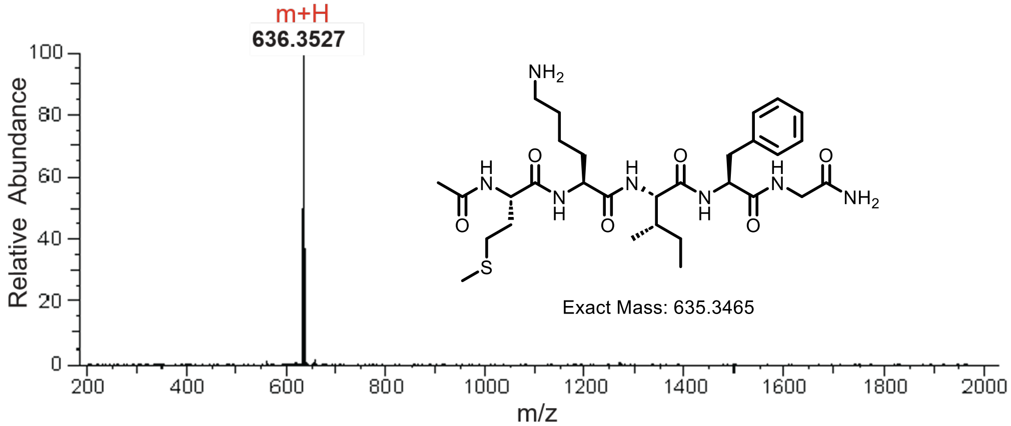

HPLC of Ac-MKIFG-CONH₂ Reaction Mixture at 220 nm

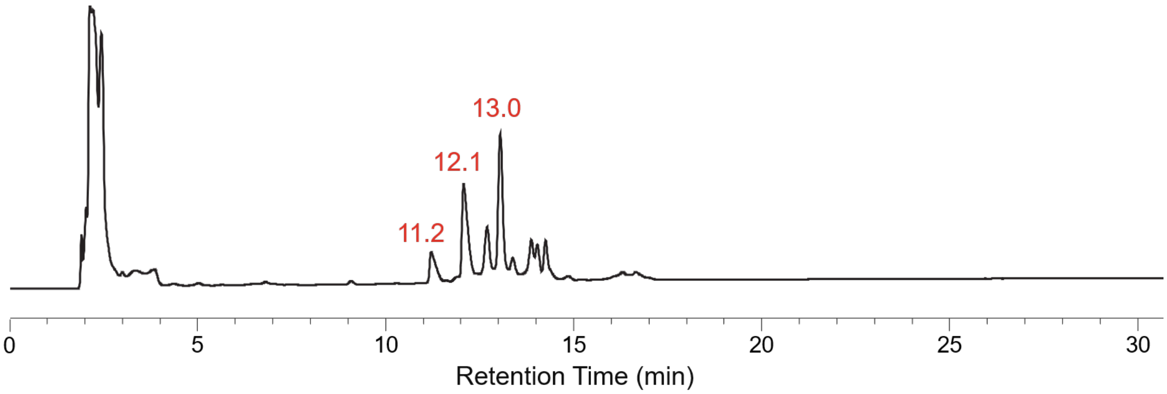

HRMS Trace of Peak at 11.2 min

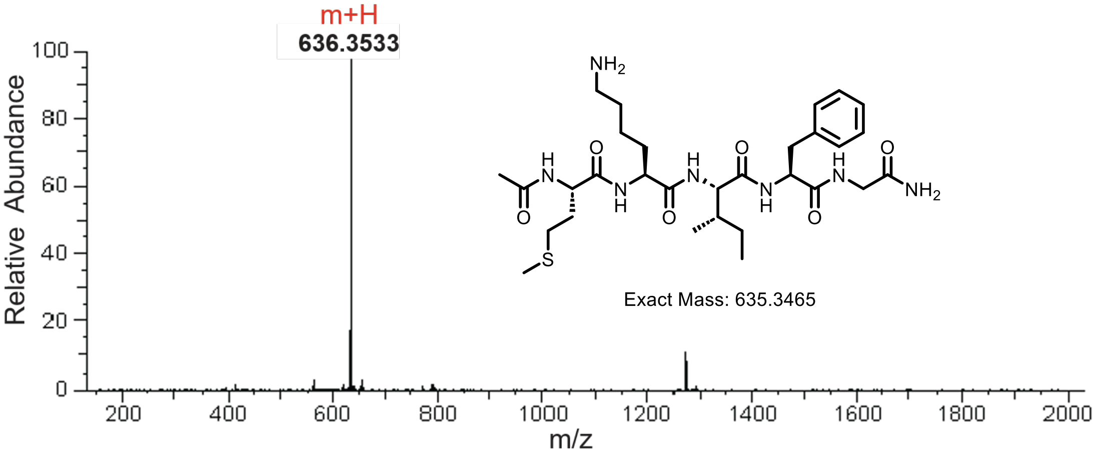

HRMS Trace of Peak at 12.1 min

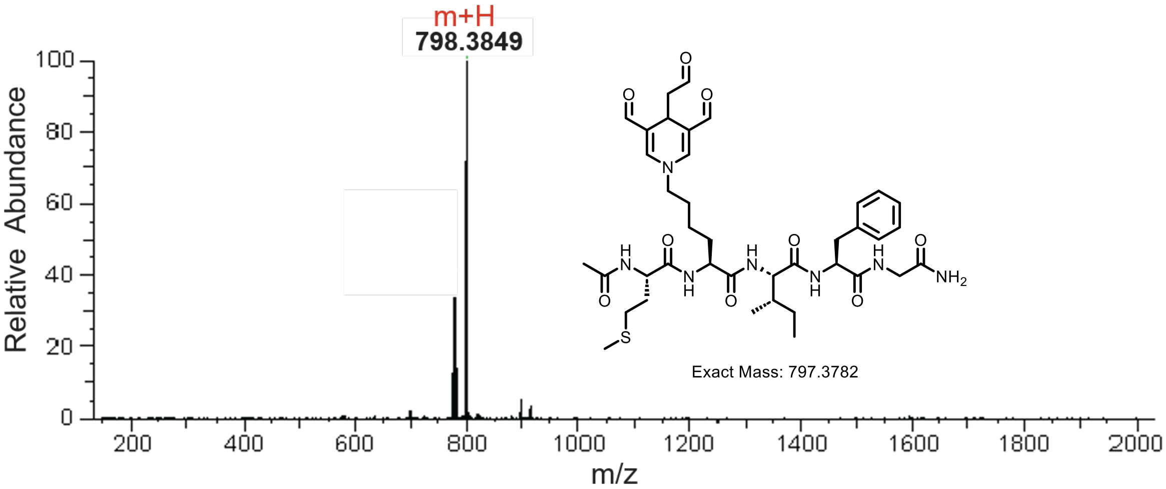

HRMS Trace of Peak at 13.0 min

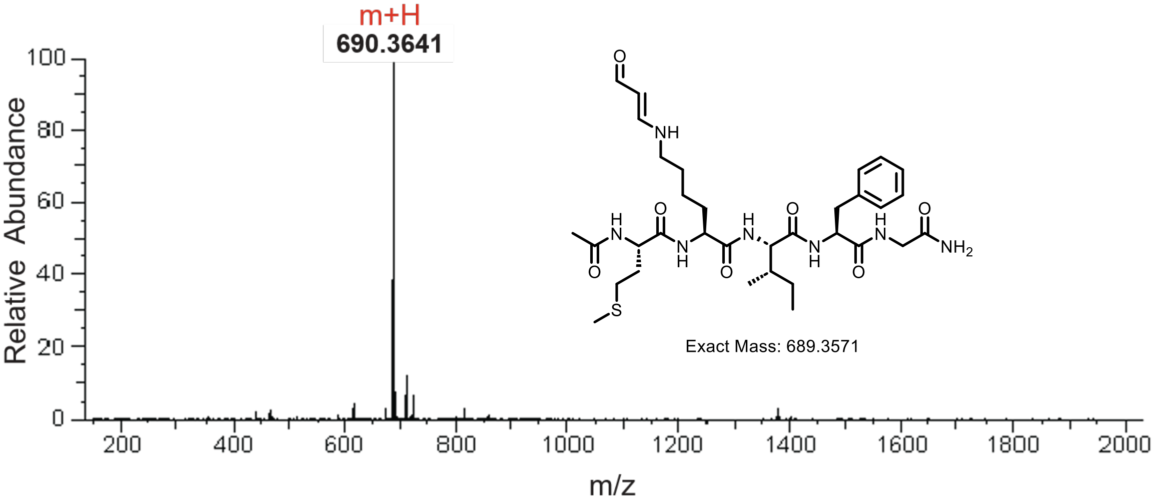

Peptide 1f. (pre-formed intermediate)

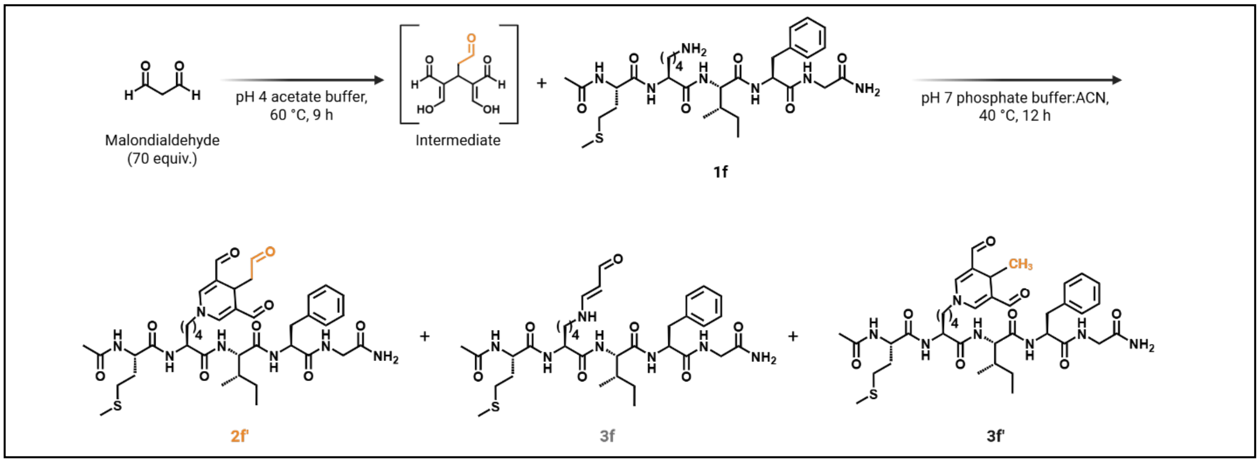

In a one-dram vial, MDA sodium salt (10.1 mg, 0.14 mmol) was dissolved in 100 µL of sodium acetate buffer (100 mM, pH 4) and left to stir at 60 °C for 9 hours. Then, peptide **1f** (Ac-MKIFG-CONH$_2$) (1.0 mg, 0.002 mmol) was dissolved in 300 µL of sodium phosphate buffer (100 mM, pH 7) and 100 µL of acetonitrile, added to the reaction mixture and left to stir at 37 °C for 12 hours. Subsequently, the reaction mixture was injected into the HPLC for determining the % conversion of peptide **1f** to the labeled peptides **2f'**, **3f**, or **3f'** and their mass confirmed with LC-MS. HPLC analysis was carried out utilizing **HPLC Method A** at detection wavelength 220 nm. The masses of the products were confirmed with LC-MS and compiled below. The conversion was determined to be 48% of **2f'**, 18% of **3f**, and 34% **3f'**.

Ac-MKIFG-CONH$_2$ ($C_{30}H_{49}N_7O_6S$) peptide 1f. LCMS: *m/z* 636.3527 (calcd $[M+H]^+$ = 636.3538) (HPLC analysis at 220 nm). Retention time in HPLC: 11.2 min

Reaction Mixture:

Ac-MKIFG-CONH$_2$ ($C_{39}H_{55}N_7O_9S$) labeled product 2f'. LCMS: *m/z* 798.3852 (calcd $[M+H]^+$ = 798.3855) (HPLC analysis at 220 nm). Retention time in HPLC: 12.1 min. (48%)

Ac-MKIFG-CONH$_2$ ($C_{33}H_{51}N_7O_7S$) side product 3f. LCMS: *m/z* 690.3643 (calcd $[M+H]^+$ = 690.3643), *m/z* 712.3457 (calcd $[M+Na]^+$ = 712.3463) (HPLC analysis at 220 nm). Retention time in HPLC: 13.1 min. (18%)

Ac-MKIFG-CONH$_2$ ($C_{38}H_{55}N_7O_8S$) labeled product 3f'. LCMS: *m/z* 770.3910 (calcd $[M+H]^+$ = 770.3906), *m/z* 792.3721 (calcd $[M+Na]^+$ = 792.3725) (HPLC analysis at 220 nm). Retention time in HPLC: 14.1 min. (34%)

HPLC of Ac-MKIFG-CONH$_2$ Starting Peptide at 220 nm

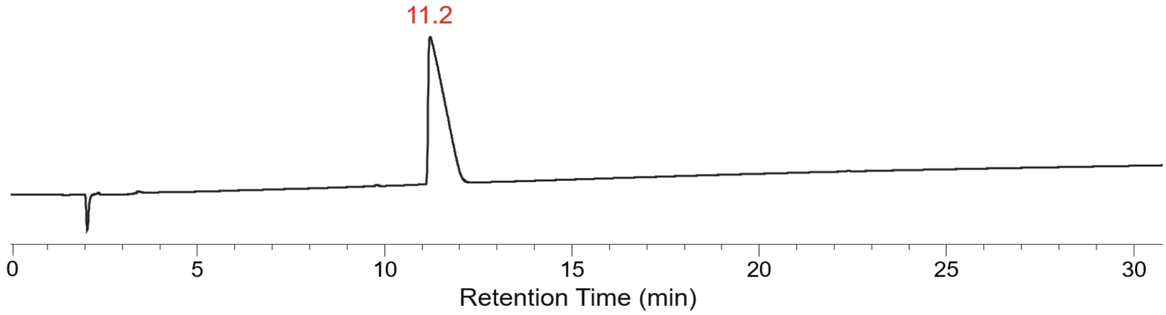

HRMS Trace of Peak at 11.2 min

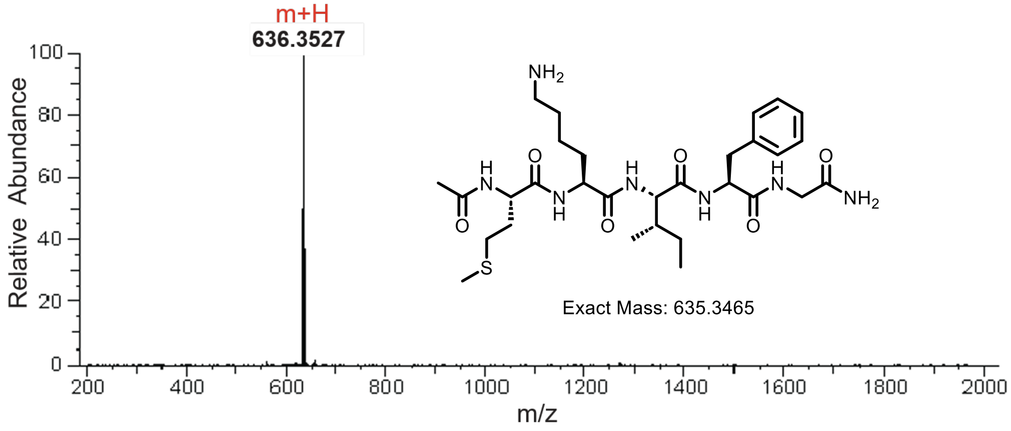

HPLC of Ac-MKIFG-CONH₂ Reaction Mixture at 220 nm

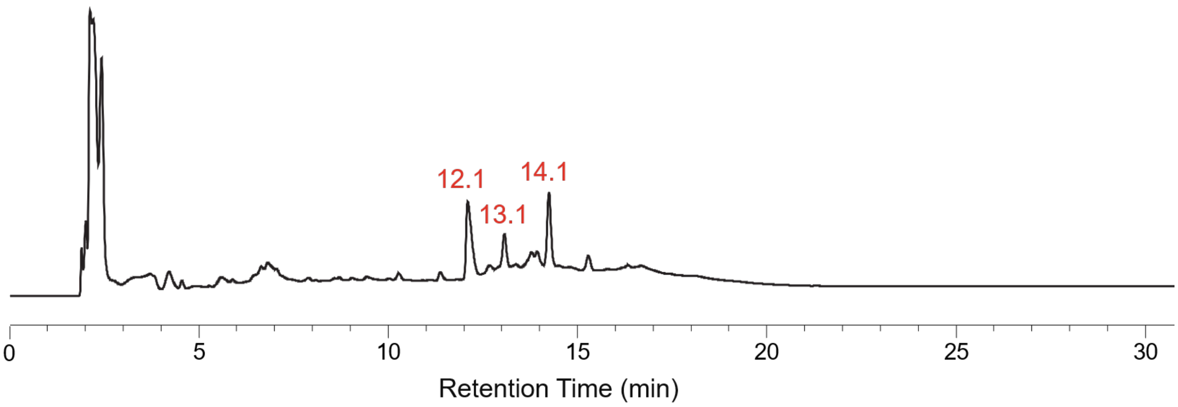

HRMS Trace of Peak at 12.1 min

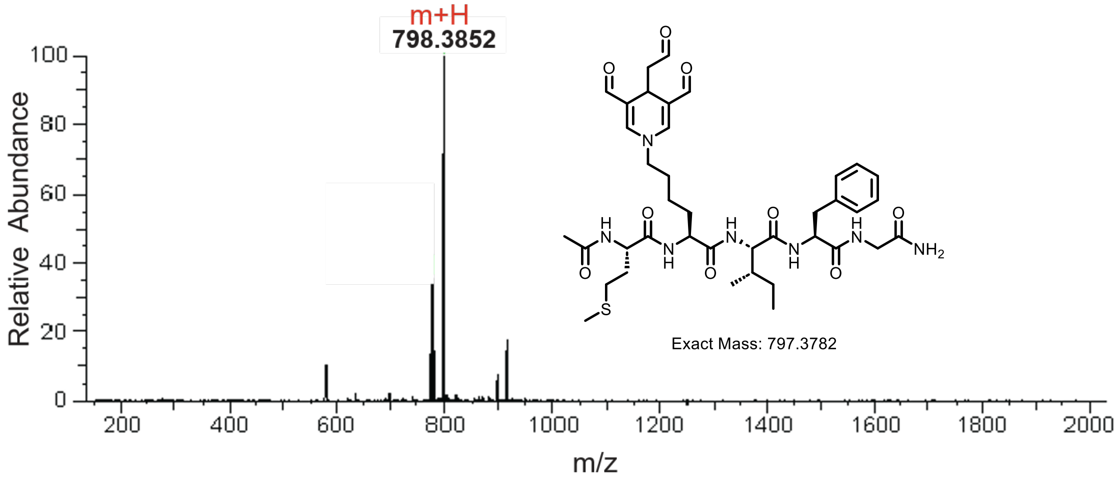

HRMS Trace of Peak at 13.1 min

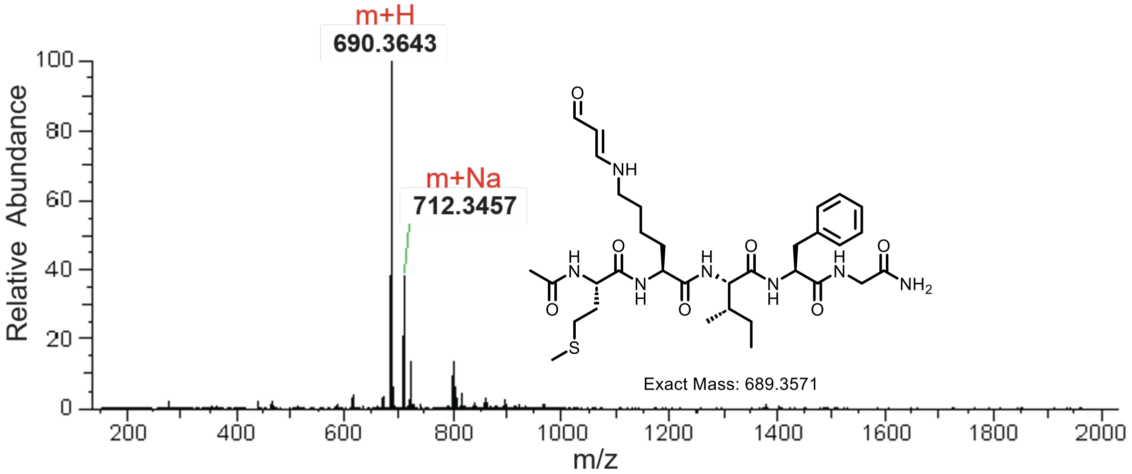

HRMS Trace of Peak at 14.1 min

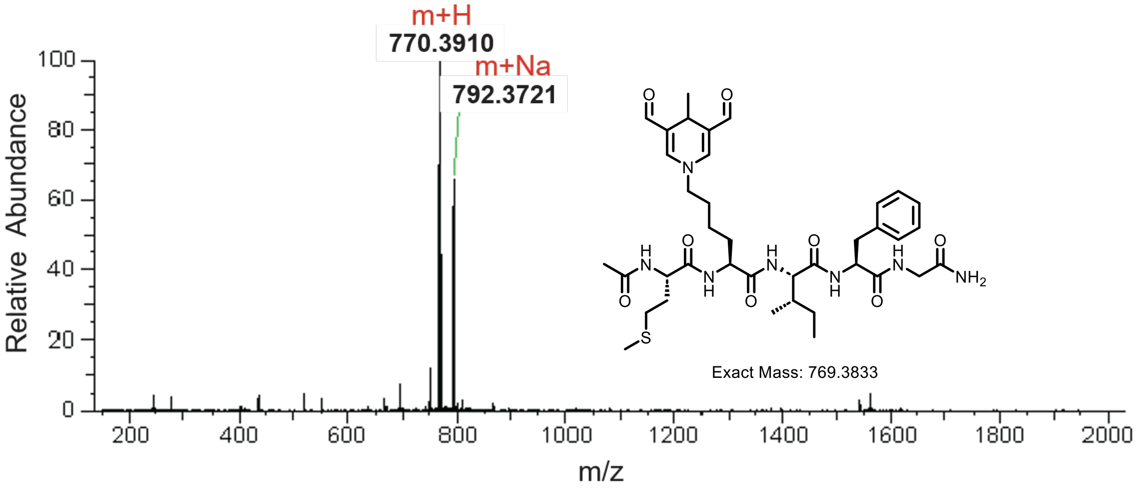

Peptide 1g.

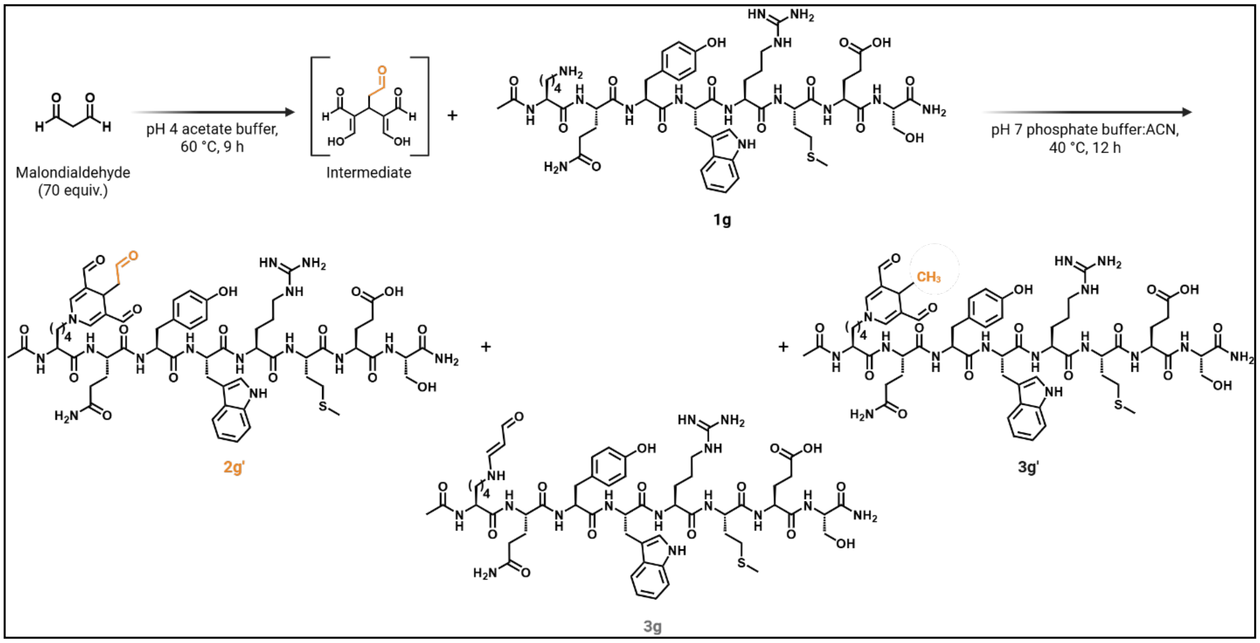

In a one-dram vial, MDA sodium salt (4.5 mg, 0.06 mmol) was dissolved in 100 µL of sodium acetate buffer (100 mM, pH 4) and left to stir at 60 °C for 9 hours. Then, peptide **1g** (Ac-KQYWRMES-$CONH_2$) (1.0 mg, 0.0009 mmol) was dissolved in 300 µL of sodium phosphate buffer (100 mM, pH 7) and 100 µL of acetonitrile, added to the reaction mixture and left to stir at 37 °C for 12 hours. Subsequently, the reaction mixture was injected into the HPLC for determining the % conversion of peptide **1g** to the labeled peptides **2g'**, **3g**, or **3g'** and their mass confirmed with LC-MS. HPLC analysis was carried out utilizing **HPLC Method A** at detection wavelength 220 nm. The masses of the products were confirmed with LC-MS and compiled below. The conversion was determined to be 34% of **2g'**, 38% of **3g**, and 28% **3g'**.

Ac-KQYWRMES-$CONH_2$ ($C_{52}H_{77}N_{15}O_{14}S$) peptide 1g. LCMS: *m/z* 1168.5573 (calcd $[M+H]^+$ = 1168.5568), *m/z* 584.7820 (calcd $[M+2H/2]^+$ = 584.7821) (HPLC analysis at 220 nm). Retention time in HPLC: 9.6 min

Reaction Mixture:

Ac-KQYWRMES-$CONH_2$ ($C_{61}H_{83}N_{15}O_{17}S$) labeled product 2g'. LCMS: *m/z* 1330.5886 (calcd $[M+H]^+$ = 1330.5885), *m/z* 665.7982 (calcd $[M+2H/2]^+$ = 665.7979) (HPLC analysis at 220 nm). Retention time in HPLC: 10.5 min. (34%)

Ac-KQYWRMES-$CONH_2$ ($C_{55}H_{79}N_{15}O_{15}S$) side product 3g. LCMS: *m/z* 1222.5669 (calcd $[M+H]^+$ = 1222.5674) *m/z* 611.7876 (calcd $[M+2H/2]^+$ = 611.7873) (HPLC analysis at 220 nm). Retention time in HPLC: 11.6 min. (38%)

Ac-KQYWRMES-$CONH_2$ ($C_{60}H_{83}N_{15}O_{16}S$) labeled product 3g'. LCMS: *m/z* 1302.5931 (calcd $[M+H]^+$ = 1302.5936) (HPLC analysis at 220 nm). Retention time in HPLC: 12.5 min. (28%)

HPLC of Ac-KQYWRMES-CONH$_2$ Starting Peptide at 220 nm

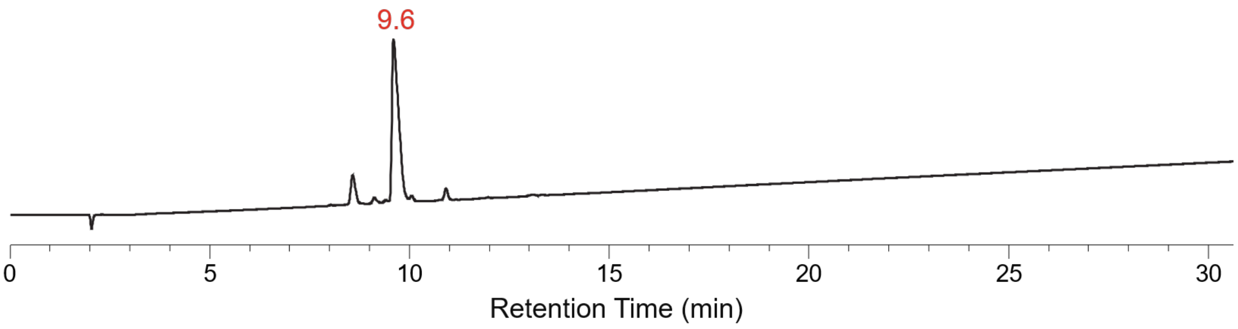

HRMS Trace of Peak at 9.6 min

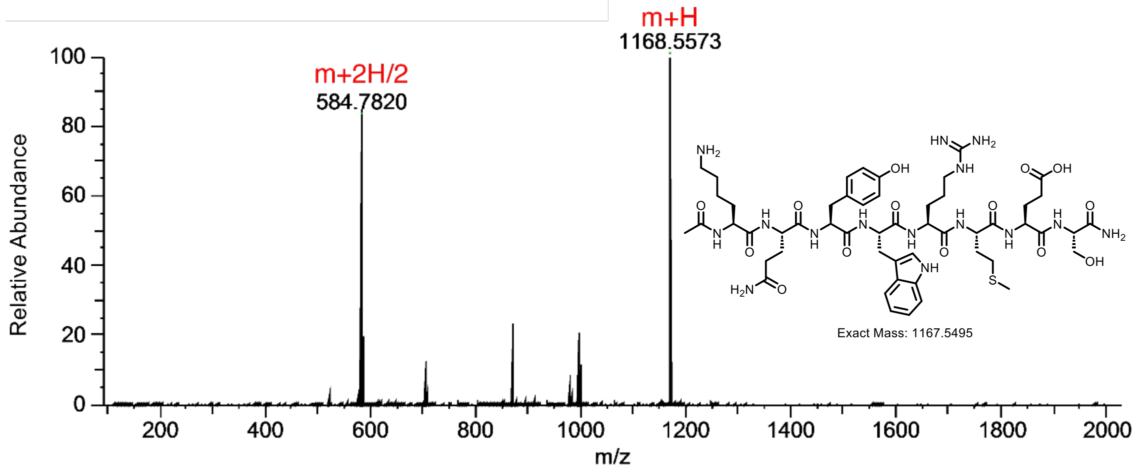

HPLC of Ac-KQYWRMES-CONH$_2$ Reaction Mixture at 220 nm

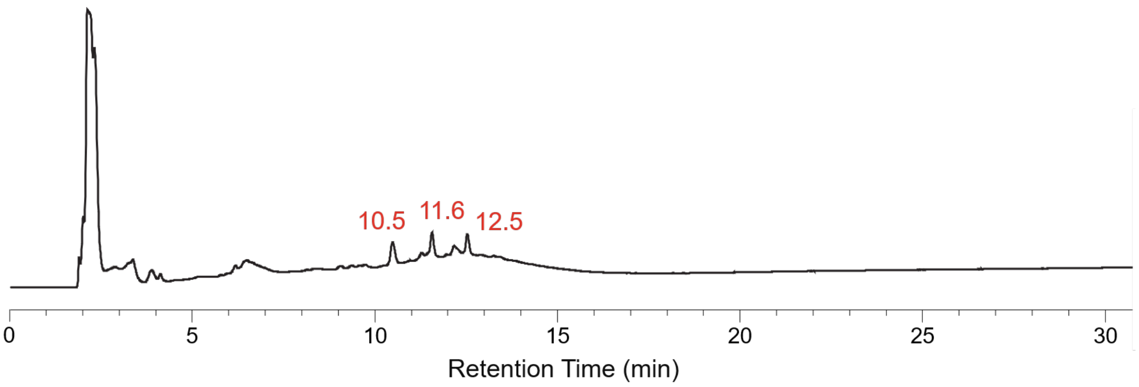

HRMS Trace of Peak at 10.5 min

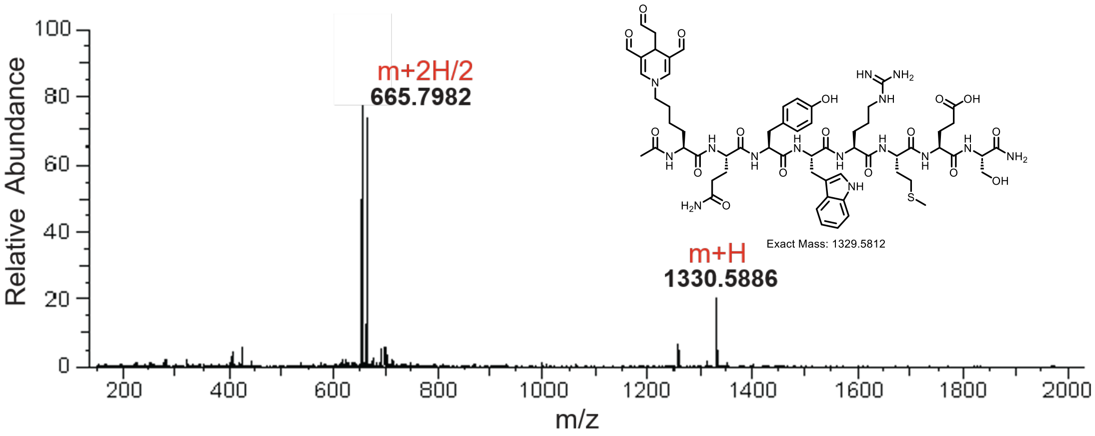

HRMS Trace of Peak at 11.6 min

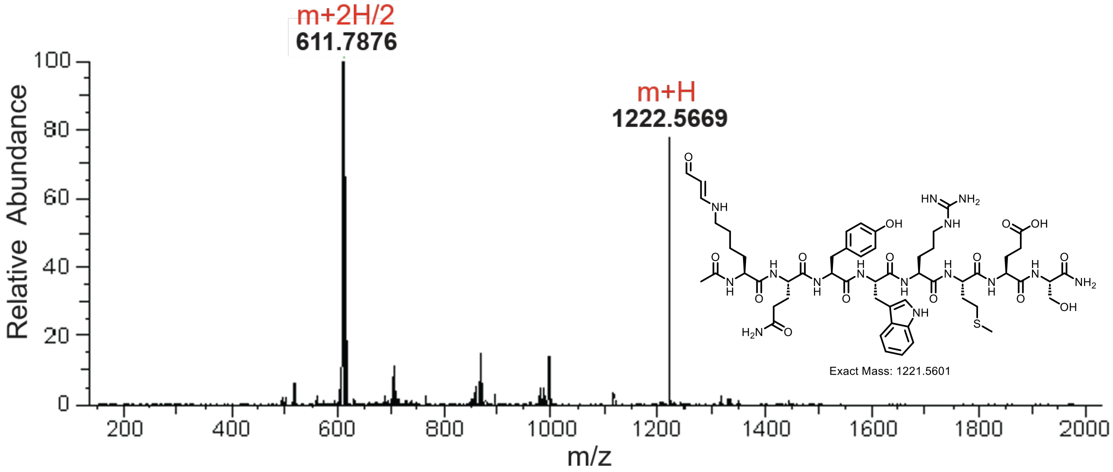

HRMS Trace of Peak at 12.5 min

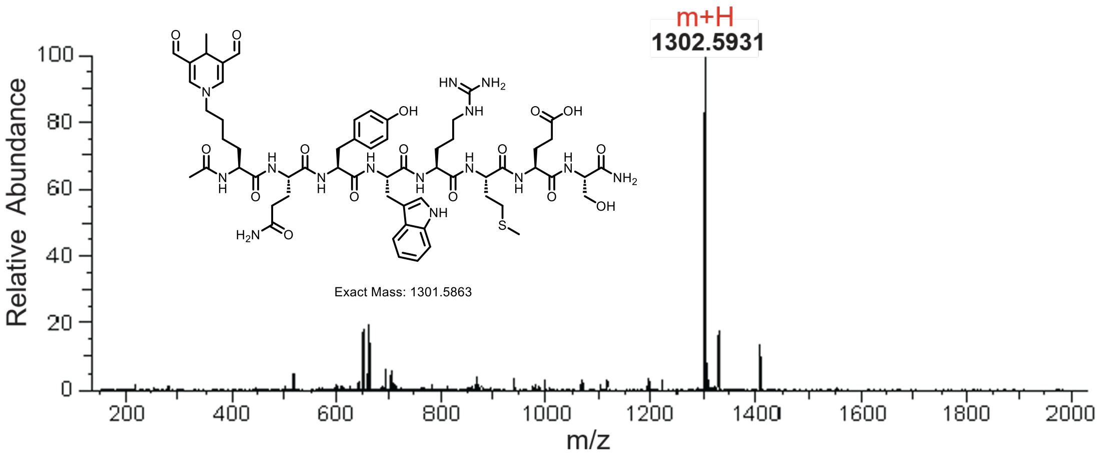

Peptide 1b.

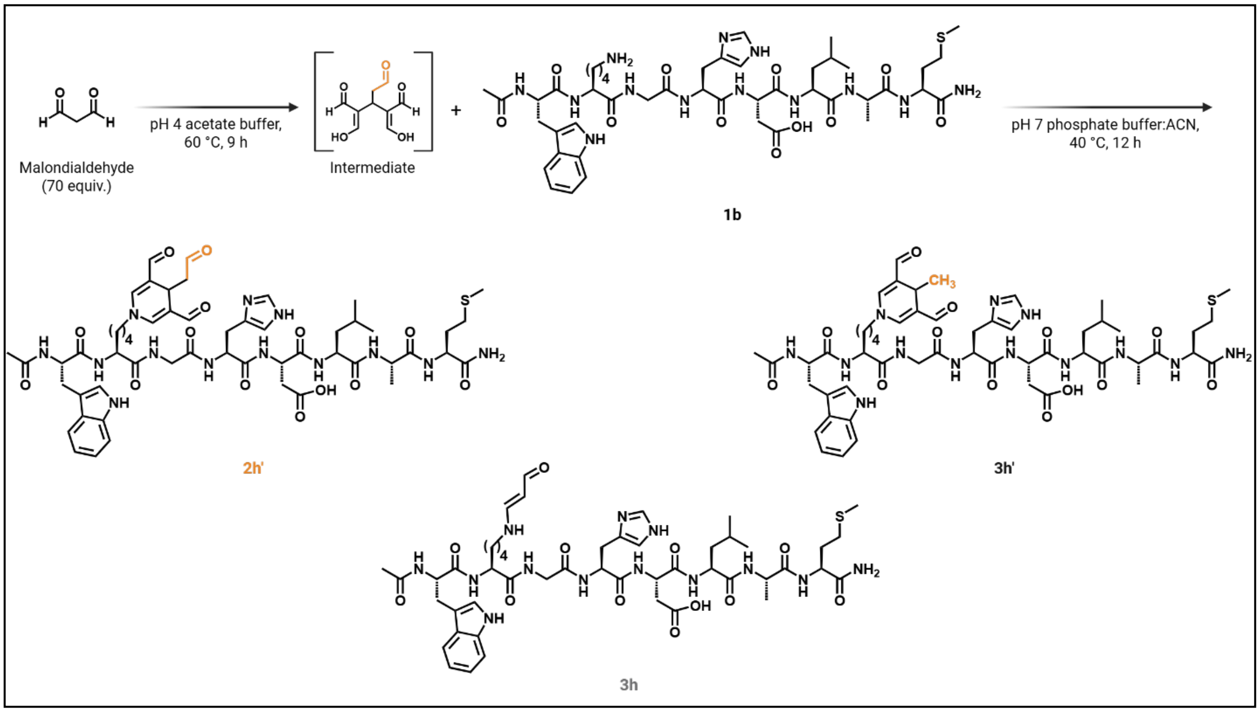

In a one-dram vial, MDA sodium salt (4.5 mg, 0.06 mmol) was dissolved in 100 µL of sodium acetate buffer (100 mM, pH 4) and left to stir at 60 °C for 9 hours. Then, peptide **1b** (Ac-WKGHDLAM-CONH$_2$) (1.0 mg, 0.001 mmol) was dissolved in 300 µL of sodium phosphate buffer (100 mM, pH 7) and 100 µL of acetonitrile, added to the reaction mixture and left to stir at 37 °C for 12 hours. Subsequently, the reaction mixture was injected into the HPLC for determining the % conversion of peptide **1b** to the labeled peptides **2h'**, **3h**, or **3h'** and their mass confirmed with LC-MS. HPLC analysis was carried out utilizing **HPLC Method A** at detection wavelength 220 nm. The masses of the products were confirmed with LC-MS and compiled below. The conversion was determined to be 34% of **2h'**, 39% of **3h**, and 27% **3h'**.

Ac-WKGHDLAM-CONH$_2$ ($C_{45}H_{67}N_{13}O_{11}S$) peptide 1b. LCMS: *m/z* 998.4881 (calcd $[M+H]^+$ = 998.4876) *m/z* 499.7474 (calcd $[M+2H/2]^+$ = 499.7475) (HPLC analysis at 220 nm). Retention time in HPLC: 7.8 min

Reaction Mixture:

Ac-WKGHDLAM-CONH$_2$ ($C_{54}H_{73}N_{13}O_{14}S$) labeled product 2h'. LCMS: *m/z* 1160.5194 (calcd $[M+H]^+$ = 1160.5193), *m/z* 580.7632 (calcd $[M+2H/2]^+$ = 580.7633) (HPLC analysis at 220 nm). Retention time in HPLC: 10.3 min. (34%)

Ac-WKGHDLAM-CONH$_2$ ($C_{48}H_{69}N_{13}O_{12}S$) side product 3h. LCMS: *m/z* 1052.4976 (calcd $[M+H]^+$ = 1052.4982), *m/z* 526.7525 (calcd $[M+2H/2]^+$ = 526.7528) (HPLC analysis at 220 nm). Retention time in HPLC: 11.3 min. (39%)

Ac-WKGHDLAM-CONH$_2$ ($C_{53}H_{73}N_{13}O_{13}S$) labeled product 3h'. LCMS: *m/z* 1132.5242 (calcd $[M+H]^+$ = 1132.5244) (HPLC analysis at 220 nm). Retention time in HPLC: 12.4 min. (27%)

HPLC of Ac-WKGHDLAM-CONH₂ Starting Peptide at 220 nm

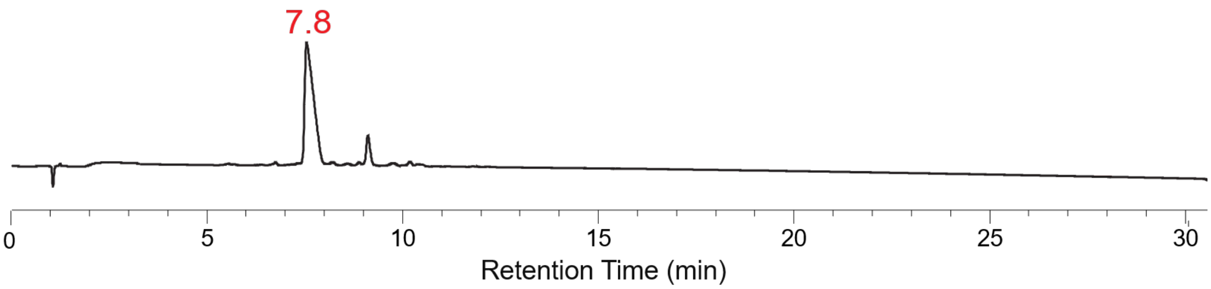

HRMS Trace of Peak at 7.8 min

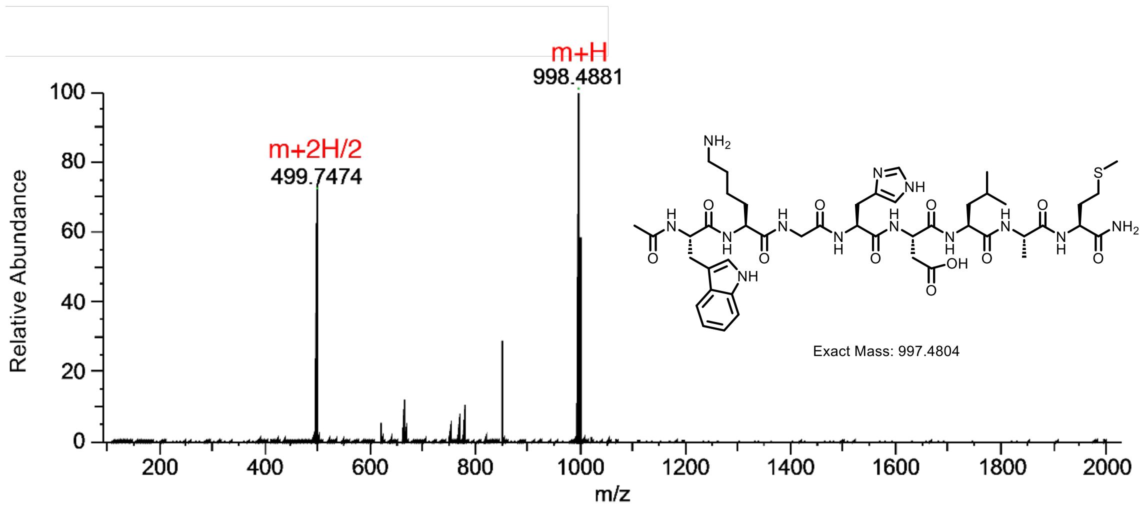

HPLC of Ac-WKGHDLAM-CONH₂ Reaction Mixture at 220 nm

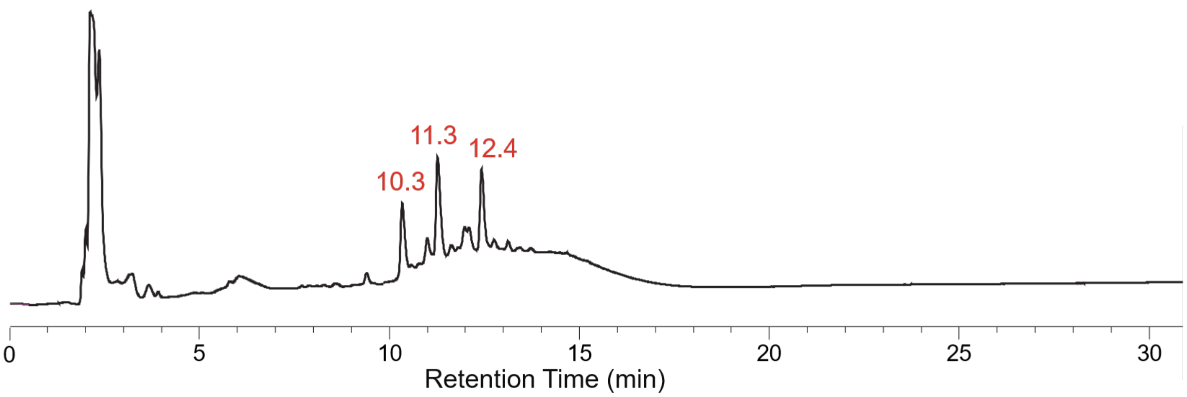

HRMS Trace of Peak at 10.3 min

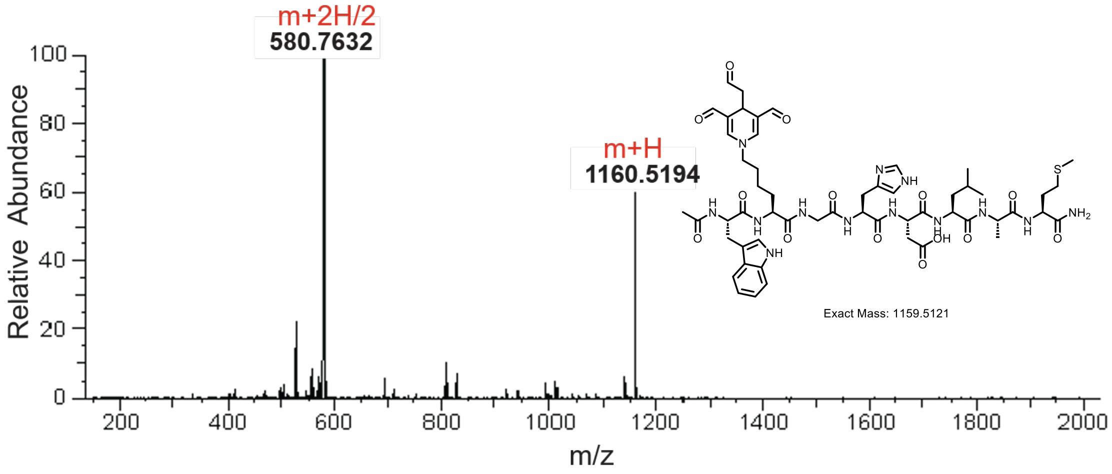

HRMS Trace of Peak at 11.3 min

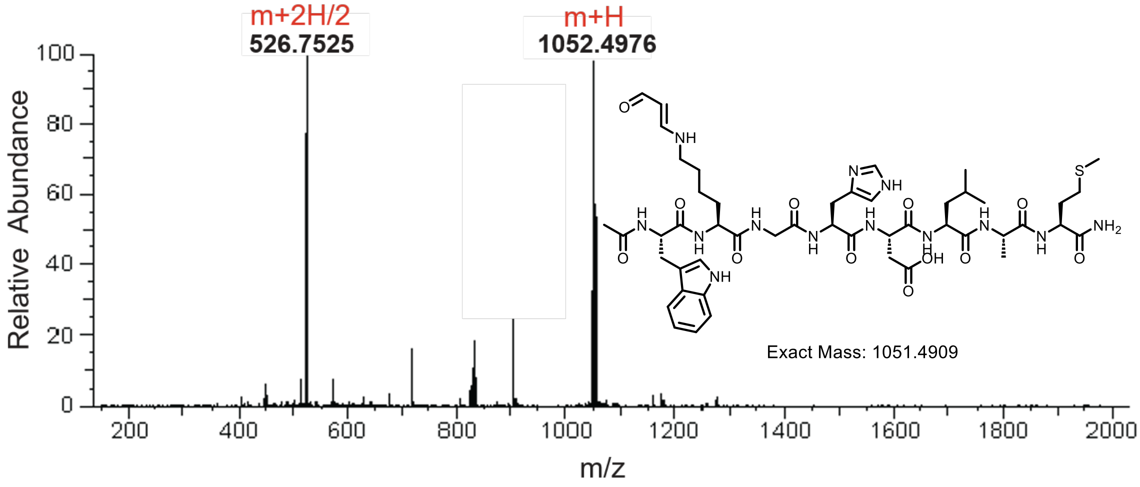

HRMS Trace of Peak at 12.4 min

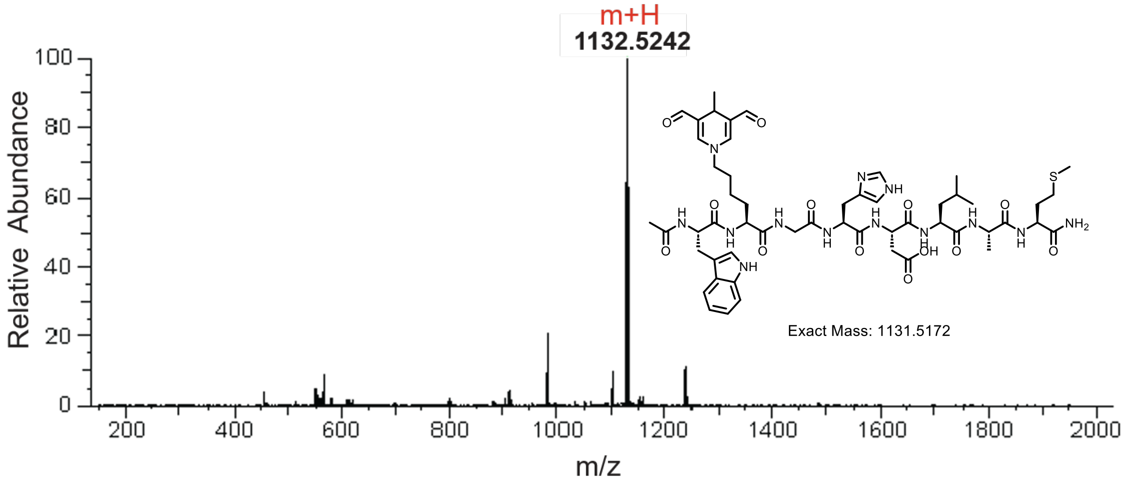

Supplementary Figure 7. Chemoselectivity Studies of MDA-monoaldehyde Cooperative Complexes.

Peptide 1i. MDA-MDA Complex

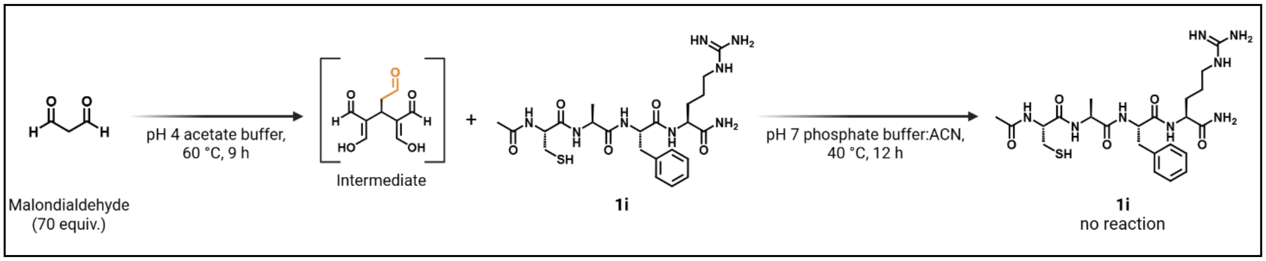

In a one-dram vial, MDA sodium salt (10.1 mg, 0.14 mmol) was dissolved in 100 µL of sodium acetate buffer (100 mM, pH 4) and left to stir at 60 °C for 9 hours. Then, peptide **1i** (Ac-CAFR-CONH$_2$) (1.0 mg, 0.002 mmol) was dissolved in 300 µL of sodium phosphate buffer (100 mM, pH 7) and 100 µL of acetonitrile, added to the reaction mixture and left to stir at 37 °C for 12 hours. Subsequently, the reaction mixture was injected into the HPLC for determining the % conversion of peptide **1i** and its mass confirmed with LC-MS. HPLC analysis was carried out utilizing **HPLC Method A** at detection wavelength 220 nm. The mass of the product was confirmed with LC-MS and compiled below.

Ac-CAFR-CONH$_2$ ($C_{23}H_{35}N_7O_6S$) peptide 1i. LCMS: *m/z* 537.2595 (calcd $[M+H]^+$ = 537.2602) (HPLC analysis at 220 nm). Retention time in HPLC: 7.5 min

Ac-CAFR-CONH$_2$ ($C_{46}H_{70}N_{16}O_{10}S_2$) peptide 1i (disulfide). LCMS: *m/z* 1071.4971 (calcd $[M+H]^+$ = 1071.4975), *m/z* 536.2528 (calcd $[M+2H/2]^+$ = 536.2524) (HPLC analysis at 220 nm). Retention time in HPLC: 10.1 min

Reaction Mixture:

Ac-CAFR-CONH$_2$ ($C_{46}H_{70}N_{16}O_{10}S_2$) peptide 1i (disulfide). LCMS: *m/z* 1071.4958 (calcd $[M+H]^+$ = 1071.4975), *m/z* 536.2520 (calcd $[M+2H/2]^+$ = 536.2524) (HPLC analysis at 220 nm). Retention time in HPLC: 10.1 min

HPLC of Ac-CAFR-CONH$_2$ Starting Peptide at 220 nm

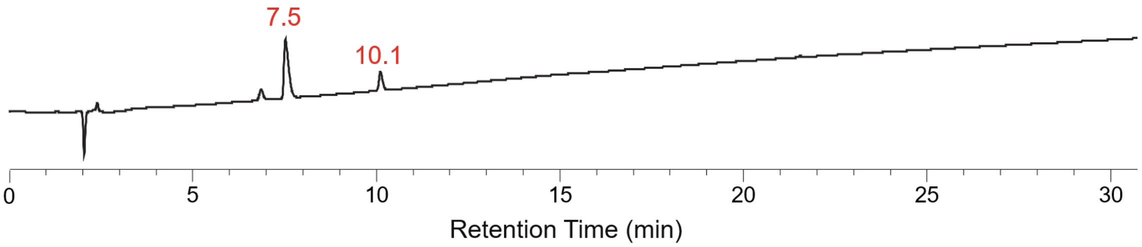

HRMS Trace of Peak at 7.5 min

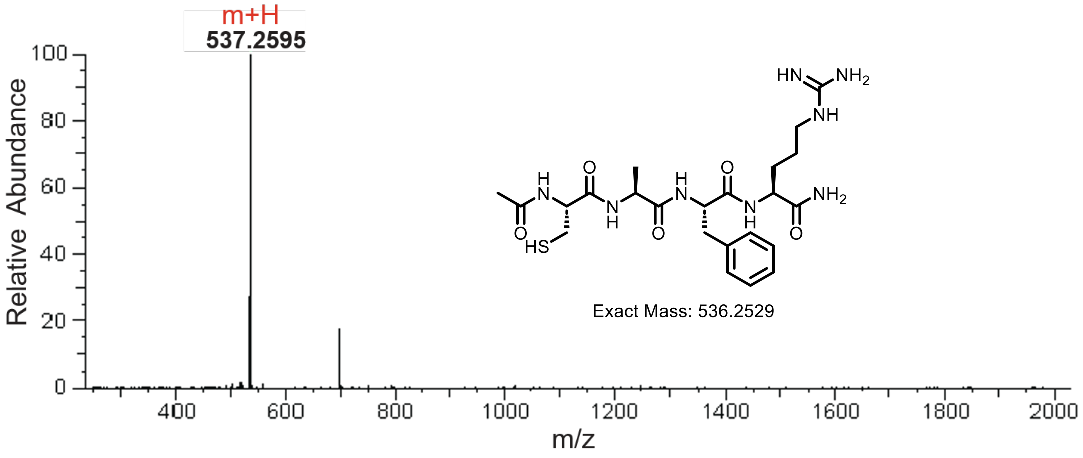

HRMS Trace of Peak at 10.1 min

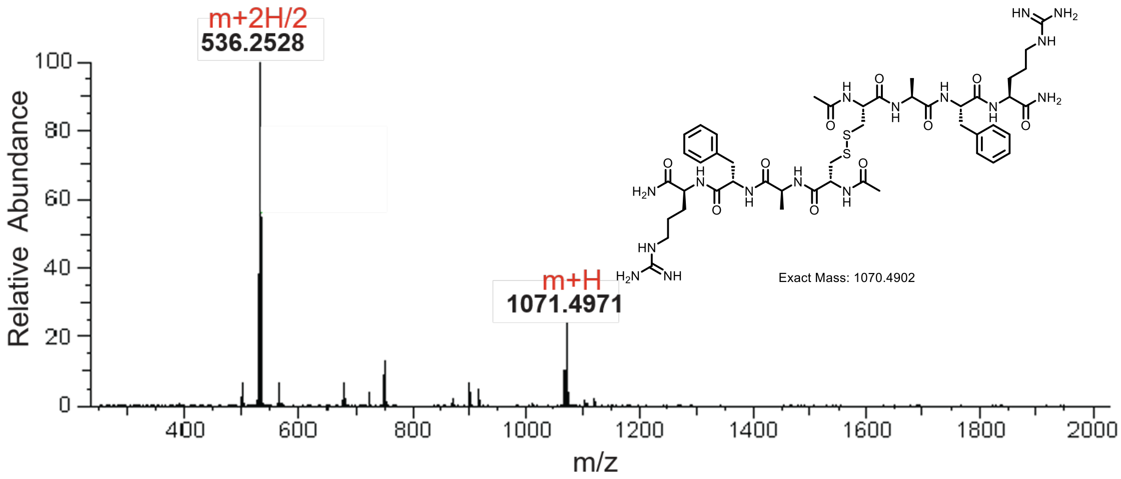

HPLC of Ac-CAFR-$CONH_2$ Reaction Mixture at 220 nm

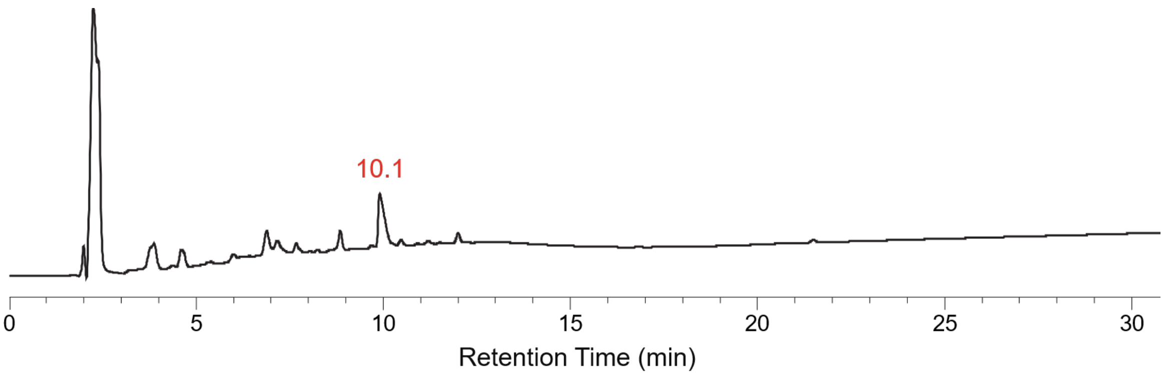

HRMS Trace of Peak at 10.1 min

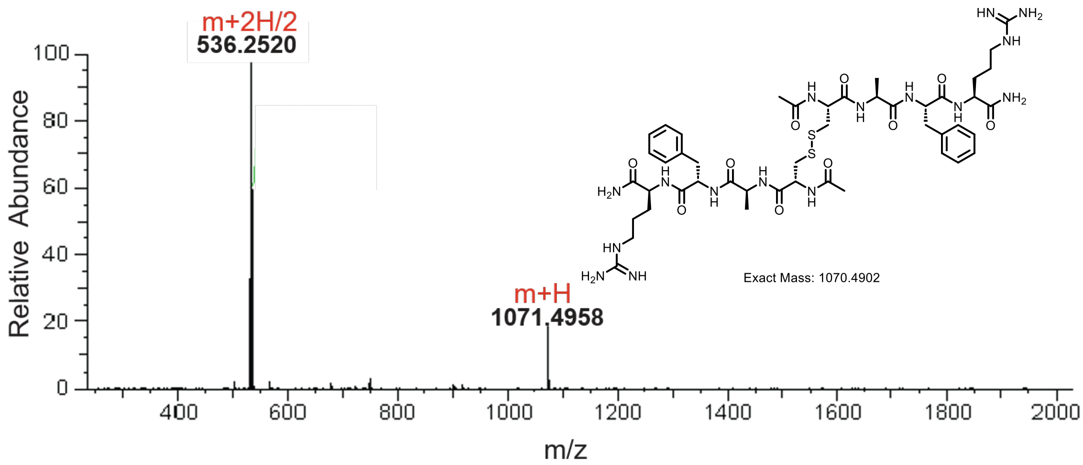

Peptide 1j. MDA-MDA Complex

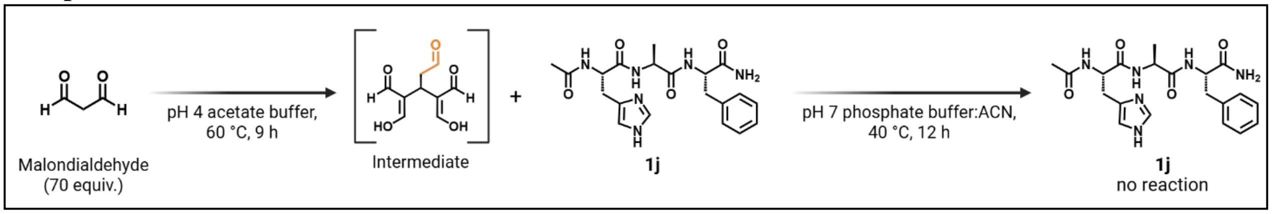

In a one-dram vial, MDA sodium salt (10.1 mg, 0.14 mmol) was dissolved in 100 µL of sodium acetate buffer (100 mM, pH 4) and left to stir at 60 °C for 9 hours. Then, peptide **1j** (Ac-HAF-$CONH_2$) (1.0 mg, 0.002 mmol) was dissolved in 300 µL of sodium phosphate buffer (100 mM, pH 7) and 100 µL of acetonitrile, added to the reaction mixture and left to stir at 37 °C for 12 hours. Subsequently, the reaction mixture was injected into the HPLC for determining the % conversion of peptide **1j** and its mass confirmed with LC-MS. HPLC analysis was carried out utilizing **HPLC Method A** at detection wavelength 220 nm. The mass of the product was confirmed with LC-MS and compiled below.

Ac-HAF-$CONH_2$ ($C_{20}H_{26}N_6O_4$) peptide 1j. LCMS: *m/z* 415.2086 (calcd $[M+H]^+$ = 415.2088) (HPLC analysis at 220 nm). Retention time in HPLC: 5.4 min

Reaction Mixture:

Ac-HAF-$CONH_2$ ($C_{20}H_{26}N_6O_4$) peptide 1j. LCMS: *m/z* 415.2087 (calcd $[M+H]^+$ = 415.2088), *m/z* 437.1905 (calcd [M+Na] = 437.1908) (HPLC analysis at 220 nm). Retention time in HPLC: 5.4 min

HPLC of Ac-HAF-$CONH_2$ Starting Peptide at 220 nm

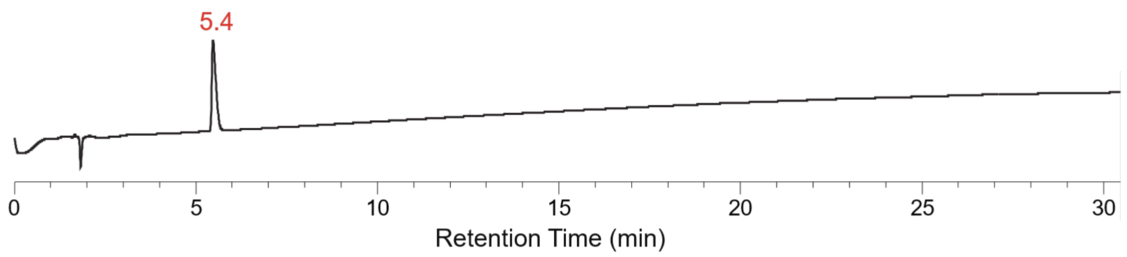

HRMS Trace of Peak at 5.4 min

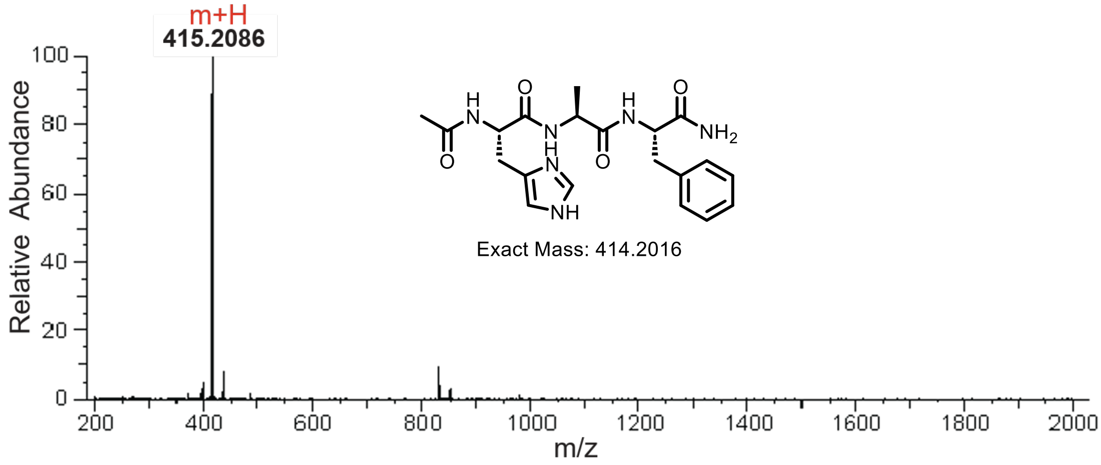

HPLC of Ac-HAF-CONH$_2$ Reaction Mixture at 220 nm

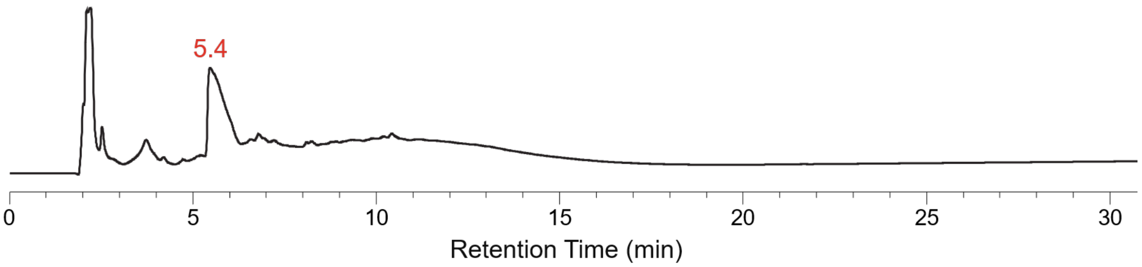

HRMS Trace of Peak at 5.4 min

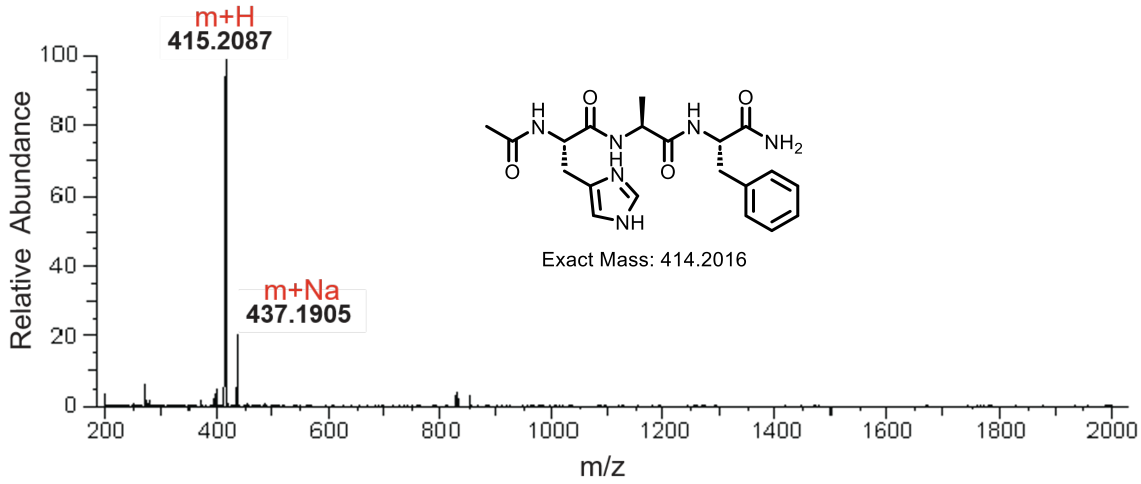

Supplementary Figure 8. Late-Stage Functionalization of Peptides.

Peptide 4b.

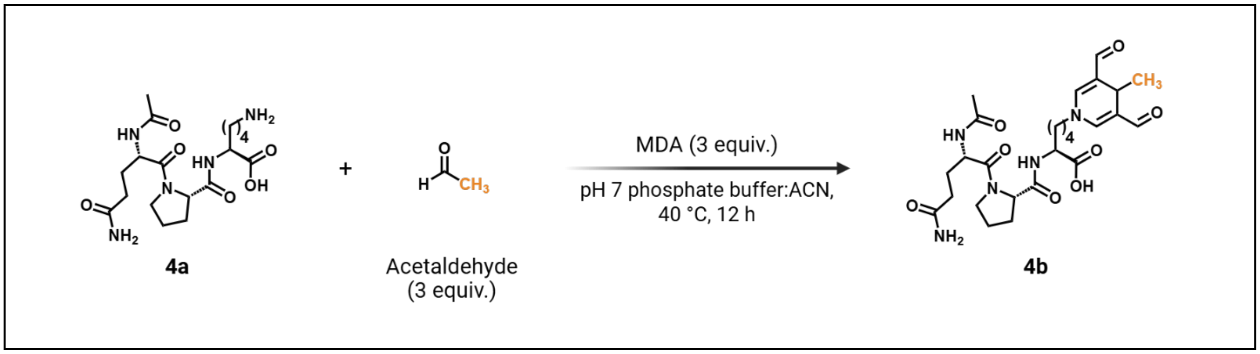

In a one-dram vial, peptide **4a** (Ac-QPK-COOH) (5 mg, 0.012 mmol) was dissolved in 500 µL of sodium phosphate buffer (100 mM, pH 7) and 500 µL of acetonitrile followed by the addition of MDA sodium salt (3 equiv.) and CH_3CHO (3 equiv.) and left to stir at 37 °C for 12 hours. Subsequently, the reaction mixture was injected into the HPLC for purification of the labeled peptide **4b**. HPLC analysis was carried out utilizing **HPLC Method A** at detection wavelength 220 nm.

Ac-QPK-COOH ($C_{18}H_{31}N_5O_6$) peptide 4a. LCMS: *m/z* 414.2343 (calcd $[M+H]^+$ = 414.2347) (HPLC analysis at 220 nm). Retention time in HPLC: 3.1 min

Purified Product:

Ac-QPK-COOH ($C_{26}H_{37}N_5O_8$) labeled product 4b. LCMS: *m/z* 548.2718 (calcd $[M+H]^+$ = 548.2715) (HPLC analysis at 220 nm). Retention time in HPLC: 9.8 min

HPLC of Ac-QPK-COOH Starting Peptide at 220 nm

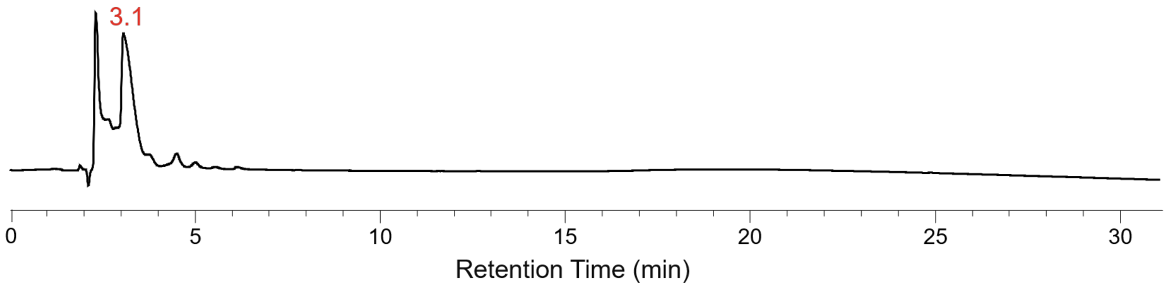

HRMS Trace of Peak at 3.1 min

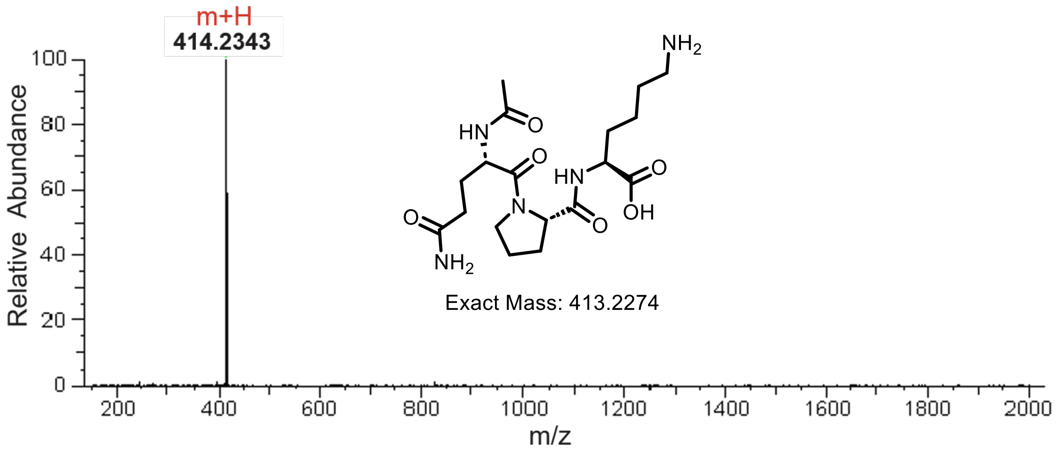

HPLC of Ac-QPK-COOH Peptide 4b at 220 nm

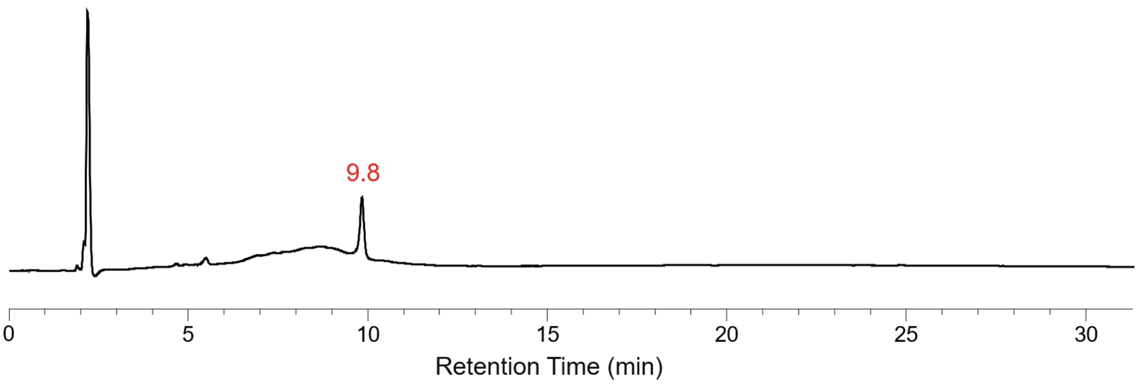

HRMS Trace of Peak at 9.8 min

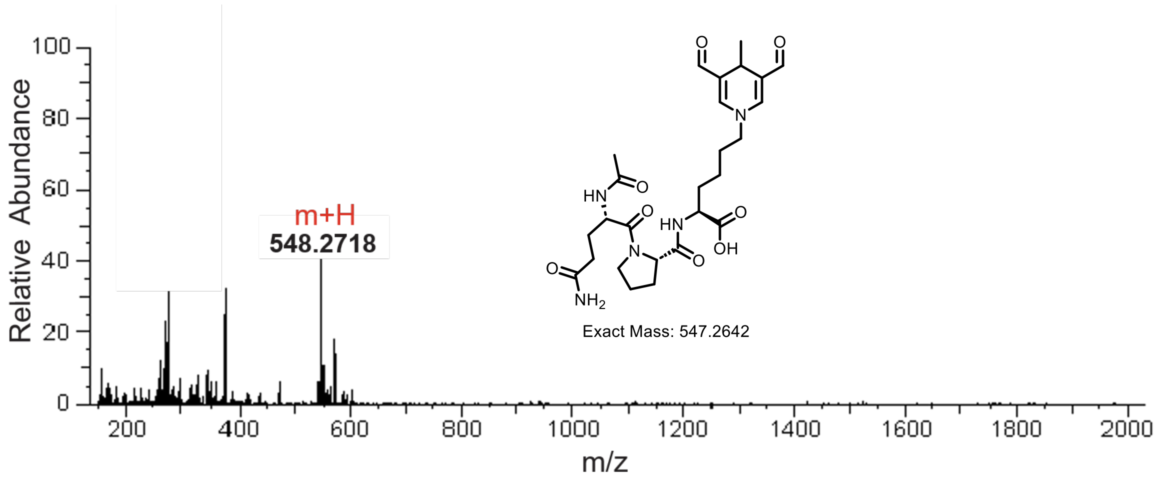

Peptide 4c.

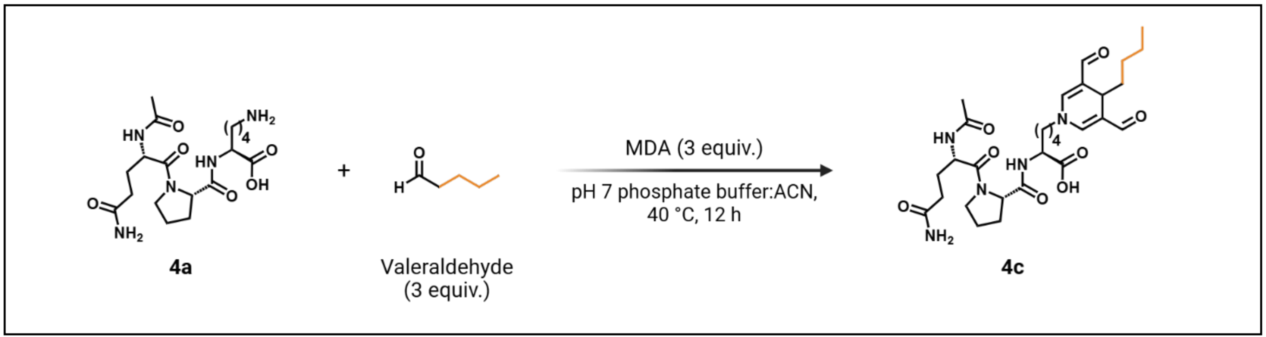

In a one-dram vial, peptide **4a** (Ac-QPK-COOH) (5 mg, 0.012 mmol) was dissolved in 500 µL of sodium phosphate buffer (100 mM, pH 7) and 500 µL of acetonitrile followed by the addition of MDA sodium salt (3 equiv.) and valeraldehyde (3 equiv.) and left to stir at 37 °C for 12 hours. Subsequently, the reaction mixture was injected into the HPLC for purification of the labeled peptide **4c**. HPLC analysis was carried out utilizing **HPLC Method A** at detection wavelength 220 nm.

Ac-QPK-COOH ($C_{29}H_{43}N_5O_8$) labeled product 4c. LCMS: *m/z* 590.3187 (calcd $[M+H]^+$ = 590.3184), *m/z* 608.3292 (calcd $[M+H+H_2O]^+$ = 608.3290) (HPLC analysis at 220 nm). Retention time in HPLC: 10.1 min

HPLC of Ac-QPK-COOH Peptide 4c at 220 nm

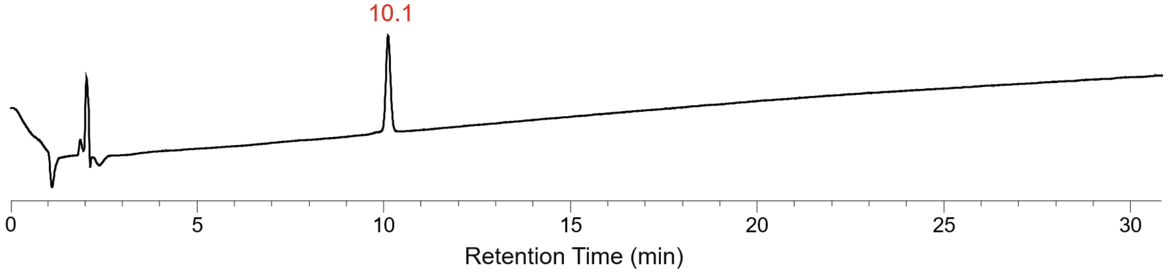

HRMS Trace of Peak at 10.1 min

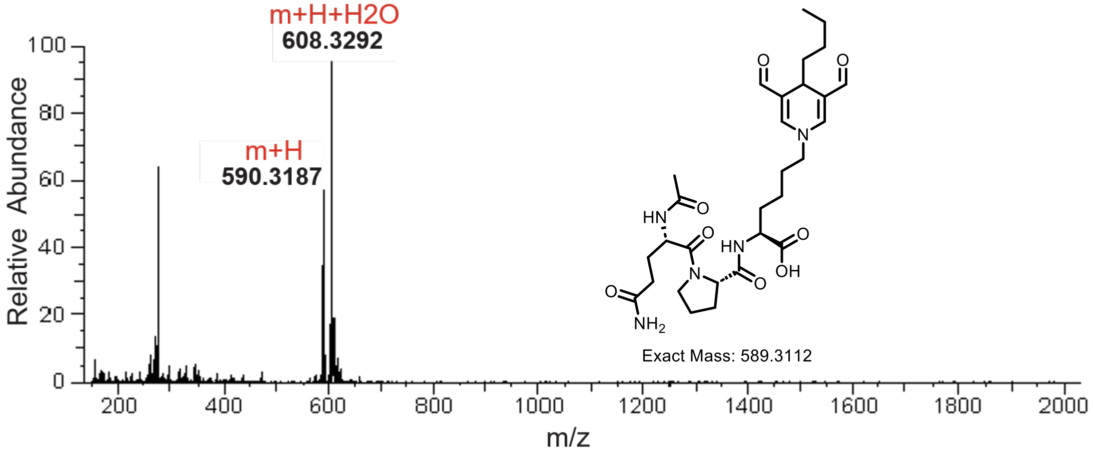

Peptide 4d.

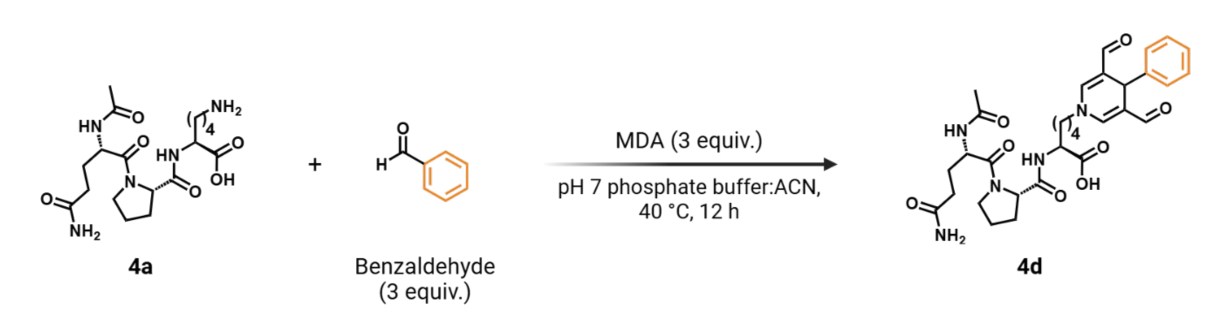

In a one-dram vial, peptide **4a** (Ac-QPK-COOH) (5 mg, 0.012 mmol) was dissolved in 500 µL of sodium phosphate buffer (100 mM, pH 7) and 500 µL of acetonitrile followed by the addition of MDA sodium salt (3 equiv.) and benzaldehyde (3 equiv.) and left to stir at 37 °C for 12 hours. Subsequently, the reaction mixture was injected into the HPLC for purification of the labeled peptide **4d**. HPLC analysis was carried out utilizing **HPLC Method A** at detection wavelength 220 nm.

Ac-QPK-COOH ($C_{31}H_{39}N_5O_8$) labeled product 4d. LCMS: *m/z* 610.2872 (calcd $[M+H]^+$ = 610.2871) (HPLC analysis at 220 nm). Retention time in HPLC: 11.8 min

HPLC of Ac-QPK-COOH Peptide 4d at 220 nm

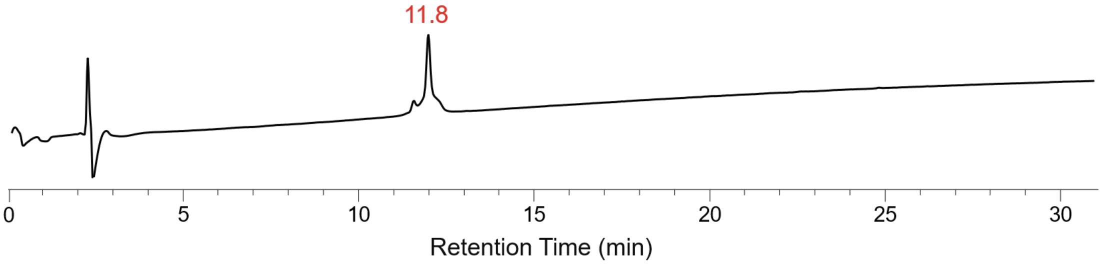

HRMS Trace of Peak at 11.8 min

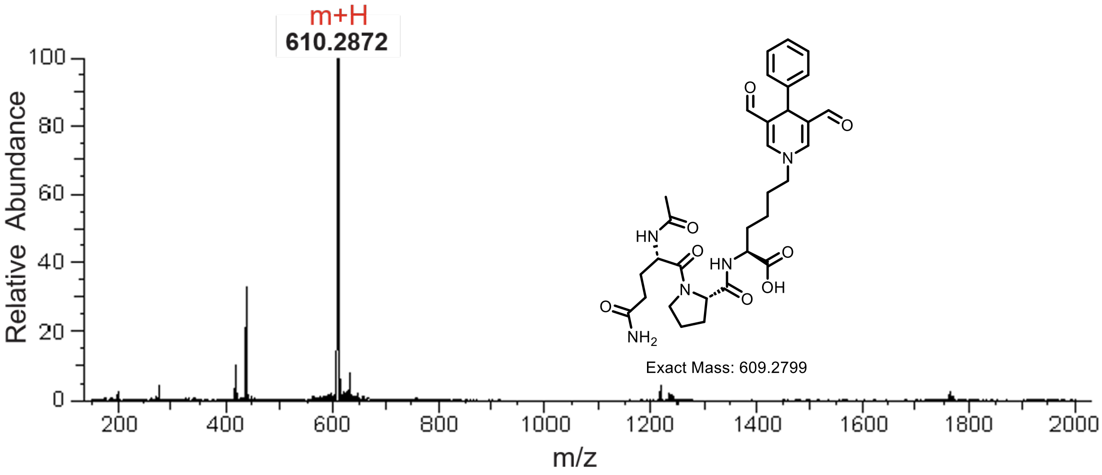

Peptide 4e.

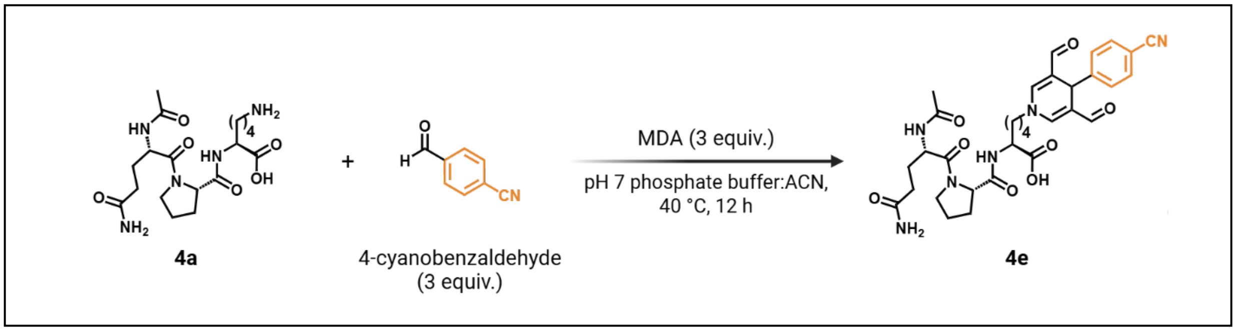

In a one-dram vial, peptide **4a** (Ac-QPK-COOH) (5 mg, 0.012 mmol) was dissolved in 500 µL of sodium phosphate buffer (100 mM, pH 7) and 500 µL of acetonitrile followed by the addition of MDA sodium salt (3 equiv.) and 4-cyanobenzaldehyde (3 equiv.) and left to stir at 37 °C for 12 hours. Subsequently, the reaction mixture was injected into the HPLC for purification of the labeled peptide **4e**. HPLC analysis was carried out utilizing **HPLC Method A** at detection wavelength 220 nm.

Ac-QPK-COOH ($C_{32}H_{38}N_6O_8$) labeled product 4e. LCMS: *m/z* 635.2825 (calcd $[M+H]^+$ = 635.2824), *m/z* 653.2931 (calcd $[M+H+H_2O]^+$ = 635.2930) (HPLC analysis at 220 nm). Retention time in HPLC: 12.3 min

HPLC of Ac-QPK-COOH Peptide 4e at 220 nm

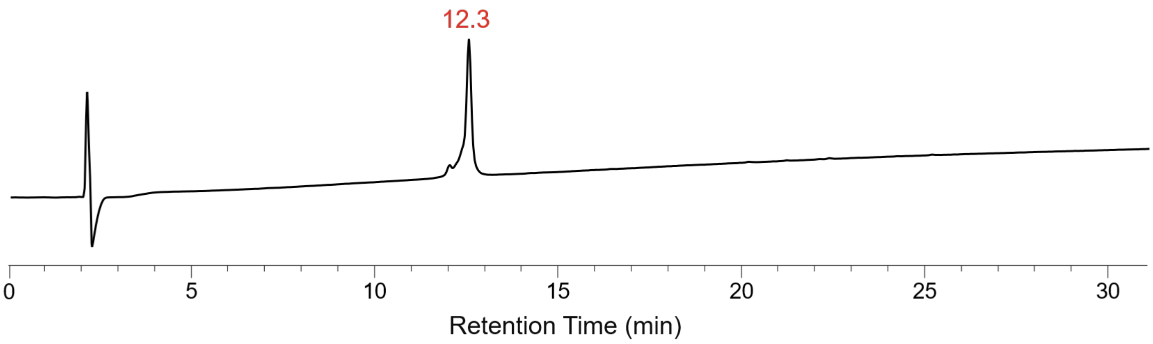

HRMS Trace of Peak at 12.3 min

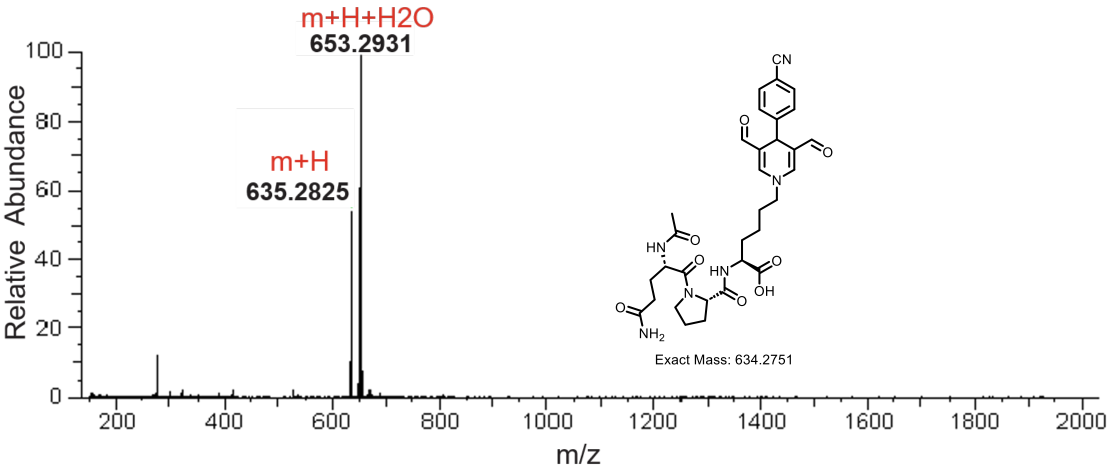

Peptide 4f.

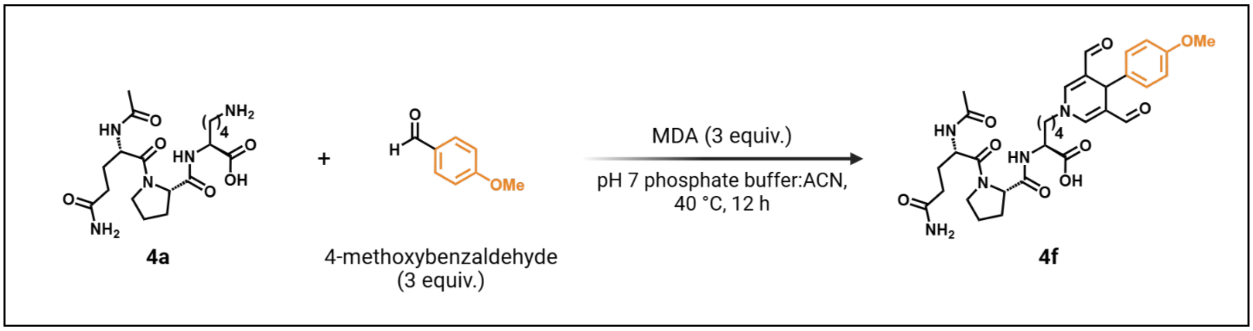

In a one-dram vial, peptide **4a** (Ac-QPK-COOH) (5 mg, 0.012 mmol) was dissolved in 500 µL of sodium phosphate buffer (100 mM, pH 7) and 500 µL of acetonitrile followed by the addition of MDA sodium salt (3 equiv.) and 4-methoxybenzaldehyde (3 equiv.) and left to stir at 37 °C for 12 hours. Subsequently, the reaction mixture was injected into the HPLC for purification of the labeled peptide **4f**. HPLC analysis was carried out utilizing **HPLC Method A** at detection wavelength 220 nm.

Ac-QPK-COOH ($C_{32}H_{41}N_5O_9$) labeled product 4f. LCMS: *m/z* 640.2977 (calcd $[M+H]^+$ = 640.2977) (HPLC analysis at 220 nm). Retention time in HPLC: 12.3 min

HPLC of Ac-QPK-COOH Peptide 4f at 220 nm

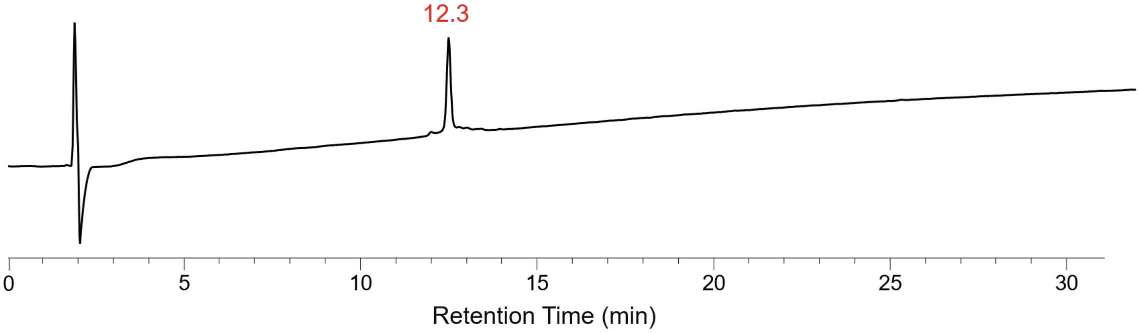

HRMS Trace of Peak at 12.3 min

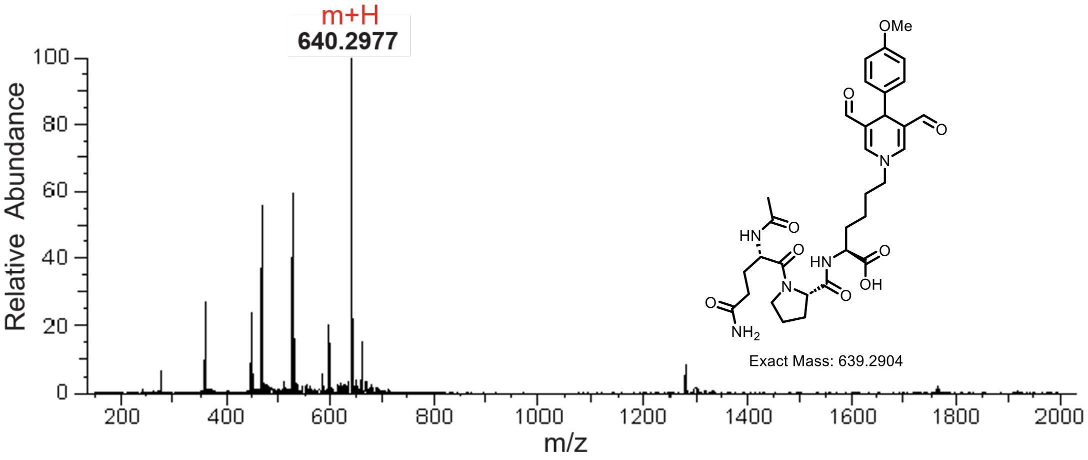

Peptide 4g.

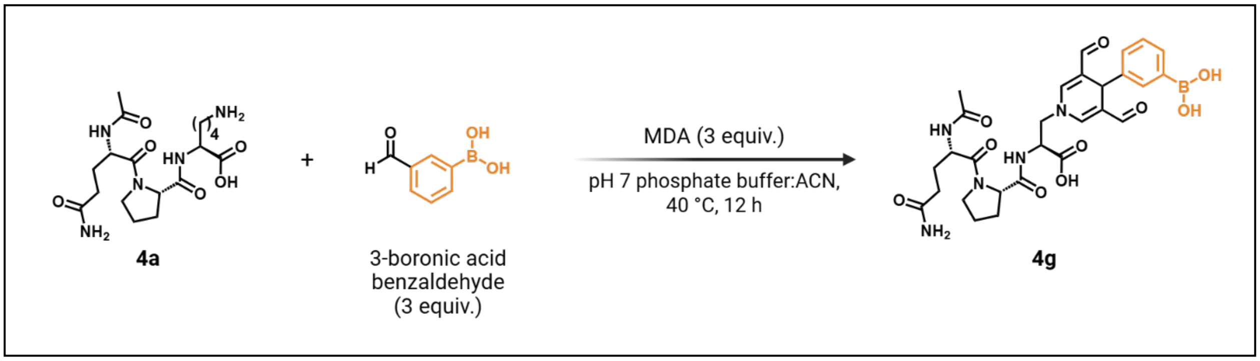

In a one-dram vial, peptide **4a** (Ac-QPK-COOH) (5 mg, 0.012 mmol) was dissolved in 500 µL of sodium phosphate buffer (100 mM, pH 7) and 500 µL of acetonitrile followed by the addition of MDA sodium salt (3 equiv.) and 3-boronic acid benzaldehyde (3 equiv.) and left to stir at 37 °C for 12 hours. Subsequently, the reaction mixture was injected into the HPLC for purification of the labeled peptide **4g**. HPLC analysis was carried out utilizing **HPLC Method A** at detection wavelength 220 nm. The mass of the product was confirmed with LC-MS and compiled below.

Ac-QPK-COOH ($C_{31}H_{40}BN_5O_{10}$) labeled product 4g. LCMS: *m/z* 654.2773 (calcd $[M+H]^+$ = 654.2941), *m/z* 672.2879 (calcd $[M+H+H_2O]^+$ = 672.3047) (HPLC analysis at 220 nm). Retention time in HPLC: 10.0 min

HPLC of Ac-QPK-COOH Peptide 4g at 220 nm

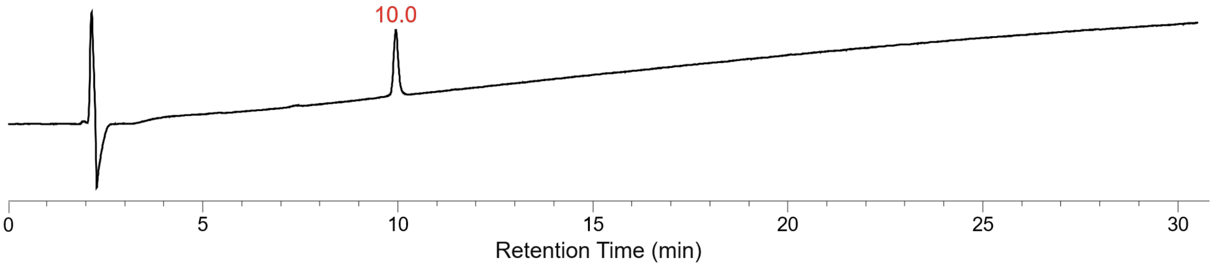

HRMS Trace of Peak at 10.0 min

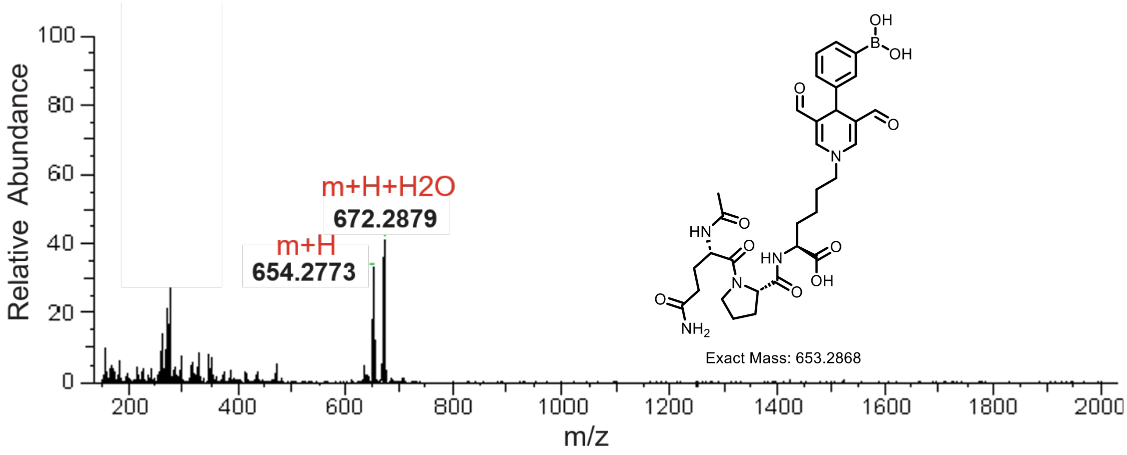

Supplementary Figure 9. Flow Cytometry of Ac-QPK-COOH Analogs (4a-4g).

HeLa cells were grown in 60 x 15 mm Nunclon™ dishes and allowed to adhere overnight in an incubator at 37 °C, 5% CO_2. Stock solutions of Ac-QPK-COOH and analogs were prepared in stock 10 mM DMSO solutions before being diluted to the final desired concentration of 25 µM in 3 mL of media. Vehicle controls were treated with equivalent dosage of DMSO. Cells were placed in an incubator for 48 hours. Cells were then detached with trypsin and stained using Annexin V/PI following manufacturer's protocol. To avoid fluorescent crosstalk, Annexin V (AV) conjugated to FITC was used to determine apoptosis. Propidium Iodide (PI) was used to determine necrosis within the cellular populations. Cells were analyzed via flow cytometry on a BD FACSympony A3 cell analyzer within 1 hour to quantify cell death. A B515/20 laser was used to detect AV-FITC while a B710/50 laser was used to detect PI. FlowJo software (v. 10.10.0) was used to analyze the cytometry data.

Data is an average of 3 replicates analyzed on separate days using different passage number of cells. Two-sided Student's t-Test was used to determine statistical significance. **Note**: Gating is based on the AV/PI controls; samples were analyzed respective to the controls on the day of analysis. Significant (~13%) vehicle cell death was observed due to overgrowth of cells over the 48-hour dosage period. All cell death was normalized to vehicle cell death at 0%.

Flow Gating Protocol

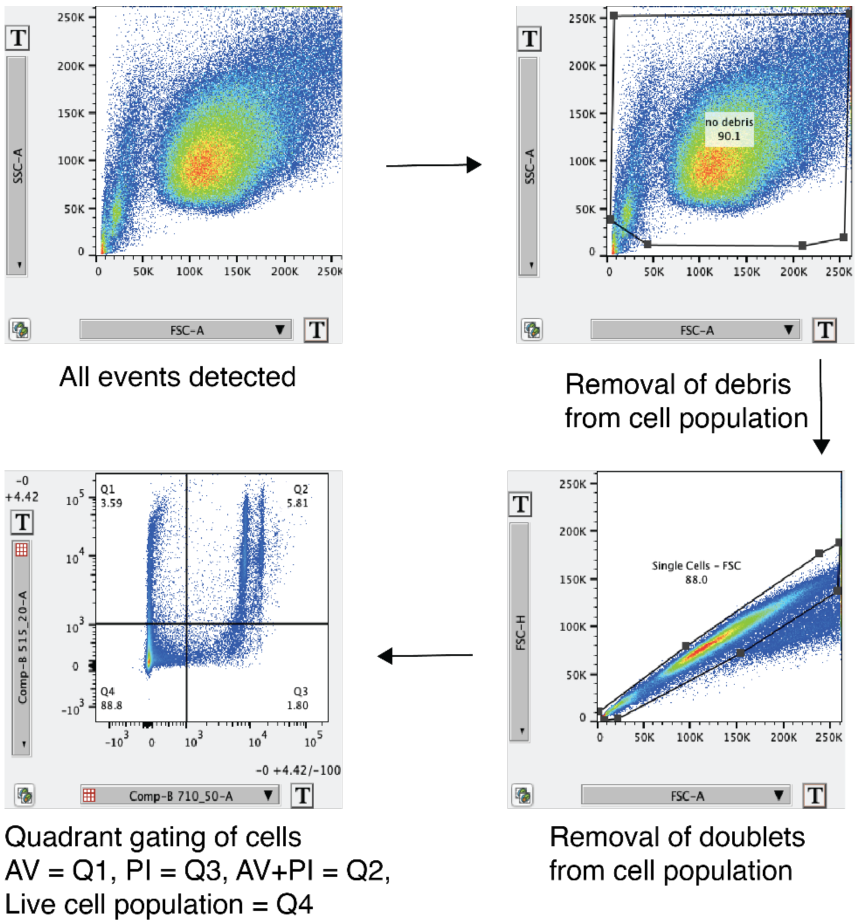

Representative Flow Cytometry Figure for Each Compound

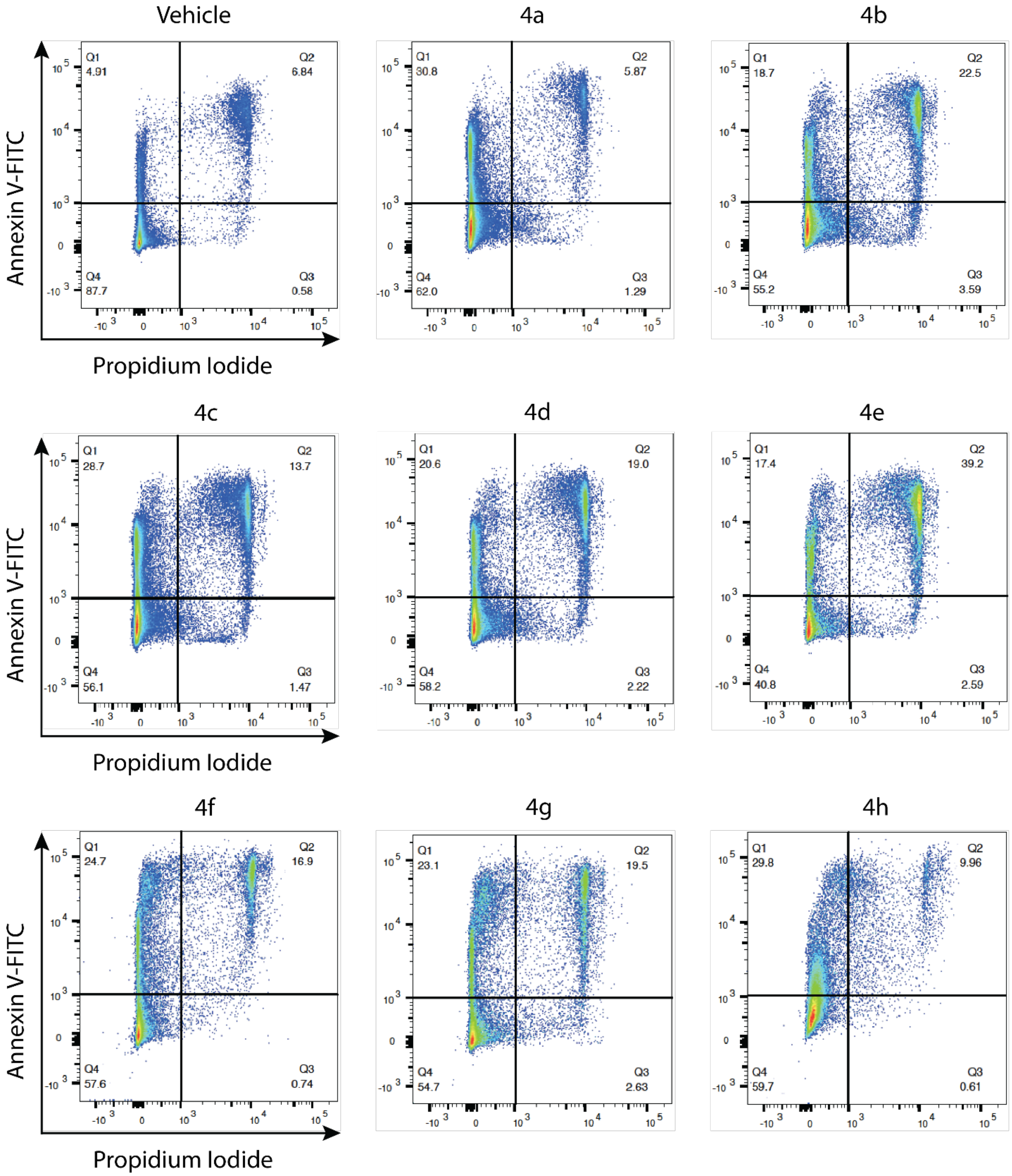

Supplementary Figure 10. Synthesis of DHP analogs with enhanced conjugation.

General procedure I for the synthesis of compounds 5b-5f:

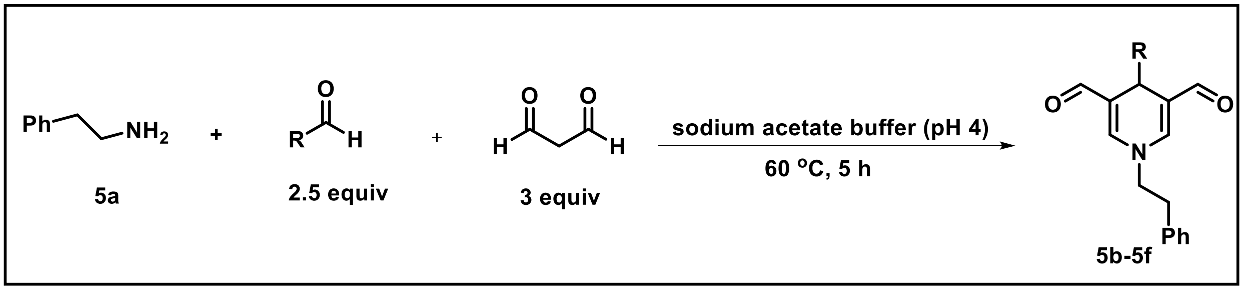

In 25 mL round-bottom flask, 2-phenylethylamine **5a** (200 mg, 1.65 mmol, 1 equiv.) was dissolved in 2 mL of sodium acetate buffer (100 mM, pH 4) followed by the addition of MDA sodium salt (3 equiv.) and corresponding aldehyde (2.5 equiv.). Then the pH of the reaction mixture was adjusted to 4.2. The reaction mixture was left to stir at 60 °C for 5 hours. After cooling the reaction mixture to room temperature, the reaction was neutralized with 2 M NaOH. Followed by workup using brine and extracted ethyl acetate (3 x 30 mL). DHP compound (**5b-5f**) was purified by column chromatography using hexane/ethyl acetate as eluent.

4-methyl-1-phenethyl-1,4-dihydropyridine-3,5-dicarbaldehyde (5b): Synthesized using general procedure I, using acetaldehyde. Compound **5b** was purified by column chromatography as a light-yellow solid (25% yield).

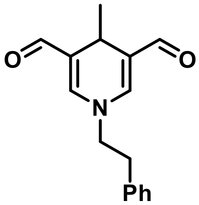

^{1}H NMR (400 MHz, $CDCl_3$): δ 9.07 (s, 2H), 7.39 – 7.17 (m, 3H), 7.15 – 7.01 (m, 2H), 6.37 (s, 2H), 3.80 (q, *J* = 6.6 Hz, 1H), 3.63 (t, *J* = 6.6 Hz, 2H), 2.92 (t, *J* = 6.6 Hz, 2H), 0.99 (d, *J* = 6.6 Hz, 3H). **^{13}C NMR (101 MHz, $CDCl_3$):** δ 188.8, 146.2, 136.7, 129.2, 129.1, 127.5, 123.7, 56.8, 36.8, 23.2, 22.2.

HRMS(ESI): *m/z* calcd for $C_{16}H_{18}NO_2$ $(M+H)^+$: 256.1332; found: 256.1328.

^{1}H NMR (400 MHz, $CDCl_3$) of compound **5b**

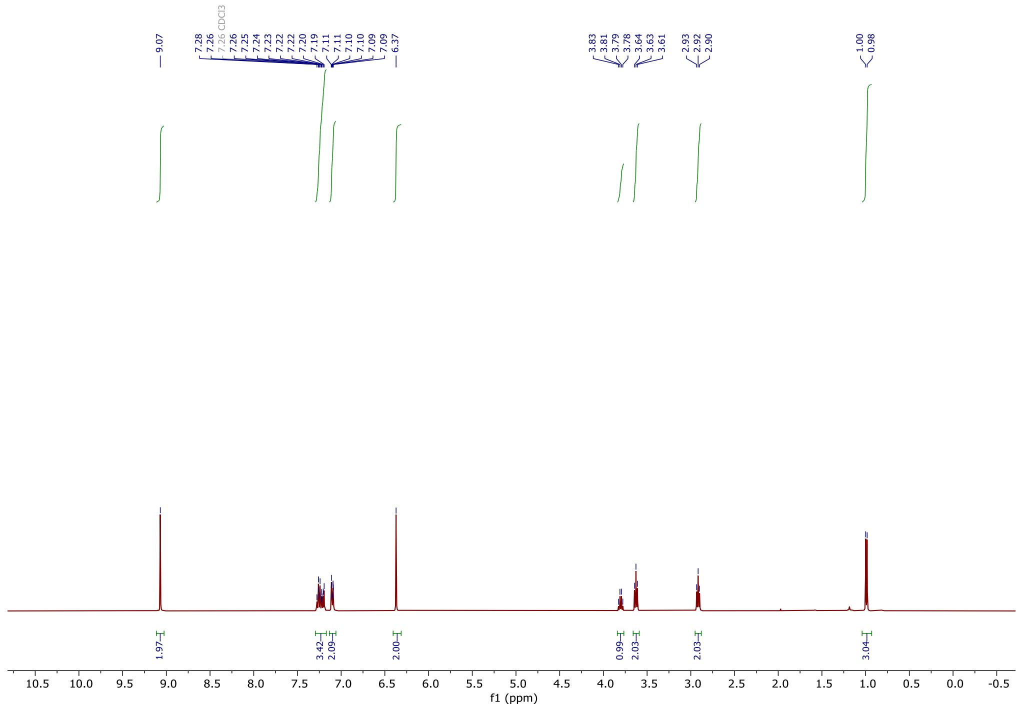

^{13}C NMR (101 MHz, $CDCl_3$) of compound **5b**

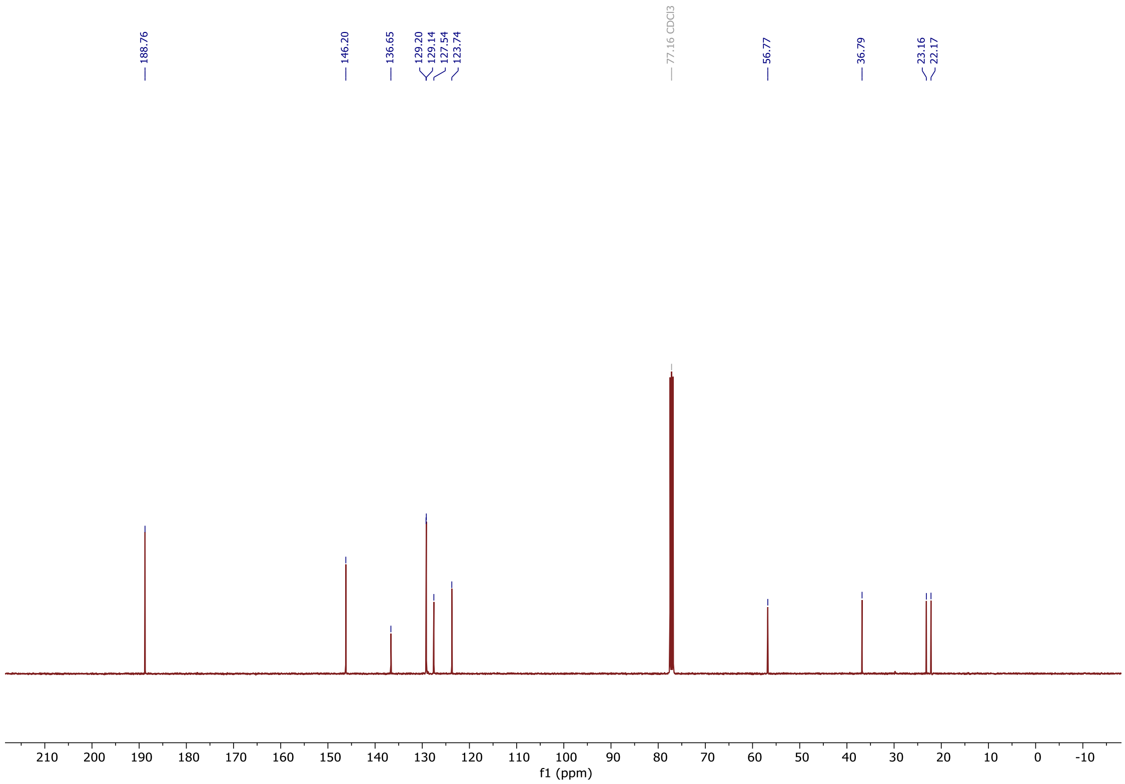

1-phenethyl-4-phenyl-1,4-dihydropyridine-3,5-dicarbaldehyde (5c): Synthesized using general procedure I, by using benzaldehyde. The compound **5c** is purified by column chromatography as an off-white solid (18% yield).

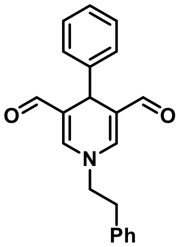

^{1}H NMR (400 MHz, $CDCl_3$): δ 9.16 (s, 2H), 7.39 – 7.29 (m, 3H), 7.24 – 7.18 (m, 6H), 7.16 – 7.10 (m, 1H), 6.66 (s, 2H), 4.97 (s, 1H), 3.82 (t, *J* = 6.8 Hz, 2H), 3.06 (t, *J* = 6.8 Hz, 2H). **^{13}C NMR (101 MHz, $CDCl_3$):** δ 188.4, 145.3, 144.5, 136.5, 129.3, 129.1, 128.4, 128.2, 127.6, 126.8, 122.1, 56.8, 36.7, 33.8.

HRMS(ESI): *m/z* calcd for $C_{21}H_{20}NO_2$ $(M+H)^+$: 318.1489; found: 318.1486.

^{1}H NMR (400 MHz, $CDCl_3$) of compound **5c**

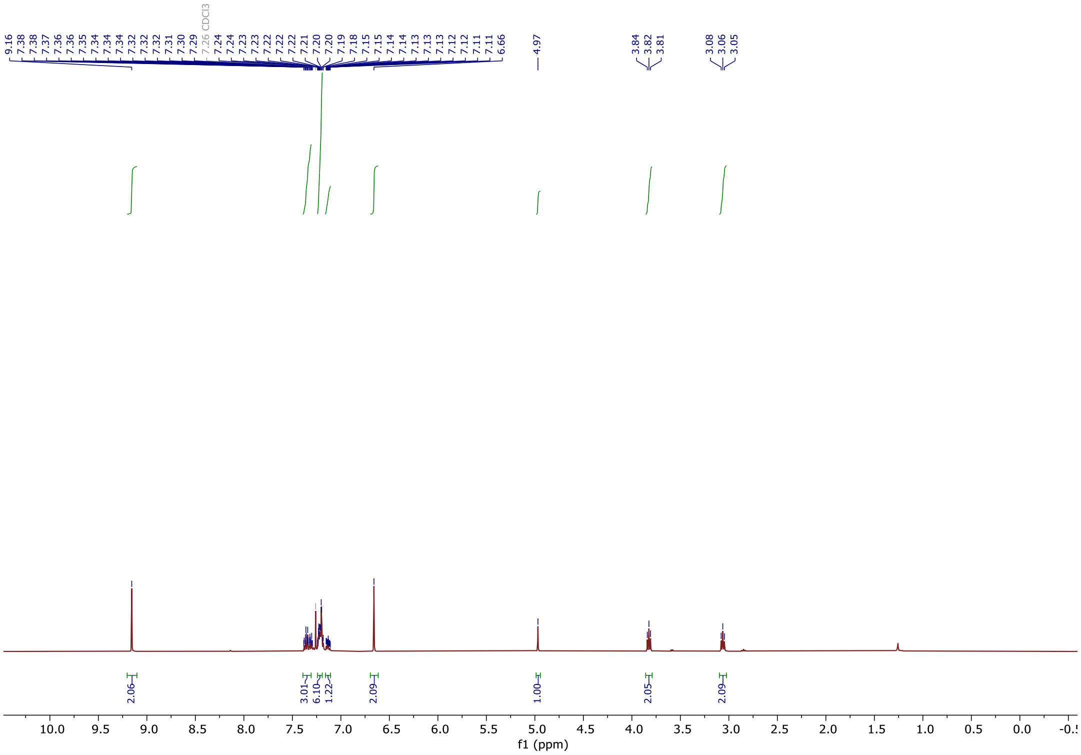

^{13}C NMR (101 MHz, $CDCl_3$) of compound **5c**

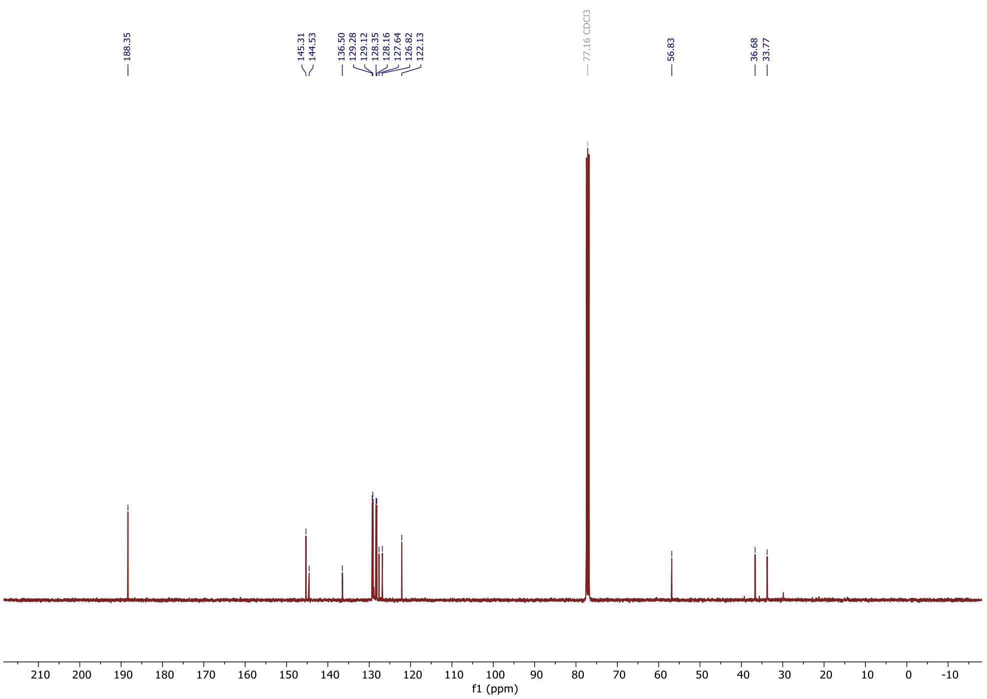

2-(3,5-diformyl-1-phenethyl-1,4-dihydropyridin-4-yl)benzonitrile (5d): Synthesized using general procedure I, by using 2-cyanobenzaldehyde. The compound **5d** is purified by column chromatography as an off-white solid (11% yield).

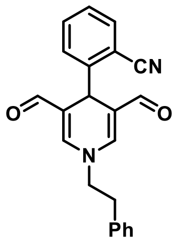

^{1}H NMR (400 MHz, $CDCl_3$): δ 9.10 (s, *J* = 0.6 Hz, 1H), 7.57 (dt, *J* = 7.6, 1.6 Hz, 2H), 7.48 (td, *J* = 7.6, 1.6 Hz, 1H), 7.40–7.33 (m, 2H), 7.33–7.26 (m, 1H), 7.26–7.19 (m, 3H), 6.72 (s, 2H), 5.17 (s, 1H), 3.85 (t, *J* = 7.0 Hz, 2H), 3.10 (t, *J* = 7.0 Hz, 2H). **^{13}C NMR (101 MHz, $CDCl_3$):** δ 188.2, 148.5, 146.7, 136.8, 134.0, 132.2, 132.1, 129.3, 129.1, 127.6, 127.2, 119.7, 118.2, 112.0, 56.8, 36.3, 35.8.

HRMS(ESI): *m/z* calcd for $C_{22}H_{19}N_2O_2$ $(M+H)^+$: 343.1441; found: 343.1442.

^{1}H NMR (400 MHz, CDCl₃) of compound **5d**

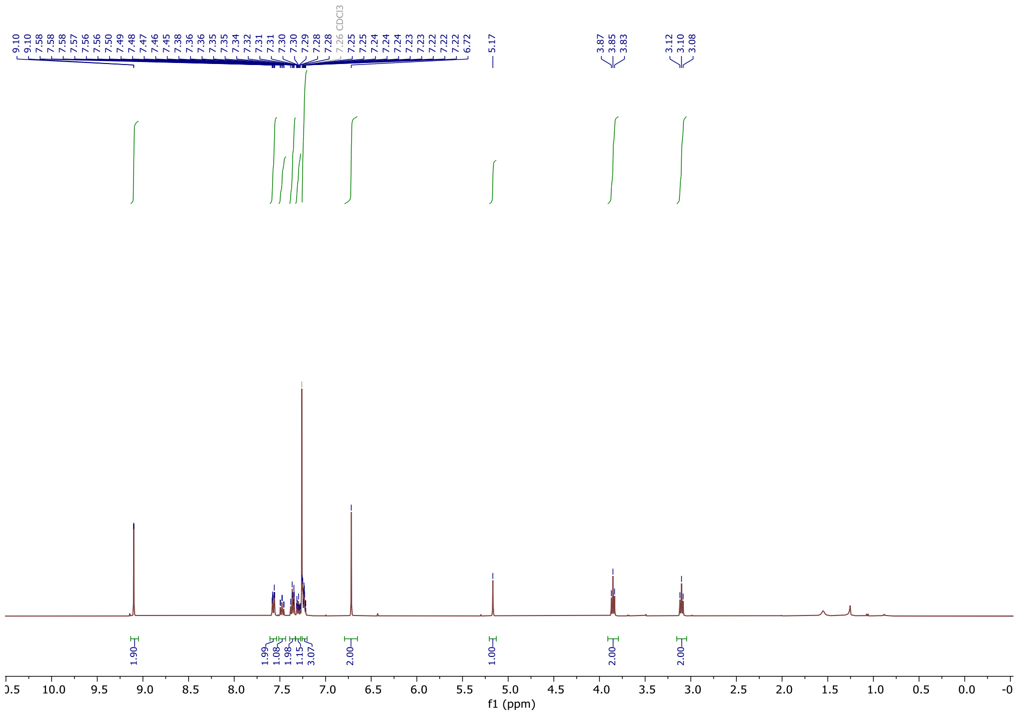

^{13}C NMR (101 MHz, CDCl₃) of compound **5d**

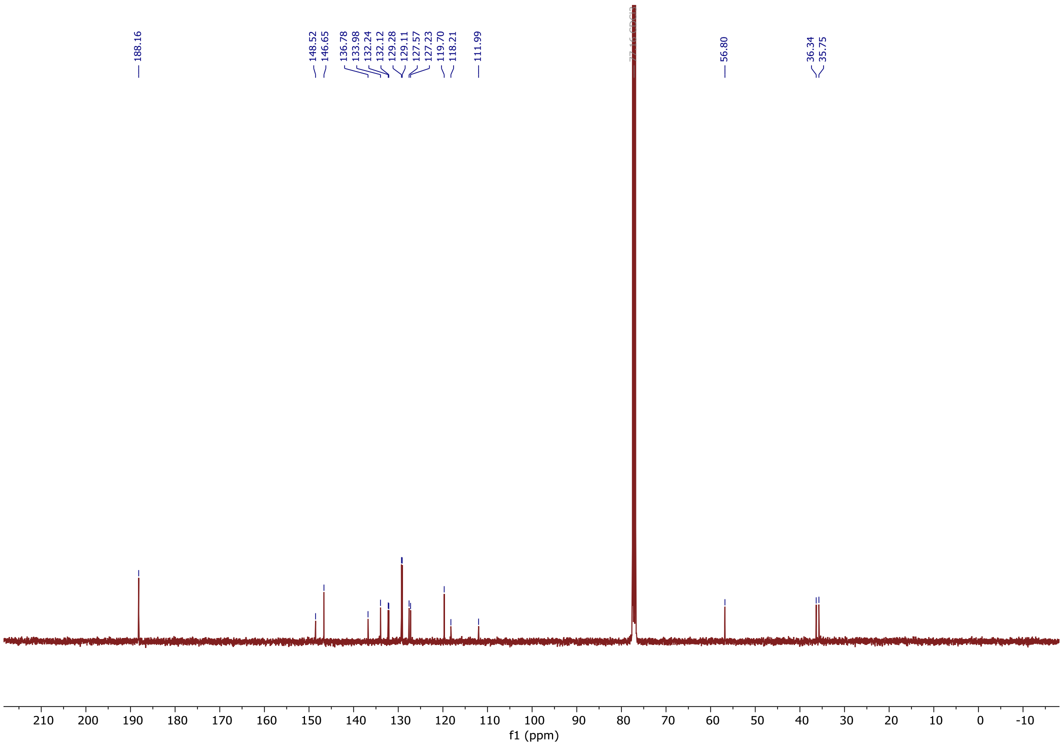

4-(4-nitrophenyl)-1-phenethyl-1,4-dihydropyridine-3,5-dicarbaldehyde (5e): Synthesized using general procedure I, by using 4-nitrobenzaldehyde. The compound **5e** is purified by column chromatography as a yellow liquid (12% yield).

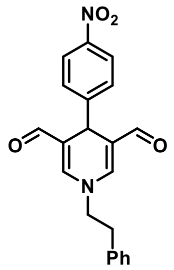

^{1}H NMR (400 MHz, $CDCl_3$): δ 9.14 (s, 2H), 8.06 (d, *J* = 8.8 Hz, 2H), 7.46 – 7.31 (m, 5H), 7.27 – 7.15 (m, 2H), 6.71 (s, 2H), 5.05 (s, 1H), 3.88 (t, *J* = 6.6 Hz, 2H), 3.10 (d, *J* = 6.6 Hz, 2H). **^{13}C NMR (101 MHz, $CDCl_3$):** δ 188.0, 151.3, 145.8, 136.2, 129.4, 129.2, 129.1, 127.9, 123.6, 121.1, 56.8, 36.6, 34.2, 29.9.

HRMS(ESI): *m/z* calcd for $C_{21}H_{19}N_2O_4$ $(M+H)^+$: 363.1339; found: 363.1340.

^{1}H NMR (400 MHz, $CDCl_3$) of compound **5e**

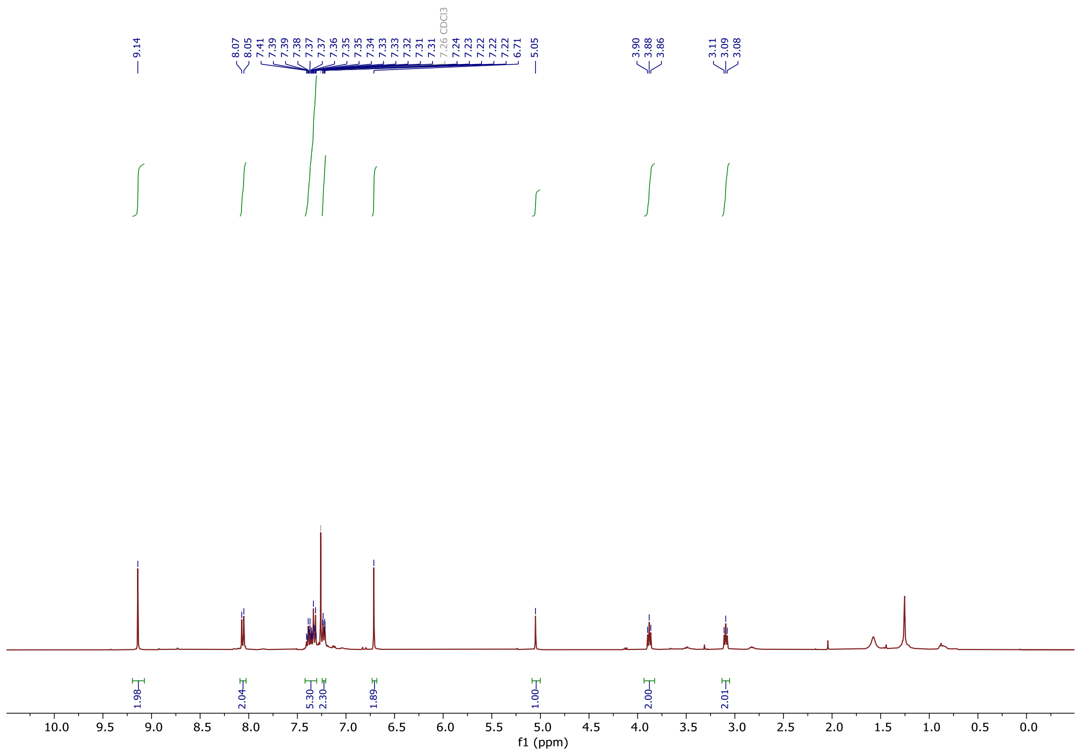

^{13}C NMR (101 MHz, CDCl$_3$) of compound **5e**

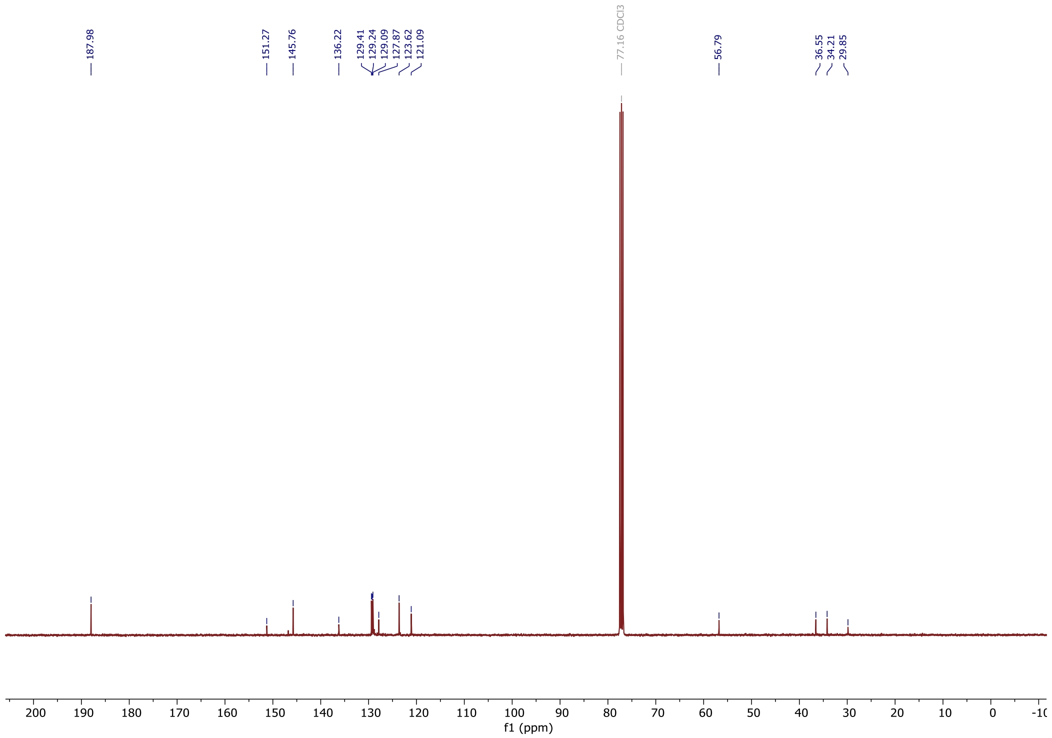

4-(4-methoxyphenyl)-1-phenethyl-1,4-dihydropyridine-3,5-dicarbaldehyde (5f): Synthesized using general procedure I, by using 4-methoxybenzaldehyde. The compound **5f** is purified by column chromatography as an off-white solid (15% yield).

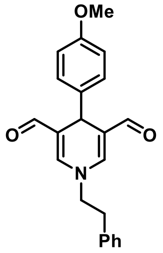

^{1}H NMR (400 MHz, CDCl$_3$): δ 9.15 (s, 2H), 7.40 – 7.29 (m, 3H), 7.25 – 7.18 (m, 2H), 7.13 – 7.07 (m, 2H), 6.79 – 6.72 (m, 2H), 6.64 (s, 2H), 4.91 (s, 1H), 3.81 (t, J = 6.8 Hz, 2H), 3.74 (s, 3H), 3.06 (t, J = 6.8Hz, 2H). **^{13}C NMR (101 MHz, CDCl$_3$):** δ 188.5, 158.4, 145.1, 137.2, 136.5, 129.3, 129.2, 129.1, 127.6, 122.4, 113.7, 56.8, 55.3, 36.7, 32.9.

HRMS(ESI): *m/z* calcd for $C_{22}H_{22}NO_3$ $(M+H)^+$: 348.1594; found: 348.1592.

^{1}H NMR (400 MHz, CDCl₃) of compound **5f**

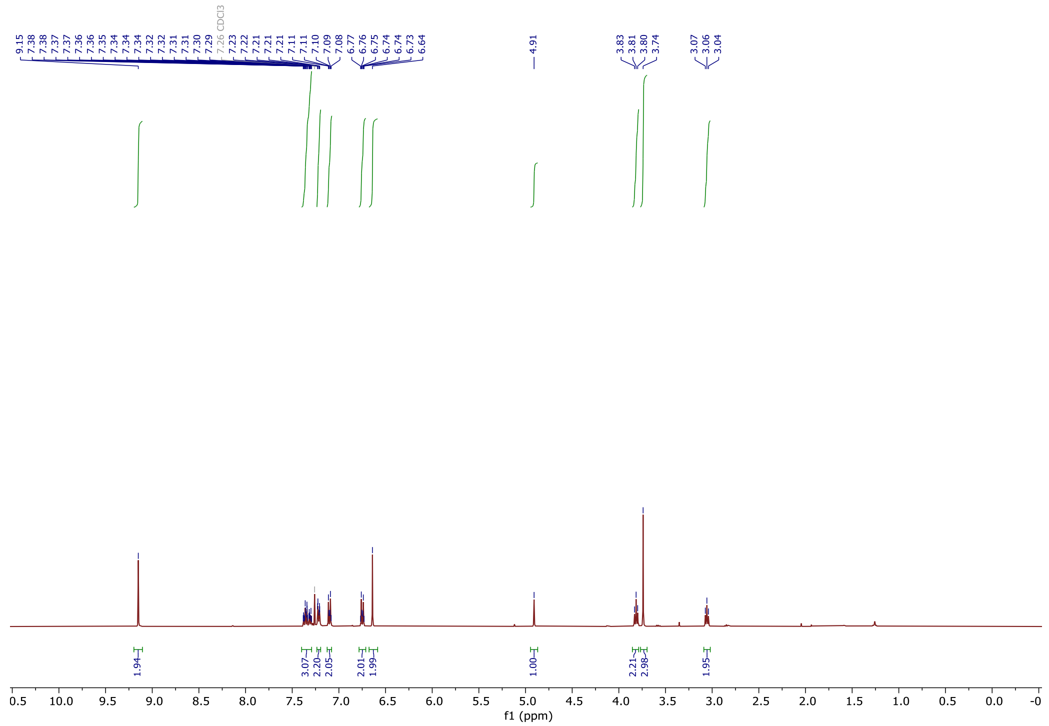

^{13}C NMR (101 MHz, CDCl₃) of compound **5f**

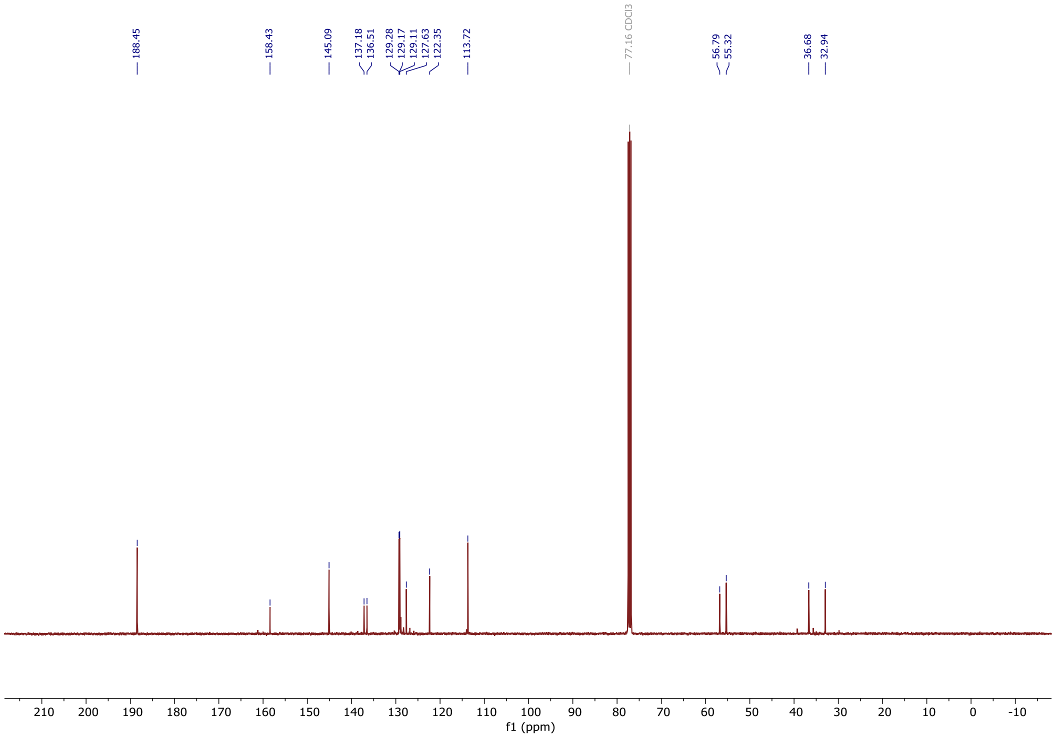

(E)-4-methyl-1-phenethyl-5-styryl-1,4-dihydropyridine-3-carbaldehyde (5g): Synthesized using Wittig reaction protocol on purified **5b**.

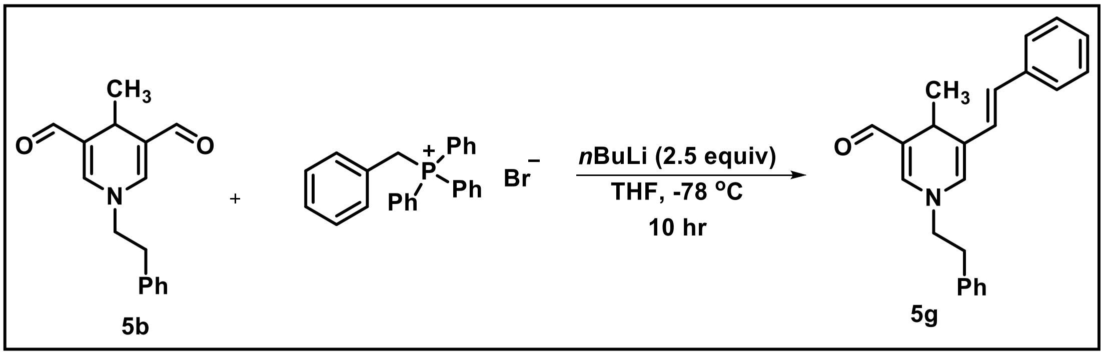

To a 100 mL two-neck round bottom flask equipped with a stirring bar and a rubber septum was charged benzyltriphenylphosphonium bromide (1 g, 2.3 mmol, 3 equiv.) and left to dry under high vacuum for 1-2 hours. The flask was cooled to -78 °C under inert atmosphere. The Wittig salt was dissolved in 2.3 mL THF followed by dropwise addition of BuLi (2.5 equiv) for around 30 minutes. The reaction mixture is allowed to stir for 30 minutes at -78 °C. Next, a solution of **5b** (200 mg, 1equiv) in THF is added dropwise to reaction mixture. After completing the addition of **5b**, the reaction mixture slowly warmed to room temperature and stirred for 10 hours. The reaction was quenched by saturated ammonium hydroxide solution and extracted with ethyl acetate. The organic layers were combined, dried over $MgSO_4$, filtered, and concentrated under reduced pressure. Compound **5g** was obtained by column chromatography as yellow solid (22%).

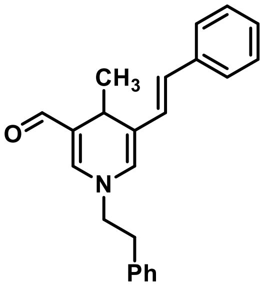

^{1}H NMR (600 MHz, $CDCl_3$): δ 9.04 (s, 1H), 7.43 – 7.36 (m, 2H), 7.31 (q, J = 7.2 Hz, 5H), 7.24 – 7.16 (m, 3H), 6.69 – 6.59 (m, 2H), 6.42 (s, 1H), 6.00 (s, 1H), 4.00 (q, J = 6.6 Hz, 1H), 3.66 – 3.54 (m, 2H), 2.95 (t, J = 6.8 Hz, 2H), 1.15 (d, J = 6.6 Hz, 3H). **^{13}C NMR (151 MHz, $CDCl_3$):** δ 188.6, 147.8, 137.8, 137.3, 129.1, 129.0, 128.8, 127.9, 127.3, 127.1, 126.5, 126.1, 125.3, 123.1, 118.1, 56.7, 36.7, 25.1, 21.8.

HRMS(ESI): *m/z* calcd for $C_{23}H_{24}NO$ $(M+H)^+$: 330.1852; found: 330.1849.

^{1}H NMR (600 MHz, CDCl$_3$) of compound **5g**

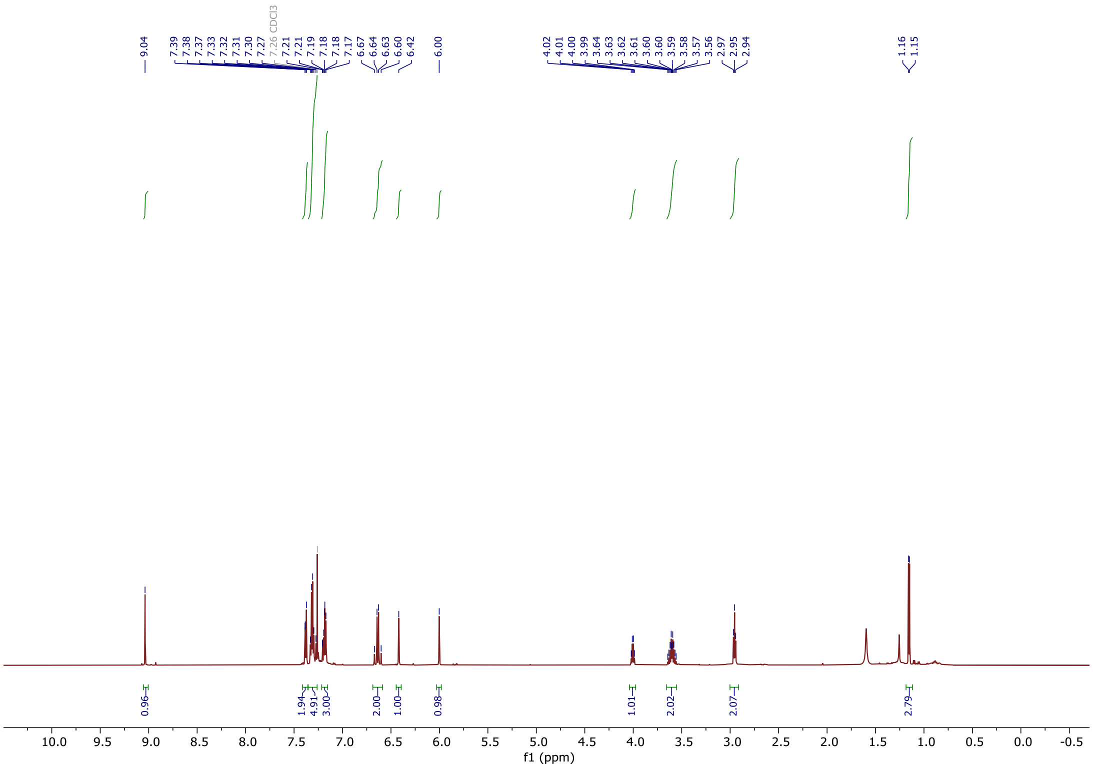

^{13}C NMR (151 MHz, CDCl$_3$) of compound **5g**

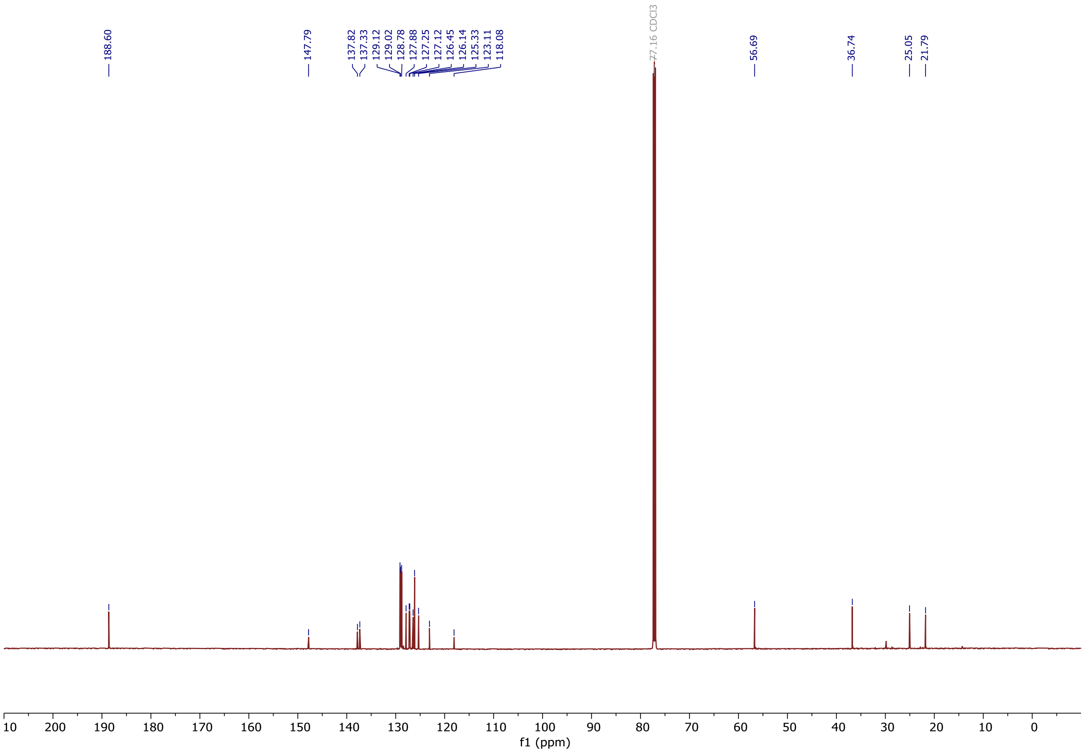

General procedure II for the synthesis of 5h and 5i: Compounds **5h** and **5i** are synthesized by using wittig protocol (used for compound **5g**) using cinnamyltriphenylphosphonium bromide salt. Both compounds, **5h** and **5i** are isolated from the same reaction.

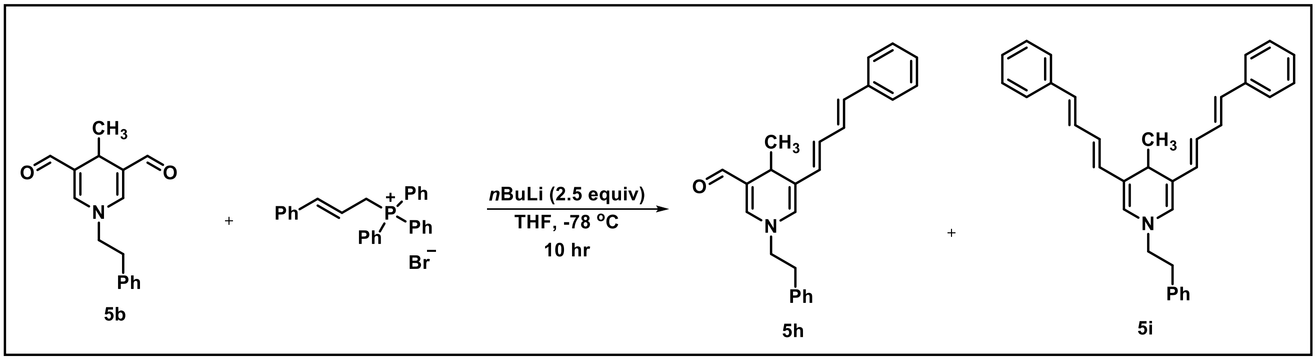

4-methyl-1-phenethyl-5-((1E,3E)-4-phenylbuta-1,3-dien-1-yl)-1,4-dihydropyridine-3-carbaldehyde (5h): Yellow liquid (18% yield).

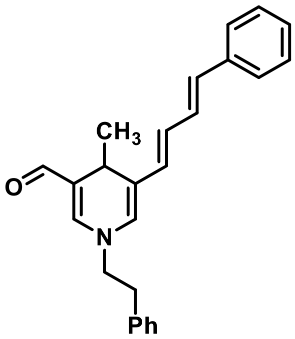

^{1}H NMR (400 MHz, CDCl$_3$): δ 9.03 (s, 1H), 7.43 – 7.38 (m, 2H), 7.37 – 7.25 (m, 5H), 7.23 – 7.15 (m, 3H), 6.82 (dd, *J* = 15.8, 10.4 Hz, 1H), 6.52 (dd, *J* = 15.4, 9.9 Hz, 2H), 6.40 (d, *J* = 1.4 Hz, 1H), 6.18 (d, *J* = 15.4 Hz, 2H), 5.92 (s, 1H), 3.91 (q, *J* = 6.6 Hz, 1H), 3.59 (td, *J* = 6.9, 4.8 Hz, 2H), 2.94 (t, *J* = 6.8 Hz, 2H), 1.13 (d, *J* = 6.6 Hz, 3H). **^{13}C NMR (151 MHz, CDCl$_3$):** δ 188.5, 147.6, 137.8, 137.3, 132.3, 131.4, 131.0, 129.6, 129.2, 129.1, 129.1, 129.0, 128.9, 128.8, 128.8, 128.8, 127.6, 127.3, 127.3, 126.6, 126.6, 126.5, 126.4, 126.4, 123.3, 118.2, 56.7, 36.8, 29.9, 25.1, 21.9.

HRMS(ESI): *m/z* calcd for $C_{25}H_{26}NO$ $(M+H)^+$: 356.2009; found: 356.2009.

^{1}H NMR (400 MHz, $CDCl_3$) of compound **5h**

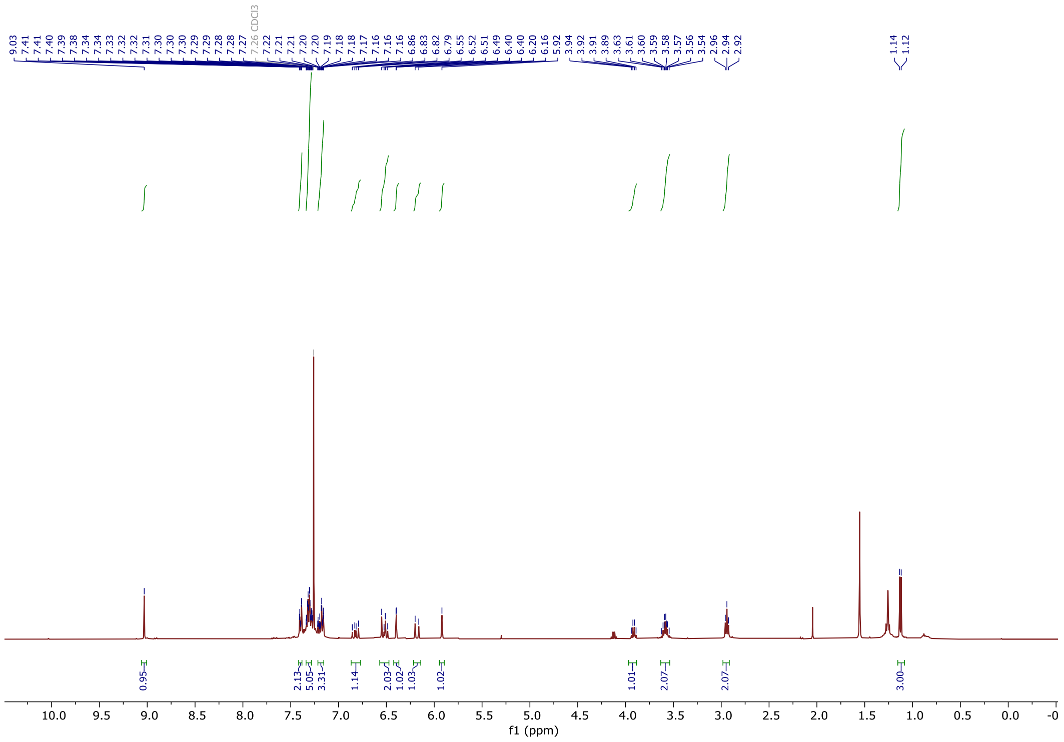

^{13}C NMR (151 MHz, $CDCl_3$) of compound **5h**

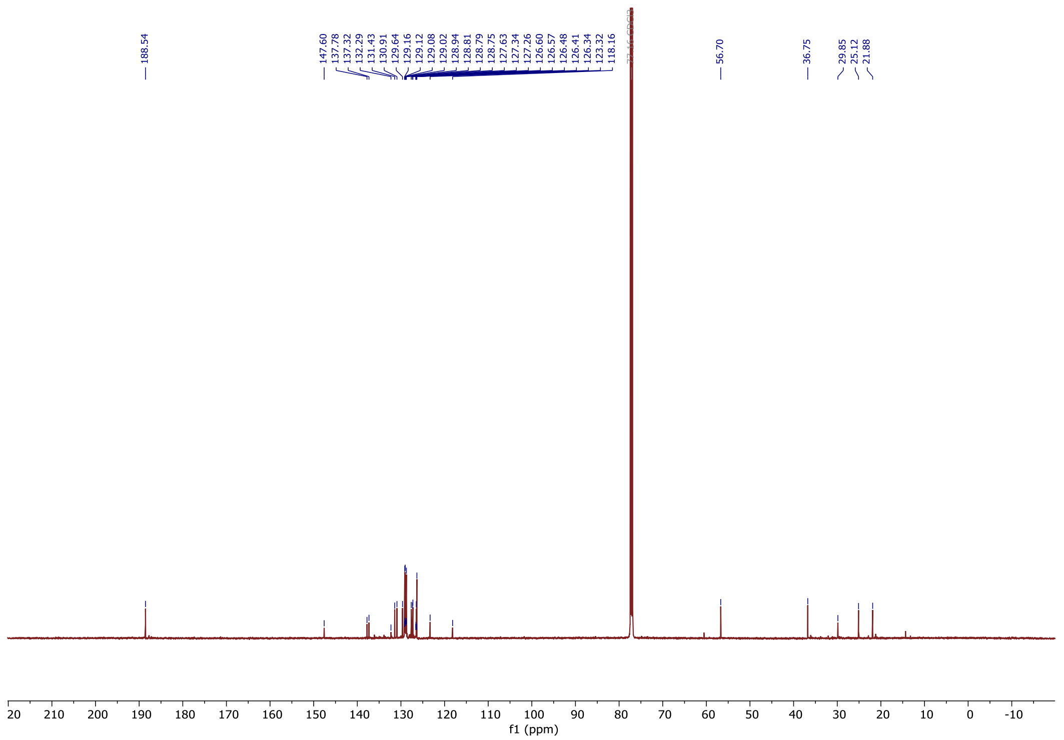

4-methyl-1-phenethyl-3,5-bis((1E,3E)-4-phenylbuta-1,3-dien-1-yl)-1,4-dihydropyridine (5i): Light-yellow liquid (24% yield).

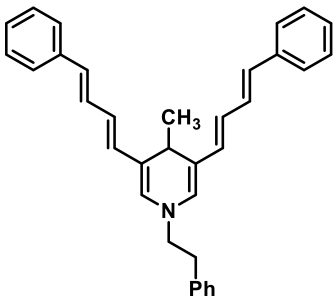

^{1}H NMR (400 MHz, $CDCl_3$): δ 7.45 – 7.30 (m, 4H), 7.30 (m, 7H), 7.21 – 7.13 (m, 4H), 6.87 (dd, J = 15.5, 10.4 Hz, 2H), 6.52 (d, J = 15.5 Hz, 2H), 6.37 (dd, J = 15.2, 10.4 Hz, 2H), 6.24 (d, J = 15.2 Hz, 2H), 5.94 (s, 2H), 3.82 (q, J = 6.4 Hz, 1H), 3.46 (t, J = 7.2 Hz, 2H), 2.88 (t, J = 7.2 Hz, 2H), 1.19 (d, J = 6.4 Hz, 3H). **^{13}C NMR (151 MHz, $CDCl_3$):** δ 138.3, 138.2, 133.1, 130.6, 130.3, 129.1, 128.8, 128.8, 128.7, 128.7, 126.9, 126.8, 126.0, 122.4, 116.3, 56.2, 37.2, 29.9, 27.5, 20.7, 14.3.

HRMS(ESI): m/z calcd for C34H34N$(M+H)^{+}$: 456.2691; found: 456.2595.

^{1}H NMR (400 MHz, $CDCl_3$) of compound **5i**

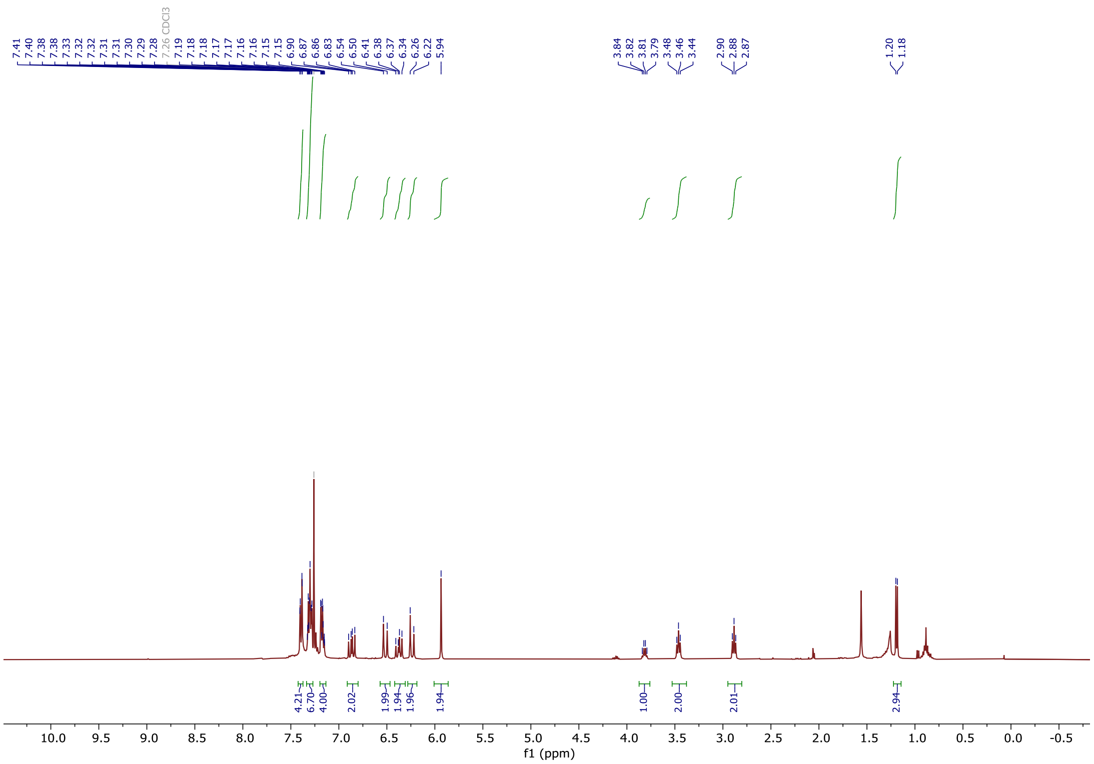

^{13}C NMR (151 MHz, $CDCl_3$) of compound **5i**

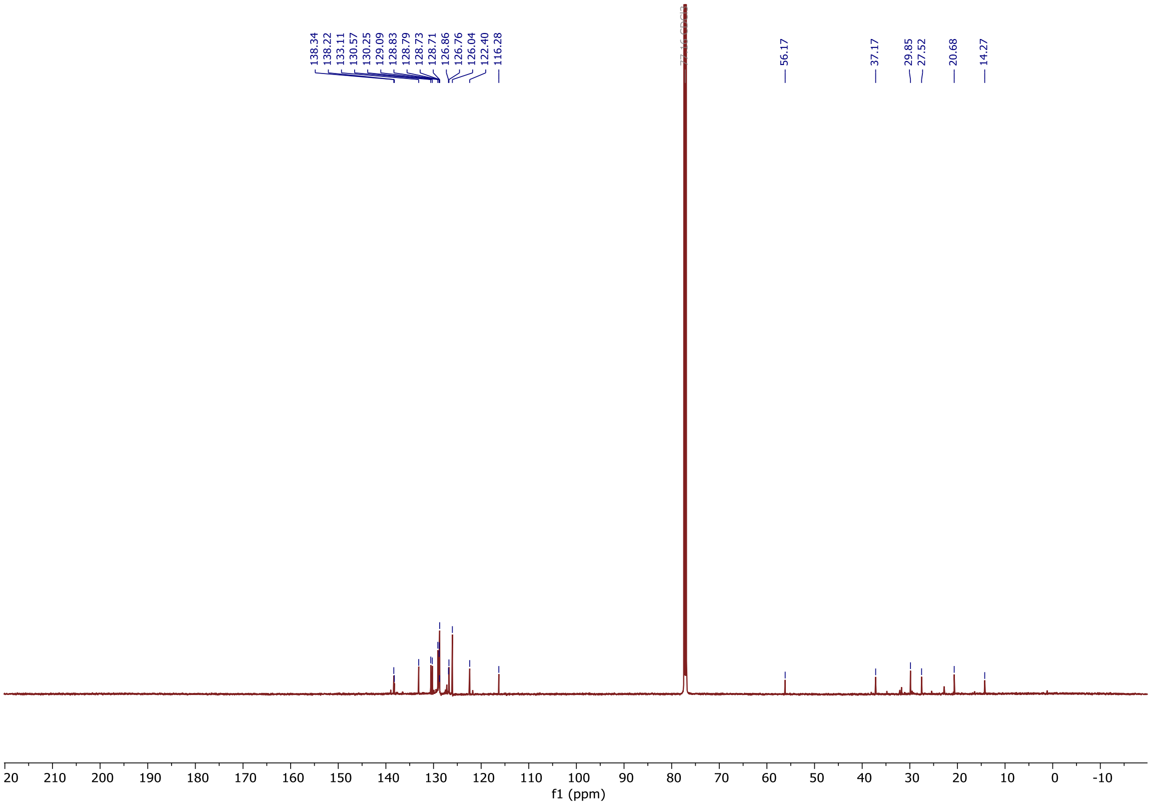

Procedure for the synthesis of compound 5j: Synthesized using Knoevenagel condensation protocol.

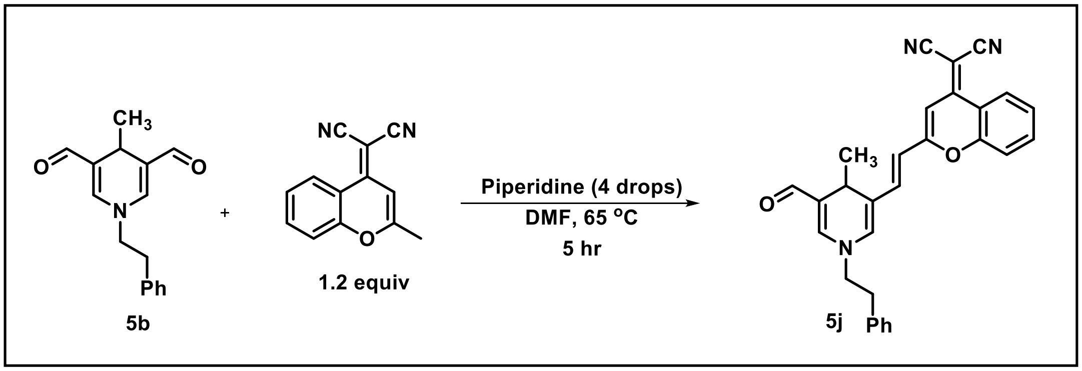

To a 25 mL round-bottom flask charged with **5b** (30 mg, 1 equiv.) and 2-(2-Methyl-4h-chromen-4-ylidene)malononitrile (32 mg, 1.2 equiv.). DMF (3 ml) is added under inert conditions. To the reaction mixture, 4 drops of piperidine were added. Followed by heating the reaction mixture at 65 °C for 5 hours. The reaction was cooled to room temperature, diluted in ice-cold brine and extracted with ethyl acetate (3 x 30 mL). The organic layers were combined, dried with $MgSO_4$, filtered, and concentrated under vacuum. The residue was purified via column chromatography using hexane/ethyl acetate as eluent as a red color solid (30% yield).

(E)-2-(2-(2-(5-formyl-4-methyl-1-phenethyl-1,4-dihydropyridin-3-yl)vinyl)-4H-chromen-4-ylidene)malononitrile (5j):

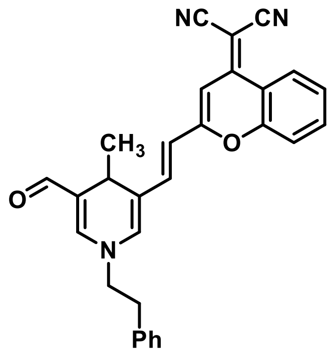

^{1}H NMR (600 MHz, $CDCl_3$): δ 9.11 (s, 1H), 8.89 (dd, *J* = 8.4, 1.4 Hz, 1H), 7.69 (ddd, *J* = 8.5, 7.2, 1.5 Hz, 1H), 7.47 – 7.38 (m, 2H), 7.34 (t, *J* = 7.2 Hz, 2H), 7.31 – 7.26 (m, 1H), 7.20 – 7.16 (m, 2H), 7.12 (d, *J* = 15.6 Hz, 1H), 6.75 (s, 1H), 6.47 (s, 1H), 6.32 – 6.24 (m, 2H), 3.88 (q, *J* = 6.6 Hz, 1H), 3.68 (t, *J* = 6.8 Hz, 2H), 2.99 (t, *J* = 6.8 Hz, 2H), 1.13 (d, *J* = 6.6 Hz, 3H). **^{13}C NMR (151 MHz, $CDCl_3$):** δ 188.5, 158.6, 152.8, 152.5, 146.6, 137.7, 136.8, 135.1, 134.4, 129.2, 129.1, 127.5, 126.0, 125.9, 121.0, 120.3, 118.5, 118.1, 117.4, 116.2, 114.2, 105.3, 61.2, 56.9, 36.8, 29.9, 24.8, 21.6.

HRMS(ESI): *m/z* calcd for C29H23N3O2 $(M+H)^+$: 446.1863; found: 446.1861.

^{1}H NMR (600 MHz, $CDCl_3$) of compound **5j**

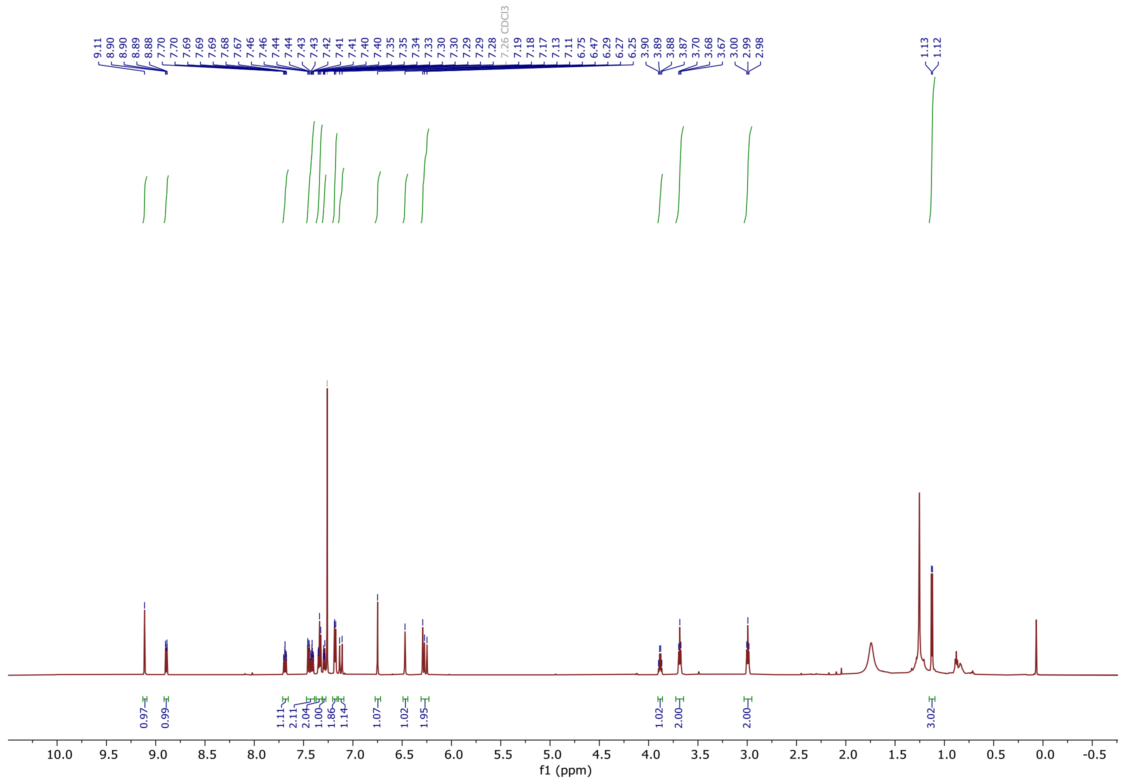

^{13}C NMR (151 MHz, $CDCl_3$) of compound **5j**

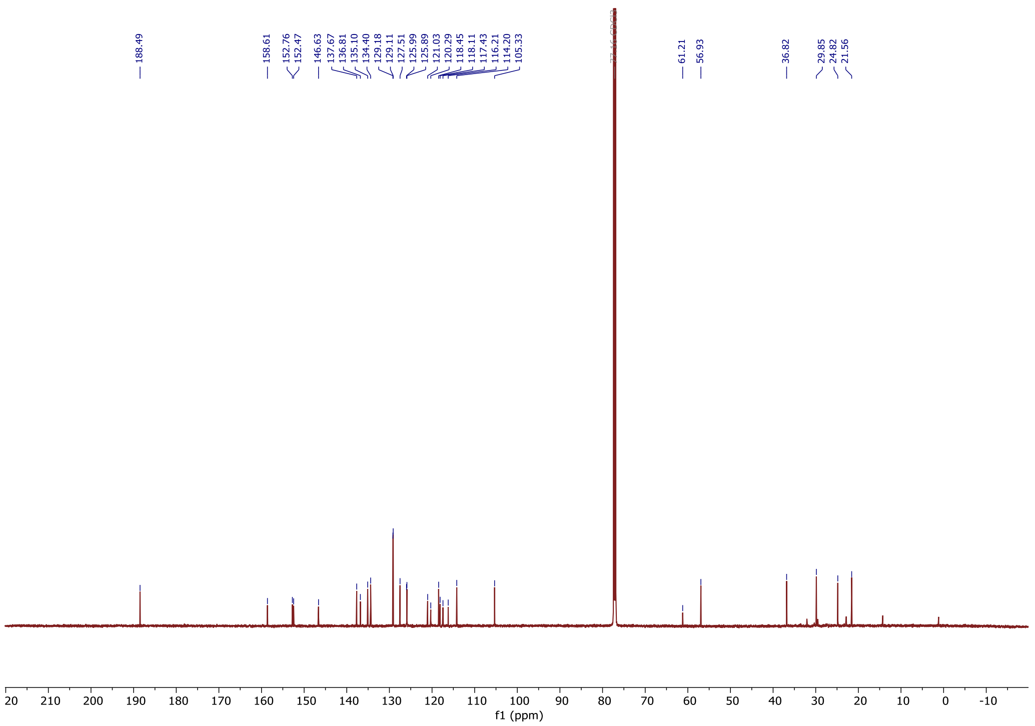

Supplementary Figure 11. Absorbance and Emission Spectra of DHP analogs.

The absorbance and fluorescence of compounds **5b-5j** were measured using acetonitrile as the solvent at 0.04 mM concentration.

For compound **5b:**

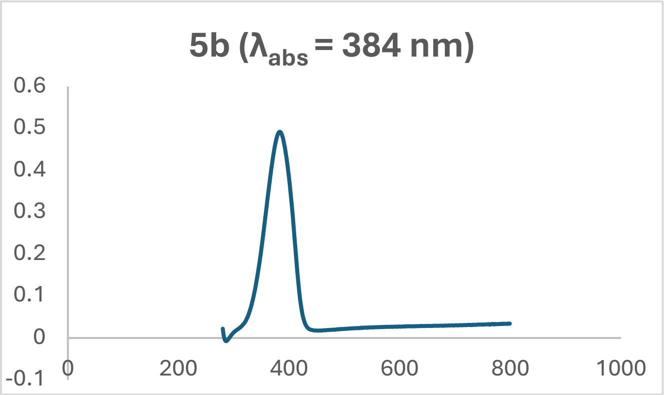

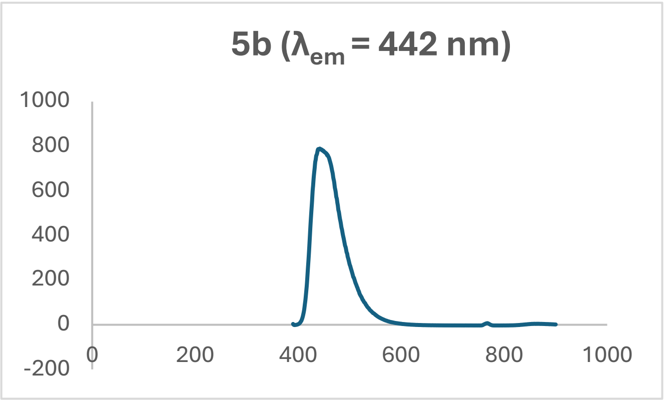

For compound **5c:**

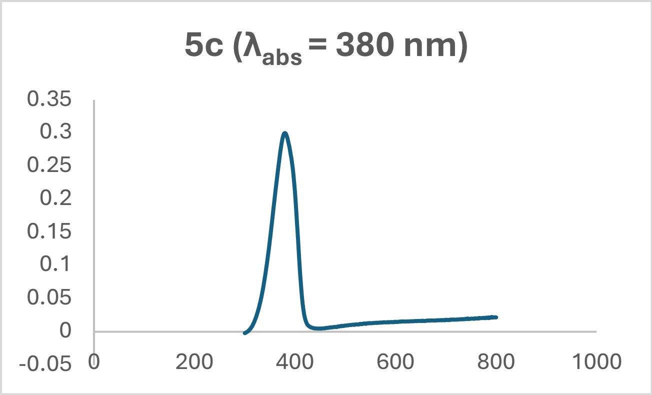

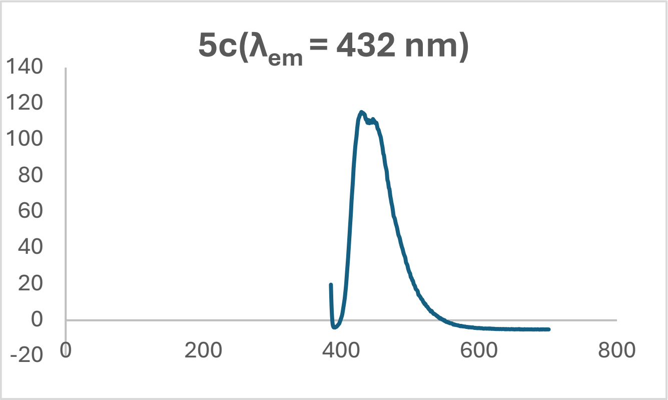

For compound **5d:**

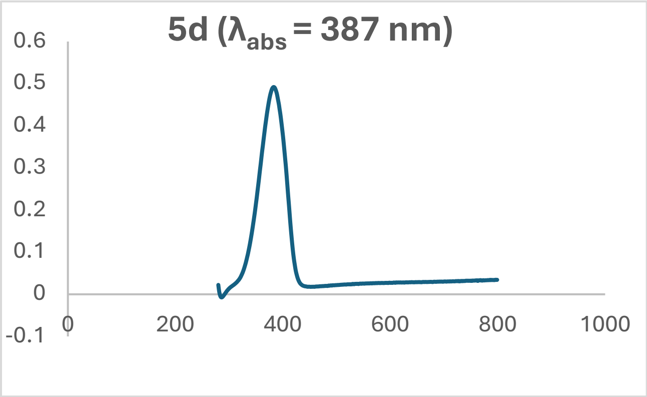

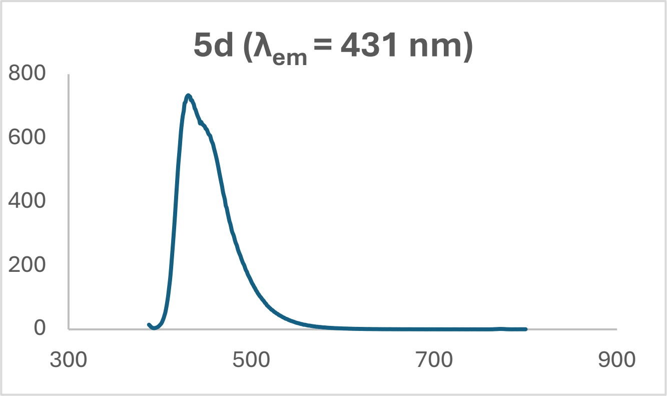

For compound **5e:**

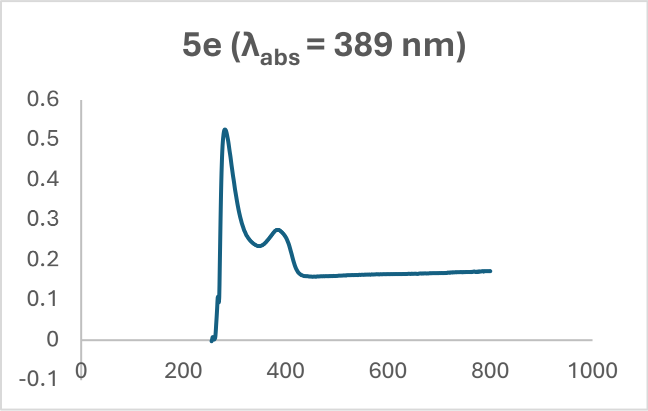

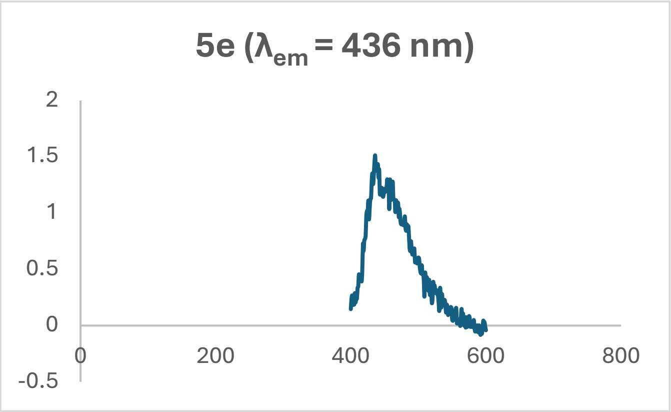

For compound **5f:**

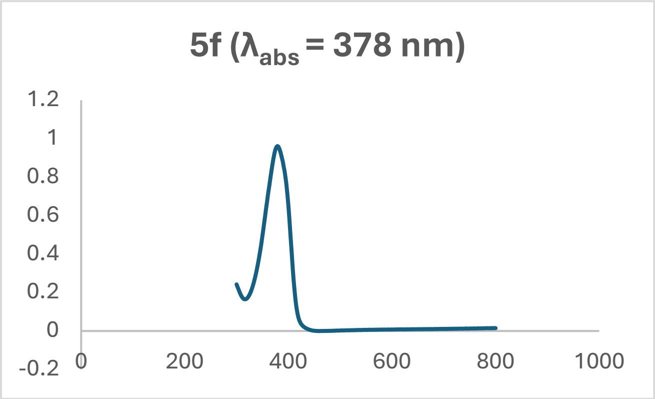

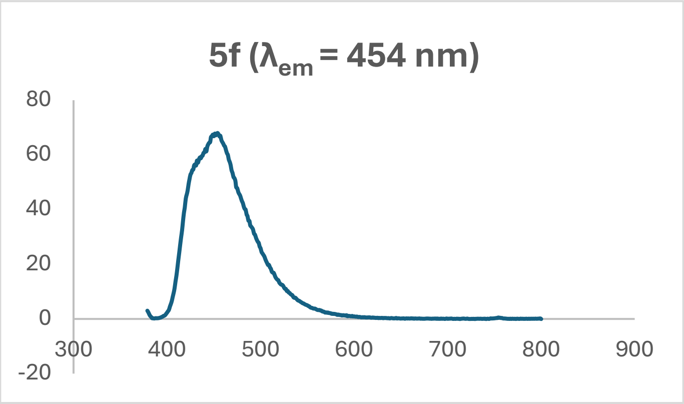

For compound **5g:**

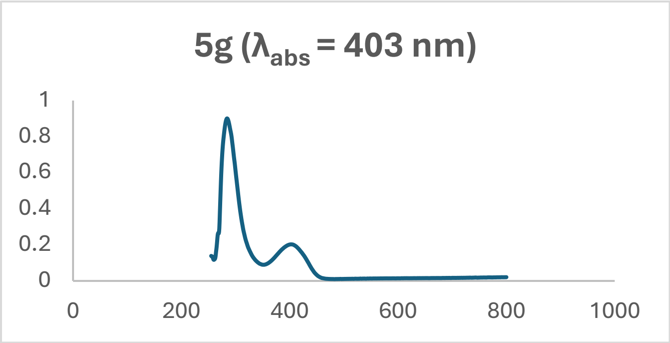

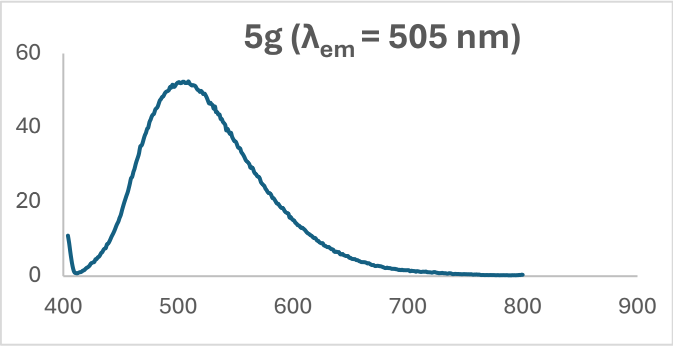

For compound **5h:**

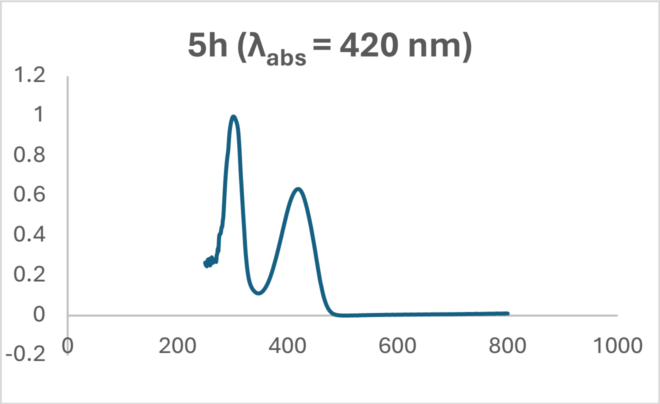

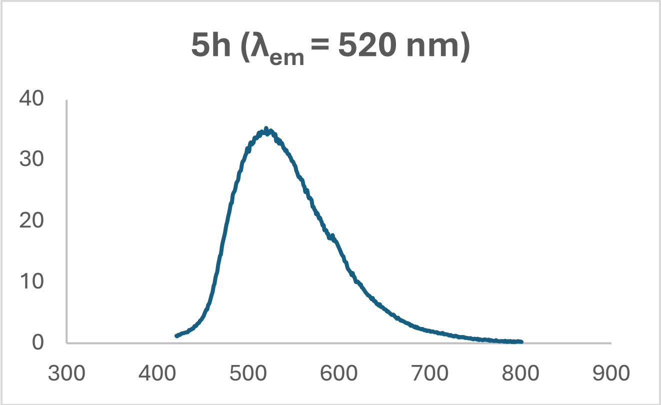

For compound **5i:**

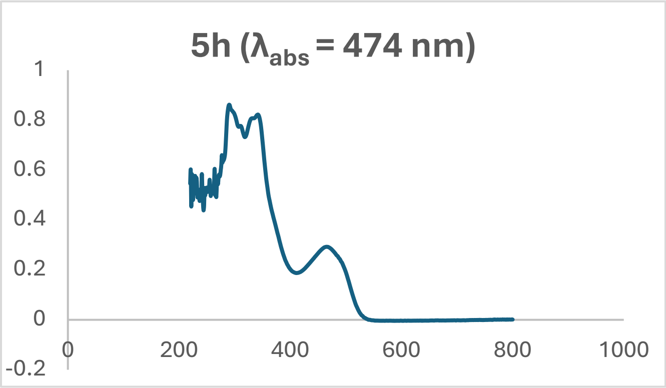

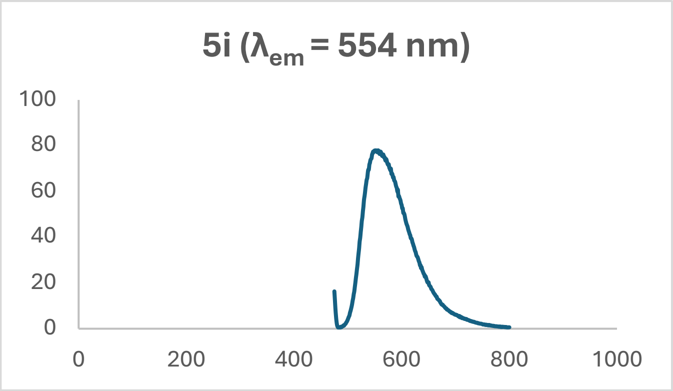

For compound **5j:**

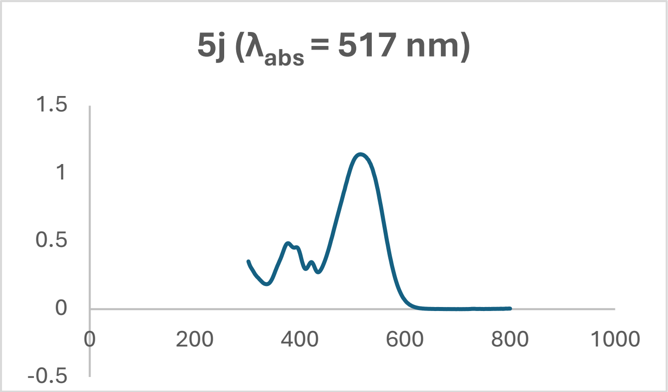

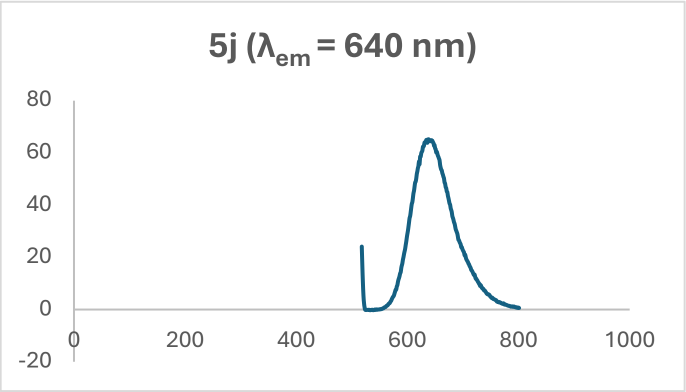

Supplementary Figure 12. Quantum Yield of 5b and 5j

Quantum yield was calculated using the area under the curve of fluorescence versus absorption. Absorption was measured with a Cary 3500 UV-Vis utilizing the samples by using four separate concentrations with values being divided by ten for accurate analysis. Fluorescence area was measured with a Cary Eclipse fluorimeter using the same samples above with a 10x dilution. All measurements were run in triplicate. Quantum yields of **5b** and **5j** were determined using Cy2 as a reference compound. The following equation was used to calculate quantum yield:

$$\phi = \phi_r \times \frac{m}{m_r} \times (\frac{n}{n_r})^2$$

Φ is the quantum yield; m is the slope of the line described above; n is the refractive index of the solvent. Subscript r denotes the appropriate values for the reference (Cy2).

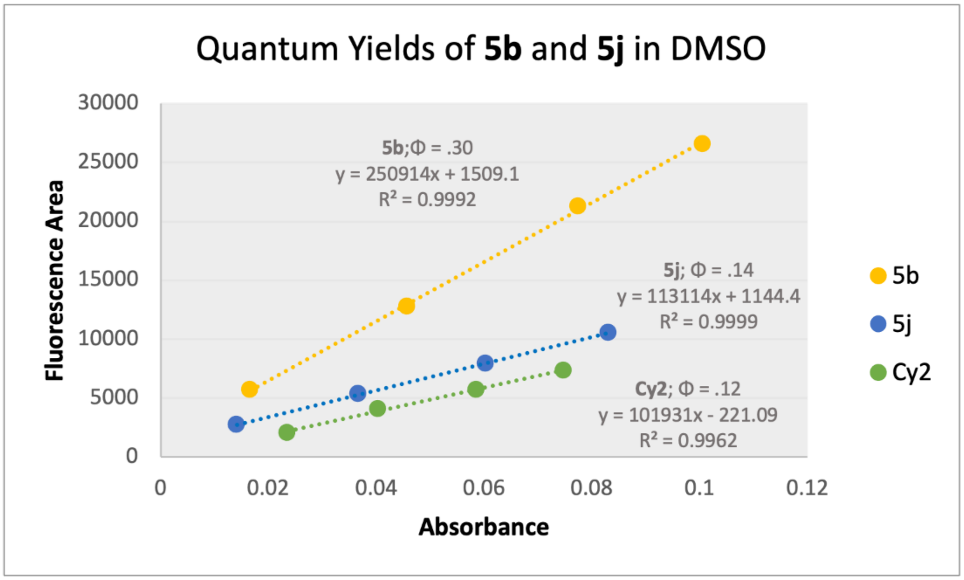

Supplementary Figure 13. Cytotoxicity of 5j.

HeLa cells were grown in 60 x 15 mm Nunclon™ dishes and allowed to adhere overnight in an incubator at 37 °C, 5% CO_2. Stock solutions of **5j** was prepared in stock 10 mM DMSO solutions before being diluted to the final desired concentration of 10 µM in 3 mL of media. Vehicle controls were treated with equivalent dosage of DMSO. Cells were placed in an incubator for 24 hours. Cells were then detached with trypsin and stained using Annexin V/PI following manufacturer's protocol. To avoid fluorescent crosstalk, Annexin V (AV) conjugated to Pacific Blue (PB) was used to determined apoptosis. Propidium Iodide (PI) was used to determine necrosis within the cellular populations. Cells were analyzed via flow cytometry on a BD FACSympony A3 cell analyzer within 1 hour to quantify cell death. A V470/15 laser was used to detect AV-PB while a B710/50 laser was used to detect PI. FlowJo software (v. 10.10.0) was used to analyze the cytometry data.

Data is an average of 3 replicates analyzed on separate days using different passage number of cells. Two-sided Student's t-Test was used to determine statistical significance. **Note**: Gating is based on the AV/PI controls; samples were analyzed respective to the controls on the day of analysis.

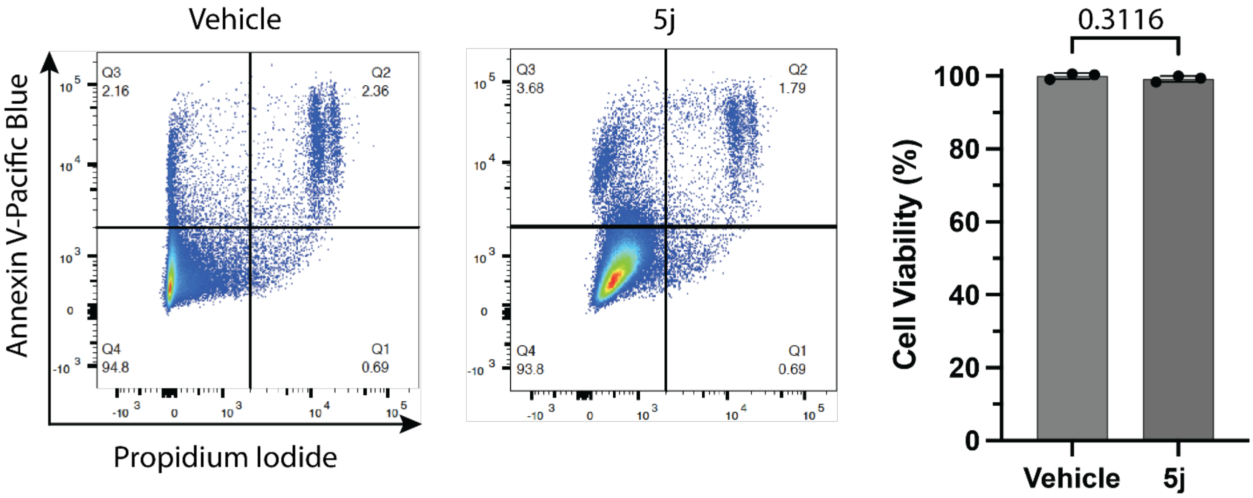

Supplementary Figure 14. Cellular Imaging of 5j in HeLa cells.

Live HeLa cells were plated in an IBIDI 8-well glass bottom chamber at a density of 25,000 cells per well in media and allowed to adhere overnight at 37 °C, 5% CO2. Cells were treated with 10 µM of **5j** and DMSO in 200 µL of media and incubated for 1 h before media was removed and cell were washed with 200 µL of PBS for 5 minutes. PBS was removed and Hoescht (1:2000 dilution) was added according to manufacturer protocol, and cells were incubated for 10 minutes before being washed with 200 µL of PBS for 5 minutes (repeated 3 times). Fresh media (200 µL) was added, and cells were imaged on a Stellaris® 8 Leica DMi8 microscope (63x objective) with fast lifetime contrast (FALCON) module. Samples were excited using a 40 MHz pulsed white light laser tuned to 405 and 517 nm with sequential acquisition. Emitted photons were detected using HyD® S and HyD® X (GaAsP hybrid photocathode). Images were processed and analyzed using ImageJ software. Scale bar = 25 µm.

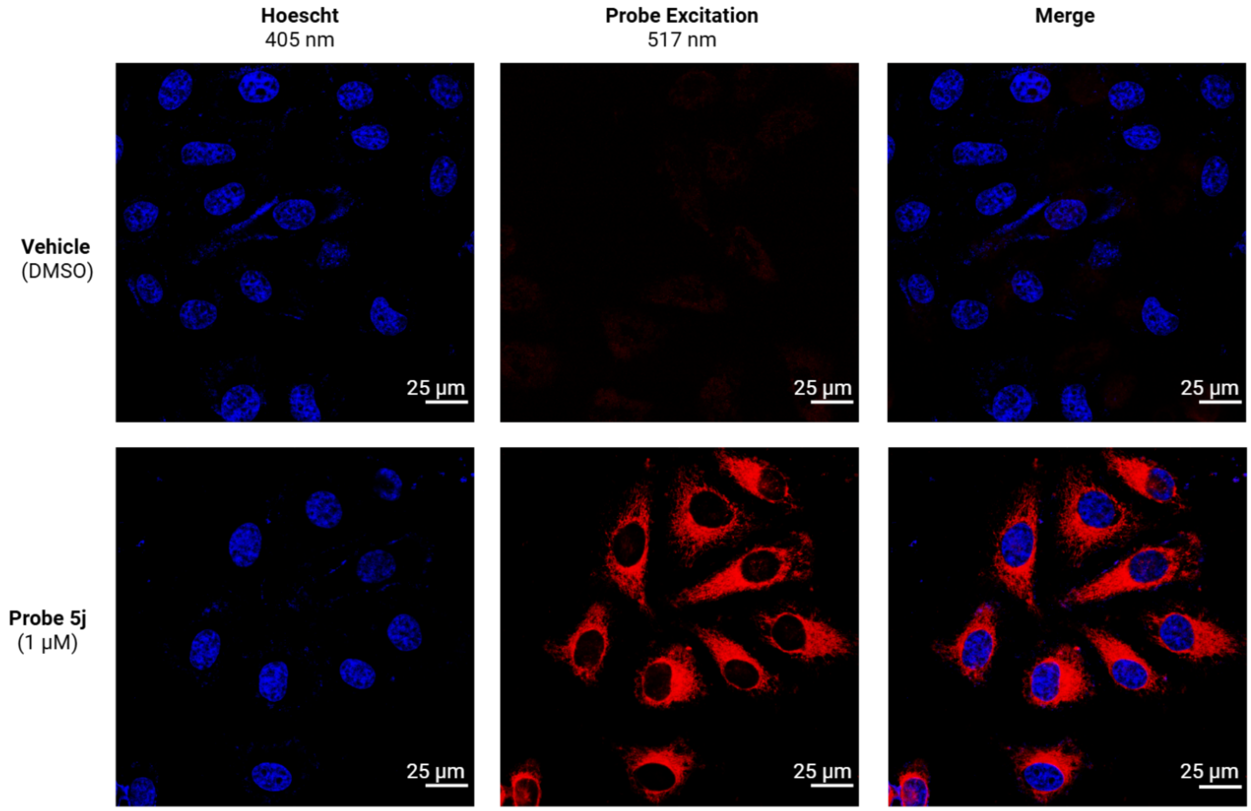

Supplementary Figure 15. Selective Modification of Proteins Reaction Optimization.

Lysozyme Chicken MDA-Benzaldehyde Complex.

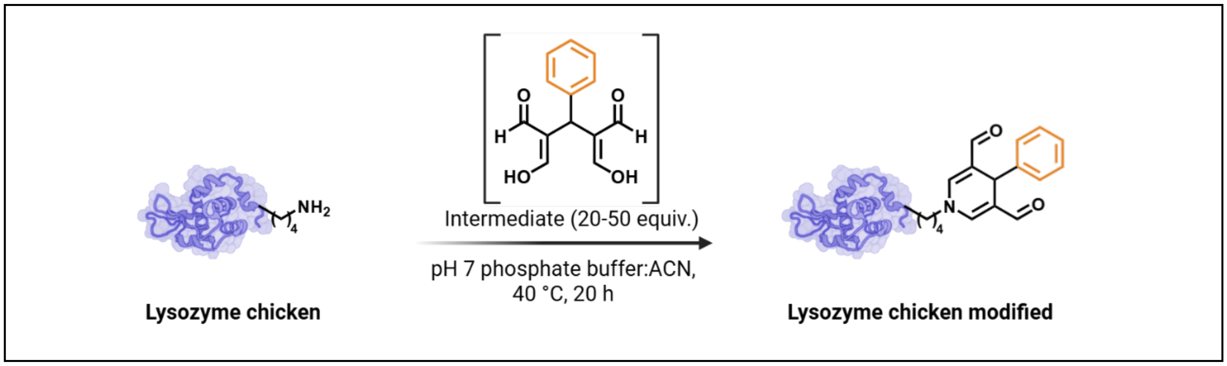

In a one-dram vial, lysozyme chicken (1 mg, 69.9 nmol) was dissolved in 300 µL of sodium phosphate buffer (100 mM, pH 7) and 100 µL of acetonitrile followed by the addition of the intermediate (20-50 equiv.) and left to stir at 37 °C for 20 hours. Subsequently, the reaction mixture was passed through Amicon Ultra 3 kDa spin-concentrator and washed with H_2O (5 x 500 µL) to remove the small molecule impurities. The labeled protein was redissolved in 0.1% formic acid in H_2O and analyzed using LC-MS. See conversion for each attempted condition in the table below. Modification of MDA-benzaldehyde is confirmed by a mass shift of +196 *m/z*. Using 50 equivalents of the intermediate (Entry 3) was found to be the optimized condition for obtaining 6 modifications (>95%).

Optimization table

Entry	Intermediate (equiv.)	% conversion							
		Unmodified	1 Mod	2 Mod	3 Mod	4 Mod	5 Mod	6 Mod	Total
1	20	24%	50%	26%	0%	0%	0%	0%	76%
2	30	13%	37%	35%	15%	0%	0%	0%	87%
3	50	0%	0%	30%	29%	14%	13%	14%	**>95%**

Intact MS Spectrum of Starting Lysozyme Chicken

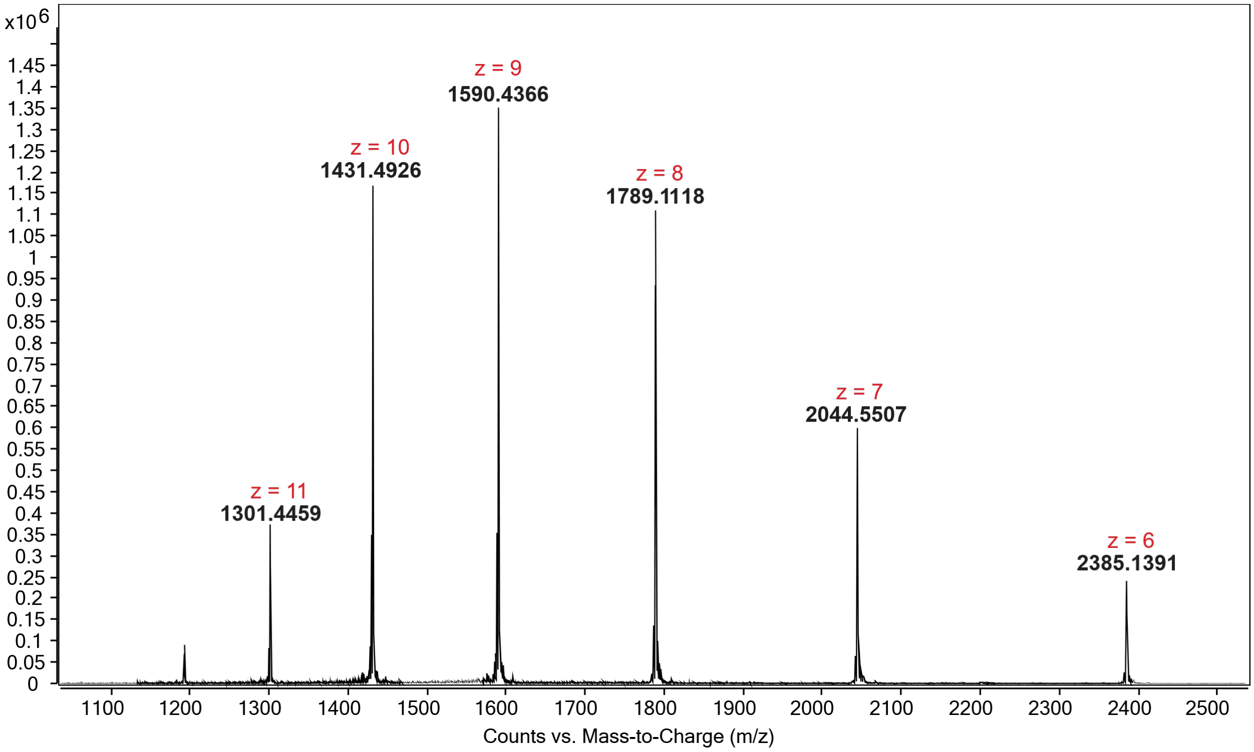

Deconvoluted MS Spectrum of Starting Lysozyme Chicken

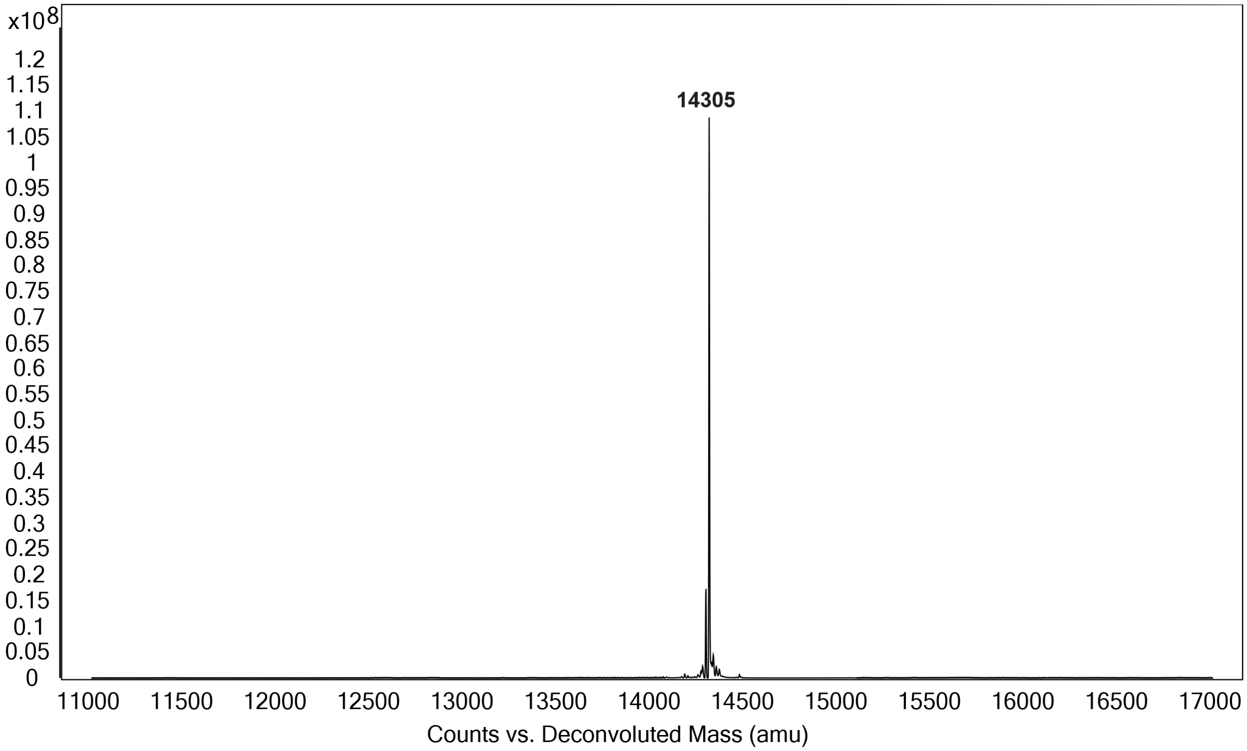

Intact MS Spectrum of Modified Lysozyme Chicken (Entry 1)

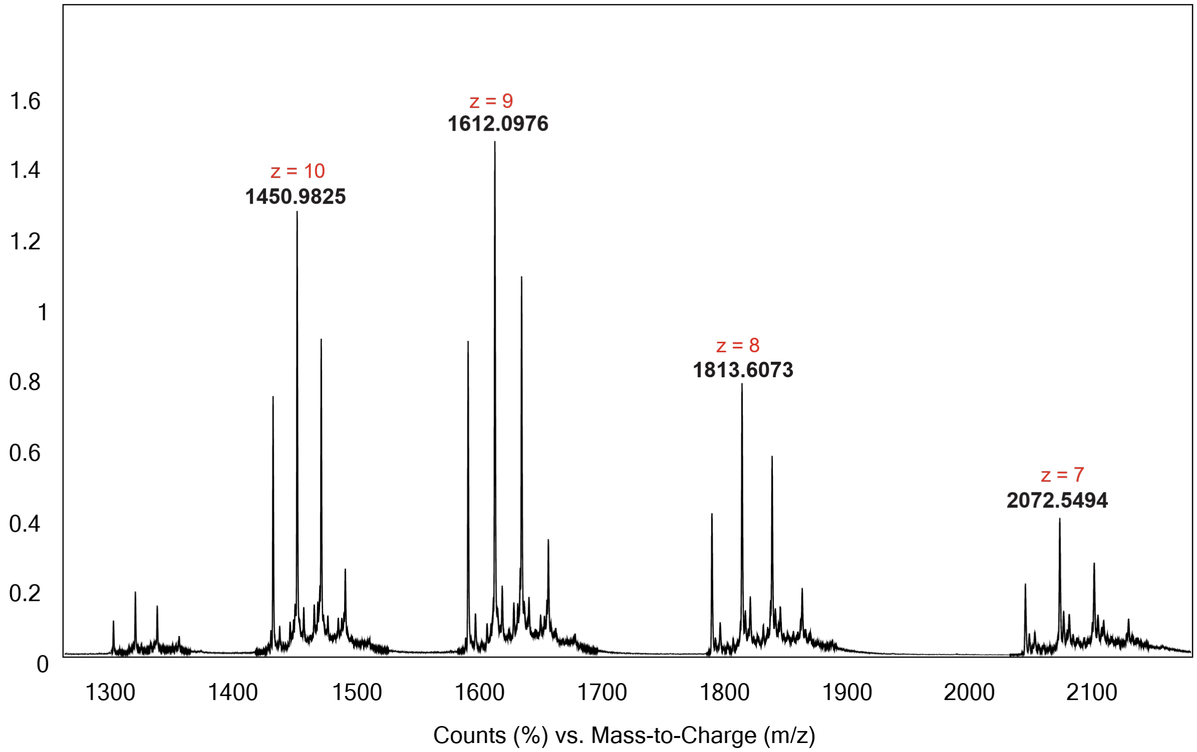

Deconvoluted MS Spectrum of Modified Lysozyme Chicken (Entry 1)

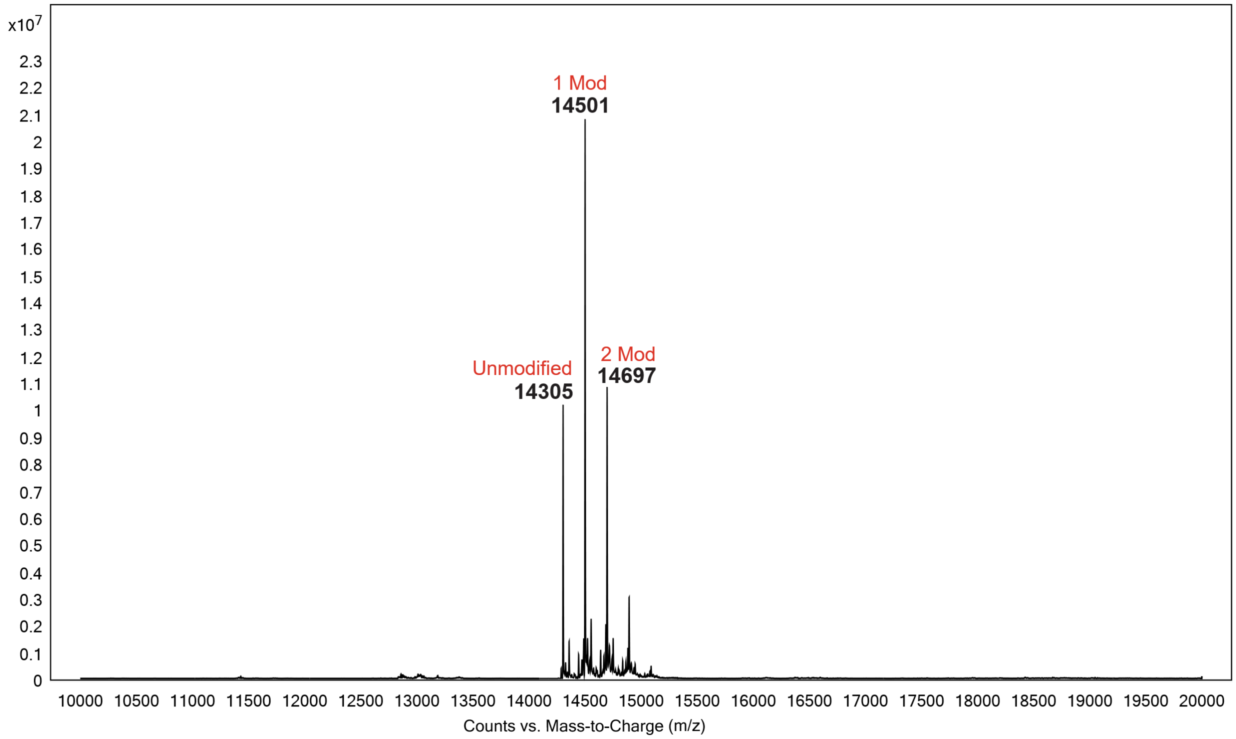

Intact MS Spectrum of Modified Lysozyme Chicken (Entry 2)

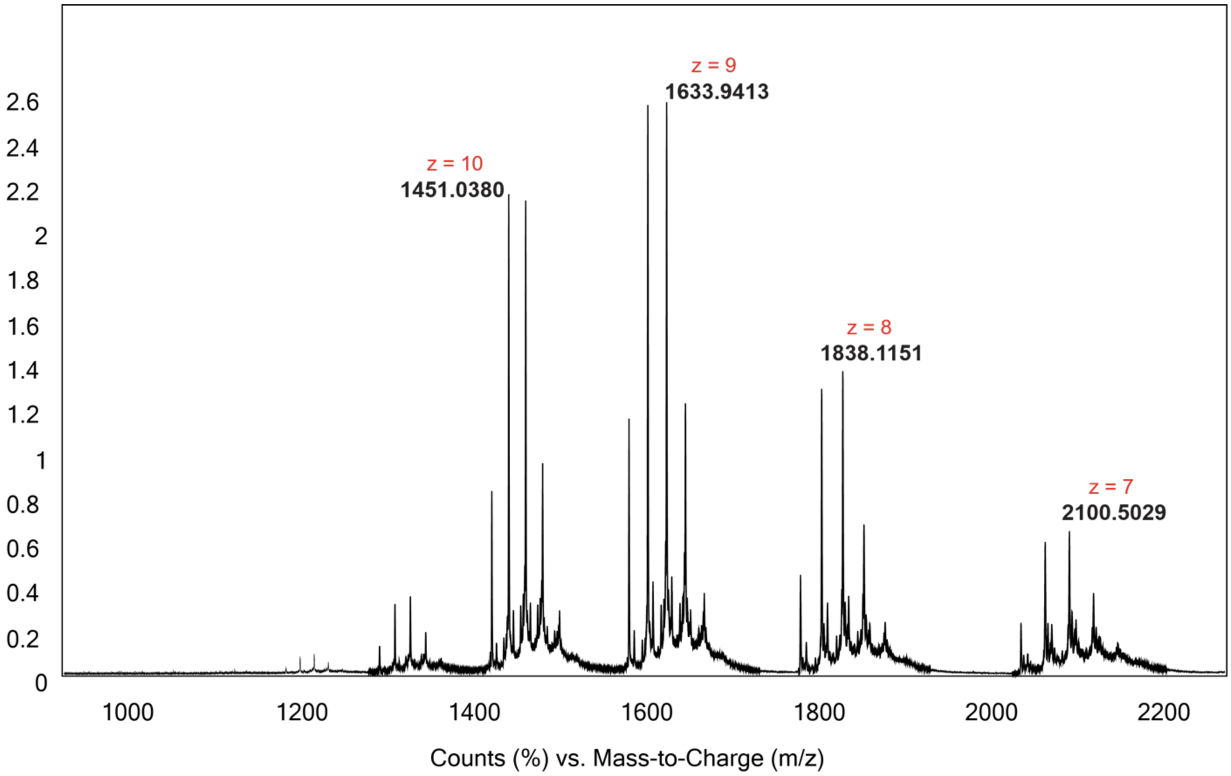

Deconvoluted MS Spectrum of Modified Lysozyme Chicken (Entry 2)

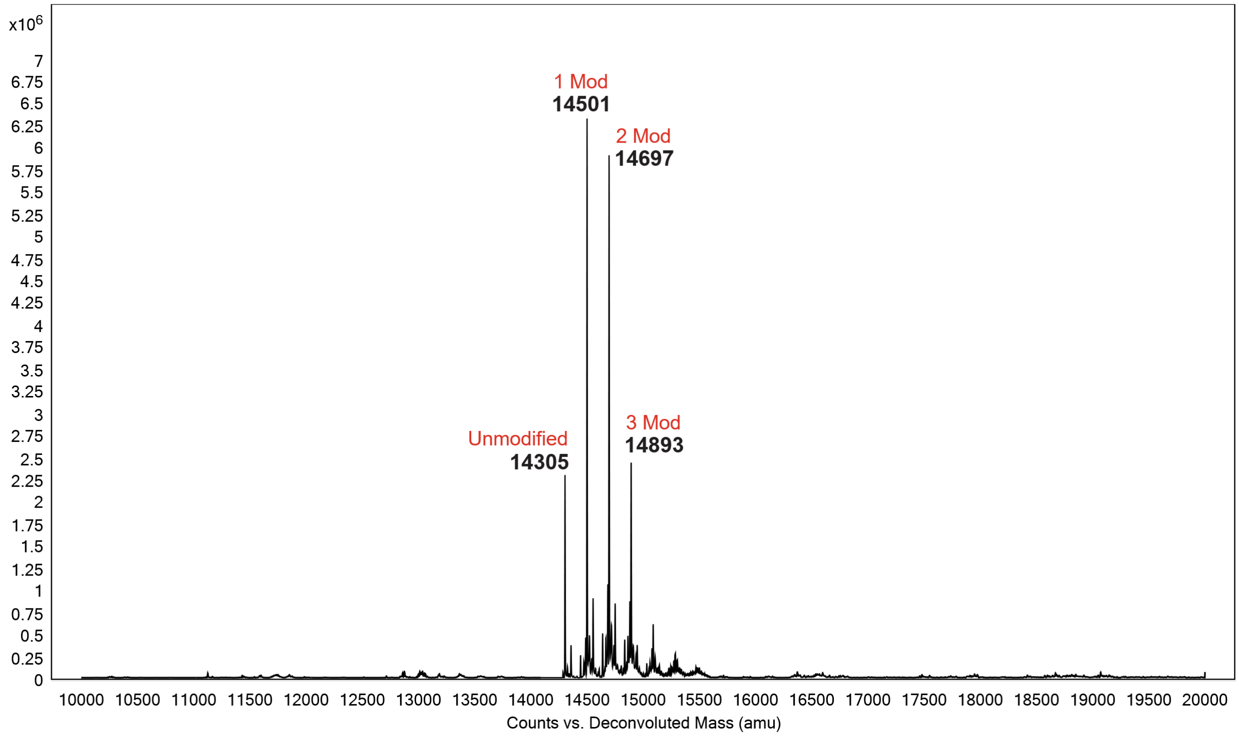

Intact MS Spectrum of Modified Lysozyme Chicken (Entry 3)

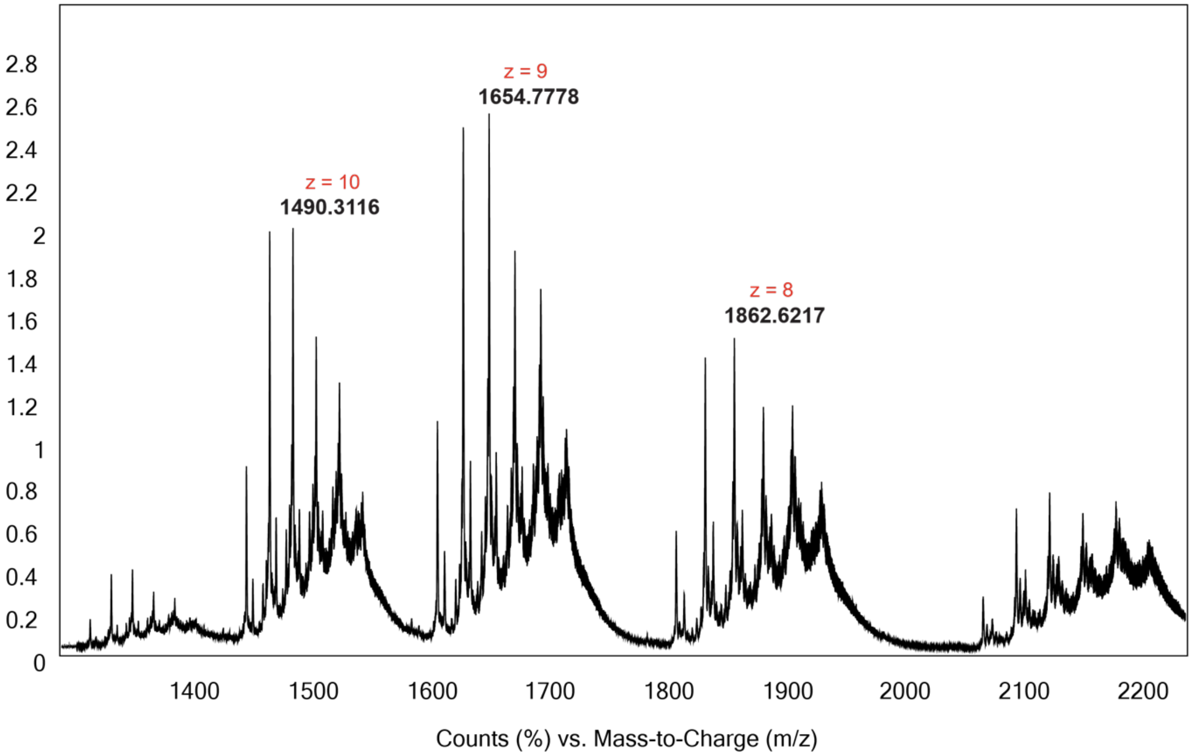

Deconvoluted MS Spectrum of Modified Lysozyme Chicken (Entry 3)

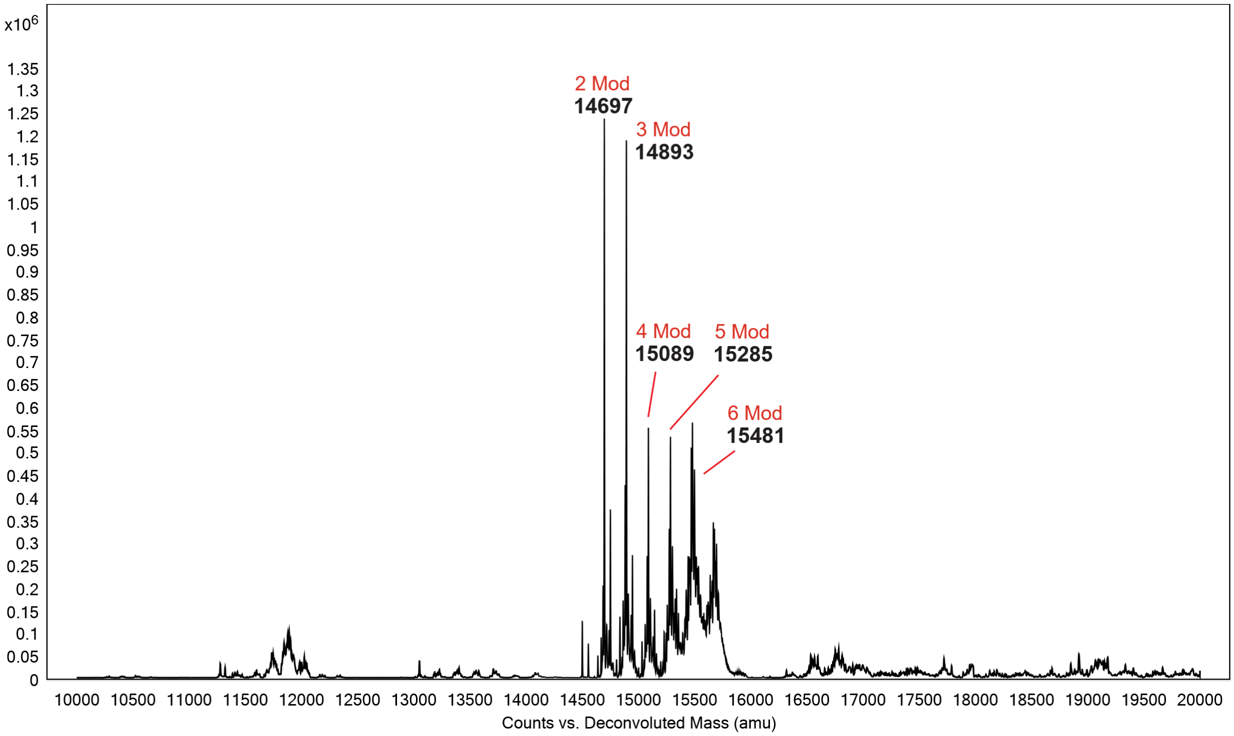

Control Reactions of Intermediate II formation on Lysozyme Chicken.

Malondialdehyde Only.

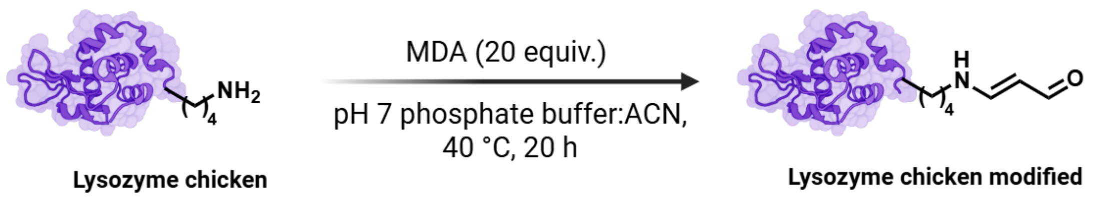

In a one-dram vial, lysozyme chicken (1 mg, 69.9 nmol) was dissolved in 300 µL of sodium phosphate buffer (100 mM, pH 7) and 100 µL of acetonitrile followed by the addition of MDA sodium salt (20 equiv.) and left to stir at 37 °C for 20 hours. Subsequently, the reaction mixture was passed through Amicon Ultra 3 kDa spin-concentrator and washed with H_2O (5 x 500 µL) to remove the small molecule impurities. The labeled protein was redissolved in 0.1% formic acid in H_2O and analyzed using LC-MS. The reaction gave no product.

Intact MS Spectrum of Starting Lysozyme Chicken

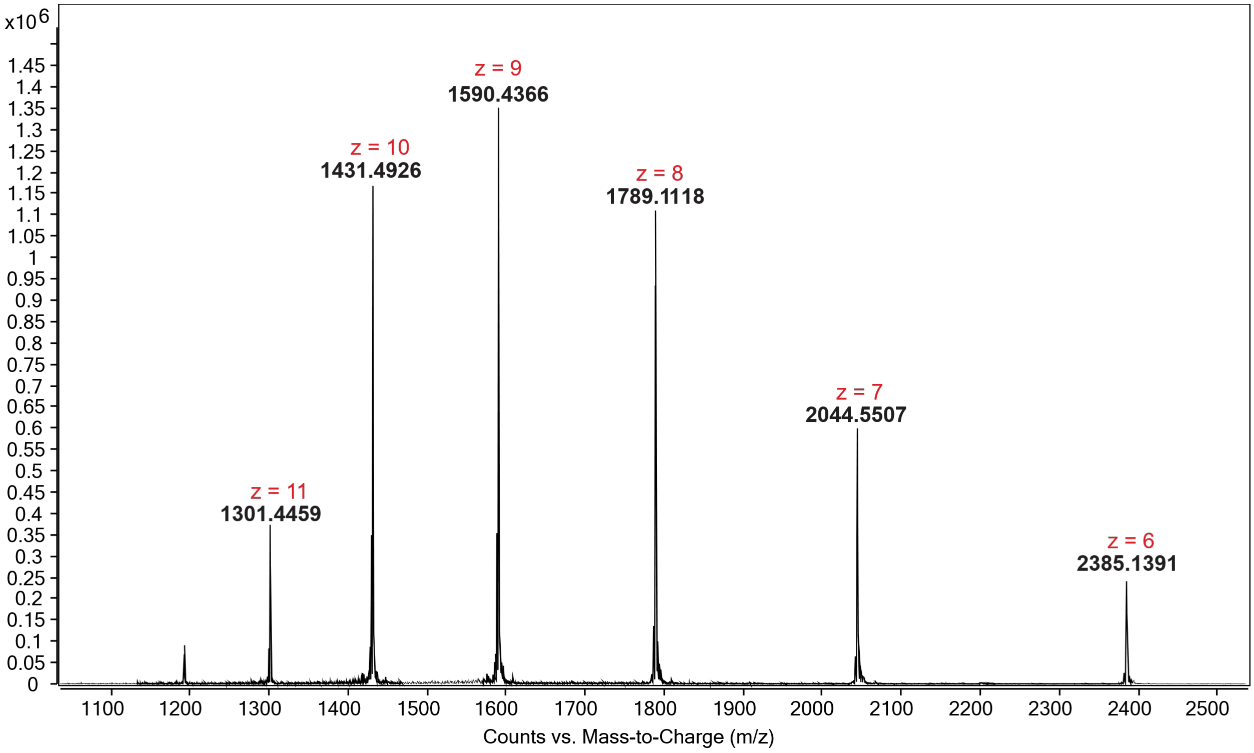

Deconvoluted MS Spectrum of Starting Lysozyme Chicken

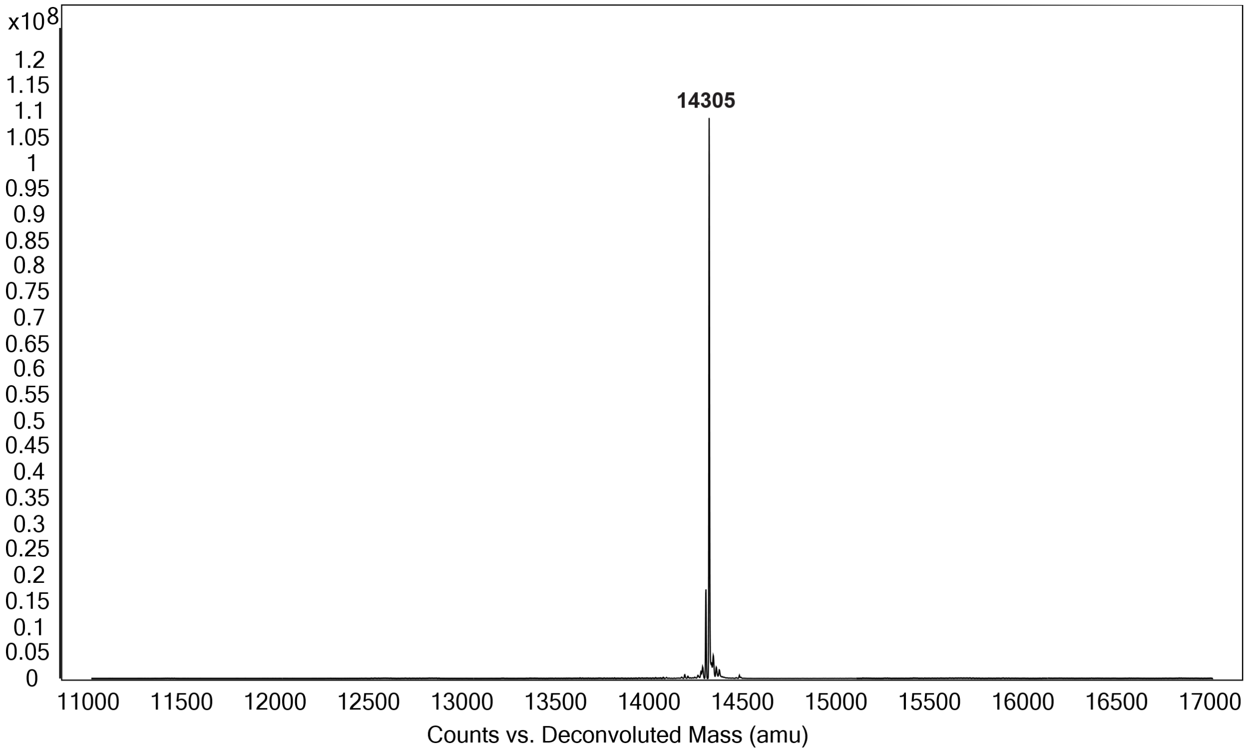

Intact MS Spectrum of Unmodified Lysozyme Chicken

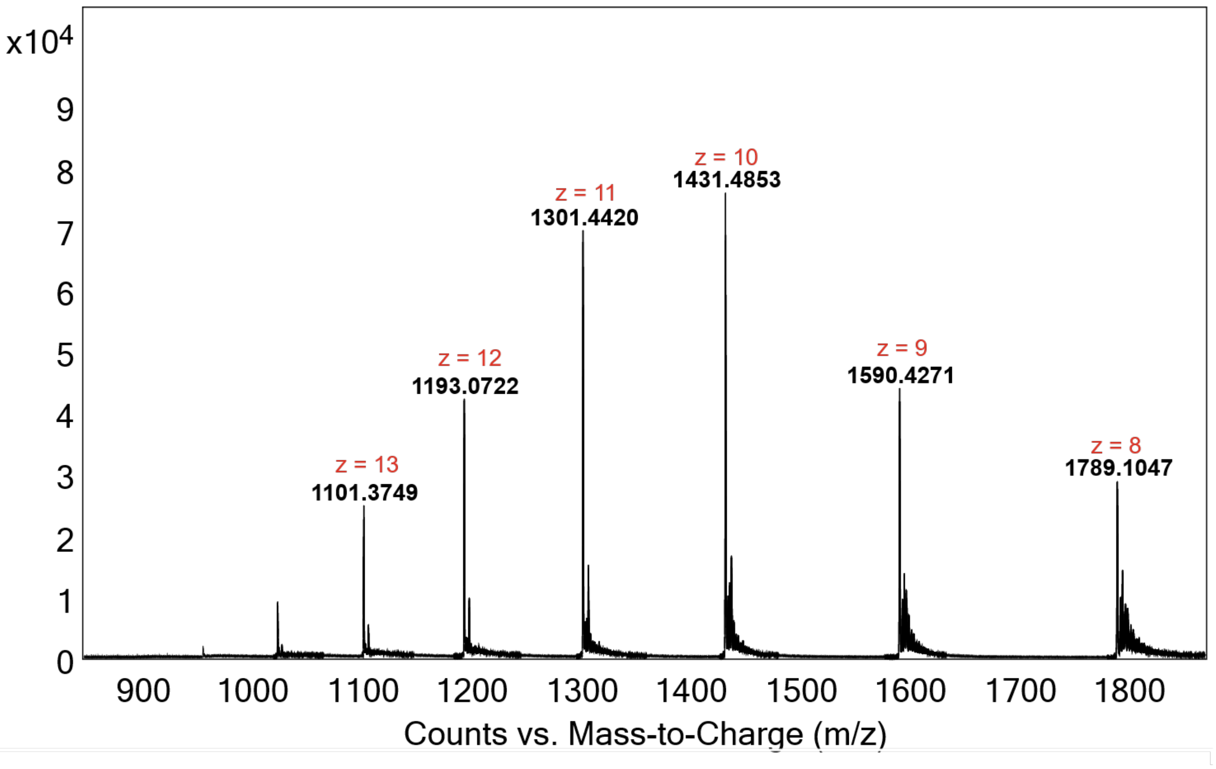

Deconvoluted MS Spectrum of Unmodified Lysozyme Chicken

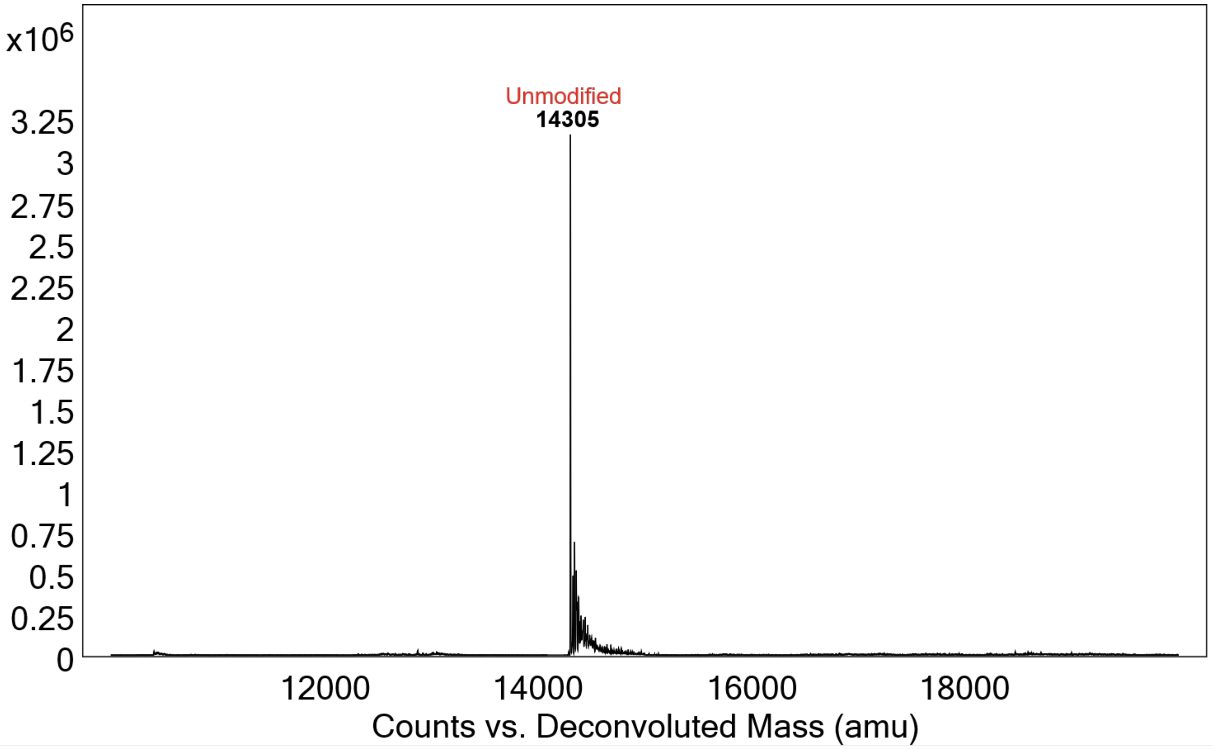

Benzaldehyde Only.

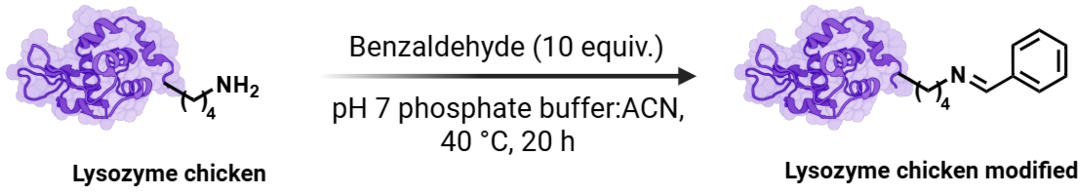

In a one-dram vial, lysozyme chicken (1 mg, 69.9 nmol) was dissolved in 300 µL of sodium phosphate buffer (100 mM, pH 7) and 100 µL of acetonitrile followed by the addition of benzaldehyde (10 equiv.) and left to stir at 37 °C for 20 hours. Subsequently, the reaction mixture was passed through Amicon Ultra 3 kDa spin-concentrator and washed with H_2O (5 x 500 µL) to remove the small molecule impurities. The labeled protein was redissolved in 0.1% formic acid in H_2O and analyzed using LC-MS. The reaction gave no product.

Intact MS Spectrum of Starting Lysozyme Chicken

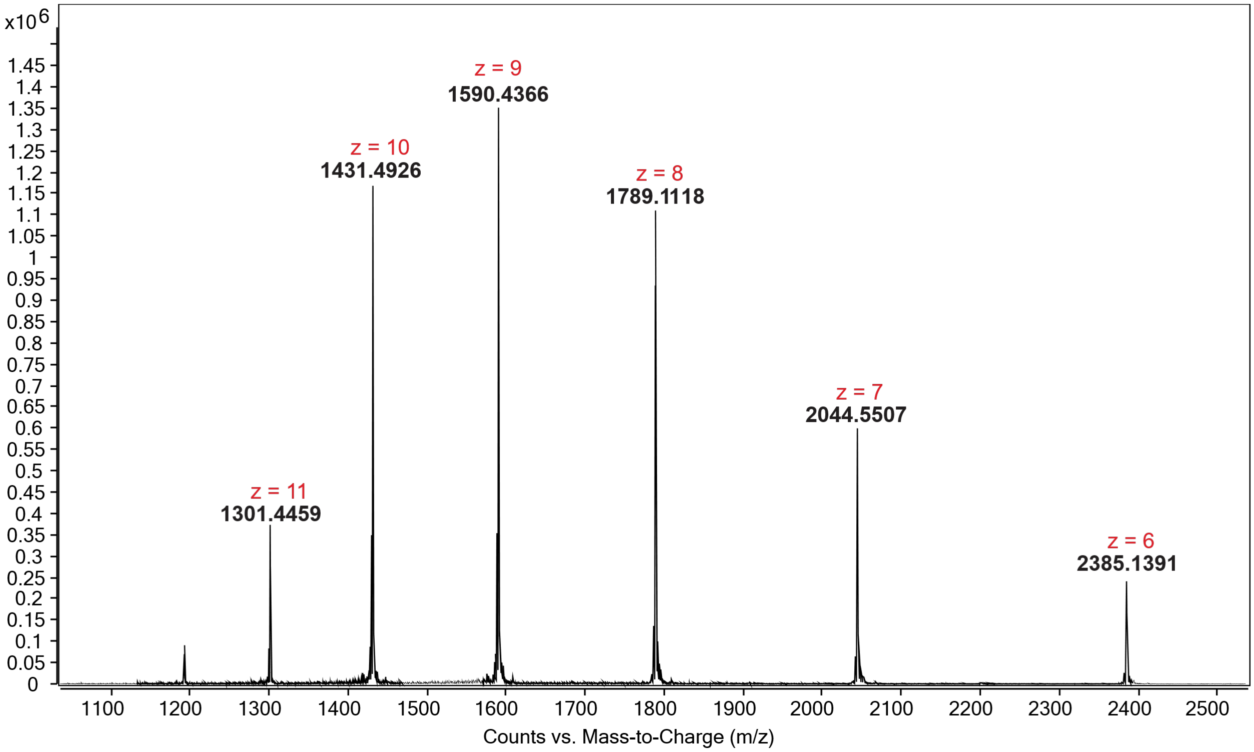

Deconvoluted MS Spectrum of Starting Lysozyme Chicken

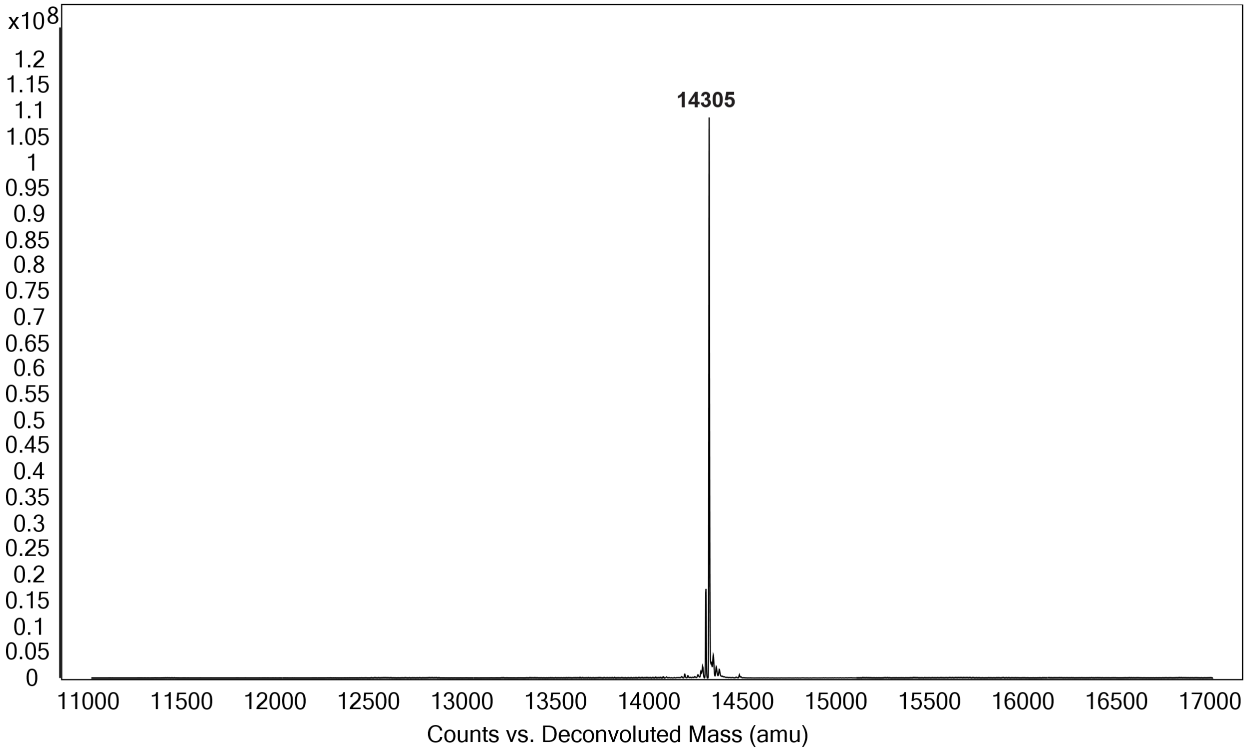

Intact MS Spectrum of Unmodified Lysozyme Chicken

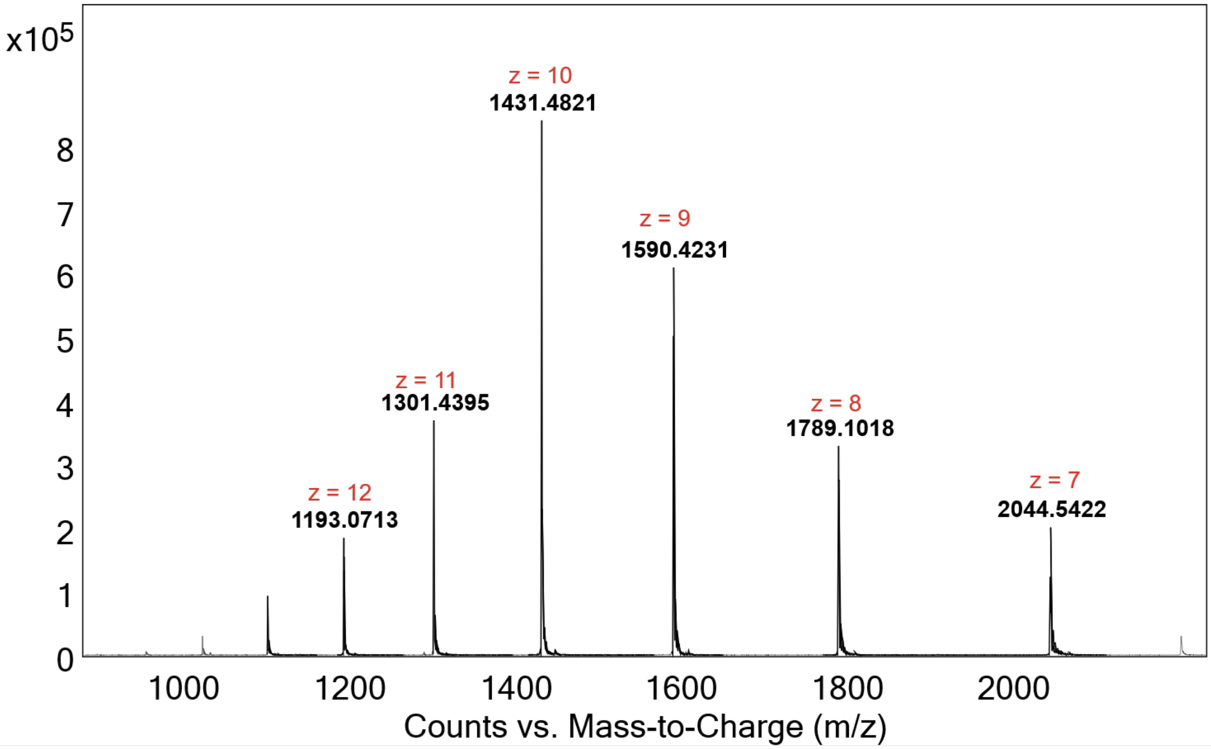

Deconvoluted MS Spectrum of Unmodified Lysozyme Chicken

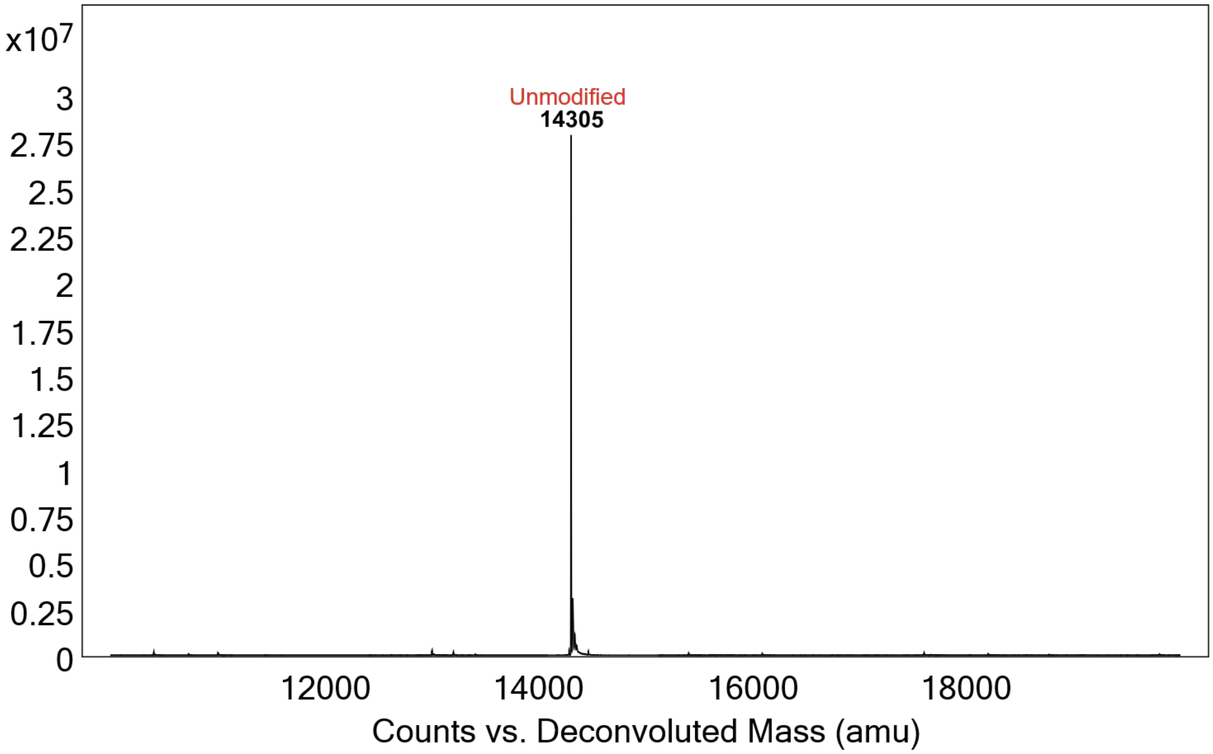

MDA and Benzaldehyde: No Pre-formation.

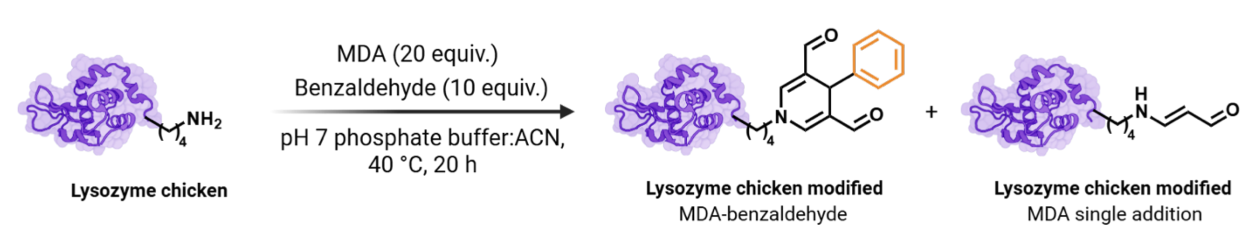

In a one-dram vial, lysozyme chicken (1 mg, 69.9 nmol) was dissolved in 300 µL of sodium phosphate buffer (100 mM, pH 7) and 100 µL of acetonitrile followed by the addition of MDA sodium salt (20 equiv.) and benzaldehyde (10 equiv.) and left to stir at 37 °C for 20 hours. Subsequently, the reaction mixture was passed through Amicon Ultra 3 kDa spin-concentrator and washed with H_2O (5 x 500 µL) to remove the small molecule impurities. The labeled protein was redissolved in 0.1% formic acid in H_2O and analyzed using LC-MS. Modification of MDA-Benzaldehyde is confirmed by a mass shift of +196 *m/z* and of MDA single addition is confirmed by a mass shift of +54 *m/z*. The reaction gave 10% conversion to 1 MDA-Benzaldehyde modification and 21% conversion to 1 MDA single addition.

Intact MS Spectrum of Starting Lysozyme Chicken

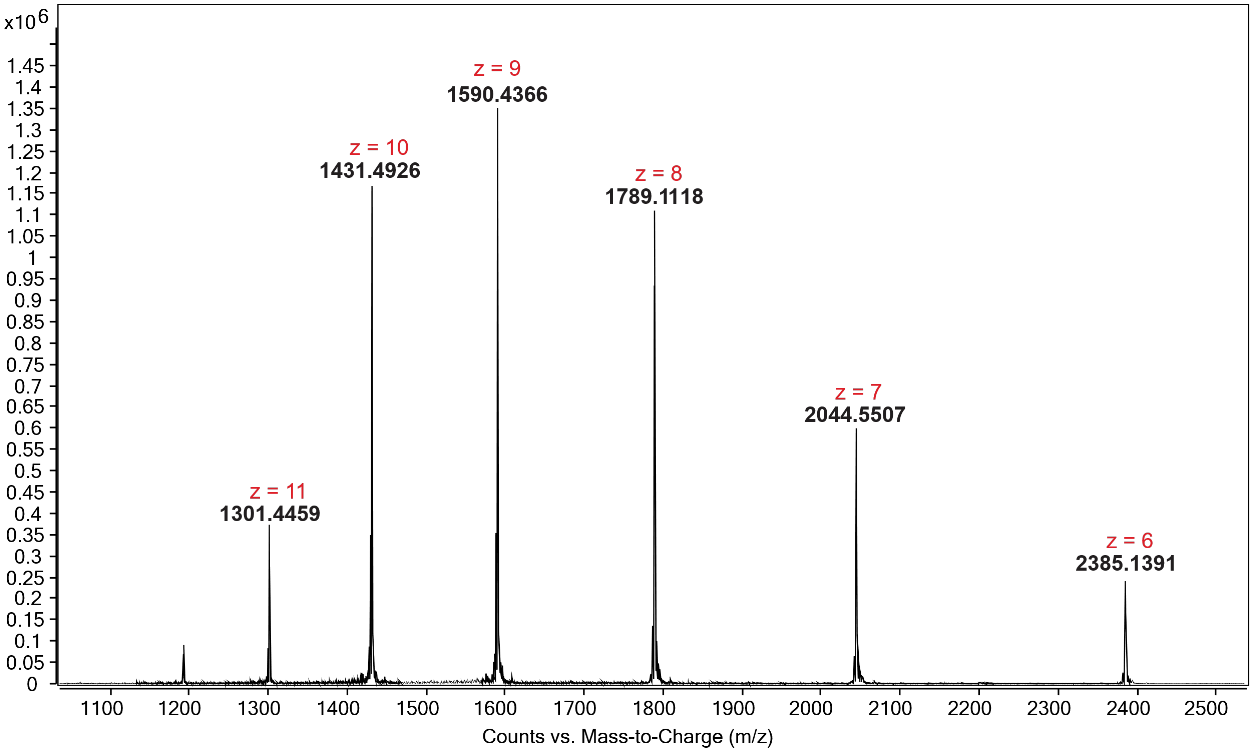

Deconvoluted MS Spectrum of Starting Lysozyme Chicken

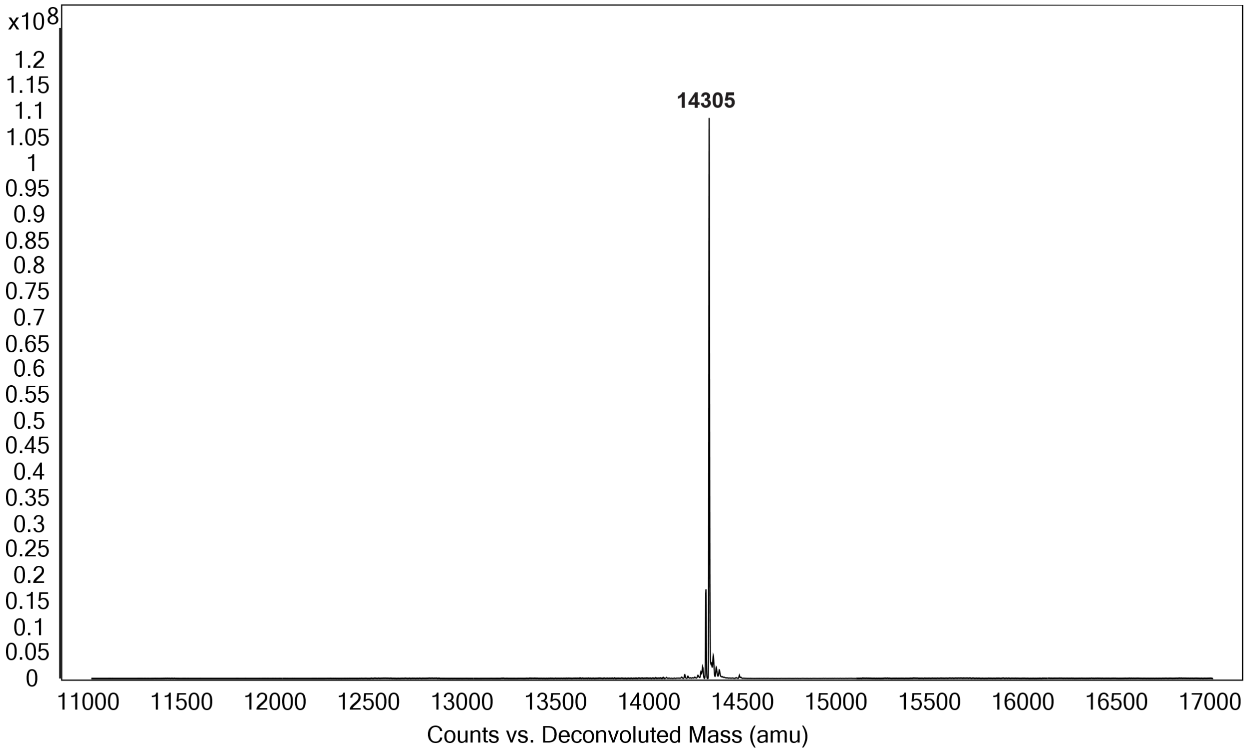

Intact MS Spectrum of Modified Lysozyme Chicken

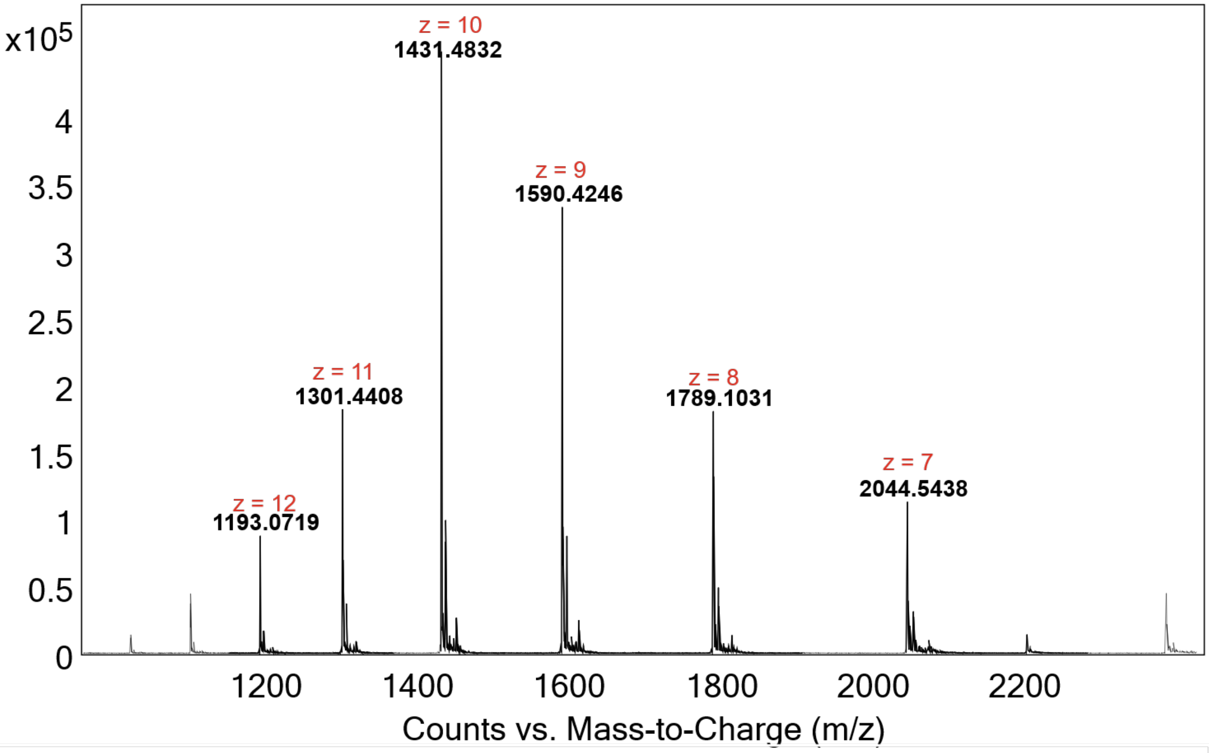

Deconvoluted MS Spectrum of Deconvoluted Lysozyme Chicken

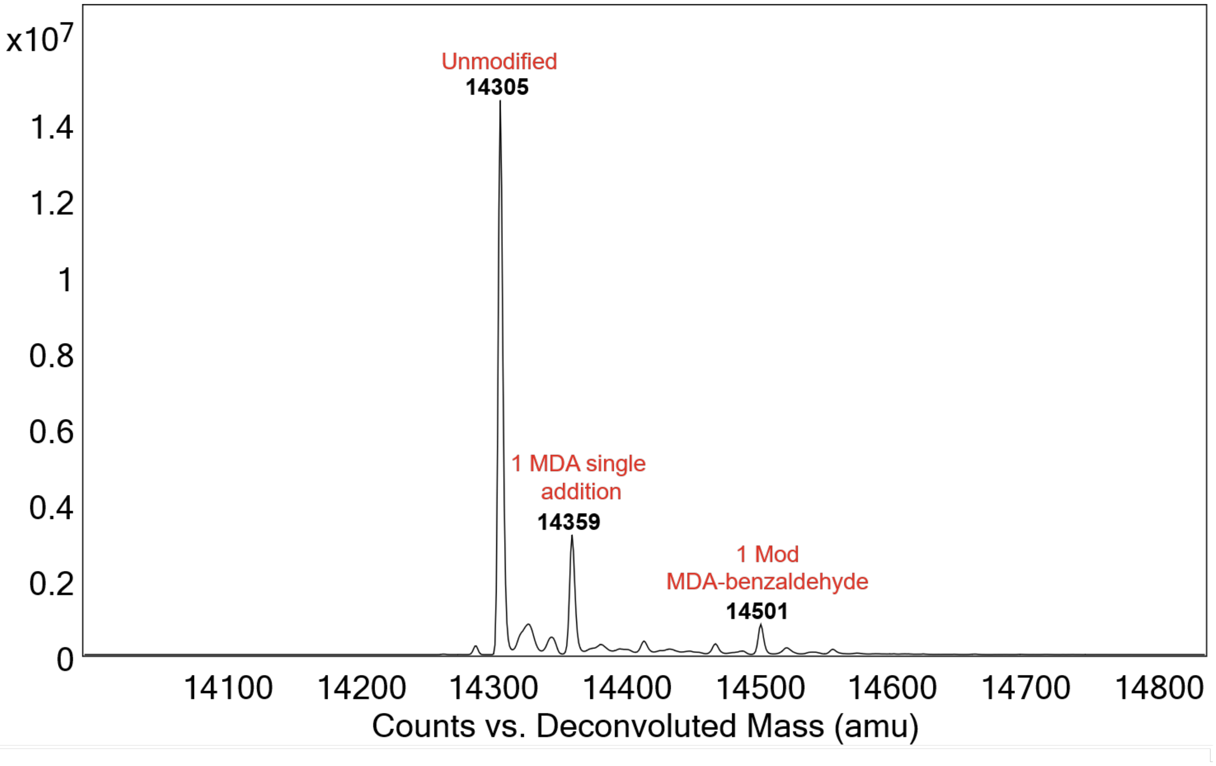

MDA and Benzaldehyde: No Pre-formation, Acidic Conditions.

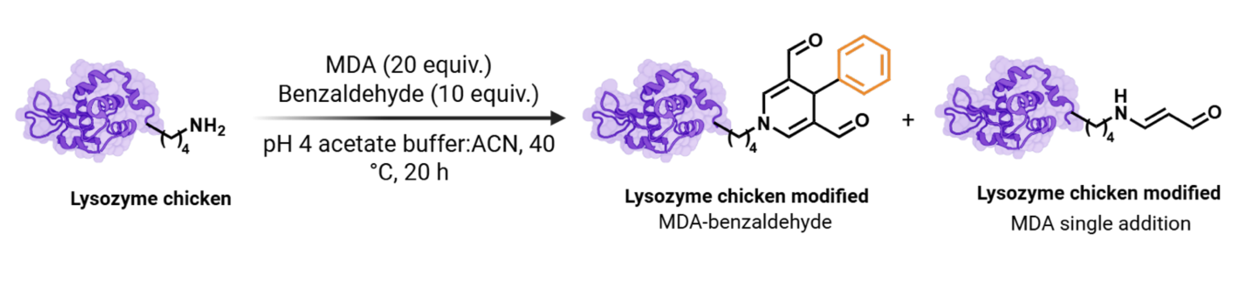

In a one-dram vial, lysozyme chicken (1 mg, 69.9 nmol) was dissolved in 300 µL of sodium acetate buffer (100 mM, pH 4) and 100 µL of acetonitrile followed by the addition of MDA sodium salt (20 equiv.) and benzaldehyde (10 equiv.) and left to stir at 37 °C for 20 hours. Subsequently, the reaction mixture was passed through Amicon Ultra 3 kDa spin-concentrator and washed with H_2O (5 x 500 µL) to remove the small molecule impurities. The labeled protein was redissolved in 0.1% formic acid in H_2O and analyzed using LC-MS. Modification of MDA-Benzaldehyde is confirmed by a mass shift of +196 *m/z* and of MDA single addition is confirmed by a mass shift of +54 *m/z*. The reaction gave 99% conversion to 6 MDA single additions: 3.9% 1 MDA single addition, 11.5% 2 MDA single additions, 23.1% 3 MDA single additions, 26.9% 4 MDA single additions, 23.1% 5 MDA single additions, and 11.5% 6 MDA single additions.

Intact MS Spectrum of Starting Lysozyme Chicken

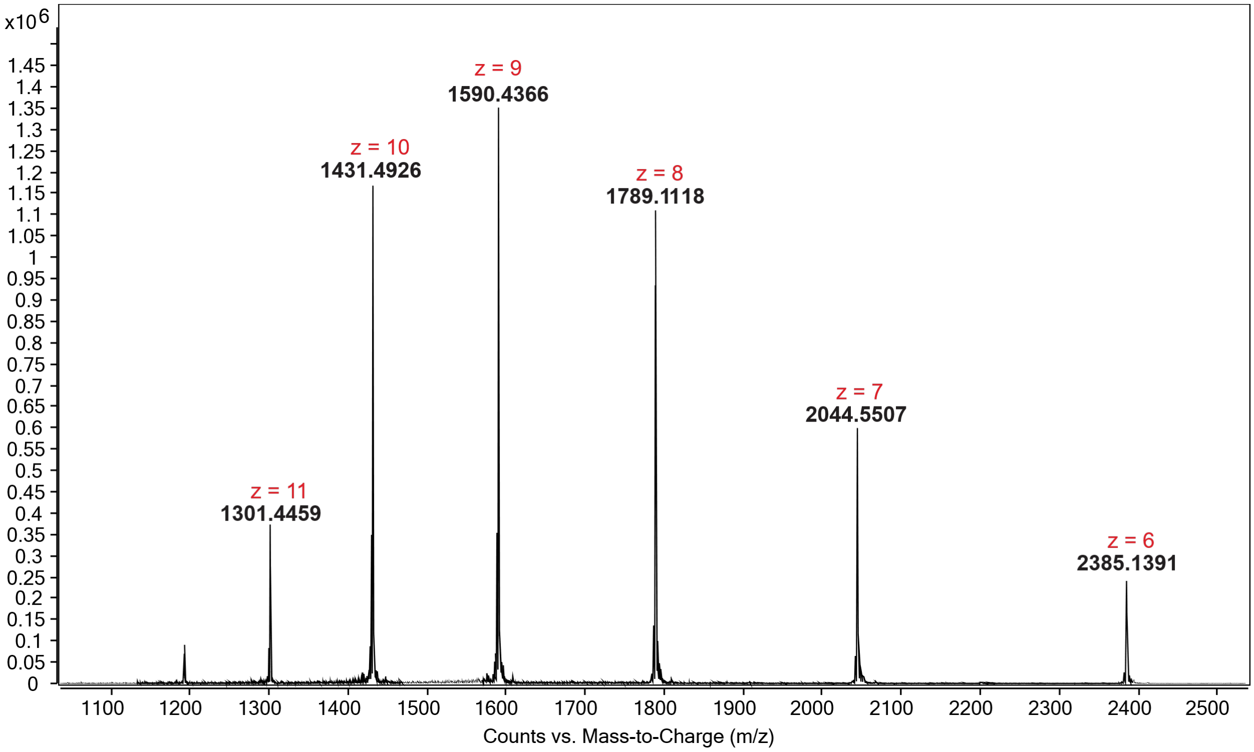

Deconvoluted MS Spectrum of Starting Lysozyme Chicken

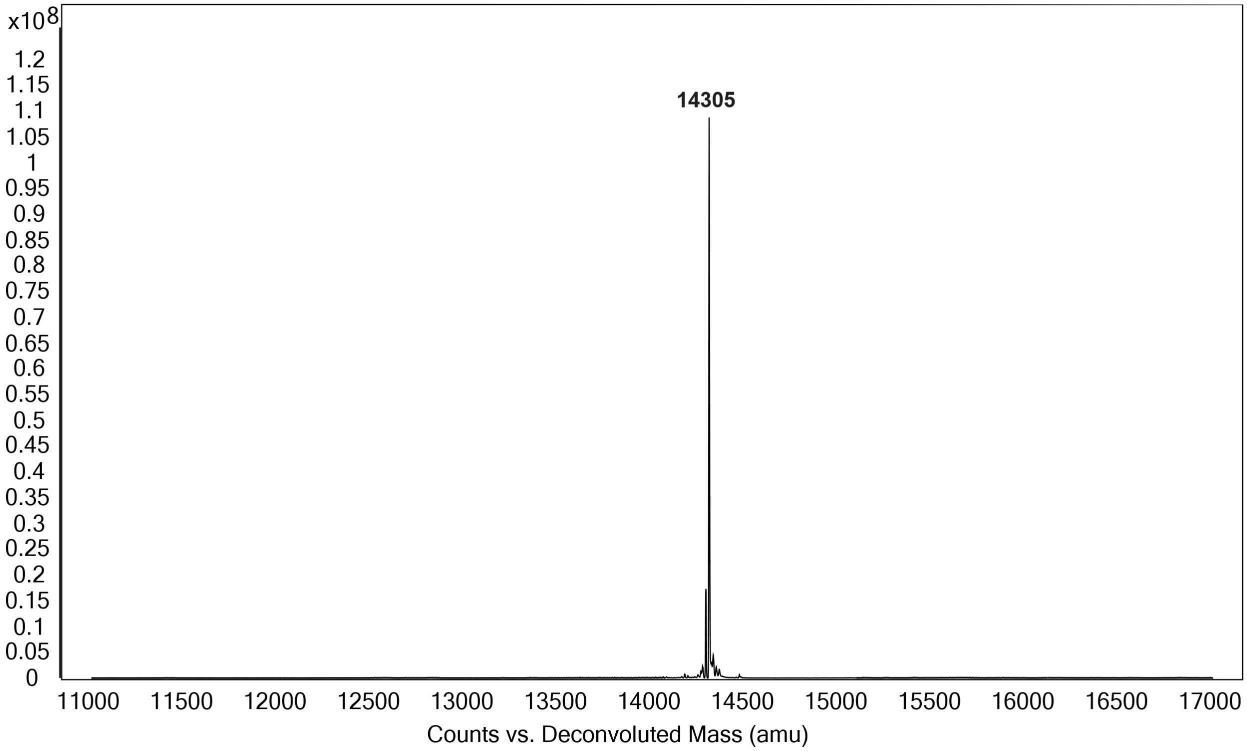

Intact MS Spectrum of Modified Lysozyme Chicken

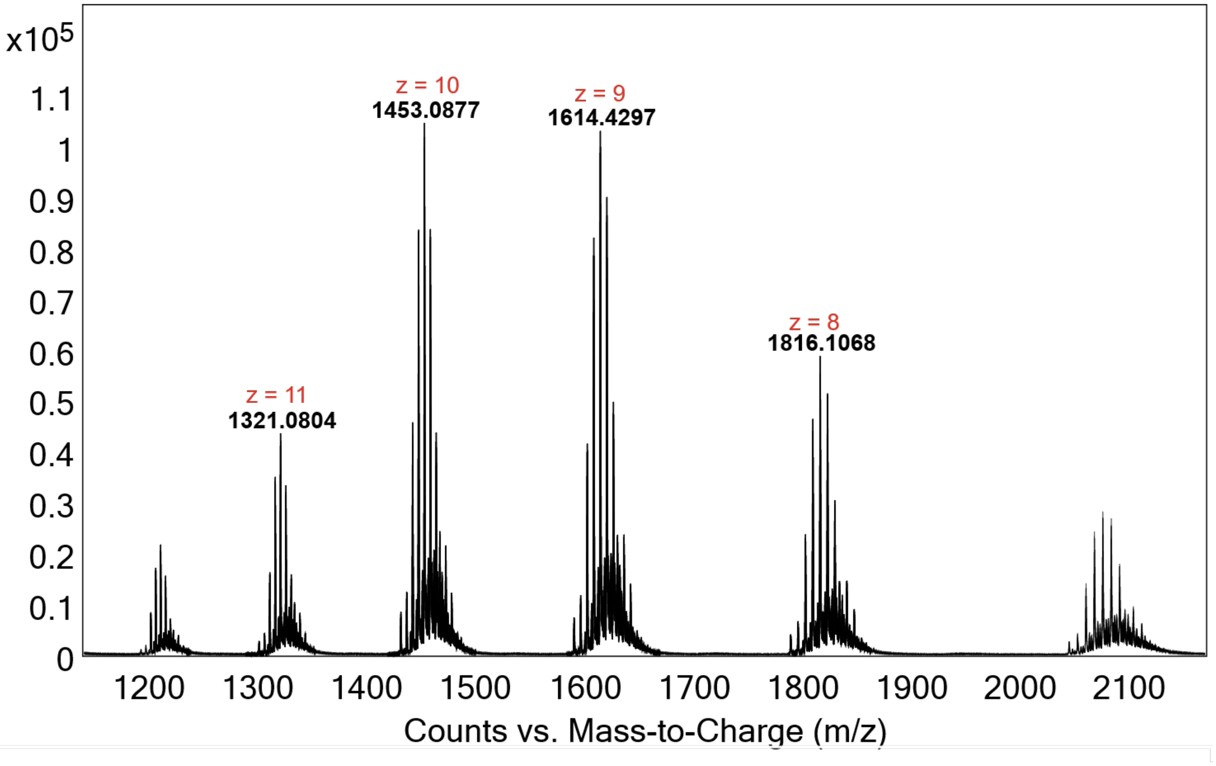

Deconvoluted MS Spectrum of Modified Lysozyme Chicken

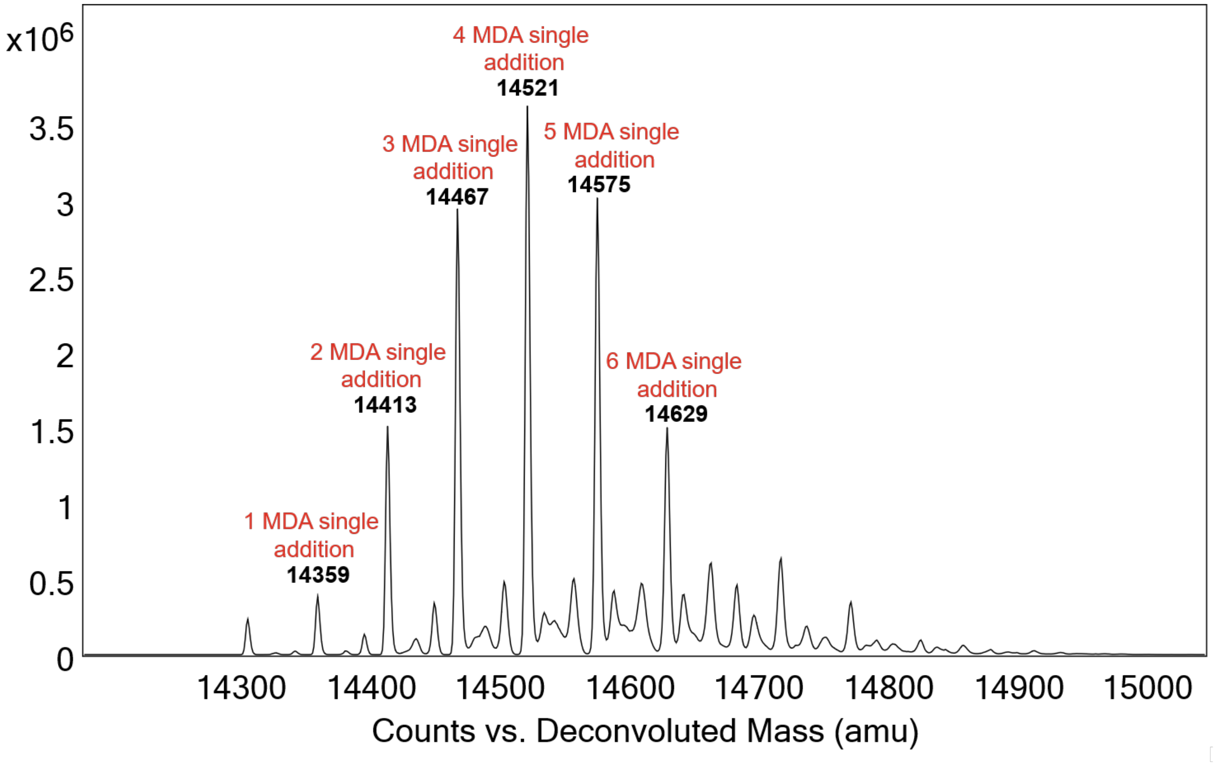

MDA and Benzaldehyde: Pre-formation.

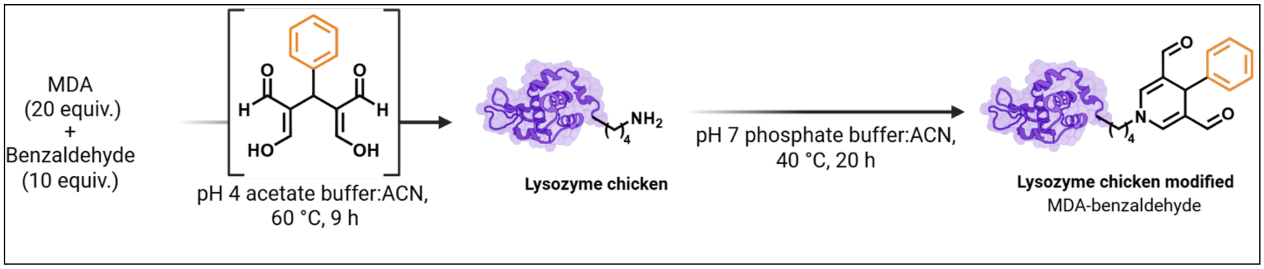

In a one-dram vial, MDA sodium salt (20 equiv.) and benzaldehyde (10 equiv.) are dissolved in 100 μL of sodium acetate buffer (100 mM, pH 4) and 50 μL of acetonitrile, and left to stir at 60 °C for 9 hours. After which, lysozyme chicken (1 mg, 69.9 nmol) is dissolved in 400 μL of sodium phosphate buffer (100 mM, pH 7) and 100 μL of acetonitrile, introduced into the reaction mixture and left to stir at 37 °C for 20 hours. Subsequently, the reaction mixture was passed through Amicon Ultra 3 kDa spin-concentrator and washed with H_2O (5 x 500 μL) to remove the small molecule impurities. The labeled protein was redissolved in 0.1% formic acid in H_2O and analyzed using LC-MS. Modification of MDA-Benzaldehyde is confirmed by a mass shift of +196 *m/z*. The reaction gave 33% conversion of 1 modification and 7% conversion of 2 modifications.

Intact MS Spectrum of Starting Lysozyme Chicken

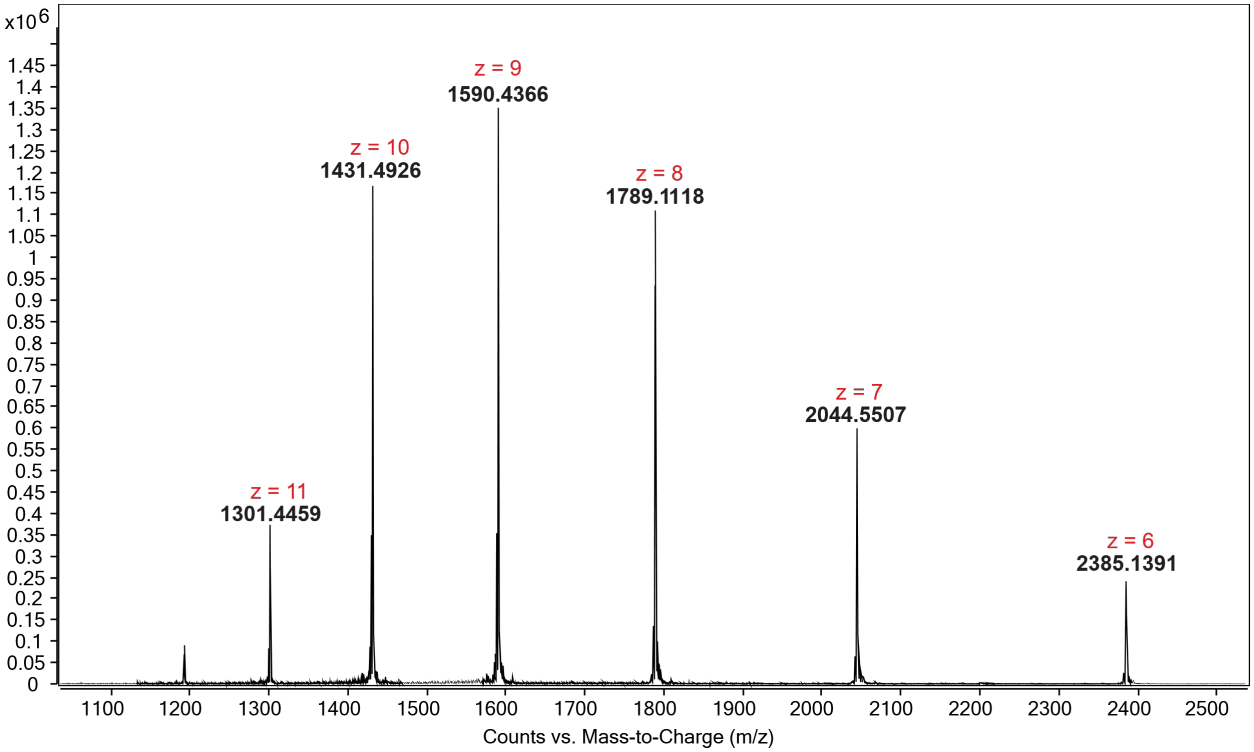

Deconvoluted MS Spectrum of Starting Lysozyme Chicken

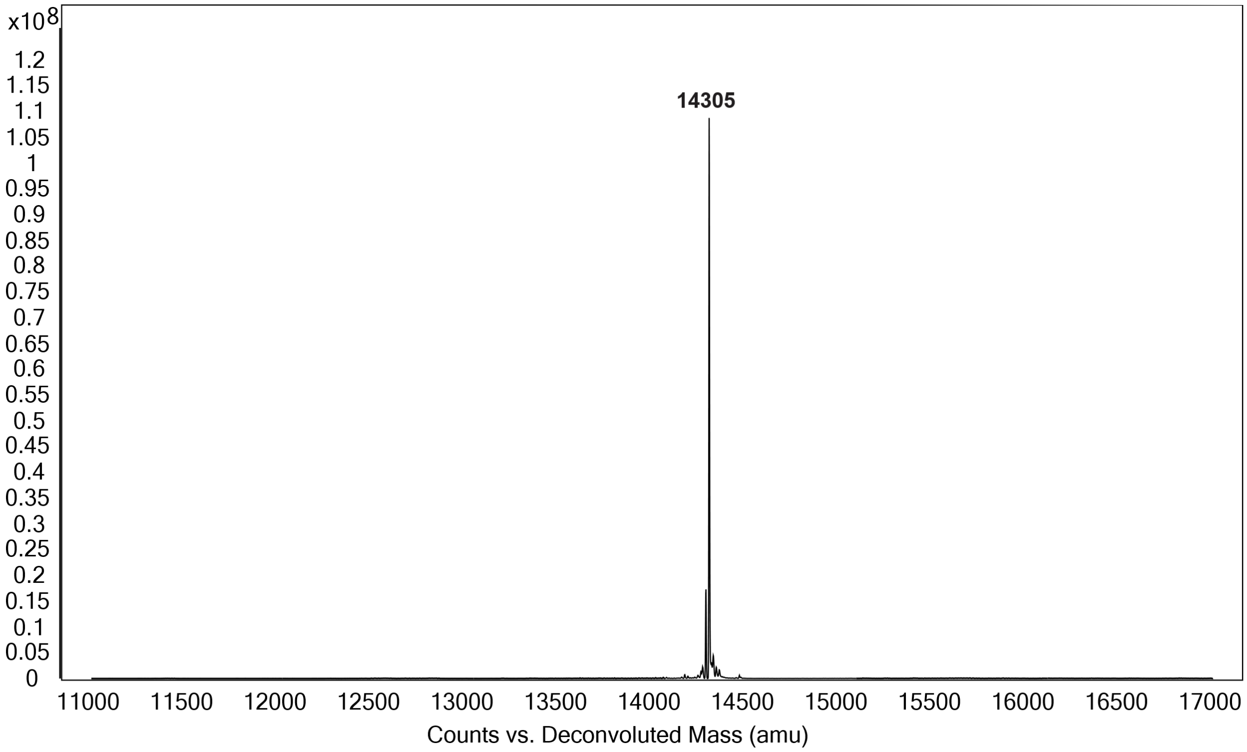

Intact MS Spectrum of Modified Lysozyme Chicken

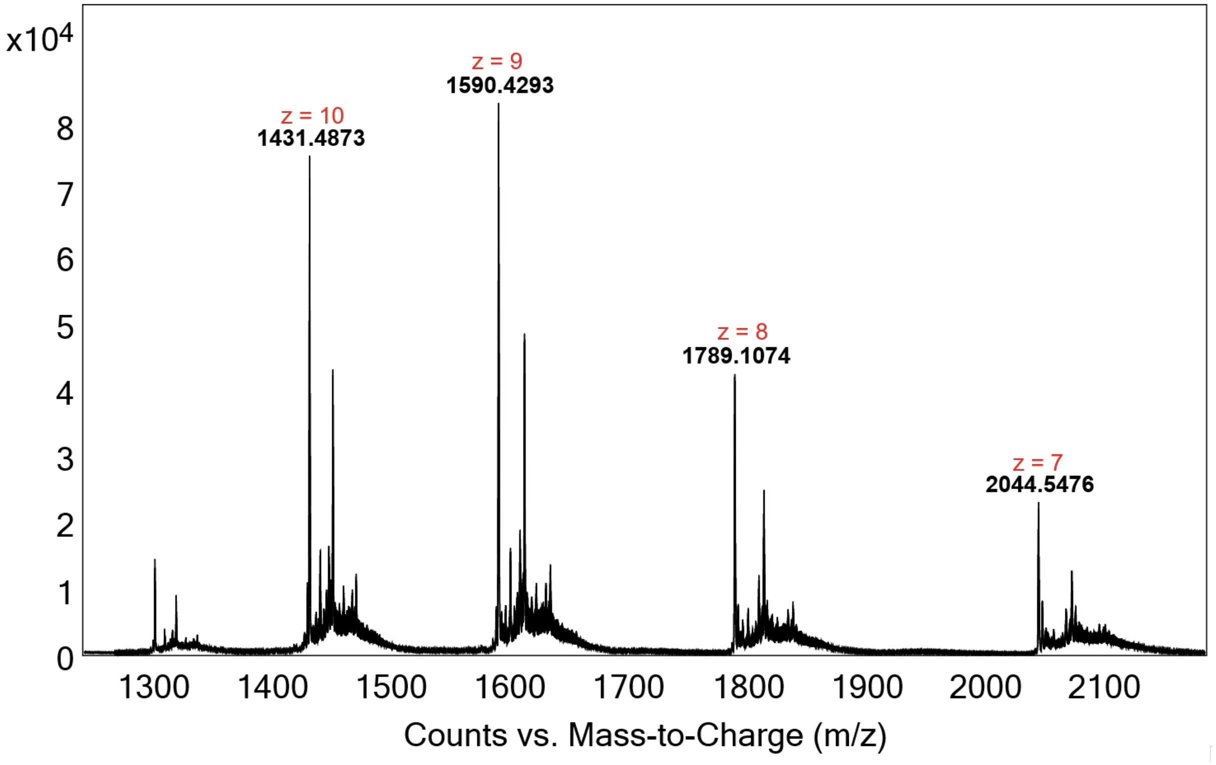

Deconvoluted MS Spectrum of Modified Lysozyme Chicken

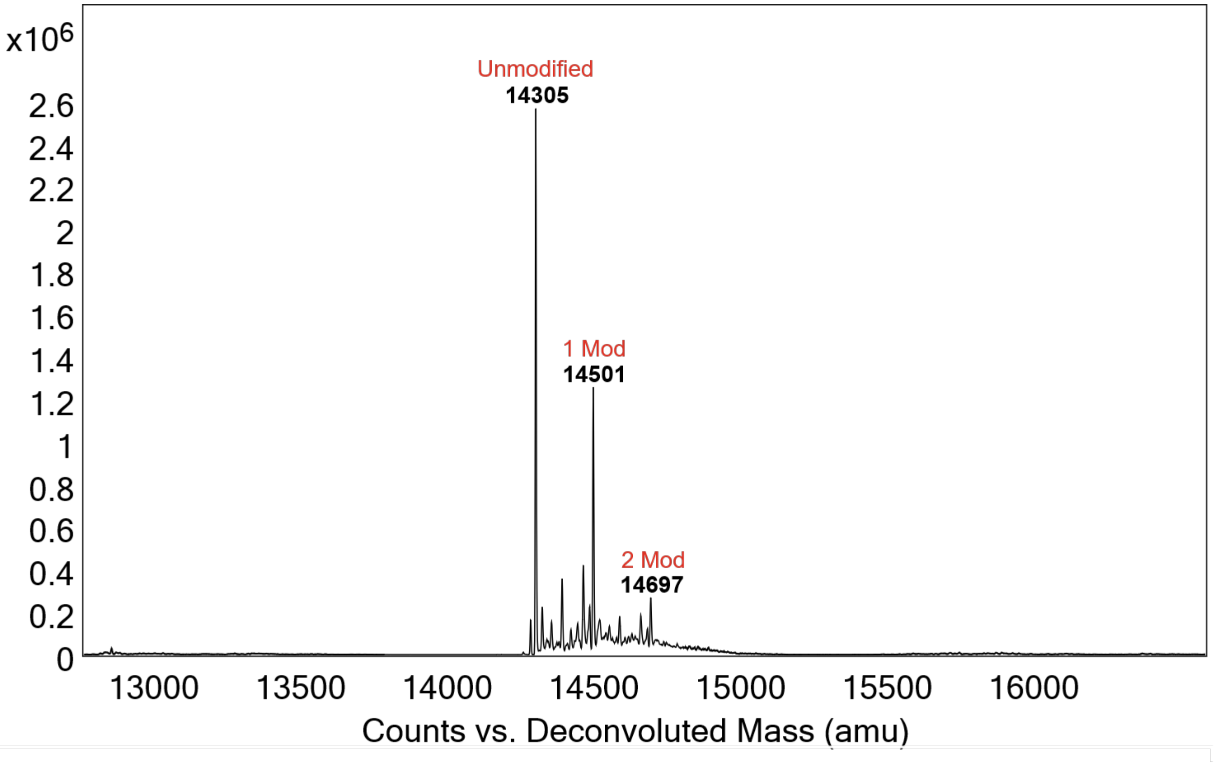

MS/MS Analysis of Lysozyme Chicken MDA-Benzaldehyde Complex.

Identified Peptide Fragment: KVFGR: (Sequence: A 1-5, Lys-MDA-Benzaldehyde 1)

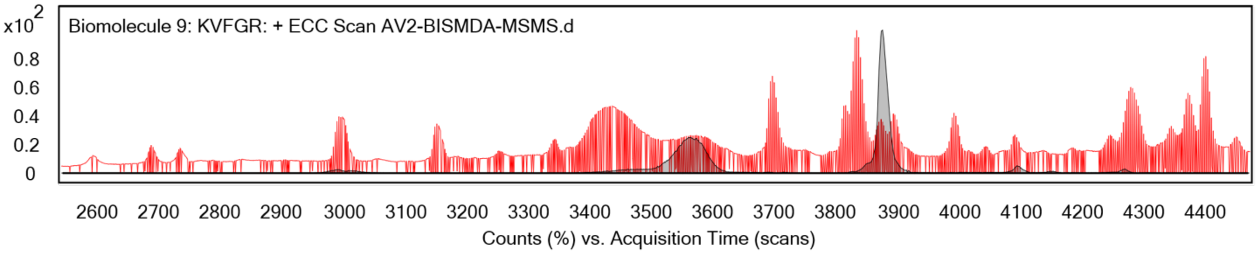

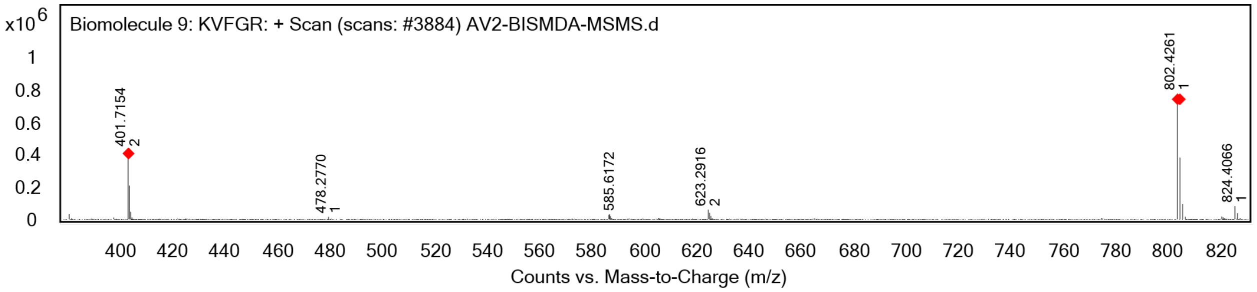

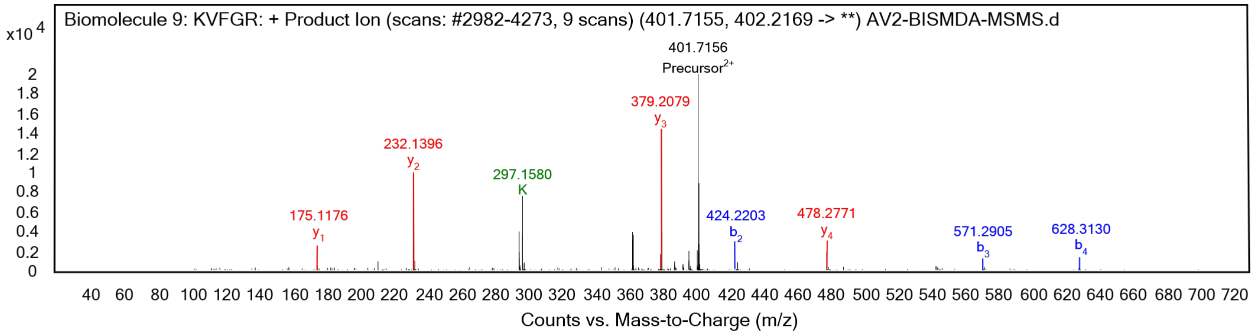

Fragment Spectrum Peaks

m/z	Diff (ppm)	Abund	Ion	Z
175.1176	7.53	515	y1	
232.1396	3.42	9871	y2	
379.2079	2.34	14152	y3	
478.2771	0.38	2812	y4	
424.2203	6.50	2561	b2	
571.2905	1.71	996	b3	
628.3130	0.00	132	b4	
120.0799	7.46	93	F	
297.1580	5.79	7489	K	
401.7156	0.90	19831	Precursor	
402.2184	-1.78	8809	Precursor	
402.7175	4.46	620	Precursor	

Identified Peptide Fragment: KIVSDGNGMNAWVAWR: (Sequence: A 97-112, Lys-MDA-Benzaldehyde 97)

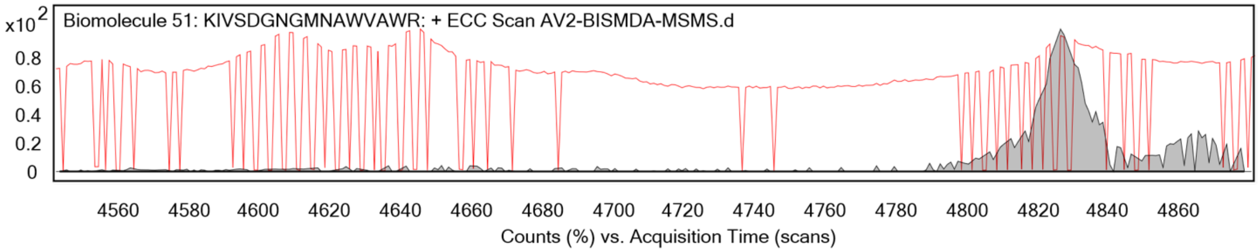

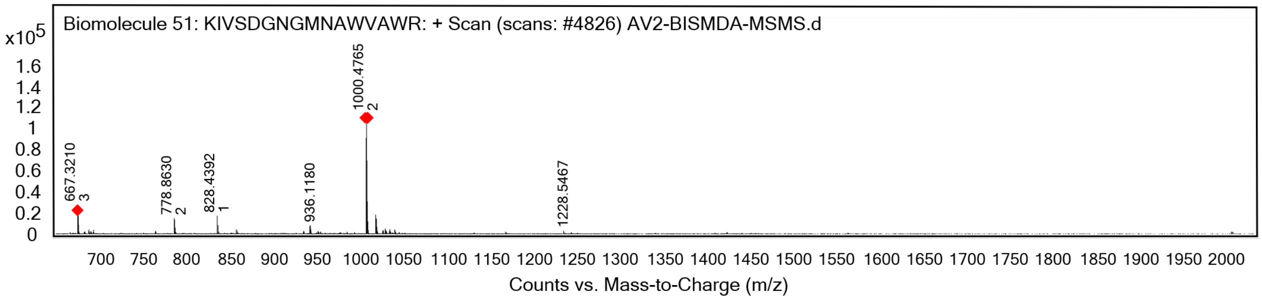

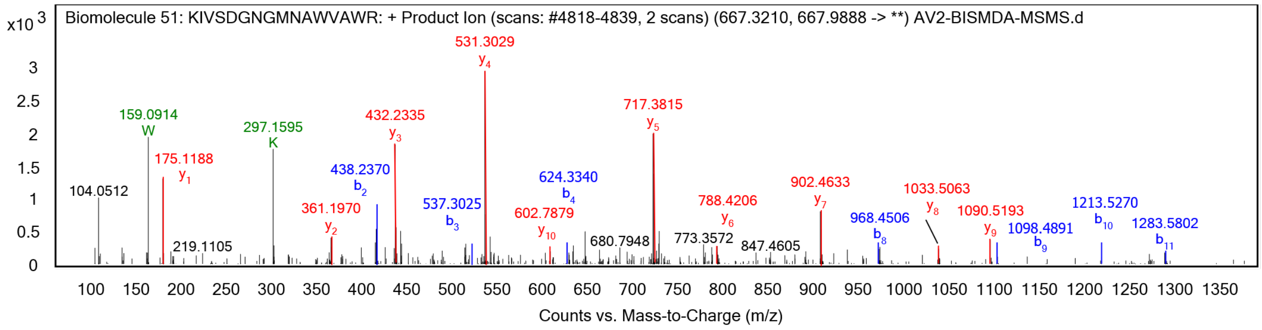

Fragment Spectrum Peaks

m/z	Diff (ppm)	Abund	Ion	Z
175.1188	1.12	1306	y1	
361.1970	3.45	403	y2	
432.2335	4.27	1825	y3	
531.3029	1.66	2947	y4	
717.3815	2.25	1990	y5	
788.4206	-0.46	278	y6	
902.4633	-0.20	807	y7	
1033.5063	-2.54	236	y8	
1090.5193	5.31	375	y9	
359.1941	2.97	180	y5	
451.7342	2.32	105	y7	
602.7879	-0.48	53	y10	
438.2370	3.93	507	b2	
537.3025	8.65	421	b3	
624.3340	8.27	177	b4	
968.4506	4.88	262	b8	
1098.4891	3.08	116	b9	
1213.5270	9.66	112	b10	
1283.5802	-6.02	181	b11	
607.2785	-9.03	107	b10	
159.0914	1.63	1942	W	
297.1595	1.00	1758	K	

Lysozyme Chicken MDA-MDA Complex.

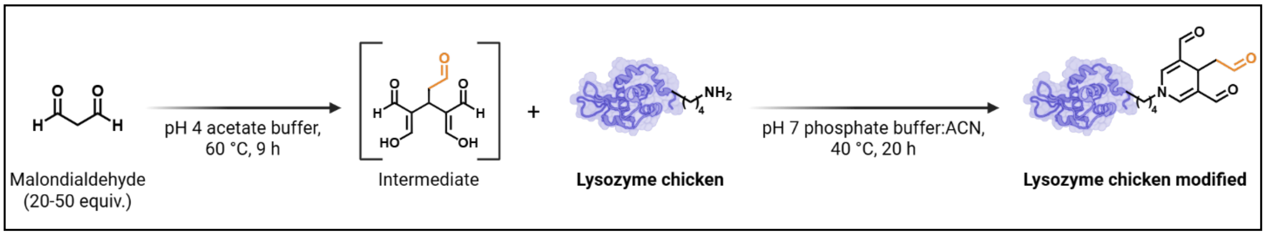

In a one-dram vial, MDA sodium salt (20-50 equiv.) was dissolved in 100 µL of sodium acetate buffer (100 mM, pH 4) and left to stir at 60 °C for 9 hours. Then, lysozyme chicken (1 mg, 69.9 nmol) was dissolved in 300 µL of sodium phosphate buffer (100 mM, pH 7) and 100 µL of acetonitrile, added to the reaction mixture and left to stir at 37 °C for 20 hours. Subsequently, the reaction mixture was passed through Amicon Ultra 3 kDa spin-concentrator and washed with H_2O (5 x 500 µL) to remove the small molecule impurities. The labeled protein was redissolved in 0.1% formic acid in H_2O and analyzed using LC-MS. See conversion for each attempted condition in the table below. Modification of MDA-MDA is confirmed by a mass shift of +162 *m/z*. Using 50 equivalents of MDA sodium salt (Entry 3) was found to be the optimized condition for obtaining 3 modifications (>95%).

Optimization table

Entry	MDA (equiv.)	% conversion				
		Unmodified	1 Mod	2 Mod	3 Mod	Total
1	20	32%	48%	19%	0%	67%
2	30	19%	51%	30%	0%	81%
3	50	0%	28%	44%	28%	**>95%**

Intact MS Spectrum of Starting Lysozyme Chicken

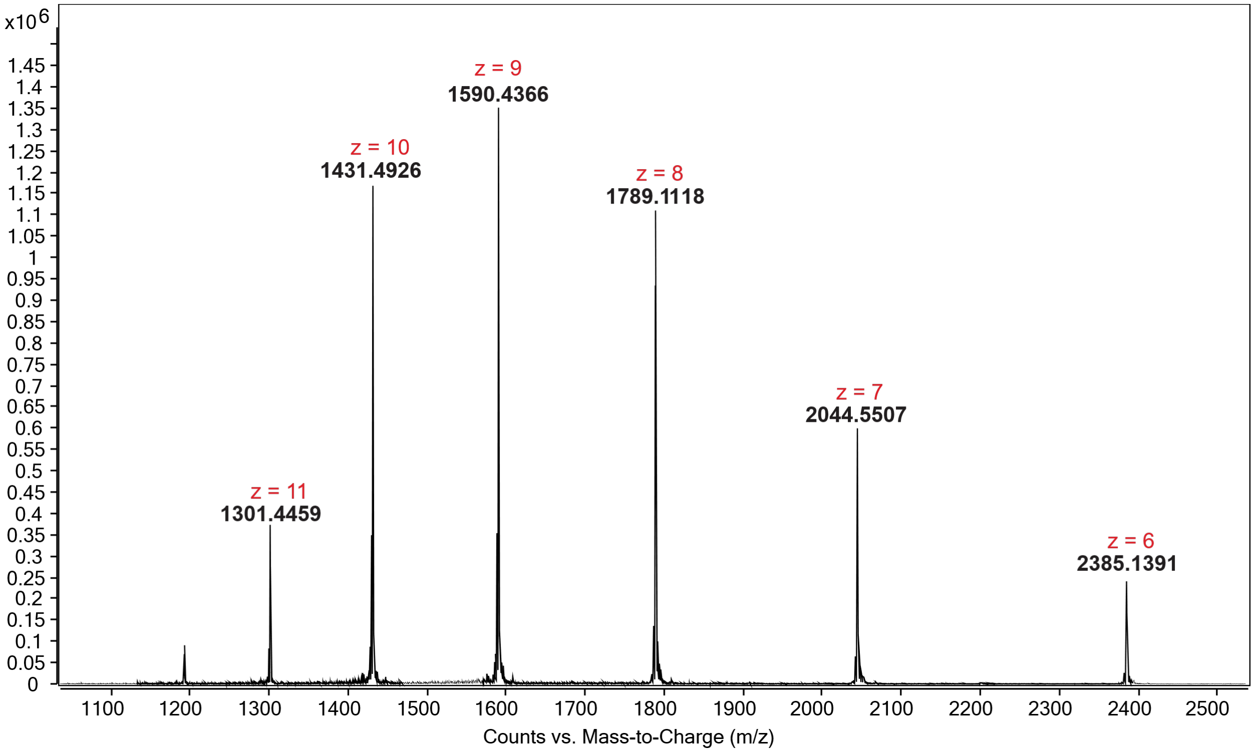

Deconvoluted MS Spectrum of Starting Lysozyme Chicken

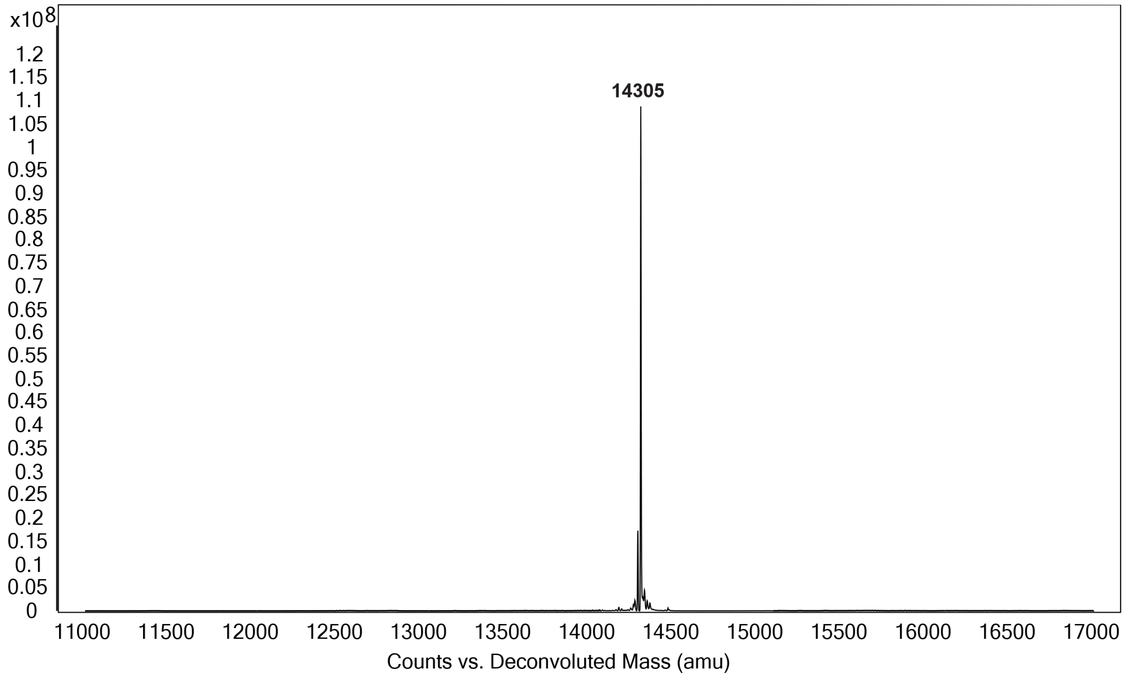

Intact MS Spectrum of Modified Lysozyme Chicken (Entry 1)

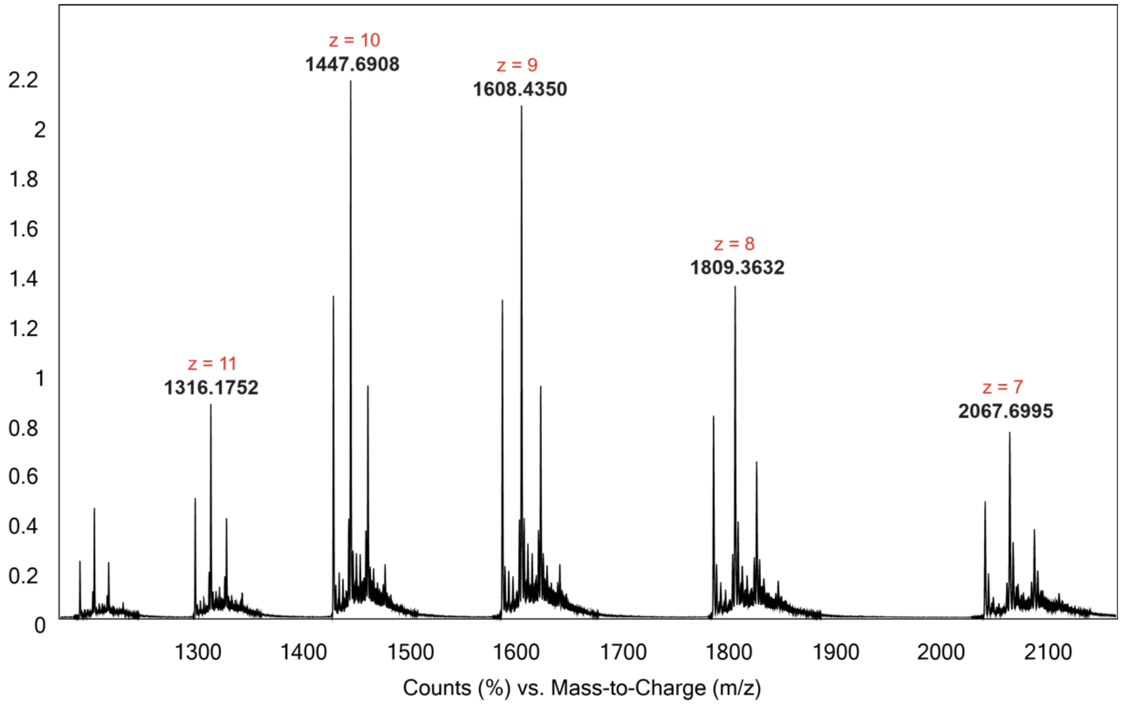

Deconvoluted MS Spectrum of Modified Lysozyme Chicken (Entry 1)

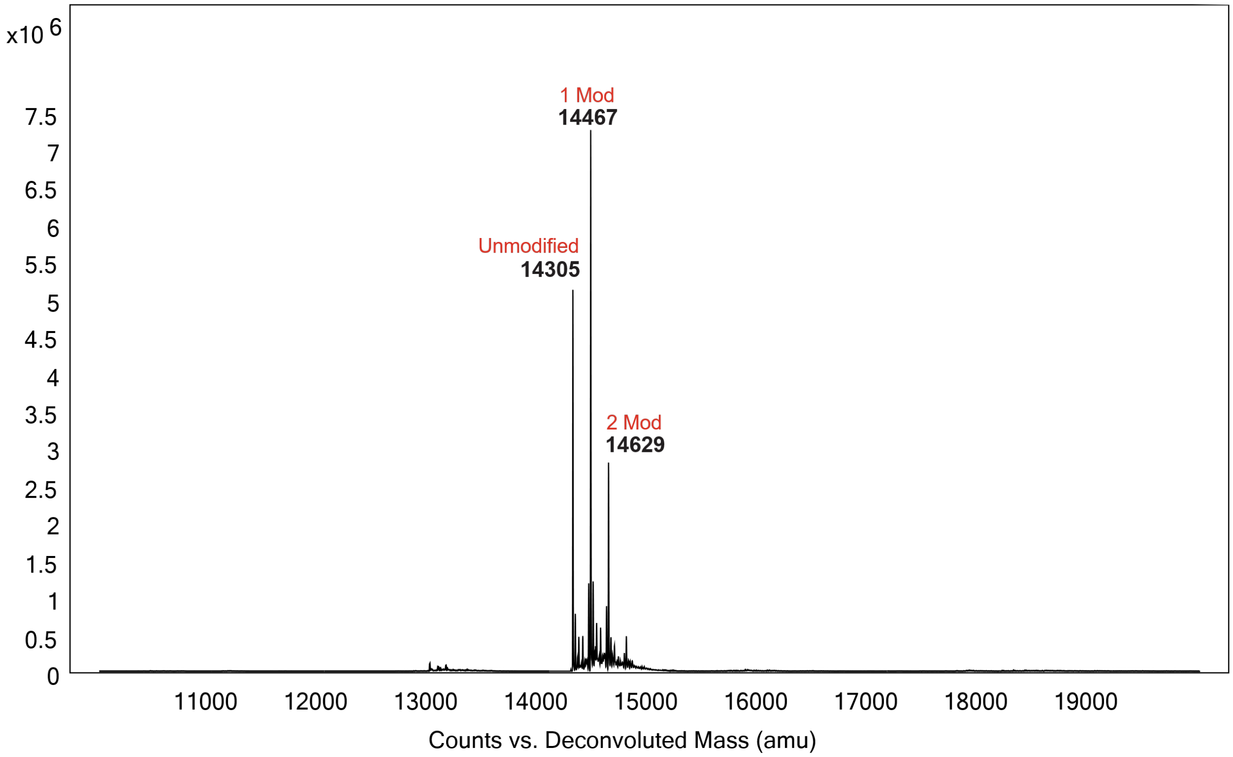

Intact MS Spectrum of Modified Lysozyme Chicken (Entry 2)

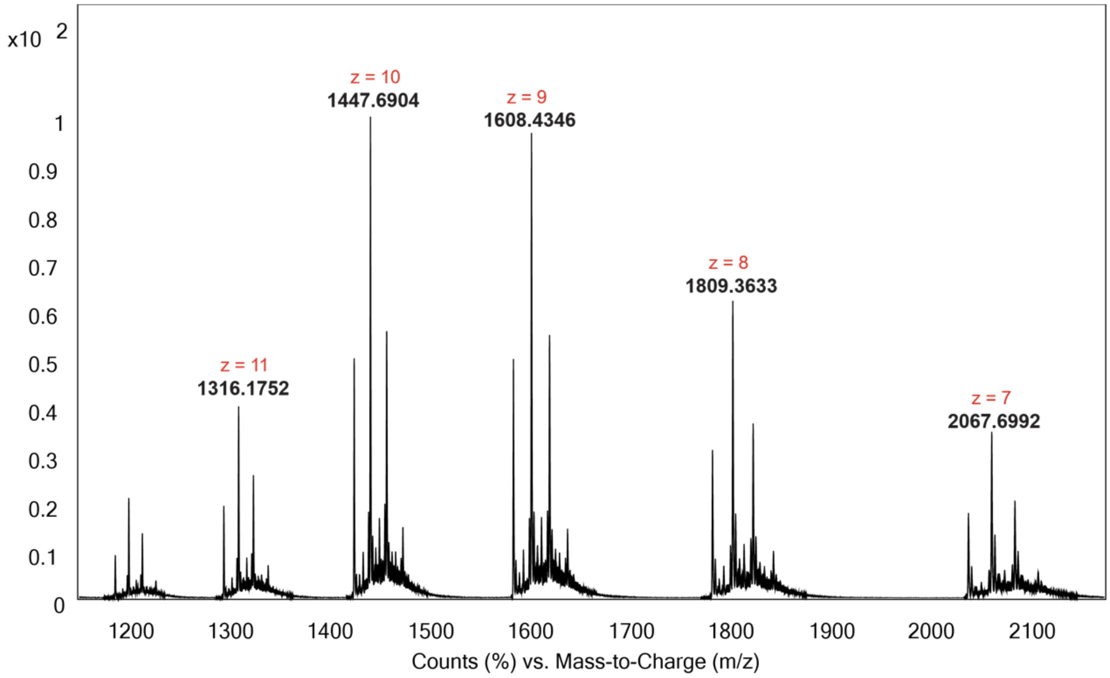

Deconvoluted MS Spectrum of Modified Lysozyme Chicken (Entry 2)

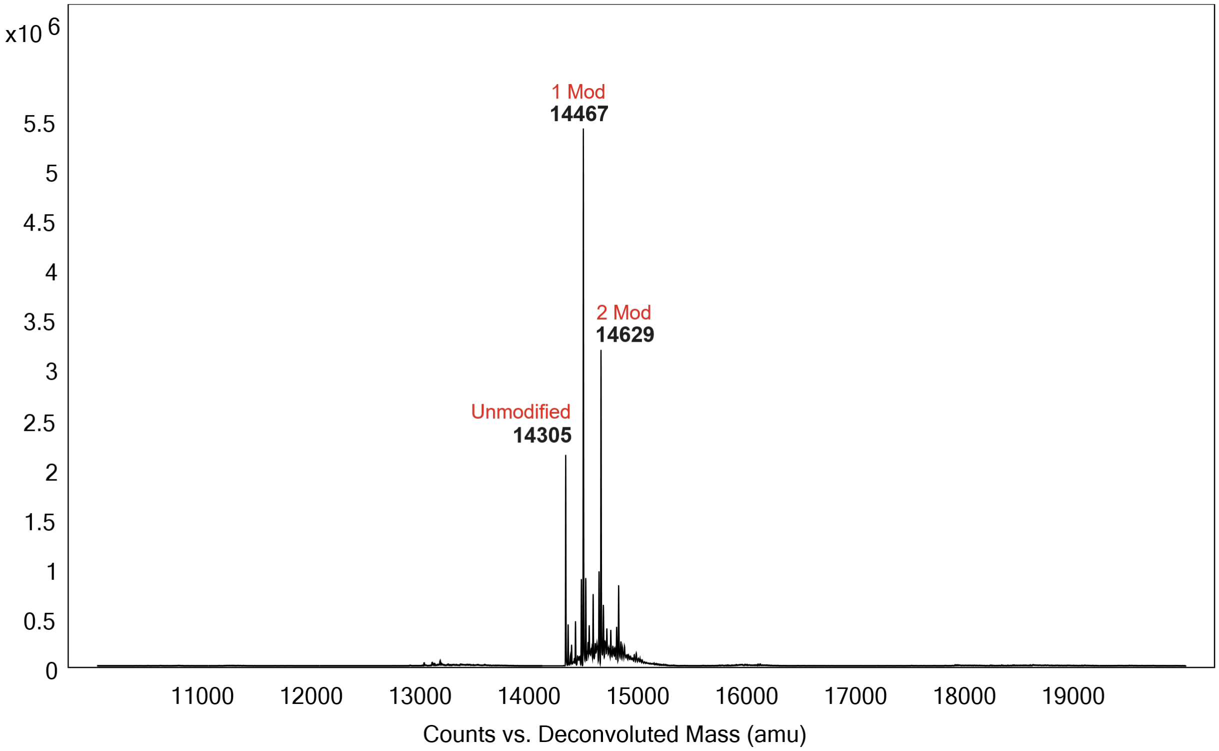

Intact MS Spectrum of Modified Lysozyme Chicken (Entry 3)

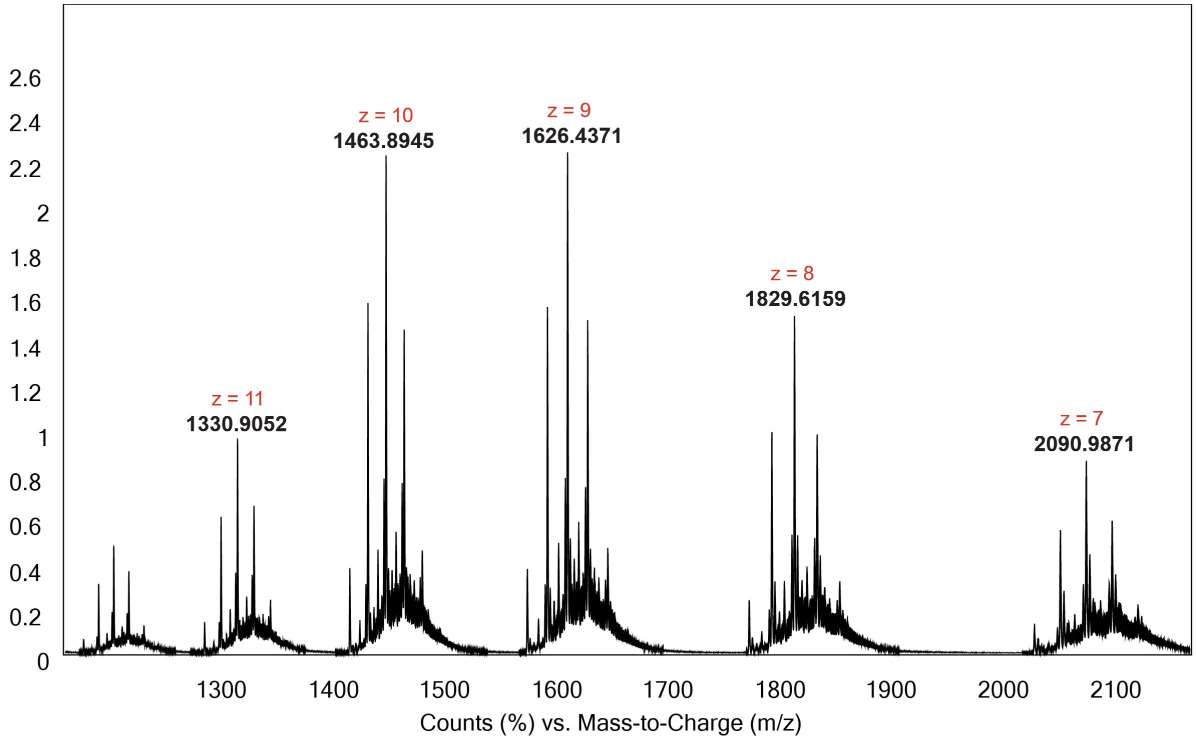

Deconvoluted MS Spectrum of Modified Lysozyme Chicken (Entry 3)

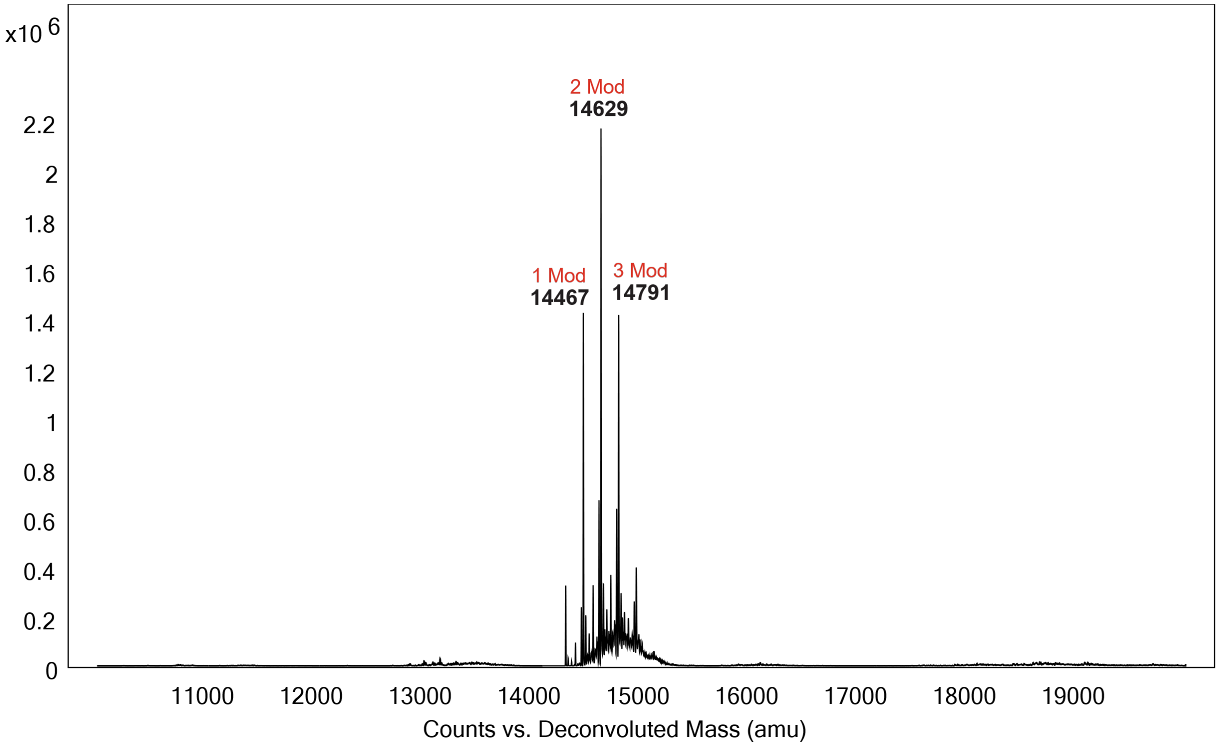

Supplementary Figure 16. Lysozyme chicken NHS-ester alkyne.

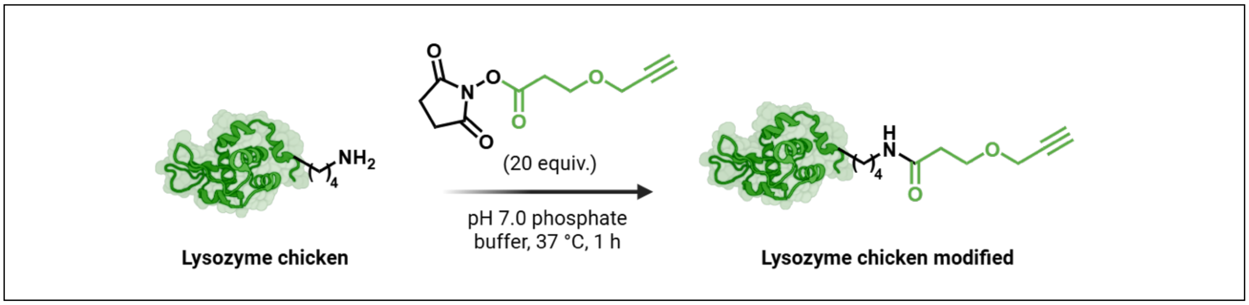

In a one-dram vial, lysozyme chicken (1 mg, 69.9 nmol) was dissolved in 500 µL of sodium phosphate buffer (100 mM, pH 7) followed by the addition of NHS-ester alkyne (20 equiv.) and left to stir at 37 °C for 1 hour. Subsequently, the reaction mixture was passed through Amicon Ultra 3 kDa spin-concentrator and washed with H_2O (5 x 500 µL) to remove the small molecule impurities. The labeled protein was redissolved in 0.1% formic acid in H_2O and analyzed using LC-MS. Modification of NHS-ester alkyne is confirmed by a mass shift of +110 *m/z*. The conversion was found to be 99% with 6 modifications.

Intact MS Spectrum of Starting Lysozyme Chicken

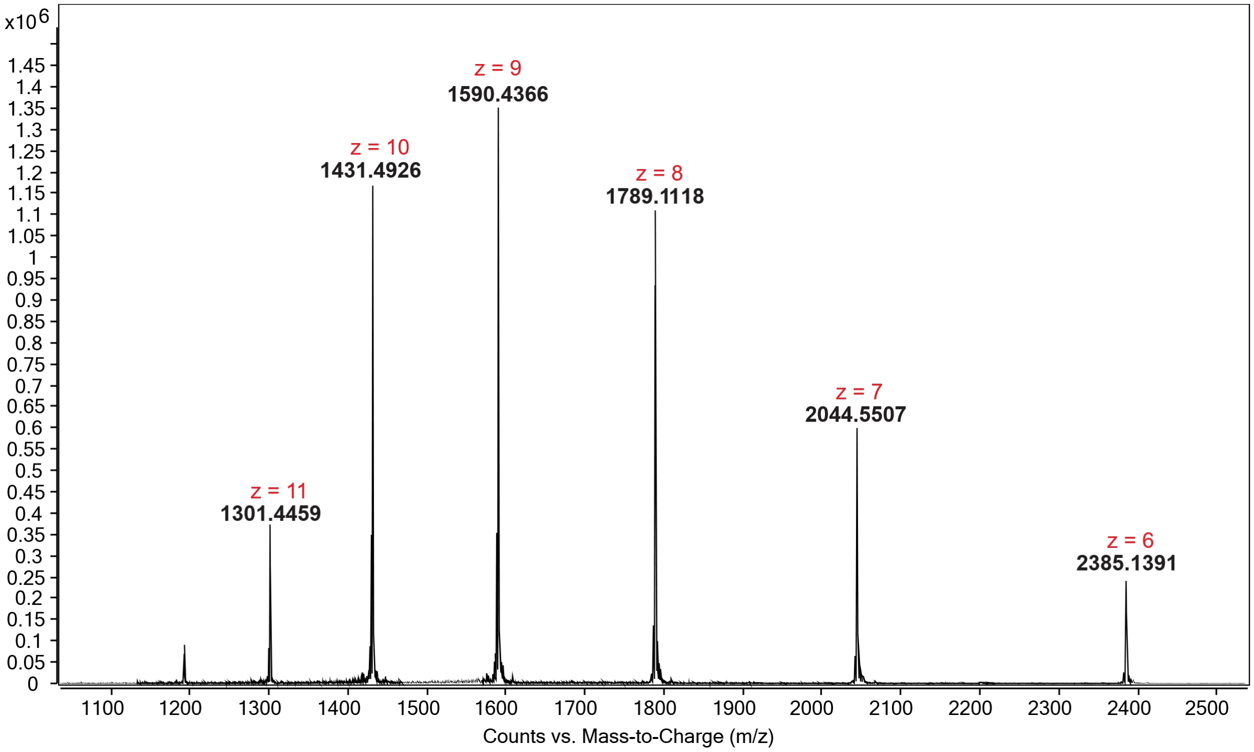

Deconvoluted MS Spectrum of Starting Lysozyme Chicken

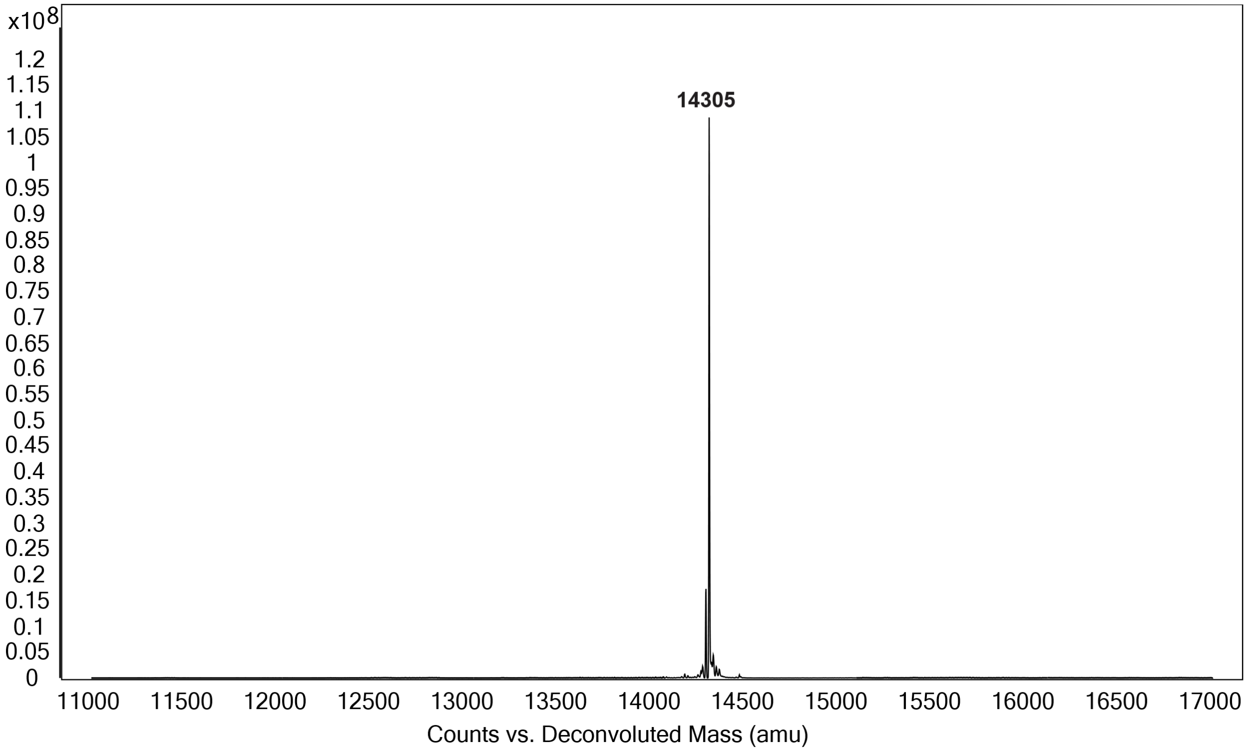

Intact MS Spectrum of Modified Lysozyme Chicken

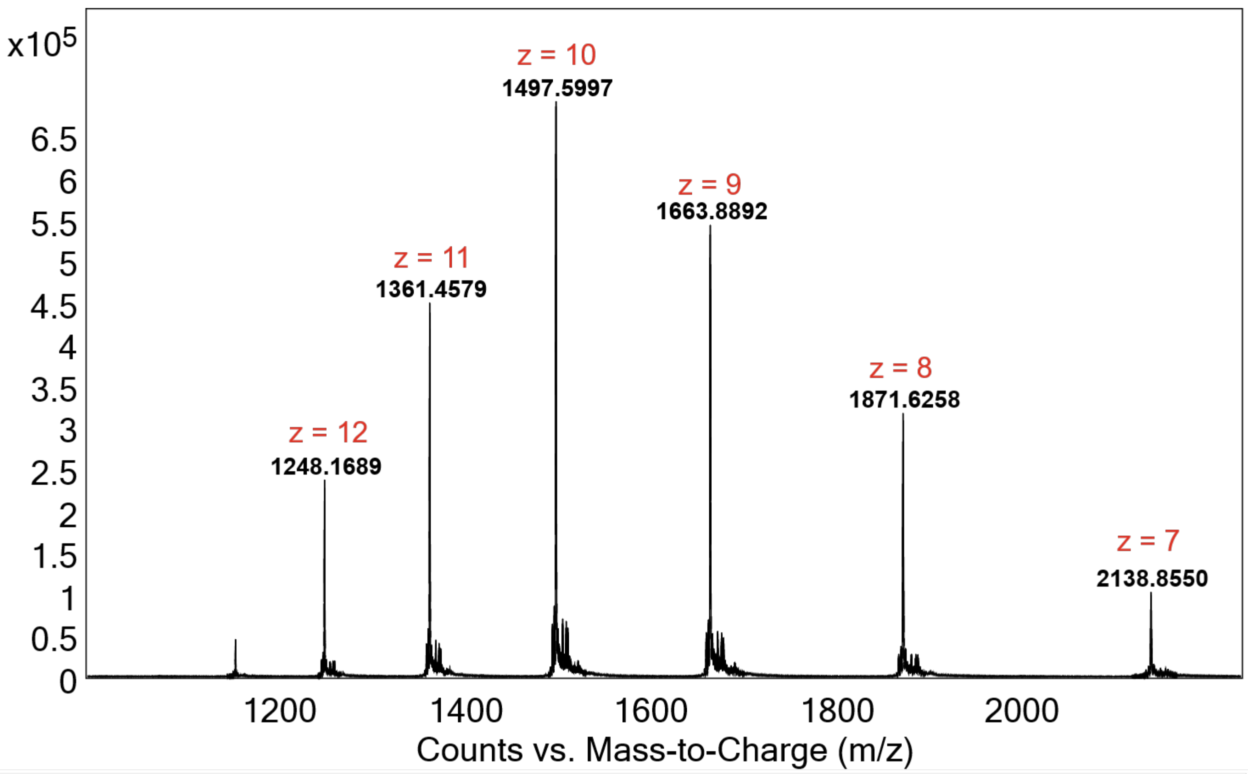

Deconvoluted MS Spectrum of Modified Lysozyme Chicken

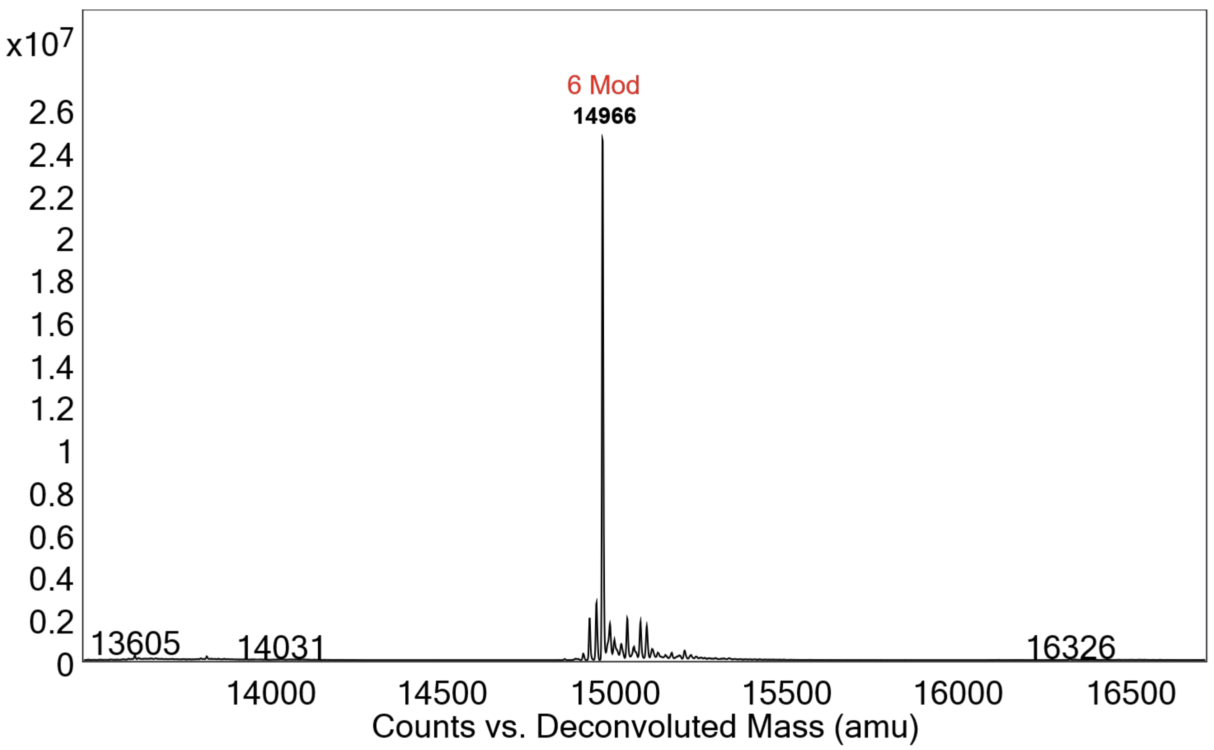

Supplementary Figure 17. Proteins Substrate Scope MDA-Benzaldehyde Complex.

Lysozyme Human.

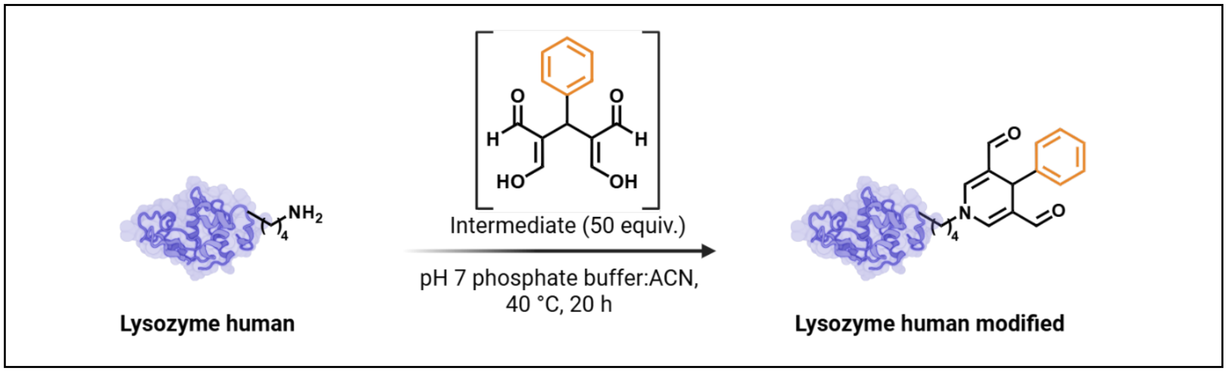

In a one-dram vial, lysozyme human (1 mg, 68.1 nmol) was dissolved in 300 µL of sodium phosphate buffer (100 mM, pH 7) and 100 µL of acetonitrile followed by the addition of the intermediate (50 equiv.) and left to stir at 37 °C for 20 hours. Subsequently, the reaction mixture was passed through Amicon Ultra 3 kDa spin-concentrator and washed with H_2O (5 x 500 µL) to remove the small molecule impurities. The labeled protein was redissolved in 0.1% formic acid in H_2O and analyzed using LC-MS. Modification of MDA-benzaldehyde is confirmed by a mass shift of +196 *m/z*. The conversion was found to be 3% with 1 modification, 40% with 2 modifications, 40% with 3 modifications and 17% with 4 modifications.

Intact MS Spectrum of Starting Lysozyme Human

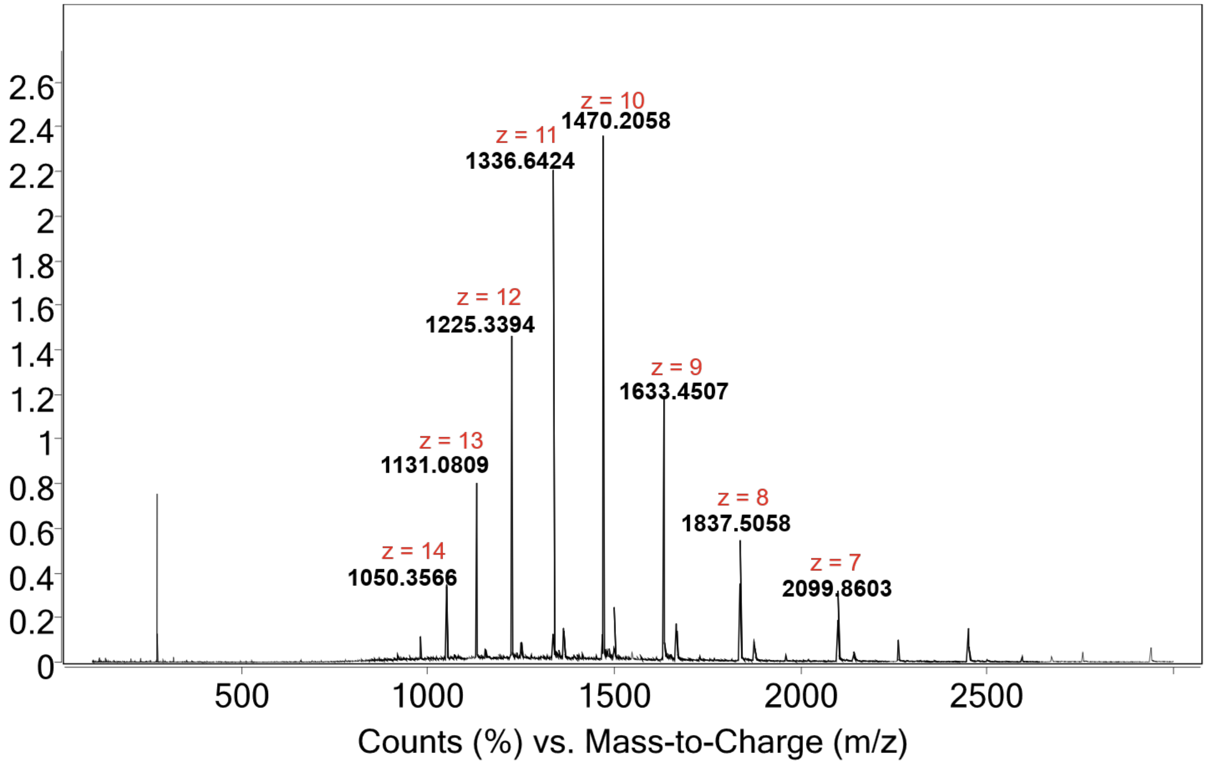

Deconvoluted MS Spectrum of Starting Lysozyme Human

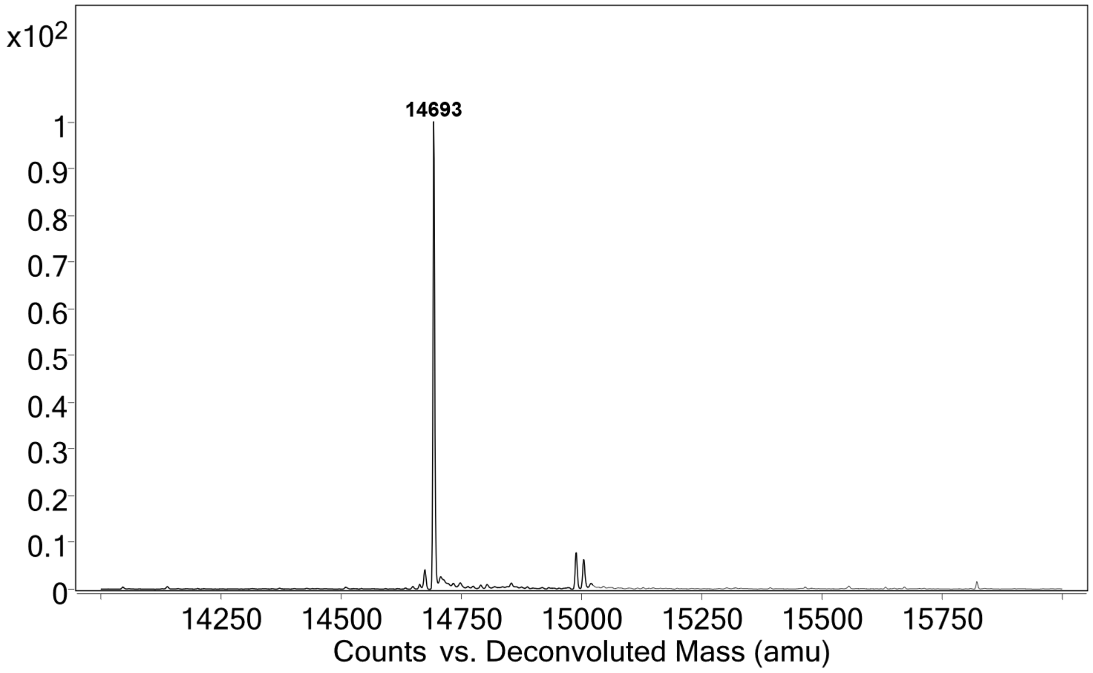

Intact MS Spectrum of Modified Lysozyme Human

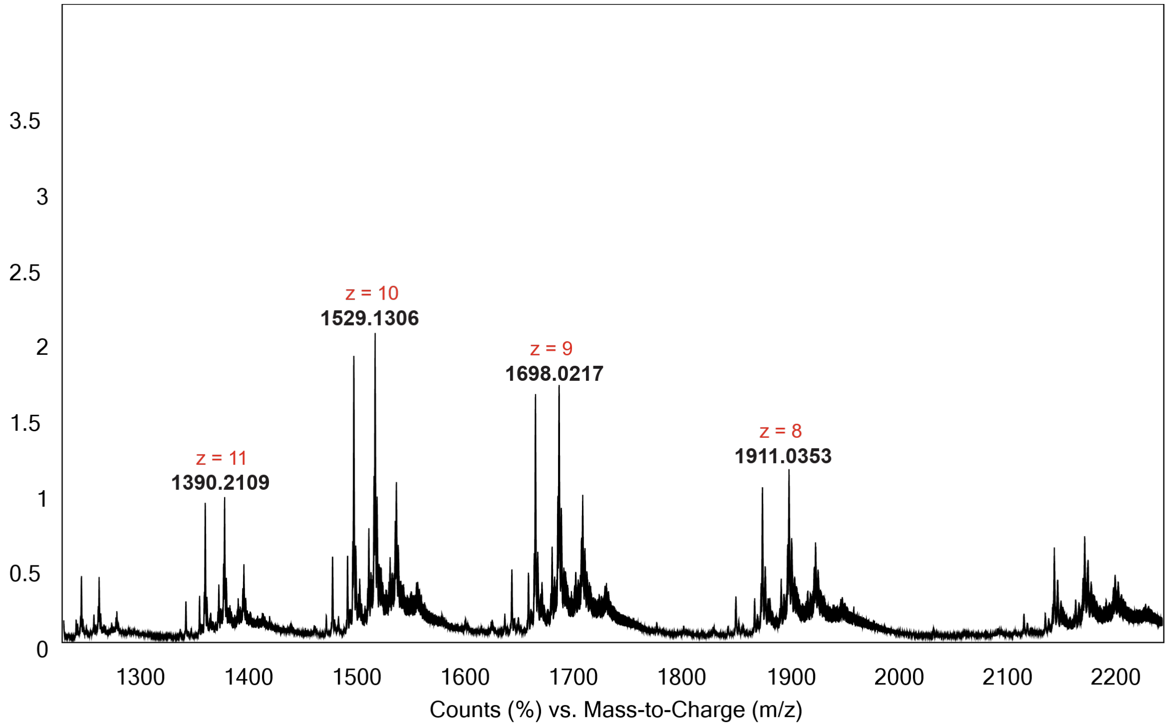

Deconvoluted MS Spectrum of Modified Lysozyme Human

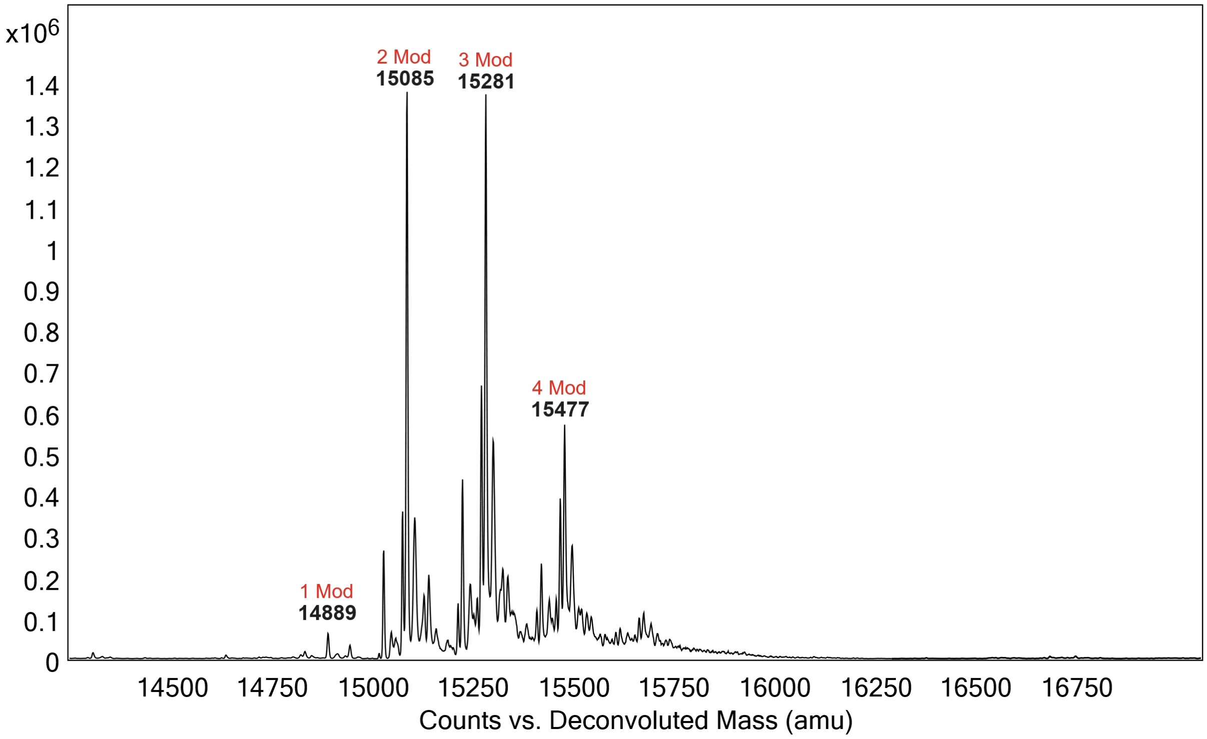

Apo-transferrin.

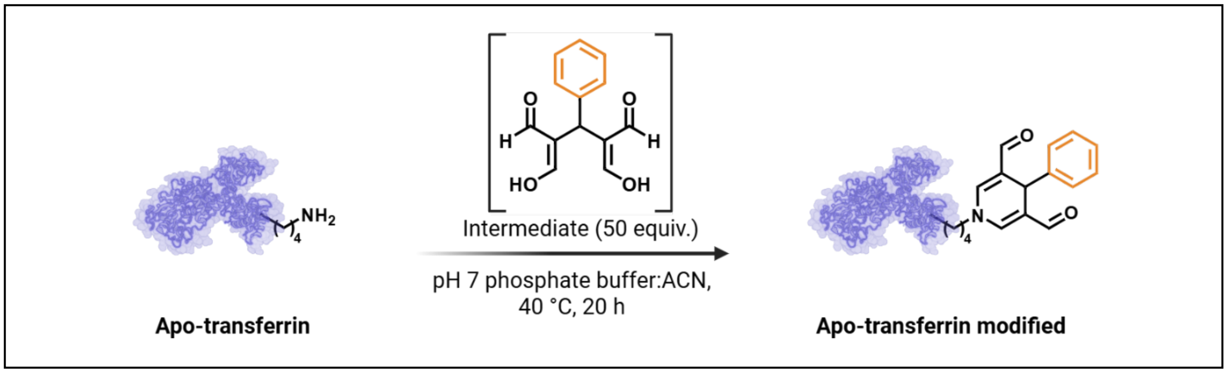

In a one-dram vial, apo-transferrin (1 mg, 12.6 nmol) was dissolved in 300 µL of sodium phosphate buffer (100 mM, pH 7) and 100 µL of acetonitrile followed by the addition of the intermediate (50 equiv.) and left to stir at 37 °C for 20 hours. Subsequently, the reaction mixture was passed through Amicon Ultra 3 kDa spin-concentrator and washed with H_2O (5 x 500 µL) to remove the small molecule impurities. The labeled protein was redissolved in 0.1% formic acid in H_2O and analyzed using LC-MS. Modification of MDA-benzaldehyde is confirmed by a mass shift of +196 *m/z*. The conversion was found to be >95% with 8 modifications.

Intact MS Spectrum of Starting Apo-transferrin

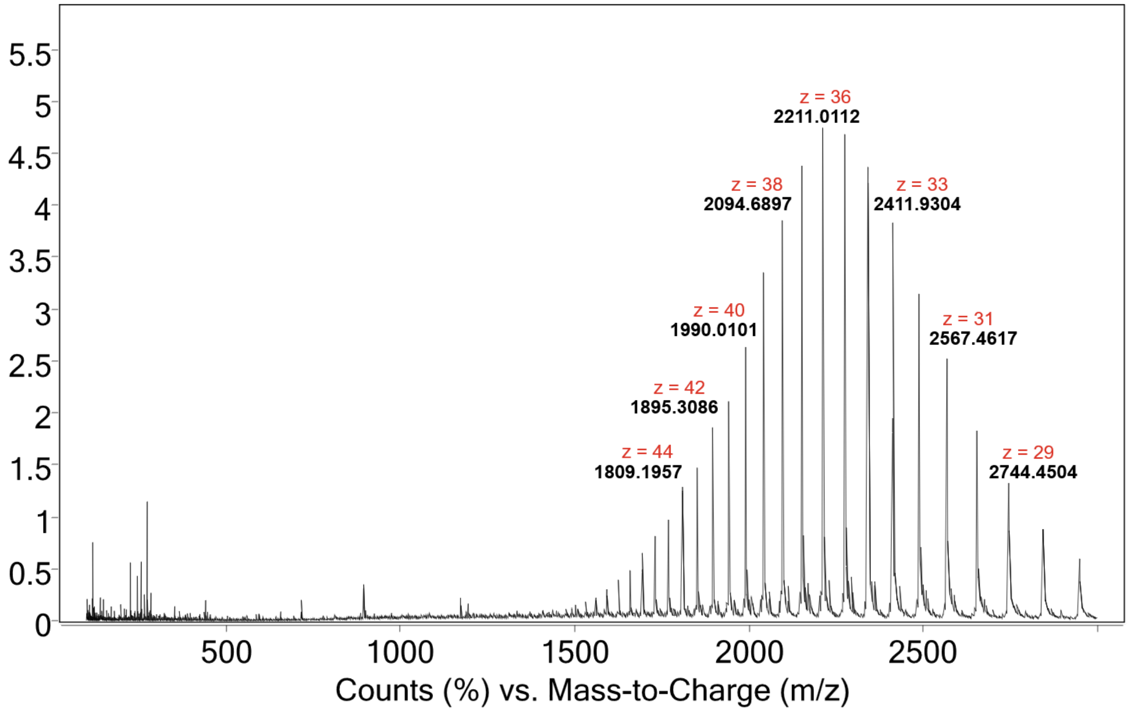

Deconvoluted MS Spectrum of Starting Apo-transferrin

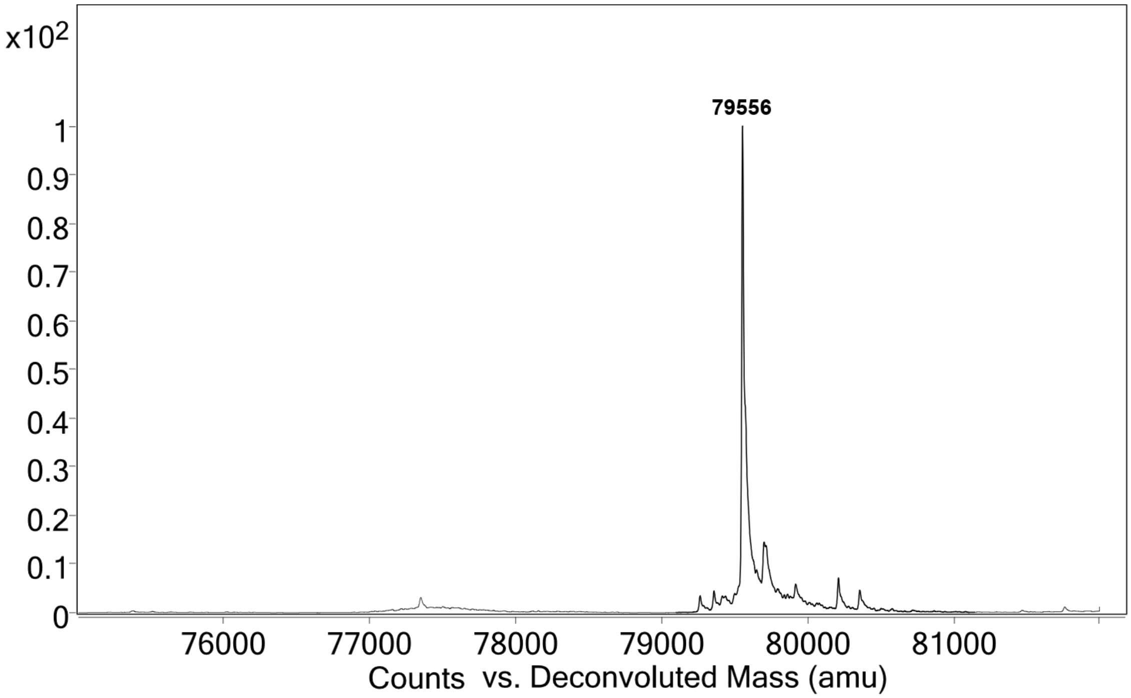

Intact MS Spectrum of Modified Apo-transferrin

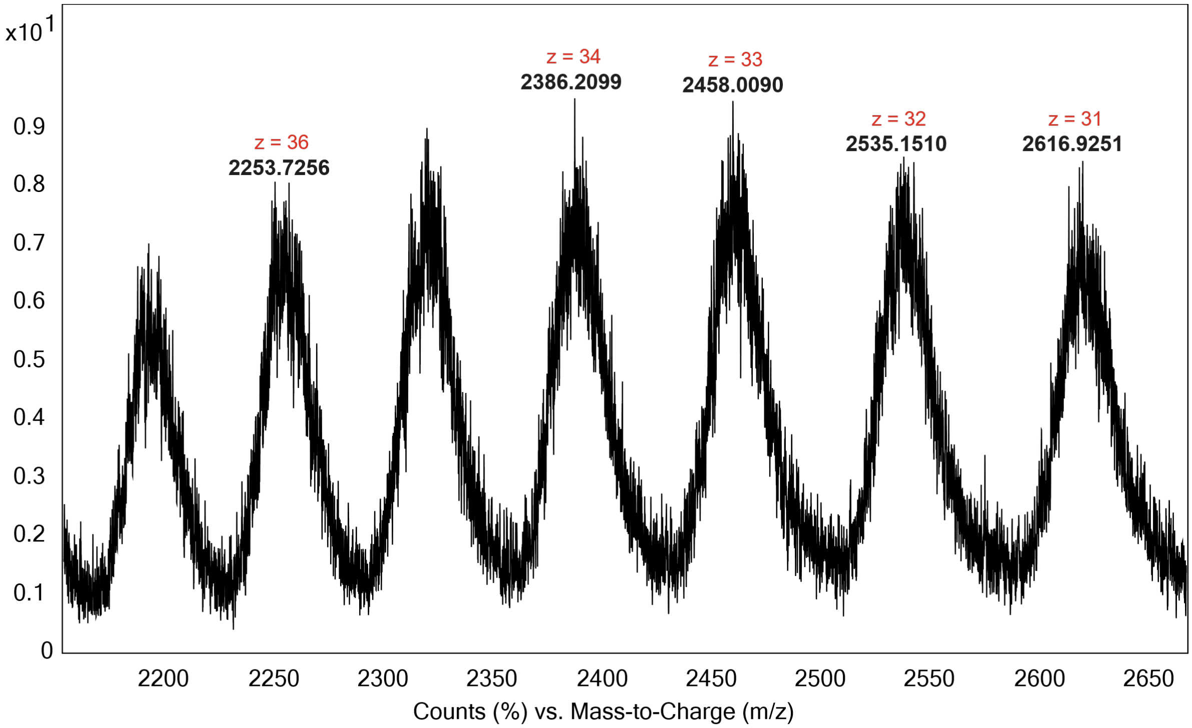

Deconvoluted MS Spectrum of Modified Apo-transferrin

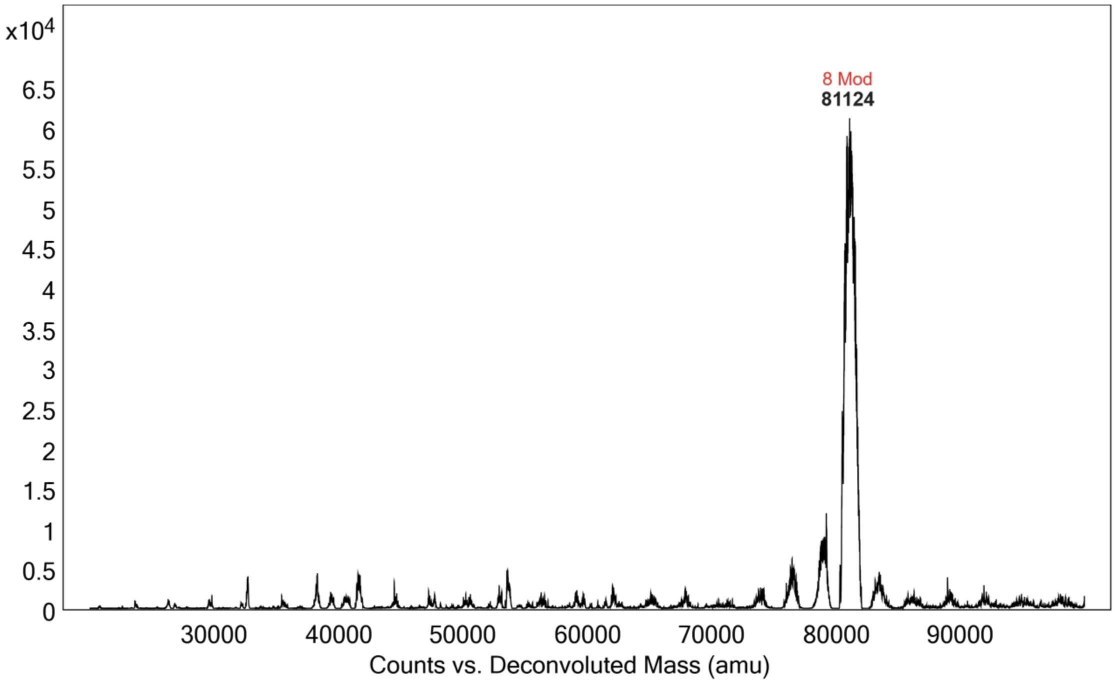

Ubiquitin.

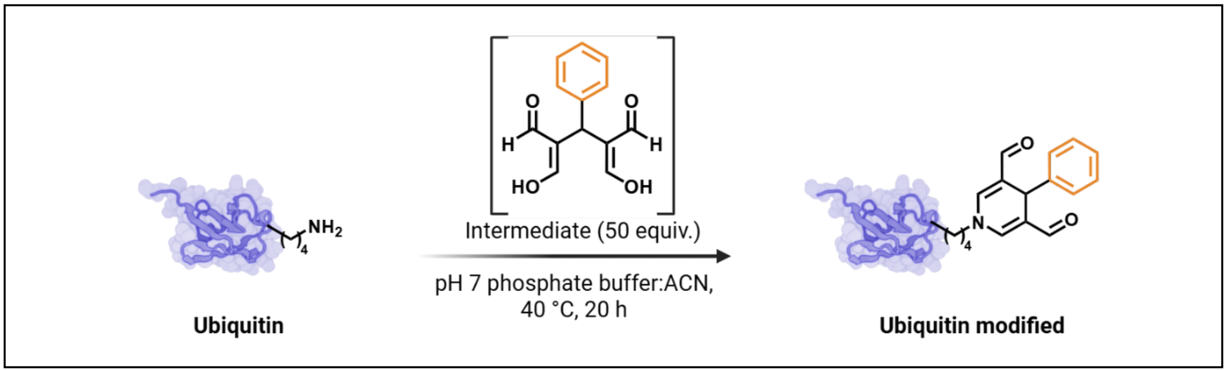

In a one-dram vial, ubiquitin (1 mg, 172.2 nmol) was dissolved in 300 µL of sodium phosphate buffer (100 mM, pH 7) and 100 µL of acetonitrile followed by the addition of the intermediate (50 equiv.) and left to stir at 37 °C for 20 hours. Subsequently, the reaction mixture was passed through Amicon Ultra 3 kDa spin-concentrator and washed with H_2O (5 x 500 µL) to remove the small molecule impurities. The labeled protein was redissolved in 0.1% formic acid in H_2O and analyzed using LC-MS. Modification of MDA-benzaldehyde is confirmed by a mass shift of +196 *m/z*. The conversion was found to be >95% with 4 modifications.

Intact MS Spectrum of Starting Ubiquitin

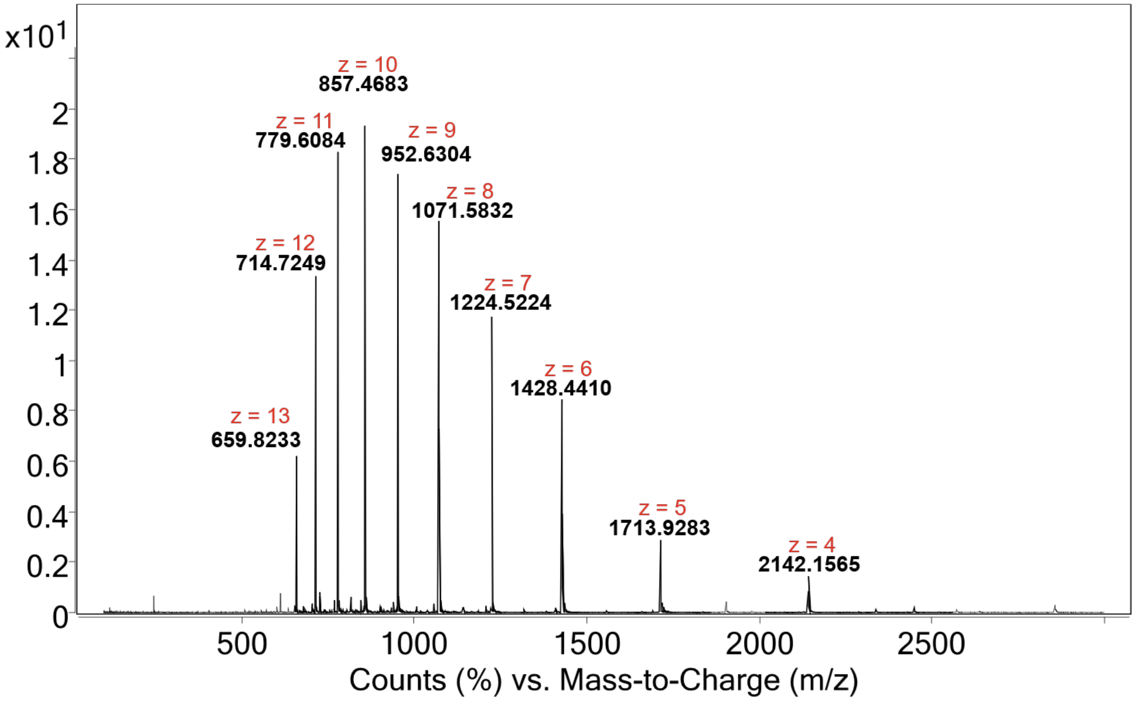

Deconvoluted MS Spectrum of Starting Ubiquitin

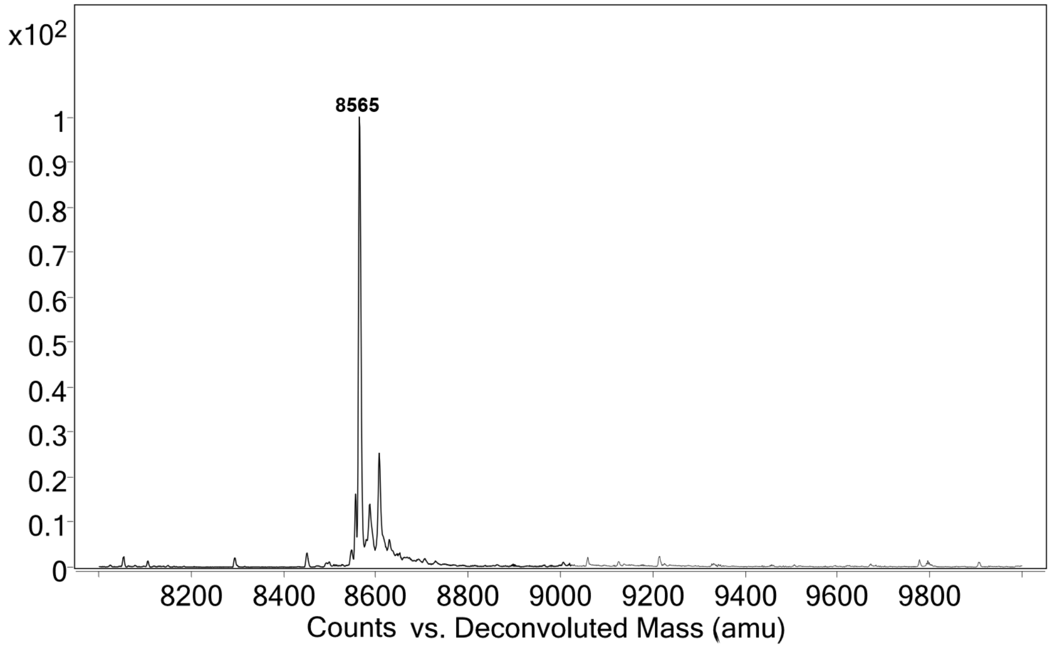

Intact MS Spectrum of Modified Ubiquitin

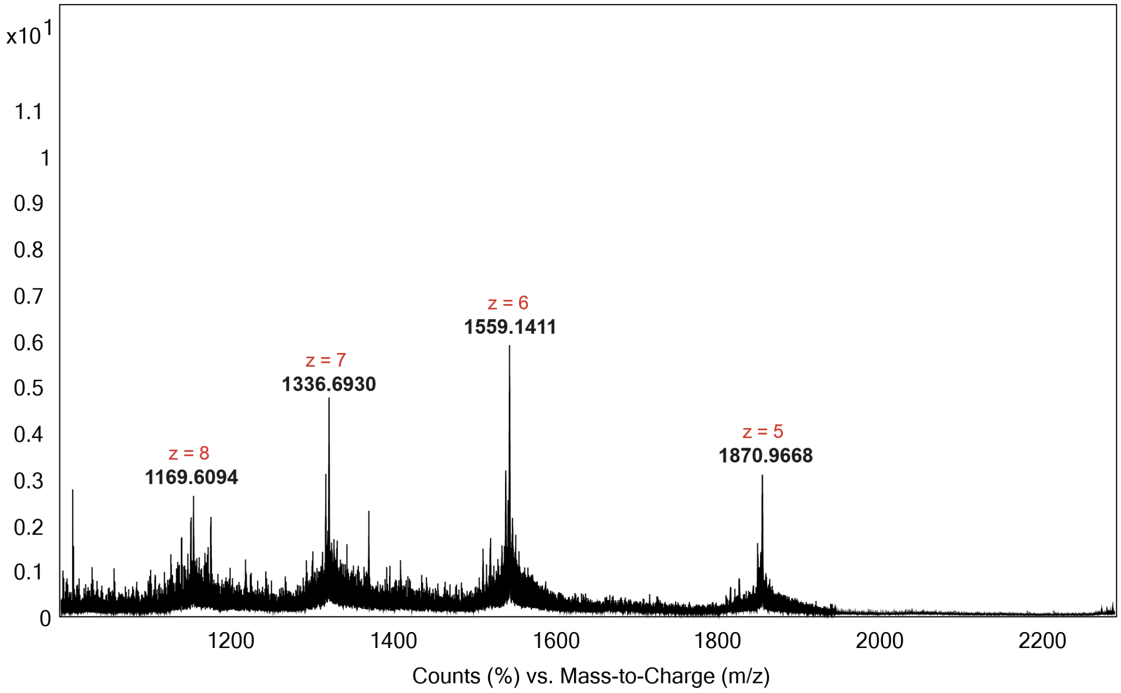

Deconvoluted MS Spectrum of Modified Ubiquitin

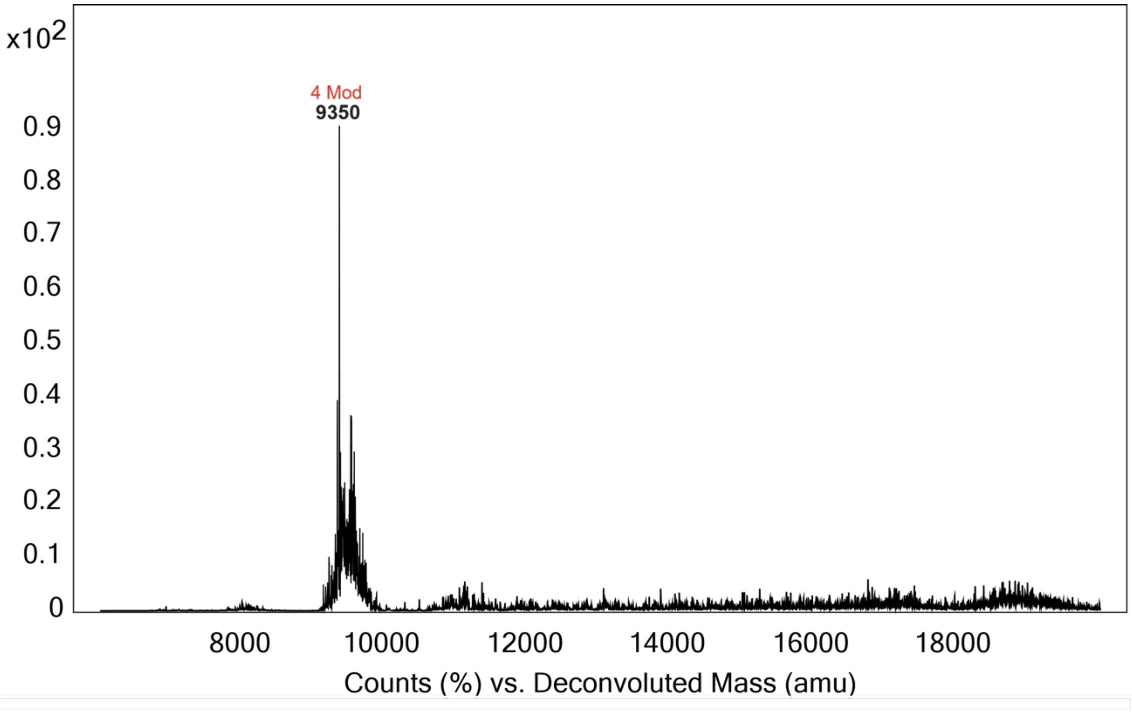

Insulin.

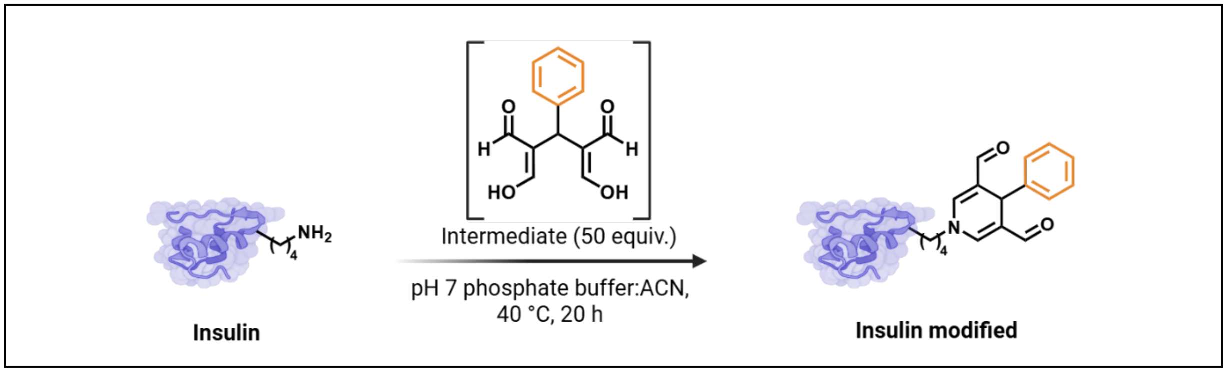

In a one-dram vial, insulin (1 mg, 172.2 nmol) was dissolved in 300 µL of sodium phosphate buffer (100 mM, pH 7) and 100 µL of acetonitrile followed by the addition of the intermediate (50 equiv.) and left to stir at 37 °C for 20 hours. Subsequently, the reaction mixture was passed through Amicon Ultra 3 kDa spin-concentrator and washed with H_2O (5 x 500 µL) to remove the small molecule impurities. The labeled protein was redissolved in 0.1% formic acid in H_2O and analyzed using LC-MS. Modification of MDA-benzaldehyde is confirmed by a mass shift of +196 *m/z*. The conversion was found to be >95% with 3 modifications.

Intact MS Spectrum of Starting Insulin

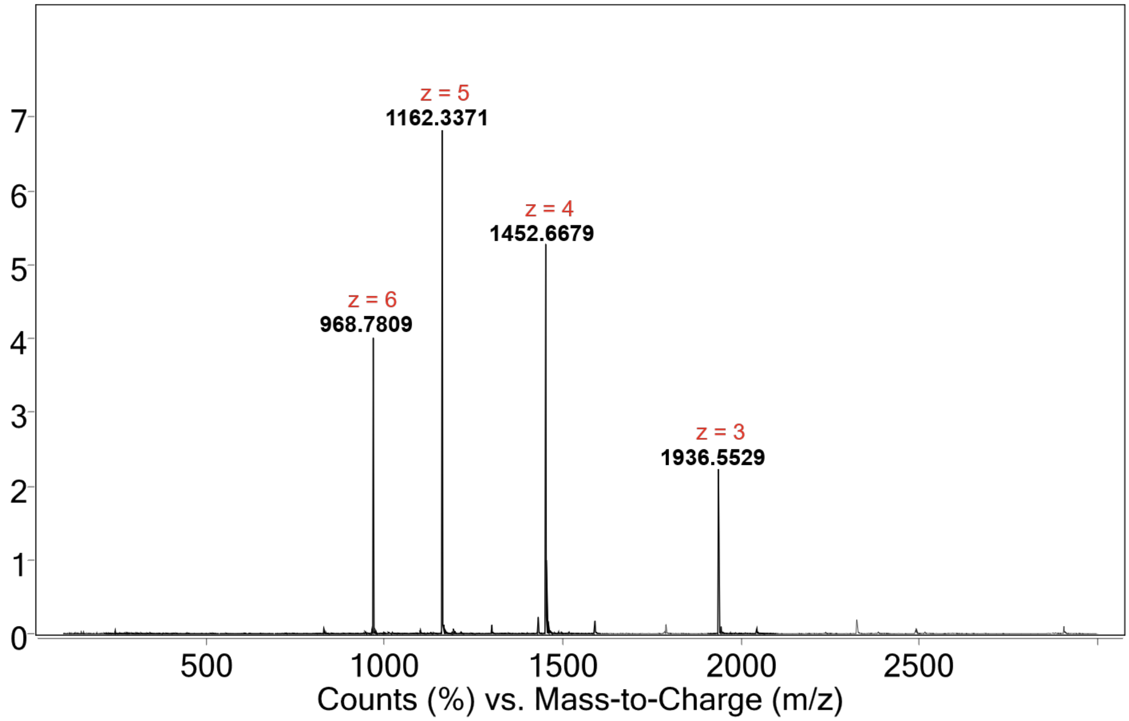

Deconvoluted MS Spectrum of Starting Insulin

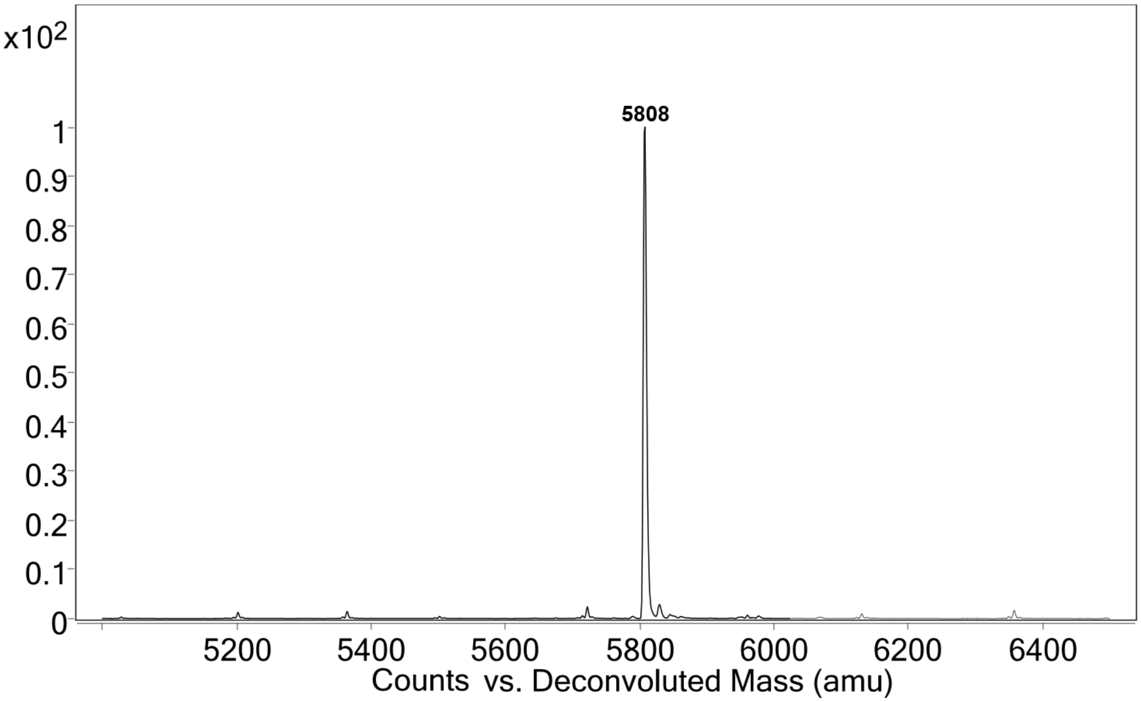

Intact MS Spectrum of Modified Insulin

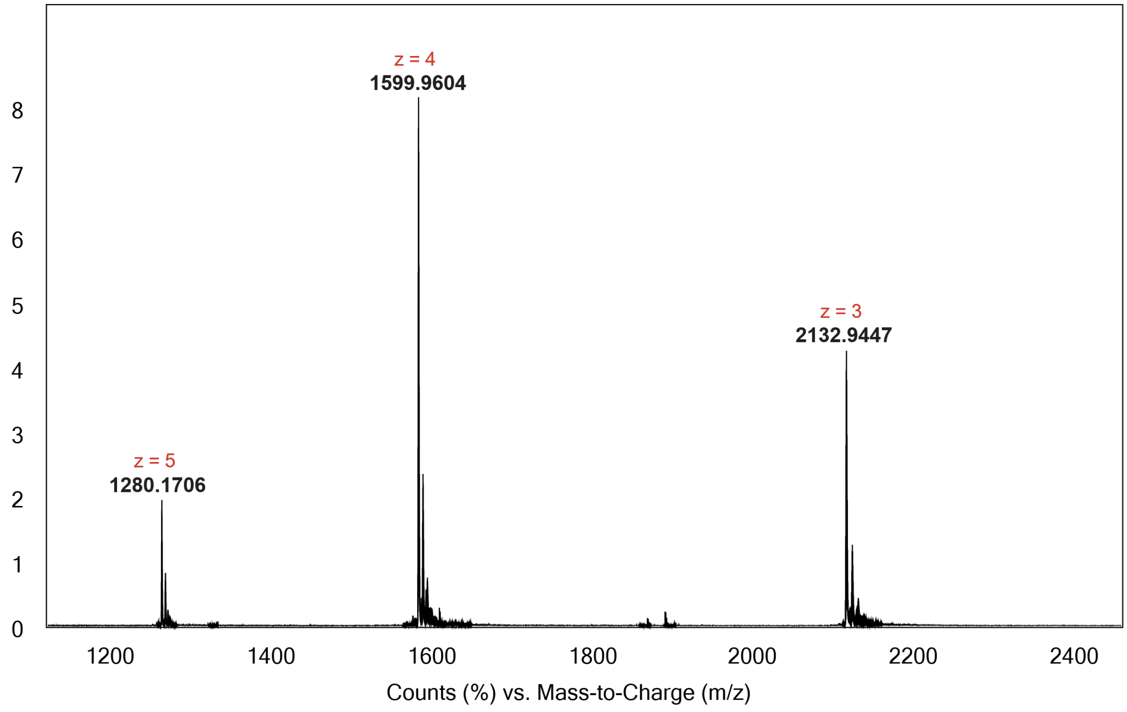

Deconvoluted MS Spectrum of Modified Insulin

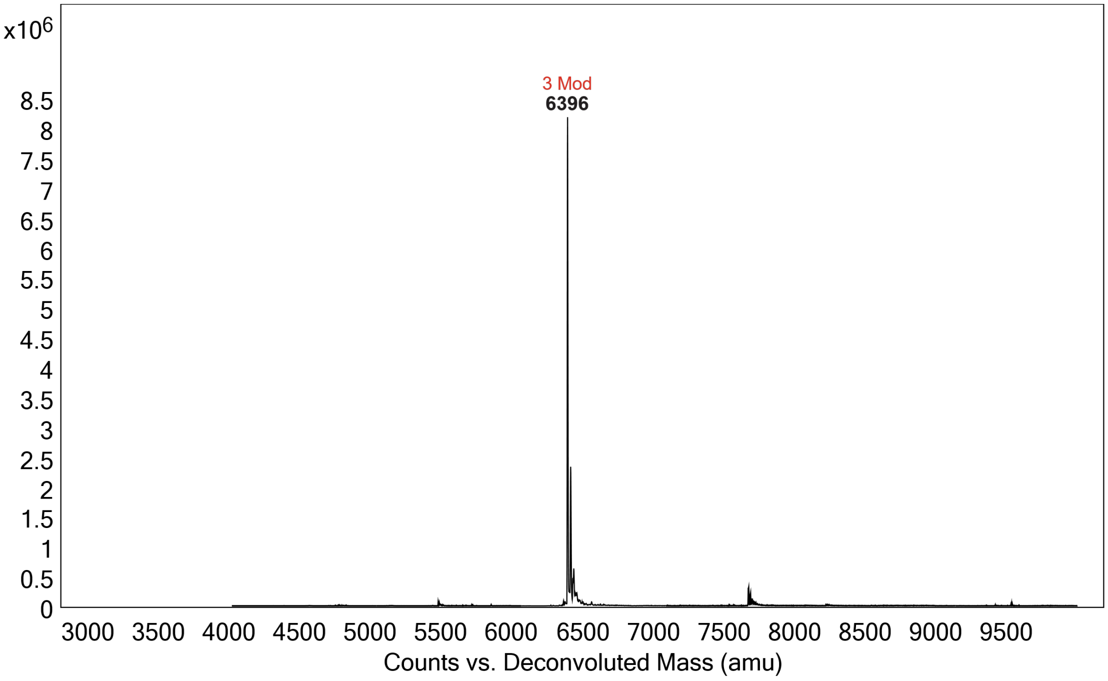

Supplementary Figure 18. Proteins Substrate Scope MDA-MDA Complex.

Lysozyme Human.

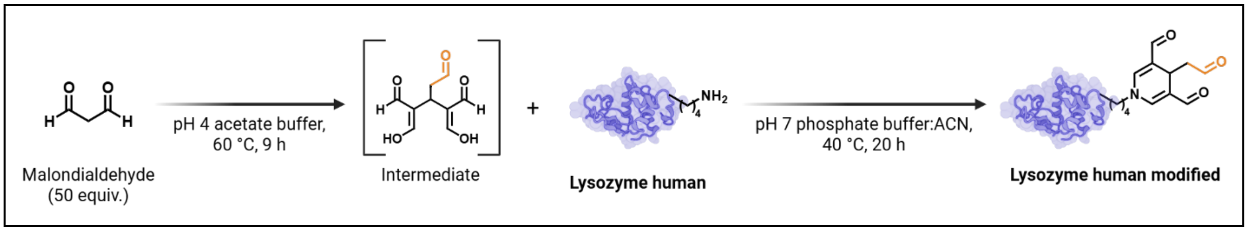

In a one-dram vial, MDA sodium salt (50 equiv.) was dissolved in 100 µL of sodium acetate buffer (100 mM, pH 4) and left to stir at 60 °C for 9 hours. Then, lysozyme human (1 mg, 68.1 nmol) was dissolved in 300 µL of sodium phosphate buffer (100 mM, pH 7) and 100 µL of acetonitrile, added to the reaction mixture and left to stir at 37 °C for 20 hours. Subsequently, the reaction mixture was passed through Amicon Ultra 3 kDa spin-concentrator and washed with H_2O (5 x 500 µL) to remove the small molecule impurities. The labeled protein was redissolved in 0.1% formic acid in H_2O and analyzed using LC-MS. Modification of MDA-MDA is confirmed by a mass shift of +162 *m/z*. The conversion was found to be >95% with 47% with 1 modification, 42% with 2 modifications and 11% with 3 modifications.

Intact MS Spectrum of Starting Lysozyme Human

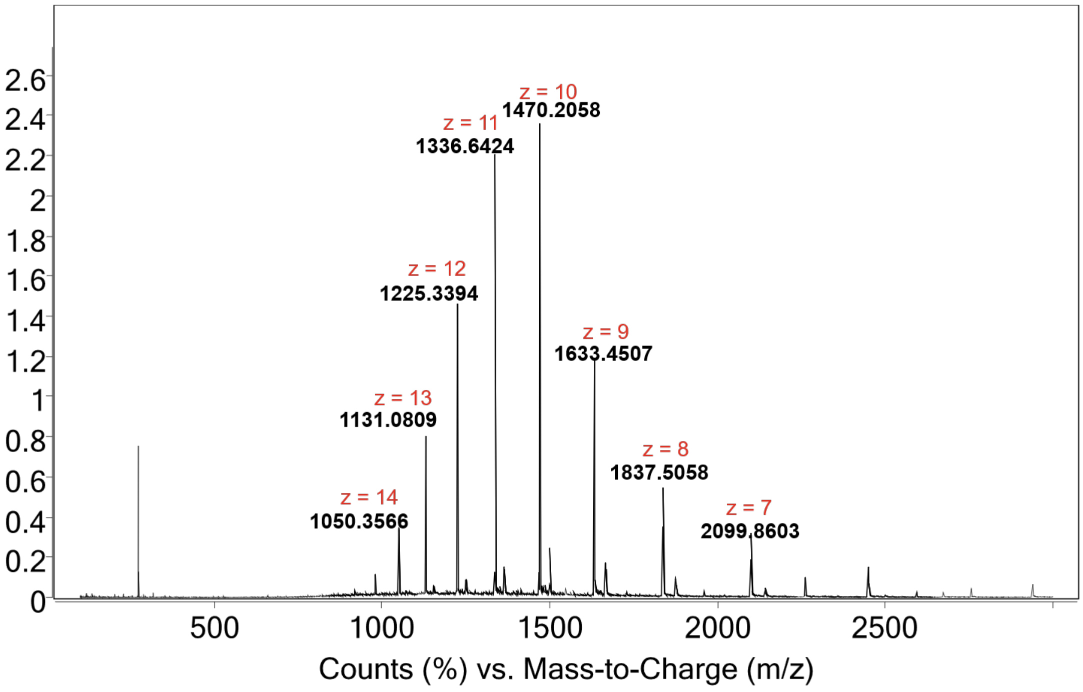

Deconvoluted MS Spectrum of Starting Lysozyme Human

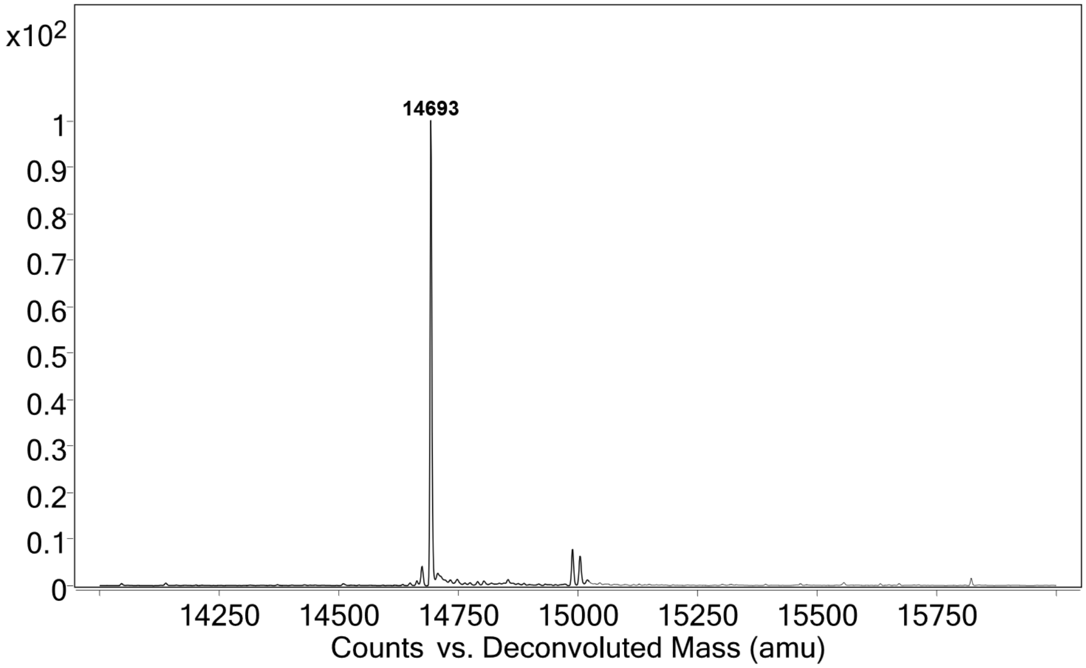

Intact MS Spectrum of Modified Lysozyme Human

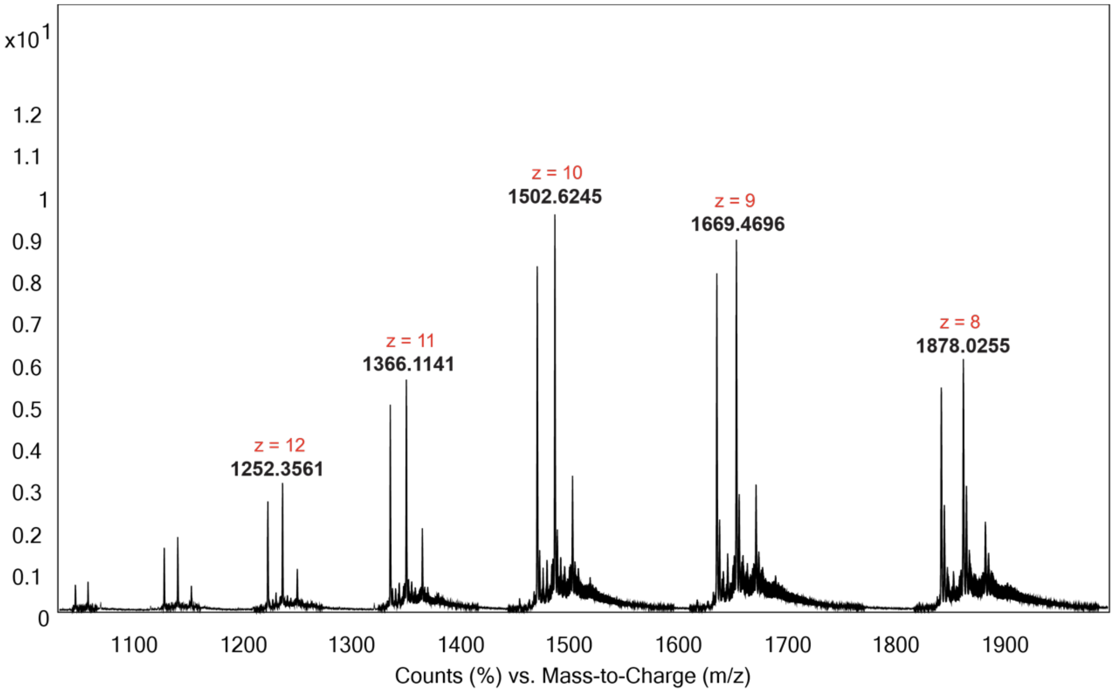

Deconvoluted MS Spectrum of Modified Lysozyme Human

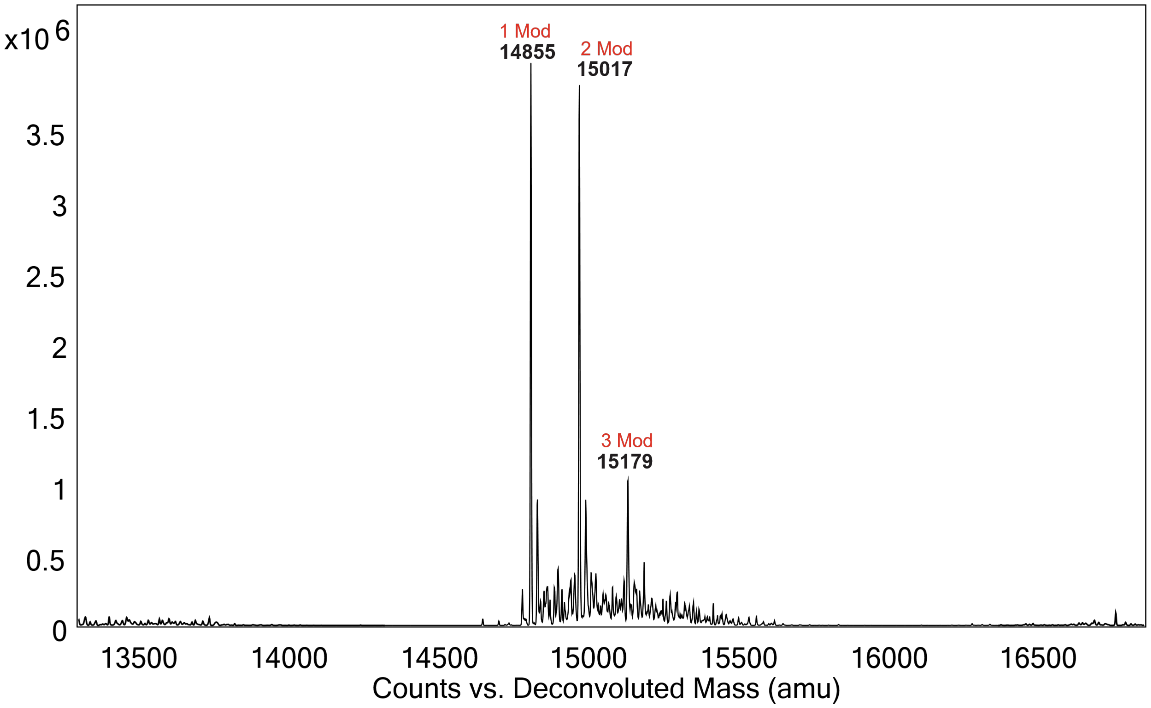

Apo-transferrin.

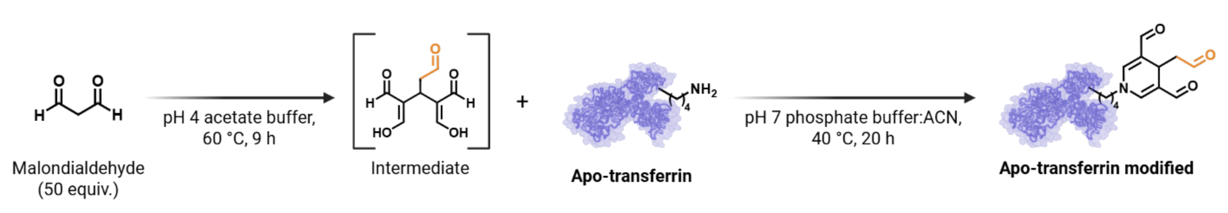

In a one-dram vial, MDA sodium salt (50 equiv.) was dissolved in 100 µL of sodium acetate buffer (100 mM, pH 4) and left to stir at 60 °C for 9 hours. Then, apo-transferrin (1 mg, 12.6 nmol) was dissolved in 300 µL of sodium phosphate buffer (100 mM, pH 7) and 100 µL of acetonitrile, added to the reaction mixture and left to stir at 37 °C for 20 hours. Subsequently, the reaction mixture was passed through Amicon Ultra 3 kDa spin-concentrator and washed with H_2O (5 x 500 µL) to remove the small molecule impurities. The labeled protein was redissolved in 0.1% formic acid in H_2O and analyzed using LC-MS. Modification of MDA-MDA is confirmed by a mass shift of +162 *m/z*. The conversion was found to be >95% with 1 modification.

Intact MS Spectrum of Starting Apo-transferrin

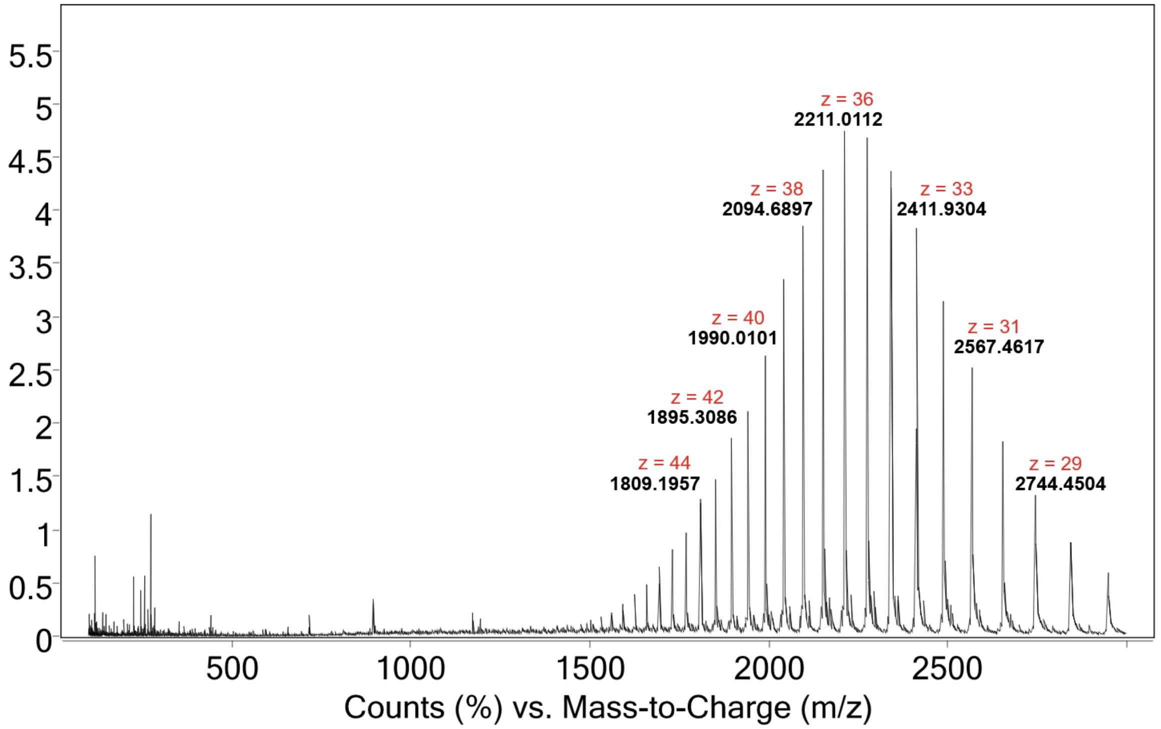

Deconvoluted MS Spectrum of Starting Apo-transferrin

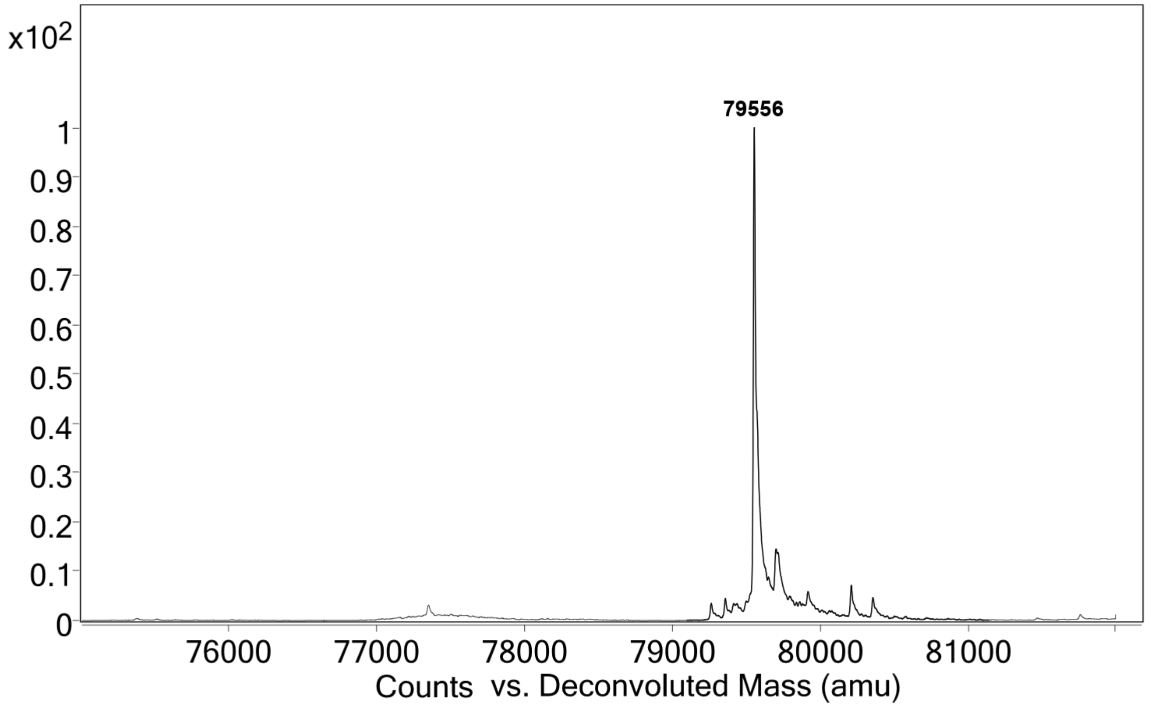

Intact MS Spectrum of Modified Apo-transferrin

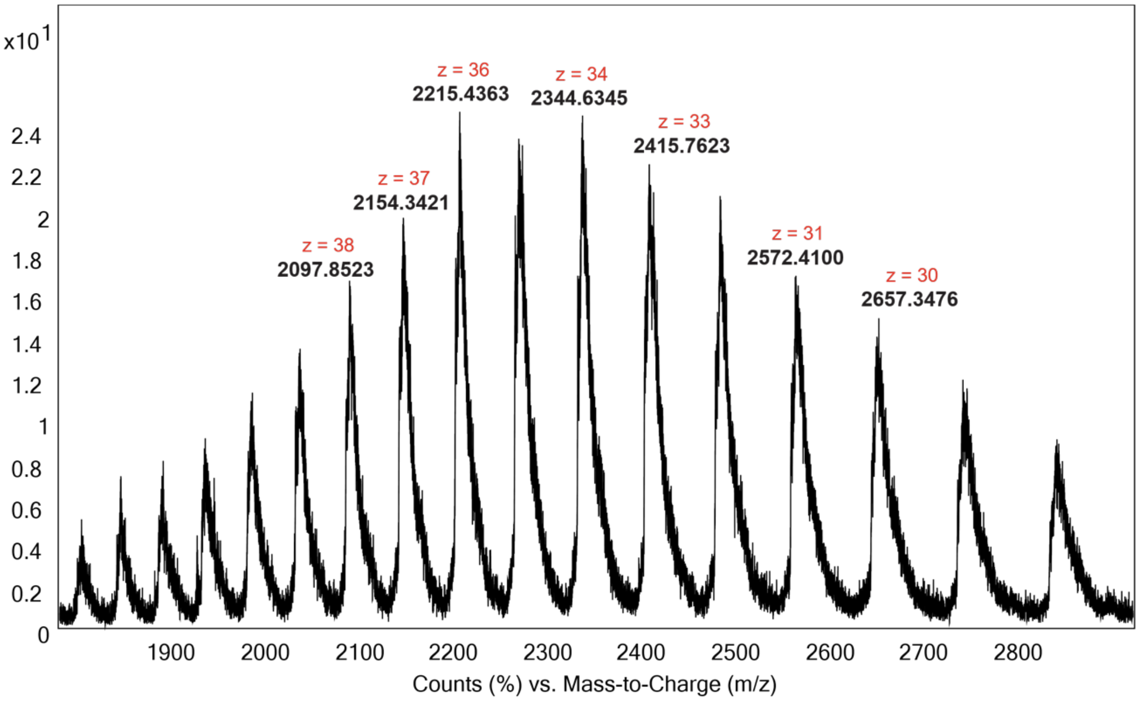

Deconvoluted MS Spectrum of Modified Apo-transferrin

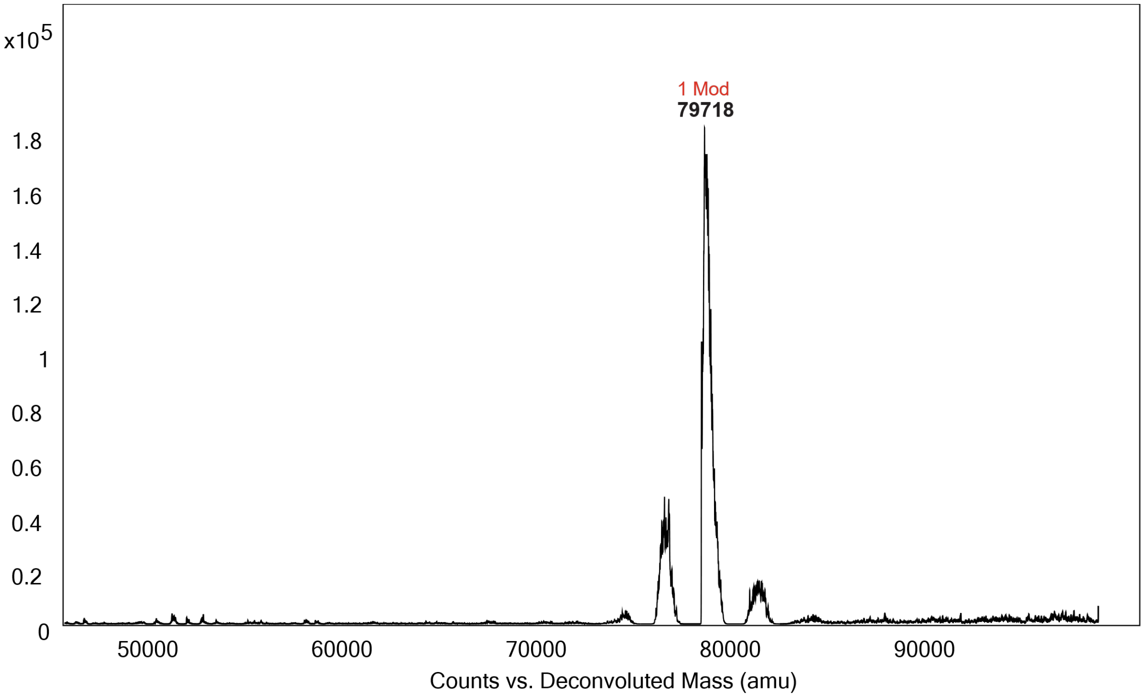

Ubiquitin.

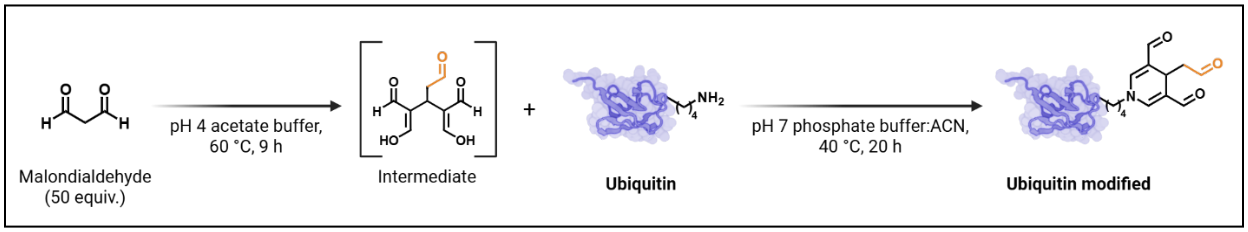

In a one-dram vial, MDA sodium salt (50 equiv.) was dissolved in 100 µL of sodium acetate buffer (100 mM, pH 4) and left to stir at 60 °C for 9 hours. Then, ubiquitin (1 mg, 172.2 nmol) was dissolved in 300 µL of sodium phosphate buffer (100 mM, pH 7) and 100 µL of acetonitrile, added to the reaction mixture and left to stir at 37 °C for 20 hours. Subsequently, the reaction mixture was passed through Amicon Ultra 3 kDa spin-concentrator and washed with H_2O (5 x 500 µL) to remove the small molecule impurities. The labeled protein was redissolved in 0.1% formic acid in H_2O and analyzed using LC-MS. Modification of MDA-MDA is confirmed by a mass shift of +162 *m/z*. The conversion was found to be >95% with 67% with 1 modification and 33% with 2 modifications.

Intact MS Spectrum of Starting Ubiquitin

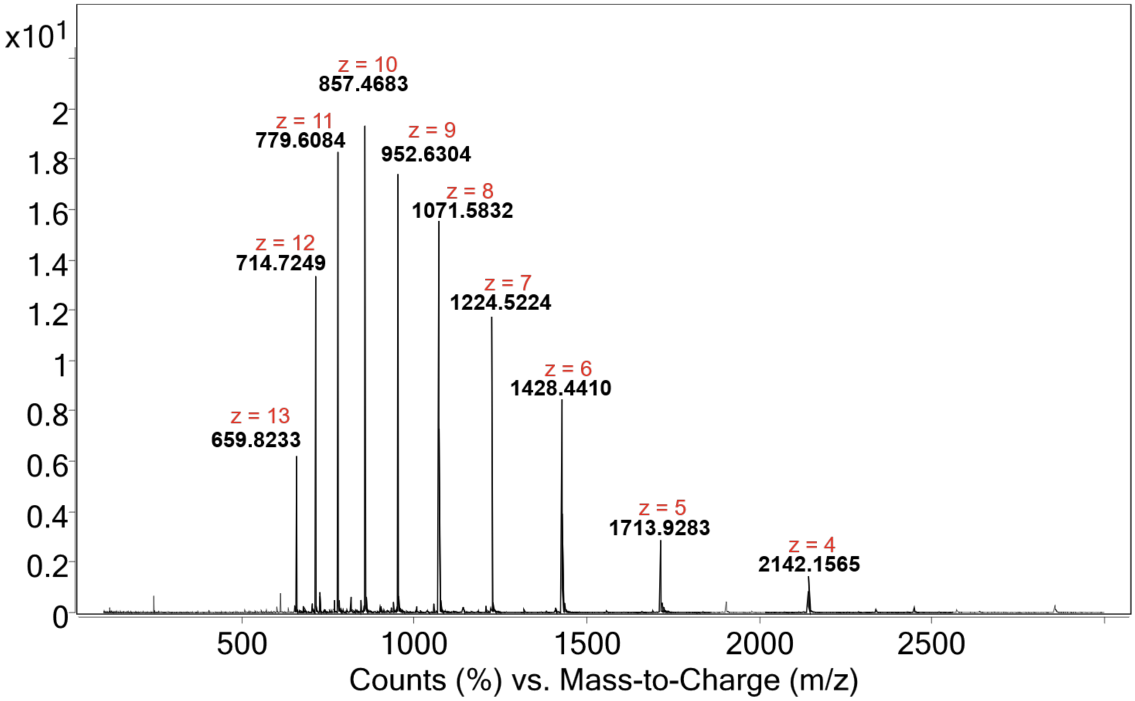

Deconvoluted MS Spectrum of Starting Ubiquitin

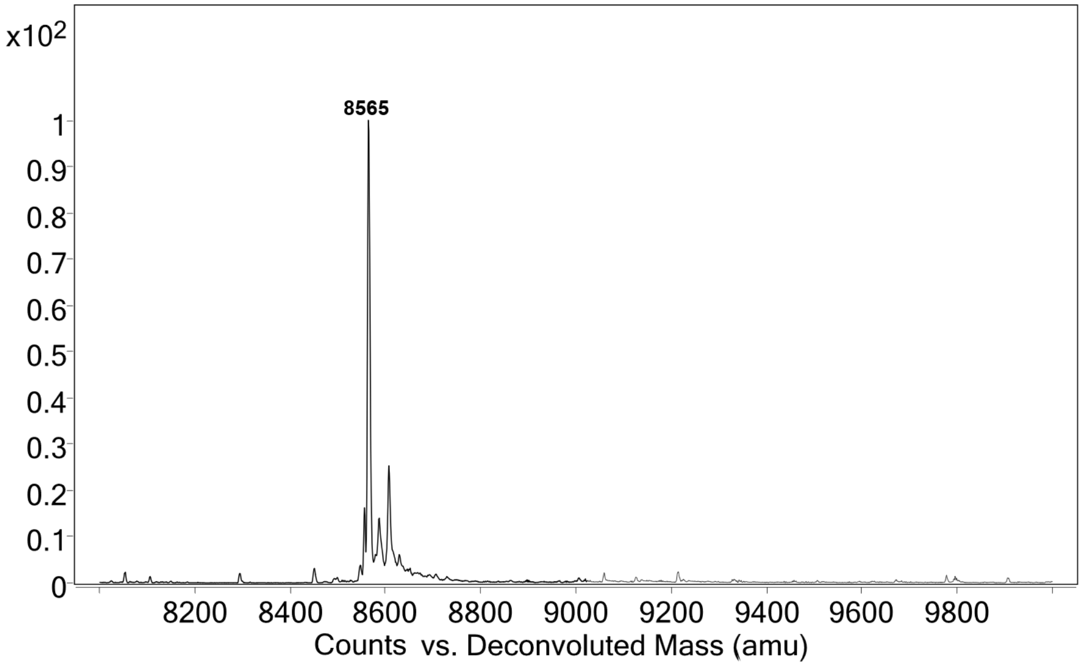

Intact MS Spectrum of Modified Ubiquitin

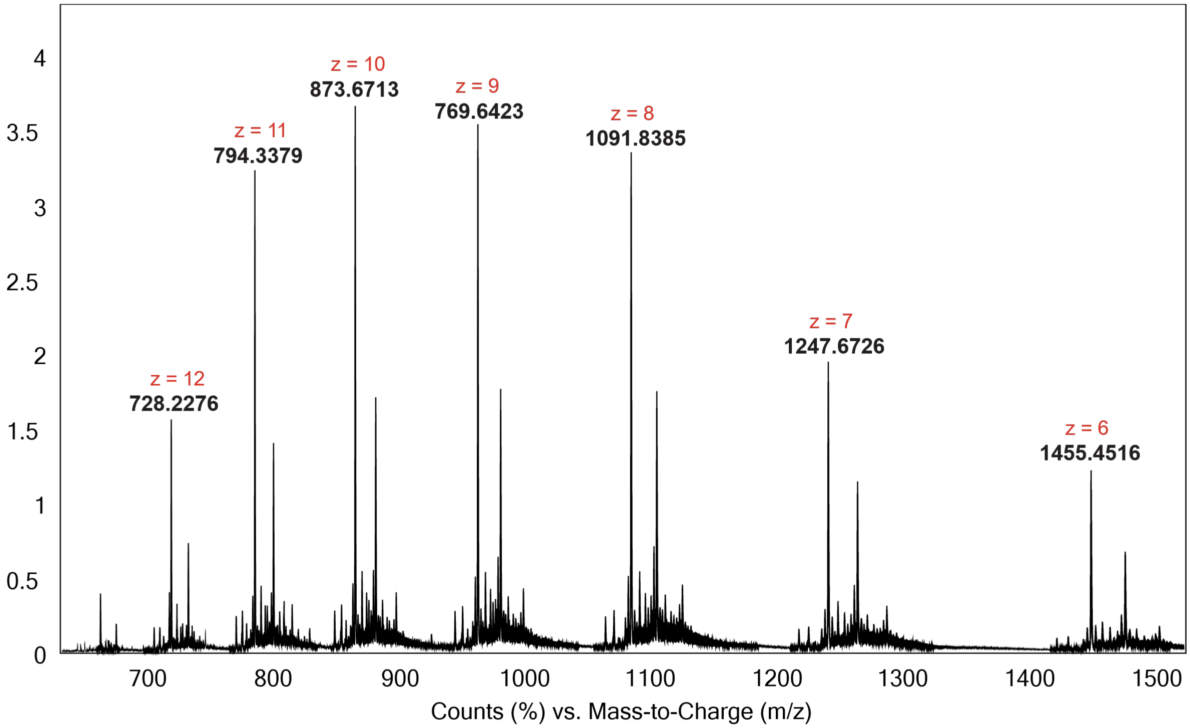

Deconvoluted MS Spectrum of Modified Ubiquitin

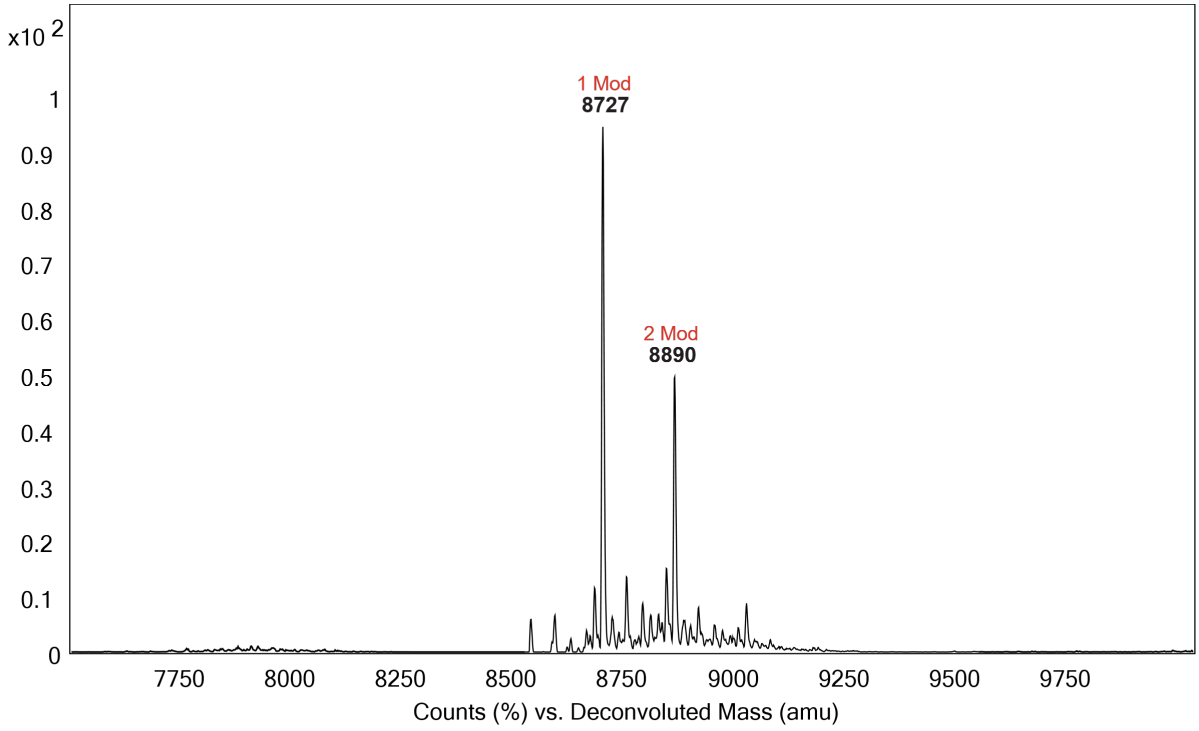

Aprotinin.

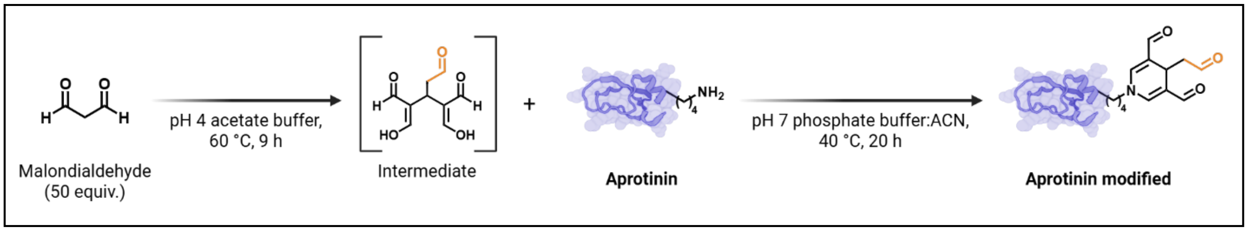

In a one-dram vial, MDA sodium salt (50 equiv.) was dissolved in 100 µL of sodium acetate buffer (100 mM, pH 4) and left to stir at 60 °C for 9 hours. Then, aprotinin (1 mg, 153.6 nmol) was dissolved in 300 µL of sodium phosphate buffer (100 mM, pH 7) and 100 µL of acetonitrile, added to the reaction mixture and left to stir at 37 °C for 20 hours. Subsequently, the reaction mixture was passed through Amicon Ultra 3 kDa spin-concentrator and washed with H_2O (5 x 500 µL) to remove the small molecule impurities. The labeled protein was redissolved in 0.1% formic acid in H_2O and analyzed using LC-MS. Modification of MDA-MDA is confirmed by a mass shift of +162 *m/z*. The conversion was found to be >95% with 2 modifications.

Intact MS Spectrum of Starting Aprotinin

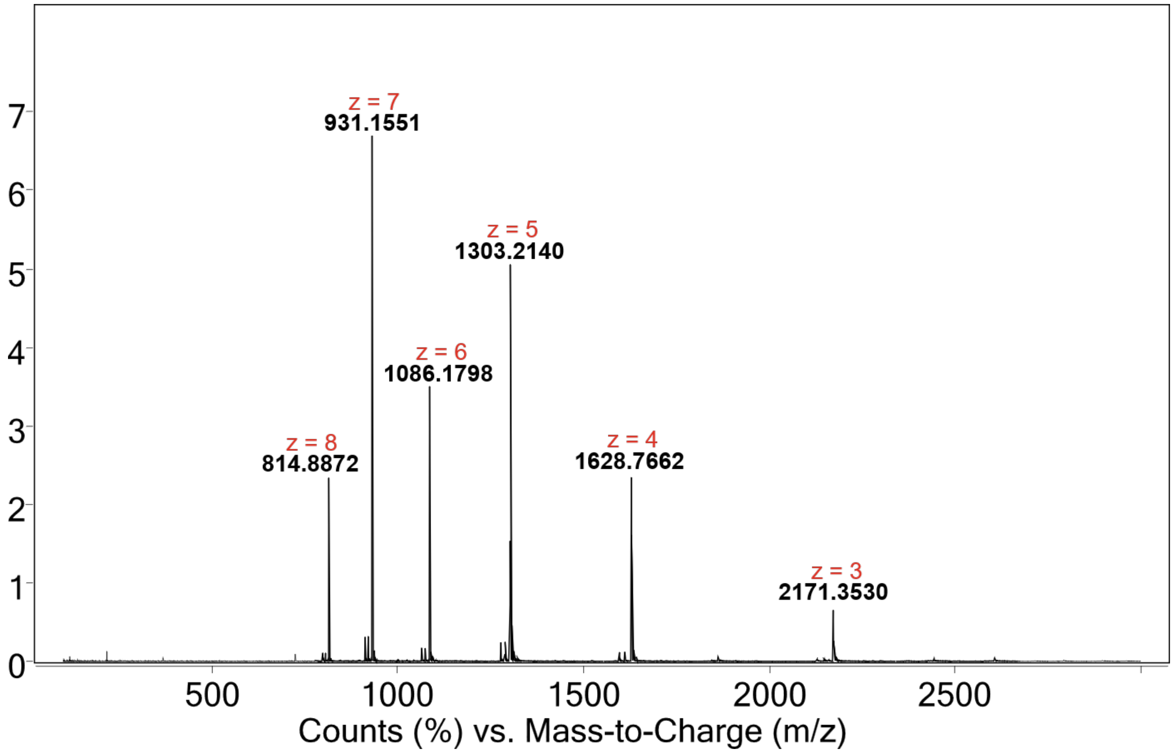

Deconvoluted MS Spectrum of Starting Aprotinin

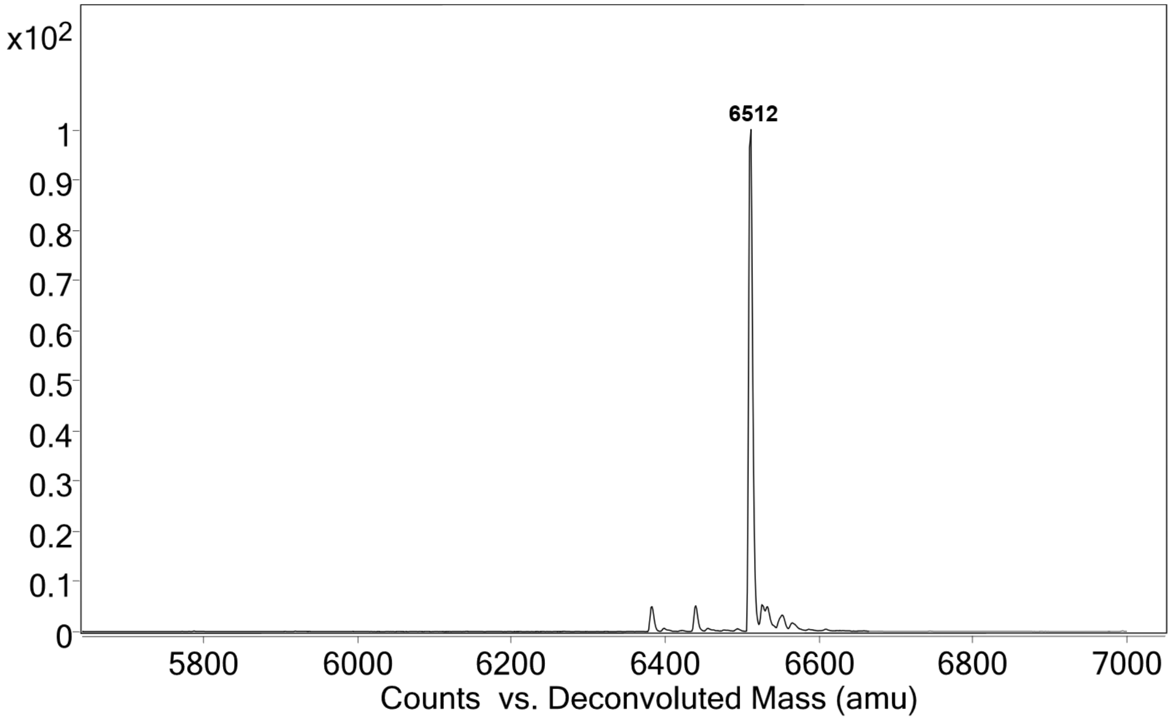

Intact MS Spectrum of Modified Aprotinin

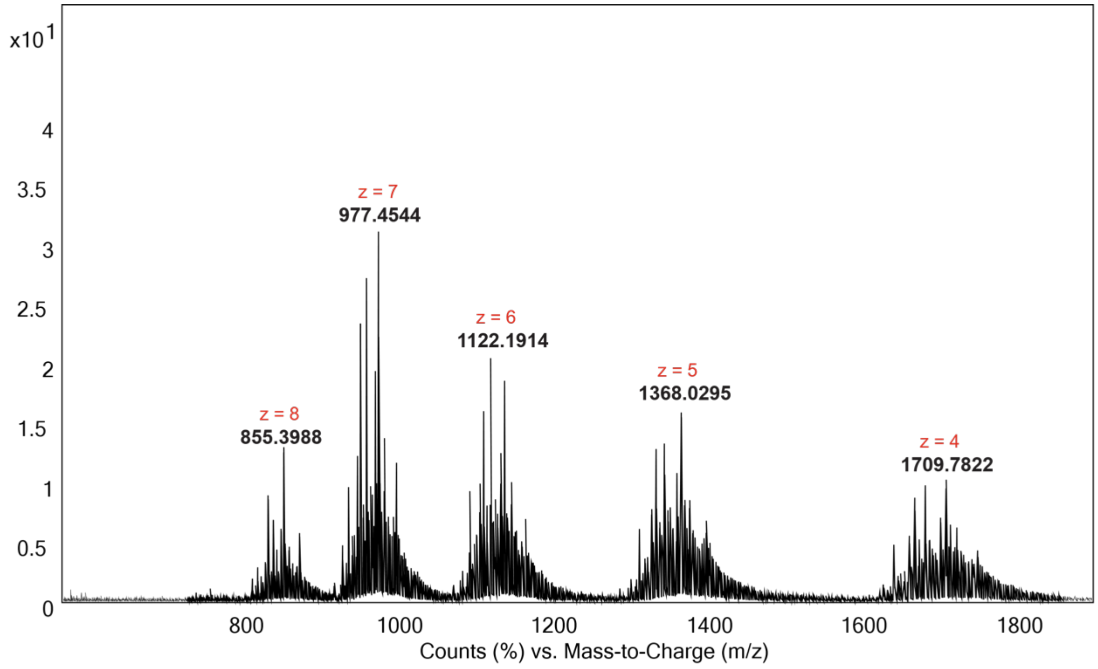

Deconvoluted MS Spectrum of Modified Aprotinin

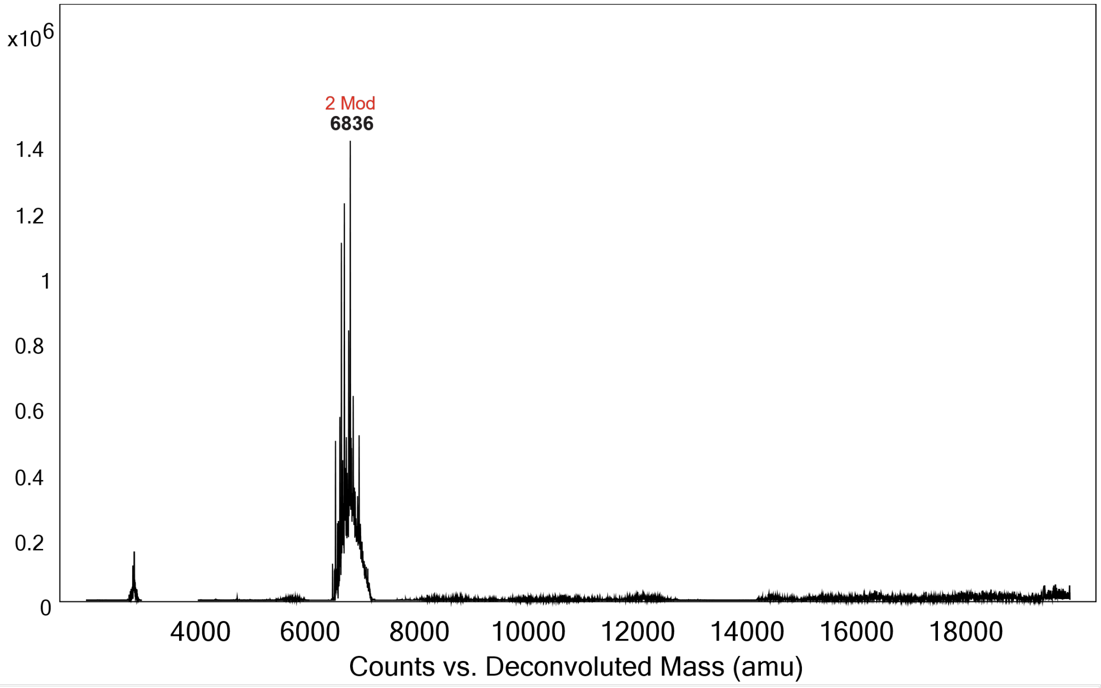

Supplementary Figure 19. Proteomic Analysis.

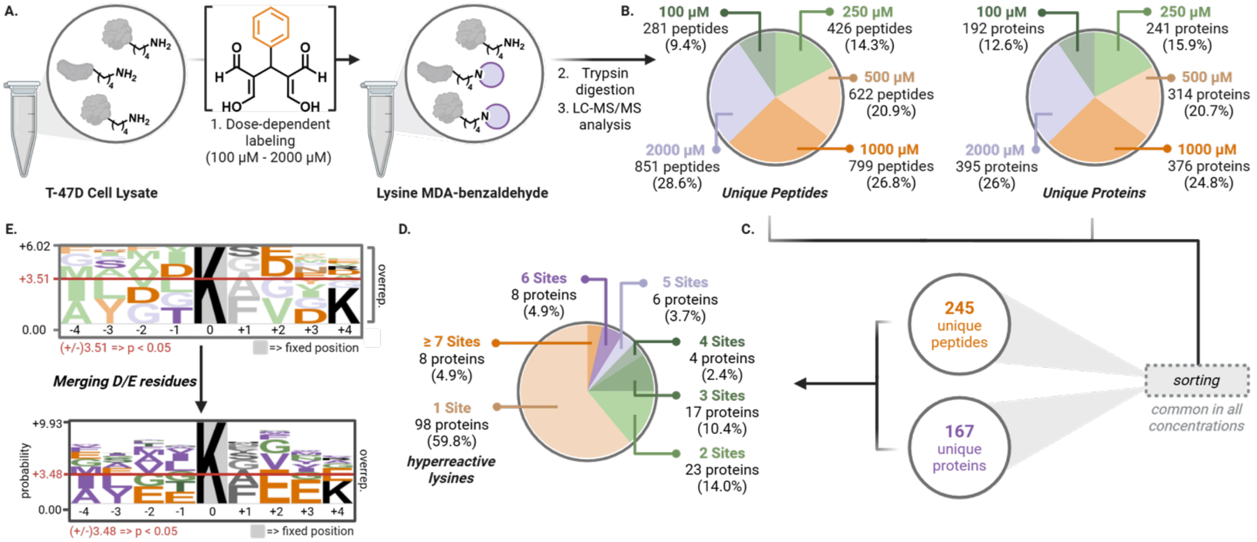

(A) Proteomic workflow for dose-dependent MDA-benzaldehyde labeling (100–2000 µM), performed in duplicate, followed by tryptic digestion and LC-MS/MS analysis. (B) Dose-dependent analysis identified 281, 426, 622, 799, and 851 unique labeled peptides at 100, 250, 500, 1000, and 2000 µM, respectively. Correspondingly, 192, 241, 314, 376, and 395 unique proteins were captured at these concentrations. (C) Filtering for peptides and proteins consistently detected across all five concentrations yielded 245 unique peptides and 167 unique proteins. (D) Among the 167 common proteins, the majority (59.8%, 98 proteins) contained a single modified lysine residue, consistent with strong site selectivity governed by the local lysine microenvironment. (E) Sequence motif analysis of the 245 unique peptides shared across all concentrations revealed pronounced enrichment of acidic residues (Asp/Glu) at the +2 position.

Analysis of Lysate Stability via SDS-PAGE.

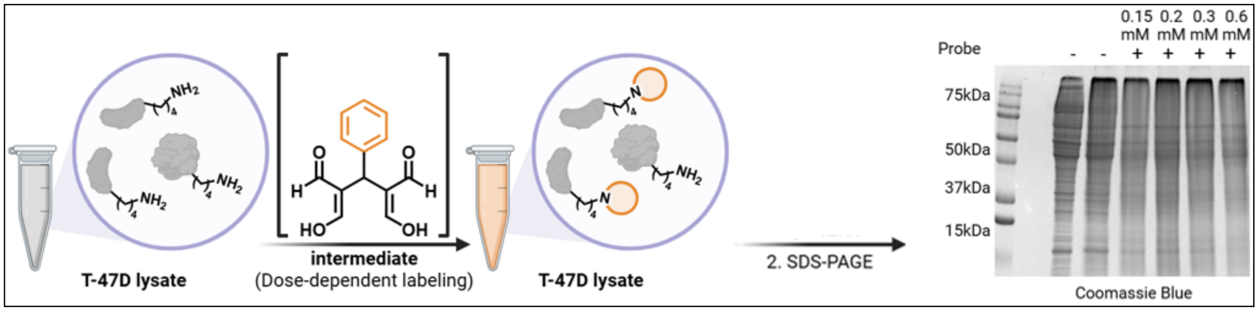

In a one-dram vial, T-47D lysate (100 µg) was dissolved in 300 µL of sodium phosphate buffer (100 mM, pH 7) and 100 µL of acetonitrile. Then, the benzaldehyde intermediate was added (150 µM – 600 µM) and the reaction mixture was left to stir at 37 °C for 20 hours. The reaction was filtered with a 3kDa filter, followed by analysis with coomassie blue staining. Samples were loaded on a Novex WedgeWell 4-20% Tris-Glycine gel. Gel was run in Tris-glycine running buffer at 180V. The gel was then stained with coomassie brilliant blue for 1 h and destained overnight. Uncropped gel data is attached as source data.

Free N-terminus with Benzaldehyde Intermediate.

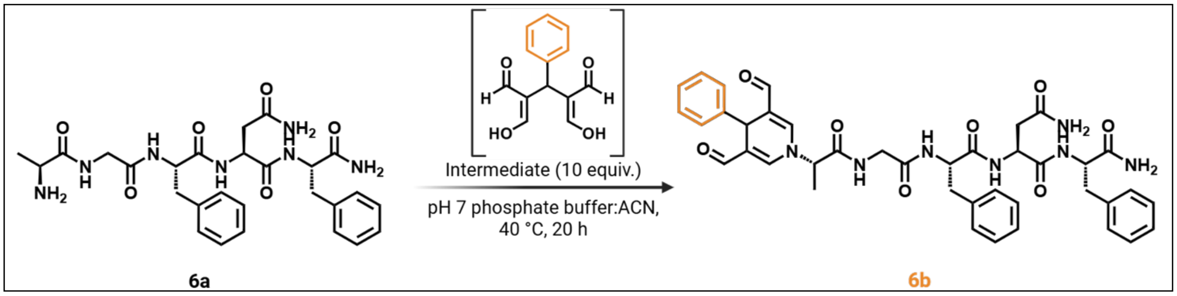

In a one-dram vial, peptide **6a** (H_2N-AGFNF-$CONH_2$) (1.0 mg, 0.002 mmol) was dissolved in 300 µL of sodium phosphate buffer (100 mM, pH 7) and 100 µL of acetonitrile followed by the addition of pre-formed benzaldehyde intermediate (10 equiv.) and left to stir at 37 °C for 20 hours. Subsequently, the reaction mixture was injected into the HPLC for determining the % conversion of peptide **6a** to the labeled peptide **6b** and its mass confirmed with LC-MS. HPLC analysis was carried out utilizing **HPLC Method A** at detection wavelength 220 nm. The mass of the product was confirmed with LC-MS. The conversion was determined to be >95% of **6b**.

H_2N-AGFNF-$CONH_2$ ($C_{27}H_{35}N_7O_6$) peptide 6a. LCMS: 554.2720 *m/z* (calcd $[M+H]^+$ = 554.2722) (HPLC analysis at 220 nm). Retention time in HPLC: 8.5 min

Reaction Mixture:

H_2N-AGFNF-$CONH_2$ ($C_{40}H_{43}N_7O_8$) peptide 6b. LCMS: 750.3240 *m/z* (calcd $[M+H]^+$ = 750.3246) (HPLC analysis at 220 nm). Retention time in HPLC: 15.2 min. (>95%)

HPLC of H_2N-AGFNF-$CONH_2$ Starting Peptide at 220 nm

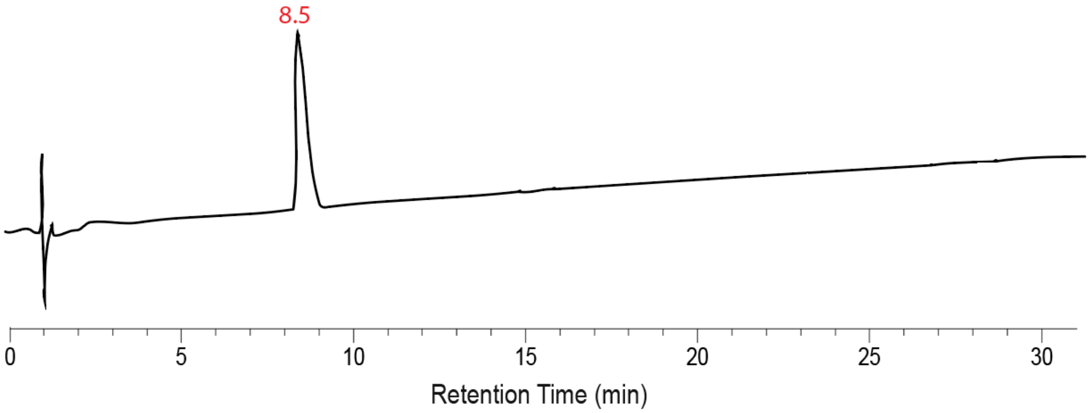

HRMS Trace of Peak at 8.5 min

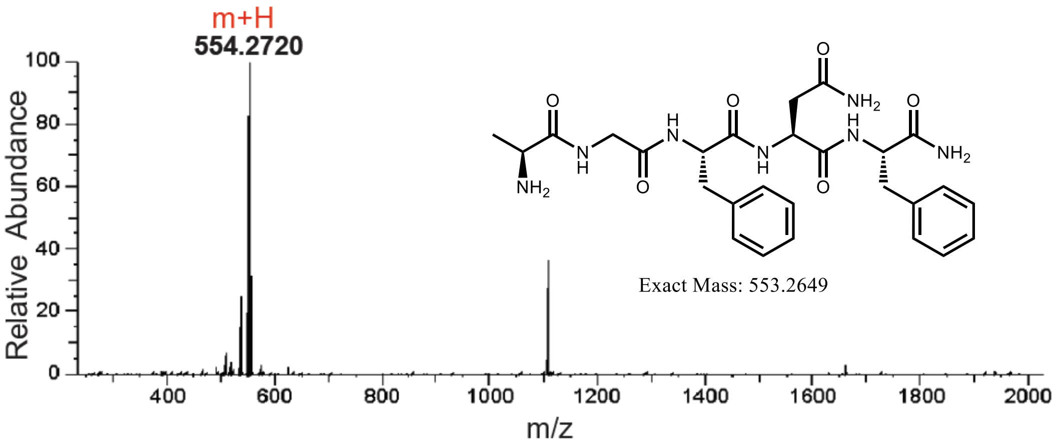

HPLC of H_2N-AGFNF-$CONH_2$ Reaction Mixture at 220 nm

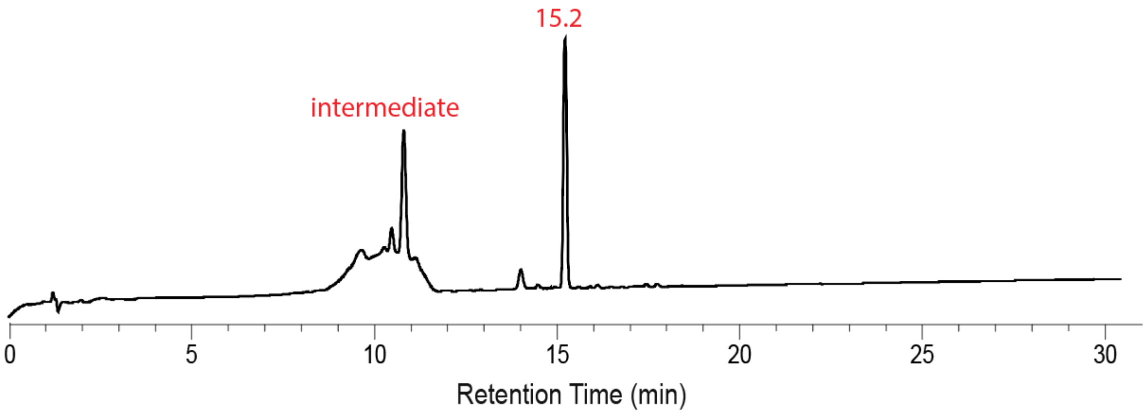

HRMS Trace of Peak at 15.2 min

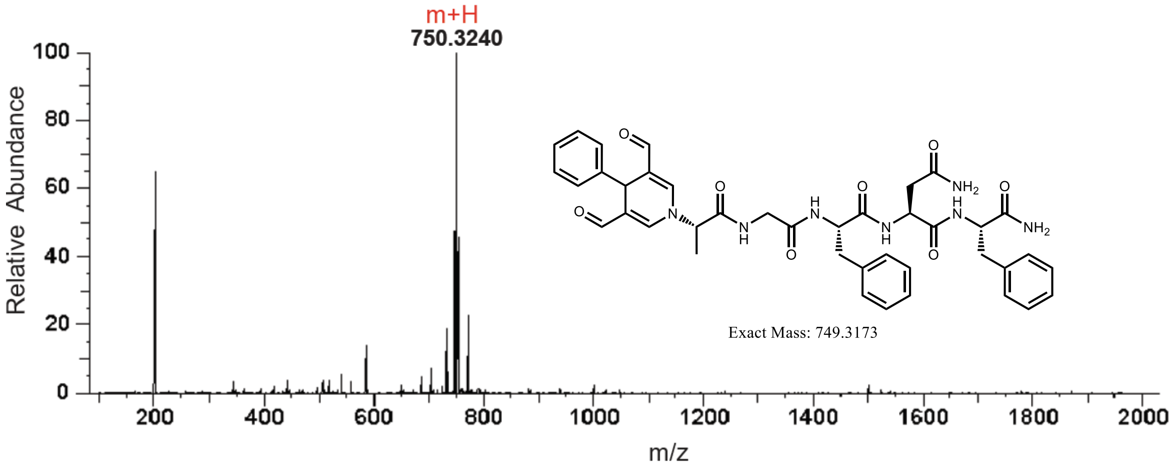

Comparison of NHS-ester-alkyne labeling workflows and chemoselectivity outcomes.

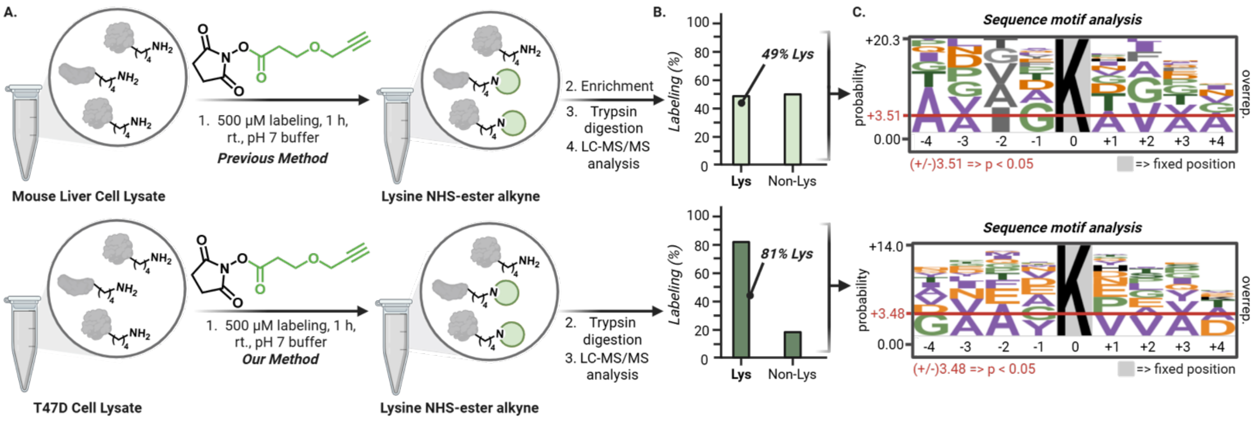

(A) Two proteomic workflows for NHS ester alkyne labeling (500 µM): the previously reported protocol and our implementation. In our workflow, labeling reactions were performed in duplicate, followed by tryptic digestion and LC–MS/MS analysis. (B) Chemoselectivity of the reported method revealed 49% lysine and 51% non-lysine modification, whereas our implementation yielded 81% lysine and 19% non-lysine modification. (C) Sequence motif analysis of both methods reveals pronounced enrichement of small residues (Ala/Val/Gly) proximal to modification sites.

Quantitative analysis of biological replicates.

Peptide level correlation

Peptide level correlation

Concentration (µM), replicates	Pearson r
100, F1 vs F2	0.957
250, F3 vs F4	0.954
500, F5 vs F6	0.973
1000, F7 vs F8	0.976
2000, F9 vs F10	0.960

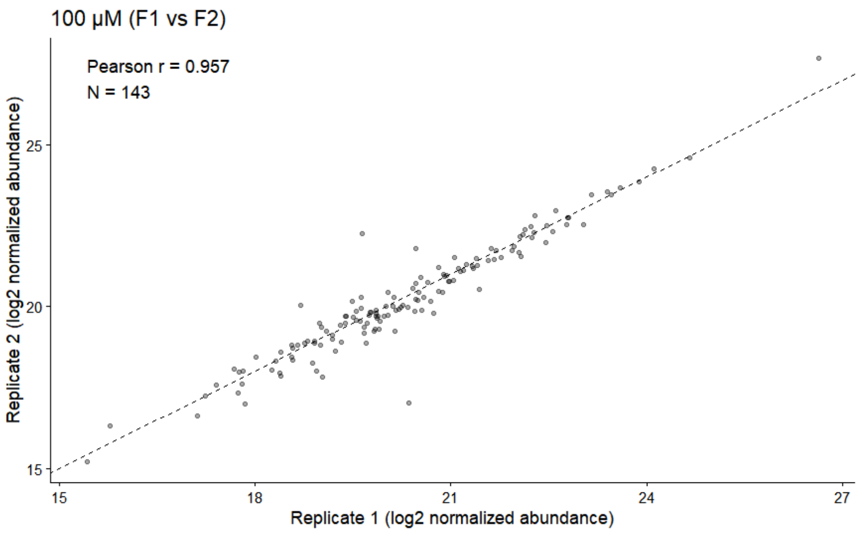
100 µM (F1 vs F2)
Pearson r = 0.957
N = 143
Replicate 2 (log2 normalized abundance)
15
20
25
15
18
21
24
27
Replicate 1 (log2 normalized abundance)

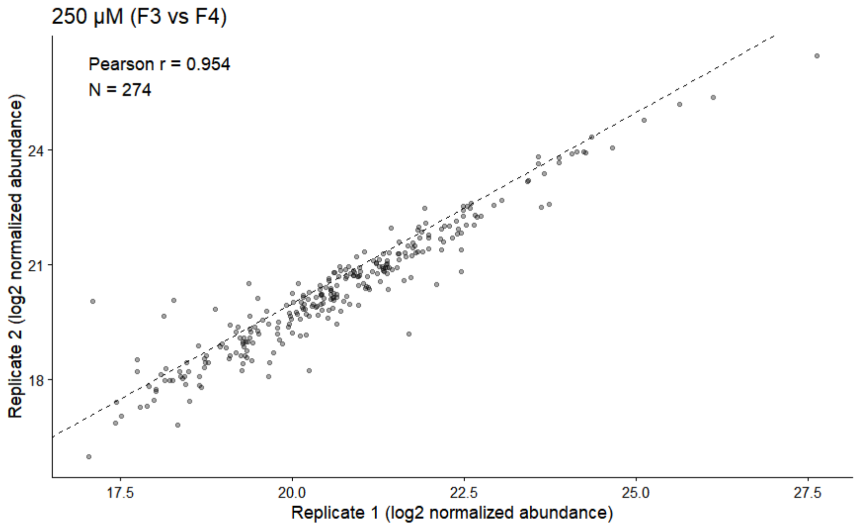
250 µM (F3 vs F4)
Pearson r = 0.954
N = 274
Replicate 2 (log2 normalized abundance)
18
21
24
17.5
20.0
22.5
25.0
27.5
Replicate 1 (log2 normalized abundance)

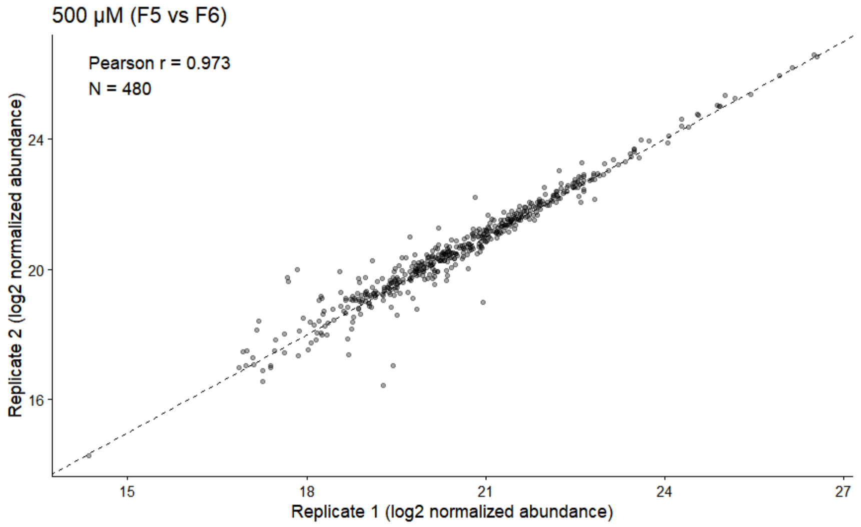
500 µM (F5 vs F6)
Pearson r = 0.973
N = 480
Replicate 2 (log2 normalized abundance)
16
20
24
15
18
21
24
27
Replicate 1 (log2 normalized abundance)

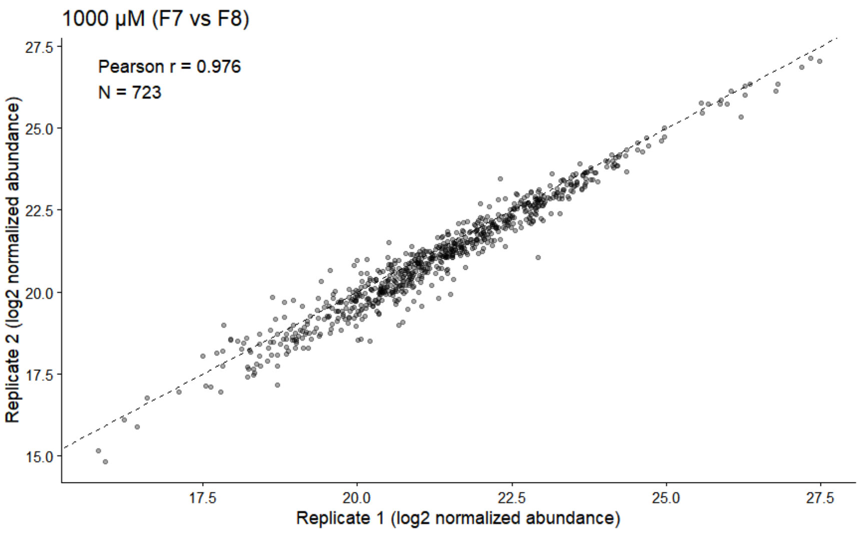

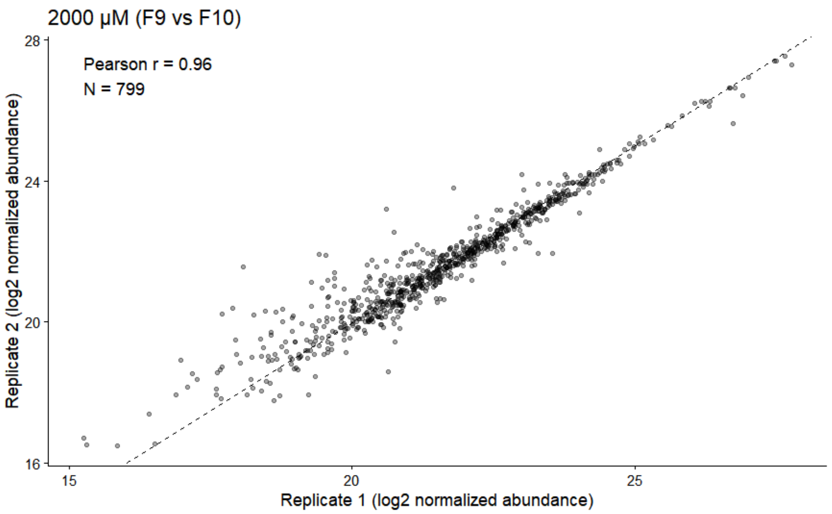

Lysine site level correlation

Site level correlation

Concentration (μM), replicates	Pearson r
100, F1 vs F2	0.969
250, F3 vs F4	0.960
500, F5 vs F6	0.969
1000, F7 vs F8	0.973
2000, F9 vs F10	0.961

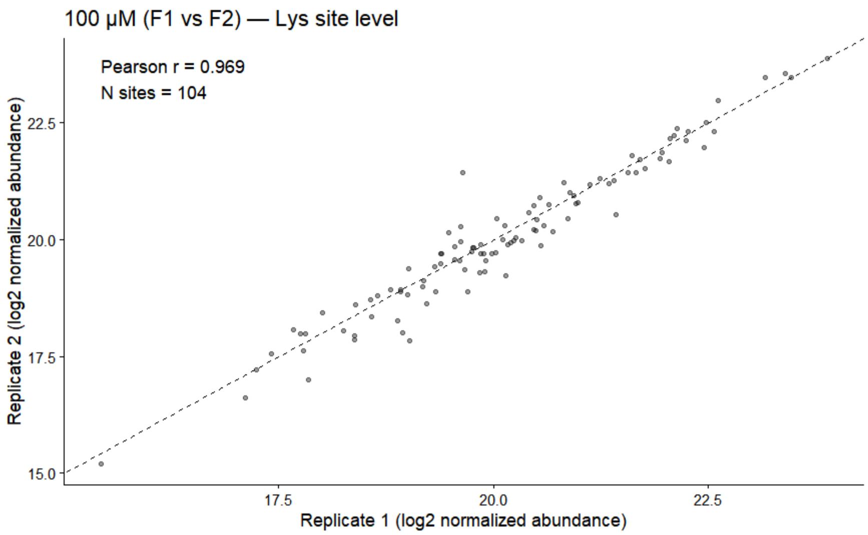
100 µM (F1 vs F2) — Lys site level
Pearson r = 0.969
N sites = 104
Replicate 2 (log2 normalized abundance)
15.0
17.5
20.0
22.5
17.5
20.0
22.5
Replicate 1 (log2 normalized abundance)

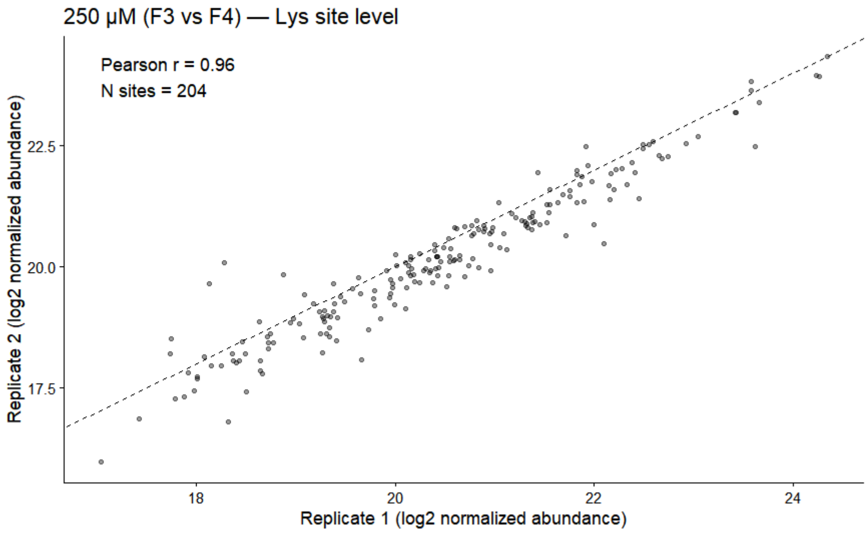
250 µM (F3 vs F4) — Lys site level
Pearson r = 0.96
N sites = 204
Replicate 2 (log2 normalized abundance)
17.5
20.0
22.5
18
20
22
24
Replicate 1 (log2 normalized abundance)

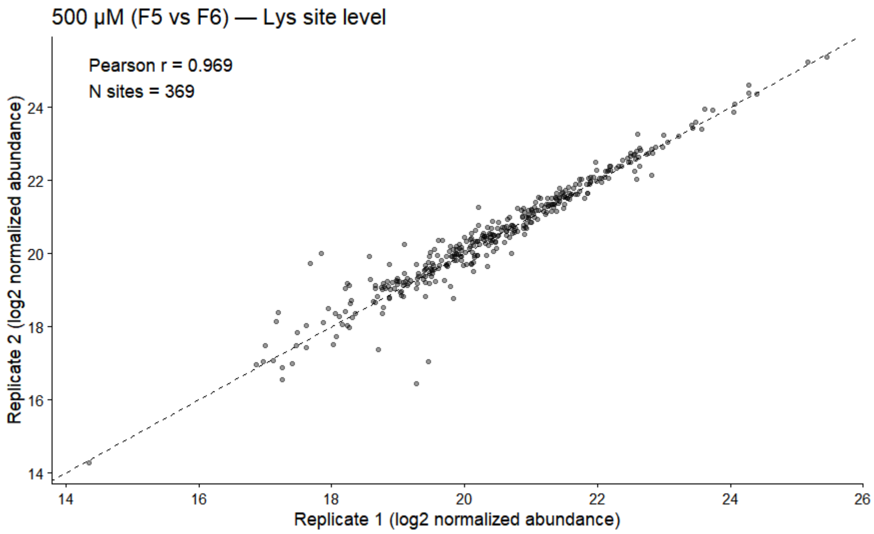
500 µM (F5 vs F6) — Lys site level
Pearson r = 0.969
N sites = 369
Replicate 2 (log2 normalized abundance)
14
16
18
20
22
24
14
16
18
20
22
24
26
Replicate 1 (log2 normalized abundance)

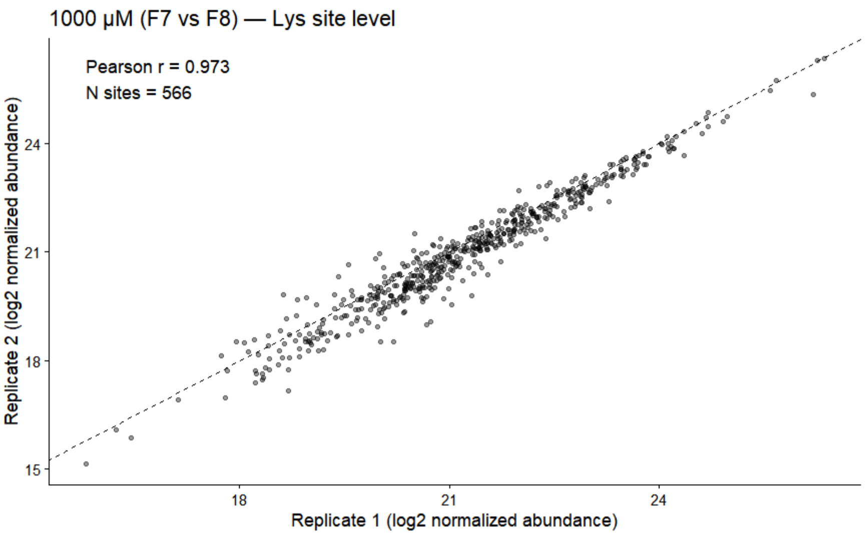

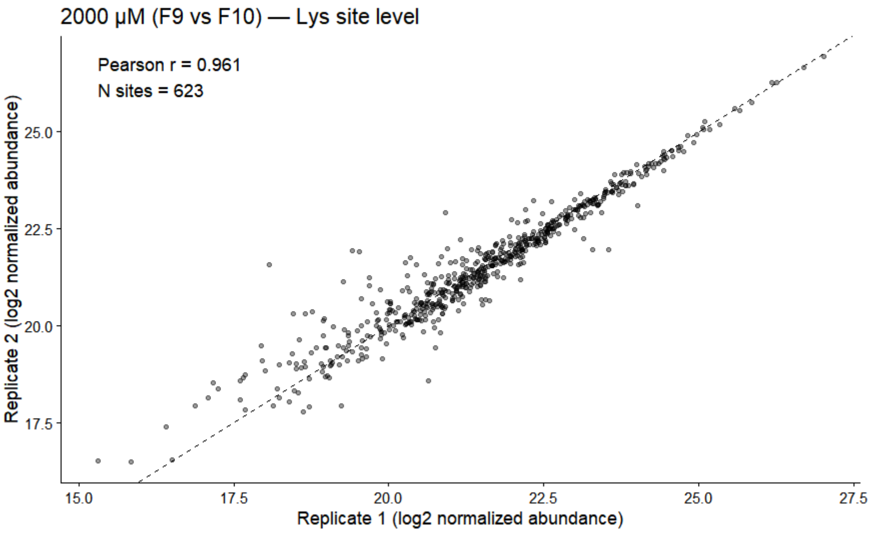

Other Key Metrics.

	condition	n_sites_rep1	n_sites_rep2	n_sites_overlap	jaccard_overlap	median_log2_abundance_rep1	median_log2_abundance_rep2	mean_median_log2_abundance	sd_median_log2_abundance
1	100	150	163	104	0.4976077	20.00148	19.90589	19.95369	0.06759136
2	250	282	258	204	0.6071429	20.42639	20.11618	20.27129	0.21935495
3	500	408	451	369	0.7530612	20.39260	20.46152	20.42706	0.04872986
4	1000	609	583	566	0.9041534	21.10703	20.96429	21.03566	0.10093100
5	2000	642	656	623	0.9229630	21.48632	21.52225	21.50429	0.02541248

Representative Annotated MS/MS Spectra.

Peptide 1 Summary.

Sequence: KDLYANTVLSGGTTMYPGIADR, K1-Ana_MDA (196.05240 Da)
Charge: +2, Monoisotopic m/z: 1270.11025 Da (-2.05 mmu/-1.62 ppm), MH+: 2539.21322 Da, RT: 68.2181 min,
Identified with: Sequest HT (v1.17); XCorr:5.36, q-Value:3.8e-6, PEP:3.7e-14, SVM Score:3.6e0,
Fragment match tolerance used for search: 0.02 Da
Fragments used for search: y-H_2O; y-NH_3; b; b-H_2O; b-NH_3; y

Peptide Summary

Sequence: KDLYANTVLSGGTTMYPGIADR, K1-Ana_MDA (196.05240 Da)
Charge: +2, Monoisotopic m/z: 1270.11025 Da (-2.05 mmu/-1.62 ppm), MH+: 2539.21322 Da, RT: 68.2181 min
Identified with: Sequest HT (v1.17); XCorr:5.36, q-Value:3.8e-6, PEP:3.7e-14, SVM Score:3.6e0,
Fragment match tolerance used for search: 0.02 Da
Fragments used for search: y-H₂O; y-NH₃; b; b-H₂O; b-NH₃; y

Fragment Matches

Value Type: Theo. Mass [Da]

Ion Series | Neutral Losses | Precursor Ions | Internal Fragments

#1	b⁺	b²⁺	Seq.	y⁺	y²⁺	#2
1	325.15464	163.08096	K-Ana_MDA			22
2	440.18158	220.59443	D	2215.06996	1108.03862	21
3	553.26565	277.13646	L	2100.04302	1050.52515	20
4	716.32897	358.66813	Y	1986.95895	993.98311	19
5	787.36609	394.18668	A	1823.89562	912.45145	18
6	901.40902	451.20815	N	1752.85851	876.93289	17
7	1002.45669	501.73199	T	1638.81558	819.91143	16
8	1101.52511	551.26619	V	1537.76790	769.38759	15
9	1214.60917	607.80822	L	1438.69949	719.85338	14
10	1301.64120	651.32424	S	1325.61543	663.31135	13
11	1358.66266	679.83497	G	1238.58340	619.79534	12
12	1415.68413	708.34570	G	1181.56193	591.28461	11
13	1516.73181	758.86954	T	1124.54047	562.77387	10
14	1617.77948	809.39338	T	1023.49279	512.25003	9
15	1748.81997	874.91362	M	922.44511	461.72620	8
16	1911.88330	956.44529	Y	791.40463	396.20595	7
17	2008.93606	1004.97167	P	628.34130	314.67429	6
18	2065.95753	1033.48240	G	531.28854	266.14791	5
19	2179.04159	1090.02443	I	474.26707	237.63717	4
20	2250.07870	1125.54299	A	361.18301	181.09514	3
21	2365.10565	1183.05646	D	290.14590	145.57659	2
22			R	175.11895	88.06311	1

Fragment Spectrum

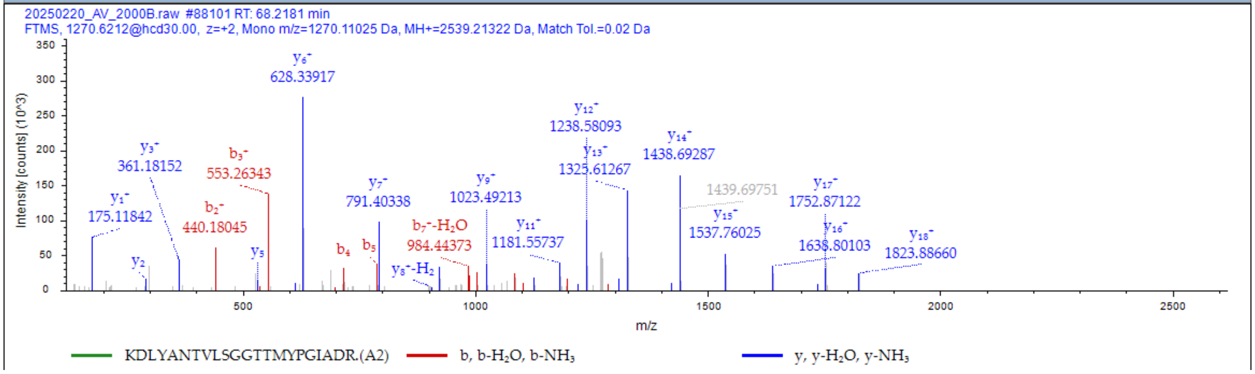

Peptide 2 Summary.
Sequence: FASFIDKVR, K7-Ana_MDA (196.05240 Da)
Charge: +2, Monoisotopic m/z: 639.82797 Da (-1.52 mmu/-2.38 ppm), MH+: 1278.64866 Da, RT: 63.0799 min,
Identified with: Sequest HT (v1.17); XCorr:2.40, q-Value:6.7e-5, PEP:4.9e-4, SVM Score:6.8e-1,
Fragment match tolerance used for search: 0.02 Da
Fragments used for search: y-H_2O; y-NH_3; b; b-H_2O; y

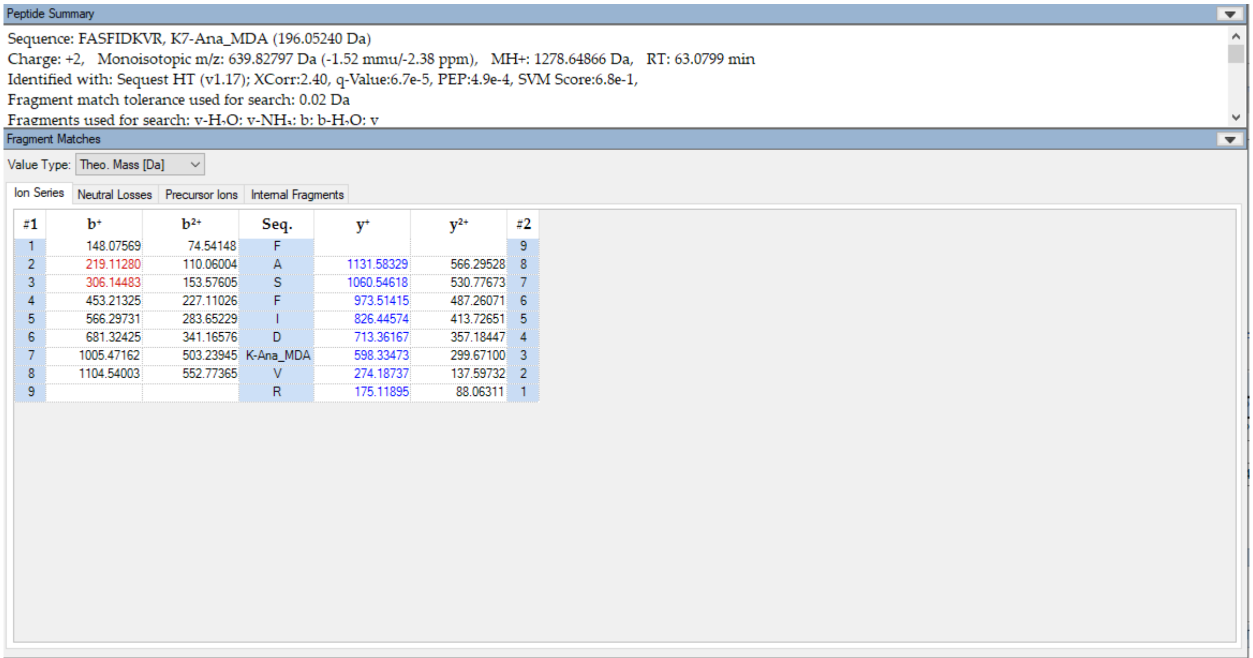

Peptide Summary

Sequence: FASFIDKVR, K7-Ana_MDA (196.05240 Da)
Charge: +2, Monoisotopic m/z: 639.82797 Da (-1.52 mmu/-2.38 ppm), MH+: 1278.64866 Da, RT: 63.0799 min
Identified with: Sequest HT (v1.17); XCorr:2.40, q-Value:6.7e-5, PEP:4.9e-4, SVM Score:6.8e-1,
Fragment match tolerance used for search: 0.02 Da
Fragments used for search: y-H₂O; y-NH₃; b; b-H₂O; y

Fragment Matches

Value Type: Theo. Mass [Da]

Ion Series | Neutral Losses | Precursor Ions | Internal Fragments

#1	b⁺	b²⁺	Seq.	y⁺	y²⁺	#2
1	148.07569	74.54148	F			9
2	219.11280	110.06004	A	1131.58329	566.29528	8
3	306.14483	153.57605	S	1060.54618	530.77673	7
4	453.21325	227.11026	F	973.51415	487.26071	6
5	566.29731	283.65229	I	826.44574	413.72651	5
6	681.32425	341.16576	D	713.36167	357.18447	4
7	1005.47162	503.23945	K-Ana_MDA	598.33473	299.67100	3
8	1104.54003	552.77365	V	274.18737	137.59732	2
9			R	175.11895	88.06311	1

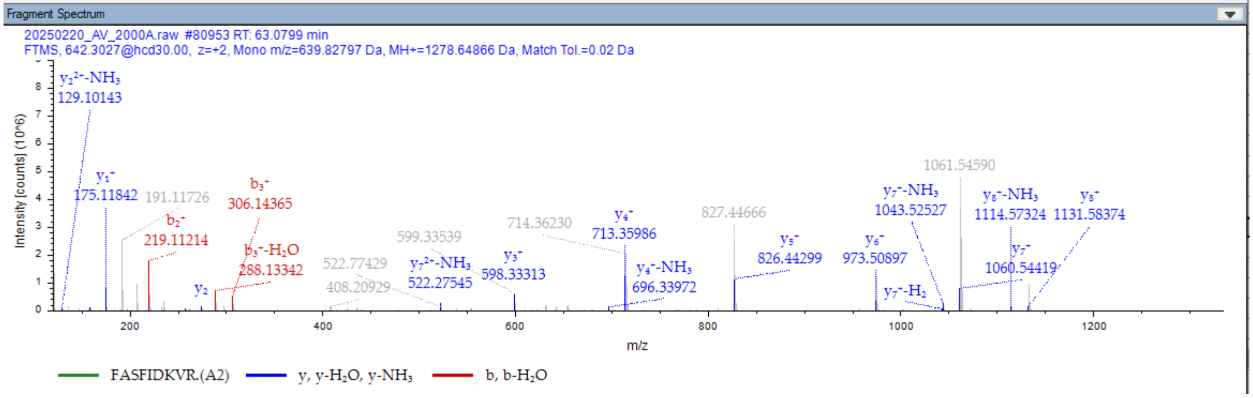

Peptide 3 Summary.

Sequence: DVDEAYMNKVELESR, K9-Ana_MDA (196.05240 Da)

Charge: +2, Monoisotopic m/z: 997.43770 Da (-8.32 mmu/-8.34 ppm), MH+: 1993.86813 Da, RT: 63.7622 min,

Identified with: Sequest HT (v1.17); XCorr:3.49, q-Value:3.8e-6, PEP:2.1e-6, SVM Score:1.4e0,

Fragment match tolerance used for search: 0.02 Da

Fragments used for search: $y-H_2O$; $y-NH_3$; b; $b-H_2O$; $b-NH_3$; y

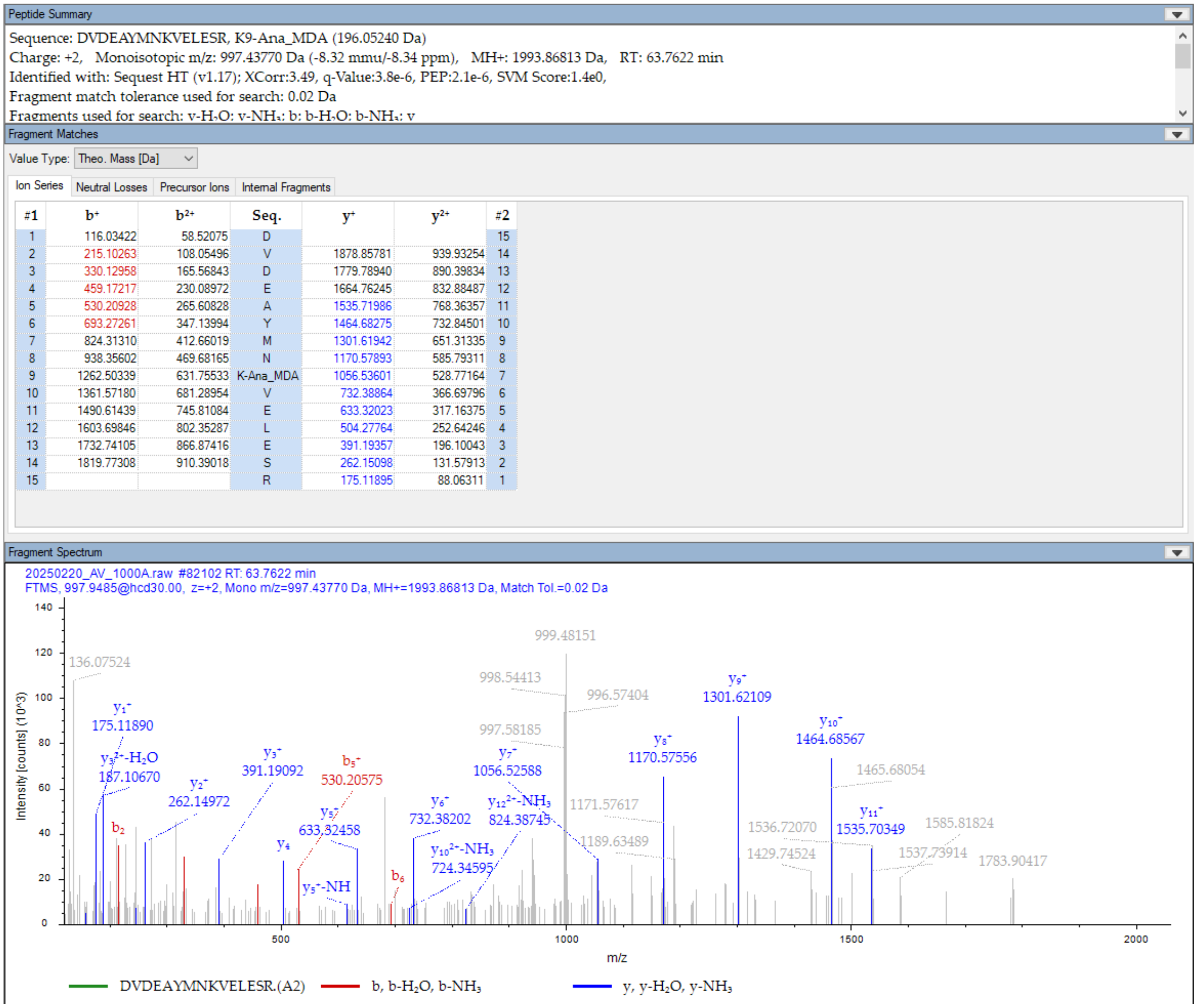

#1	b+	b2+	Seq.	y+	y2+	#2
1	116.03422	58.52075	D			15
2	215.10263	108.05496	V	1878.85781	939.93254	14
3	330.12958	165.56843	D	1779.78940	890.39834	13
4	459.17217	230.08972	E	1664.76245	832.88487	12
5	530.20928	265.60828	A	1535.71986	768.36357	11
6	693.27261	347.13994	Y	1464.68275	732.84501	10
7	824.31310	412.66019	M	1301.61942	651.31335	9
8	938.35602	469.68165	N	1170.57893	585.79311	8
9	1262.50339	631.75533	K-Ana_MDA	1056.53601	528.77164	7
10	1361.57180	681.28954	V	732.38864	366.69796	6
11	1490.61439	745.81084	E	633.32023	317.16375	5
12	1603.69846	802.35287	L	504.27764	252.64246	4
13	1732.74105	866.87416	E	391.19357	196.10043	3
14	1819.77308	910.39018	S	262.15098	131.57913	2
15			R	175.11895	88.06311	1

Supplementary Figure 20. Ionic and Non-ionic Strength Studies on Lysozyme Chicken.

MDA-benzaldehyde.

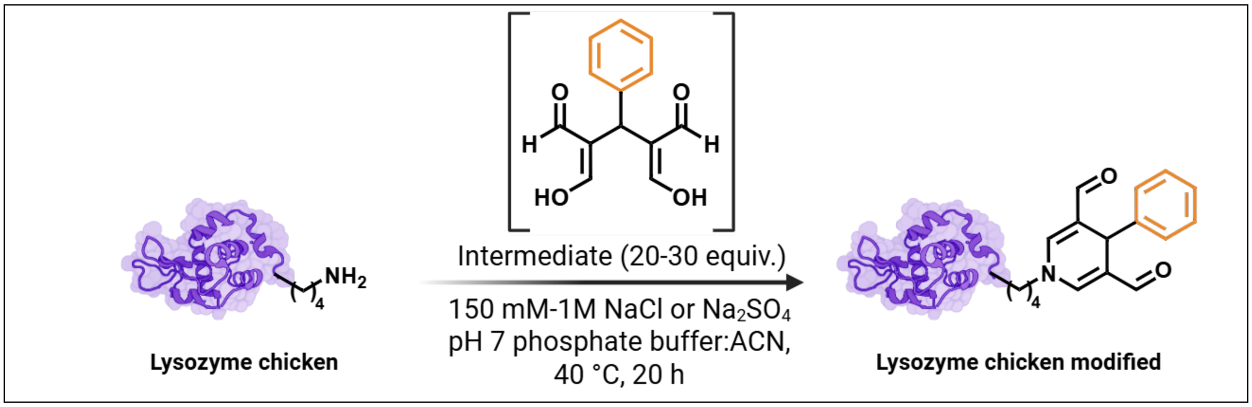

In a one-dram vial, lysozyme chicken (1 mg, 69.9 nmol) was dissolved in 100 µL of ACN and 300 µL of an NaCl or Na_2SO_4 solution made with sodium phosphate buffer (100 mM, pH 7) and varying

concentrations of NaCl or Na_2SO_4 (150 mM - 1 M). The pH of the solution was monitored after addition of the NaCl or Na_2SO_4 solution. The protein was allowed to stir in the NaCl or Na_2SO_4 solution for 30 min after which the intermediate was added (20 or 30 equiv.) and left to stir at 37 °C for 20 hours. Subsequently, the reaction mixture was passed through Amicon Ultra 3 kDa spin-concentrator and washed with H_2O (5 x 500 µL) to remove the small molecule impurities. The labeled protein was redissolved in 0.1% formic acid in H_2O and analyzed using LC-MS. Modification of MDA-benzaldehyde is confirmed by a mass shift of +196 *m/z*. Using 0 mM NaCl resulted in 67% conversion, 150 mM NaCl resulted in 33% conversion, 500 mM NaCl resulted in 31% conversion, and 1 M NaCl resulted in 17% conversion. Using 0 mM Na_2SO_4 resulted in 73% conversion (40.5% with 1 modification, 27.0% with 2 modifications, and 5.4% with 3 modifications), 150 mM Na_2SO_4 resulted in 50% conversion (37.5% with 1 modification, and 12.5% with 2 modifications), 500 mM Na_2SO_4 resulted in 50% conversion (38.2% with 1 modification, and 11.8% with 2 modifications), and 1 M Na_2SO_4 resulted in 51.2% conversion (36.6% with 1 modification, and 14.6% with 2 modifications).

Intact MS Spectrum of Starting Lysozyme Chicken

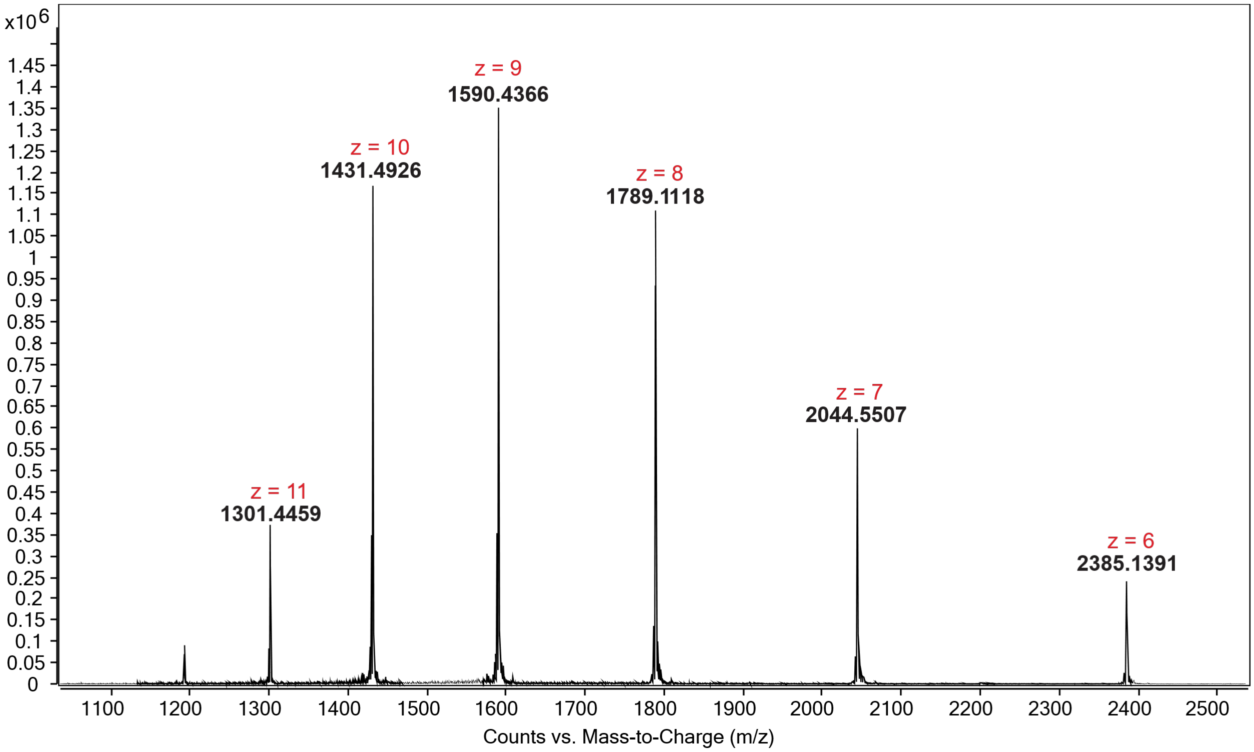

Deconvoluted MS Spectrum of Starting Lysozyme Chicken

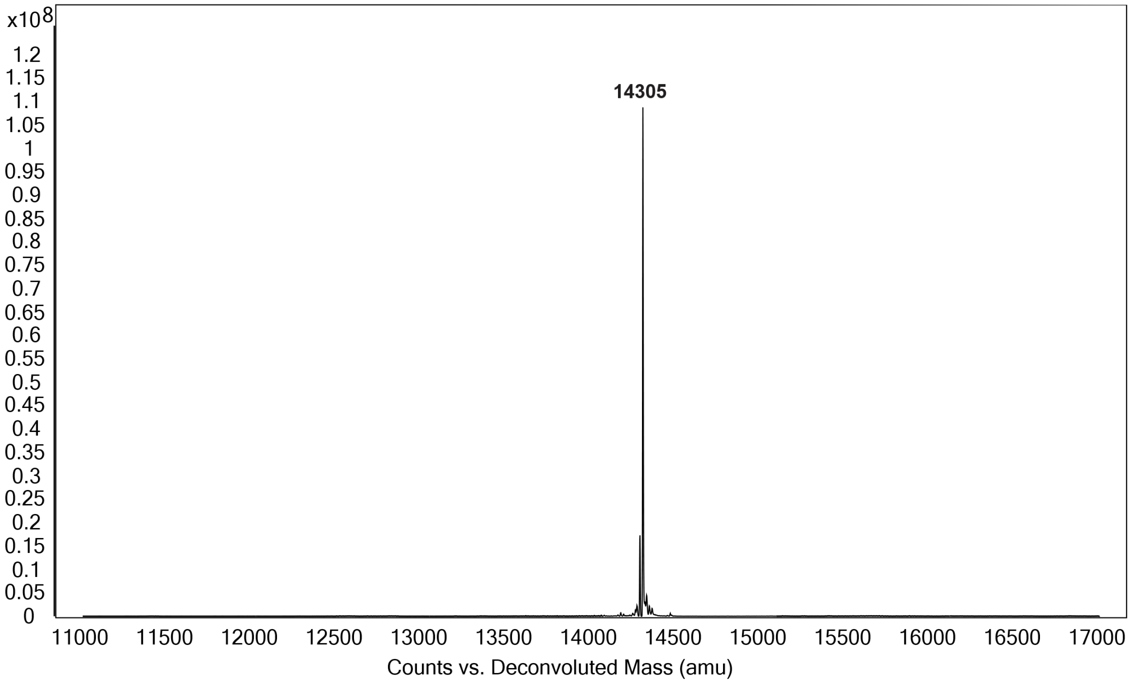

Intact MS Spectrum of Modified Lysozyme Chicken (0 mM NaCl)

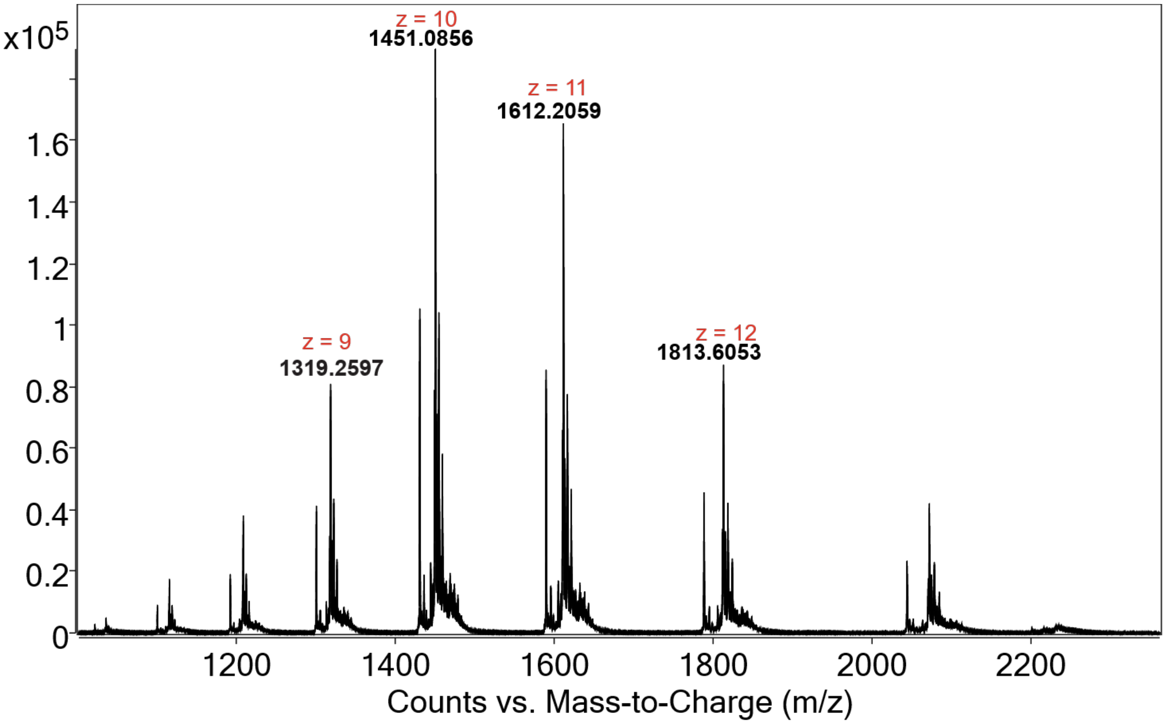

Deconvoluted MS Spectrum of Modified Lysozyme Chicken (0 mM NaCl)

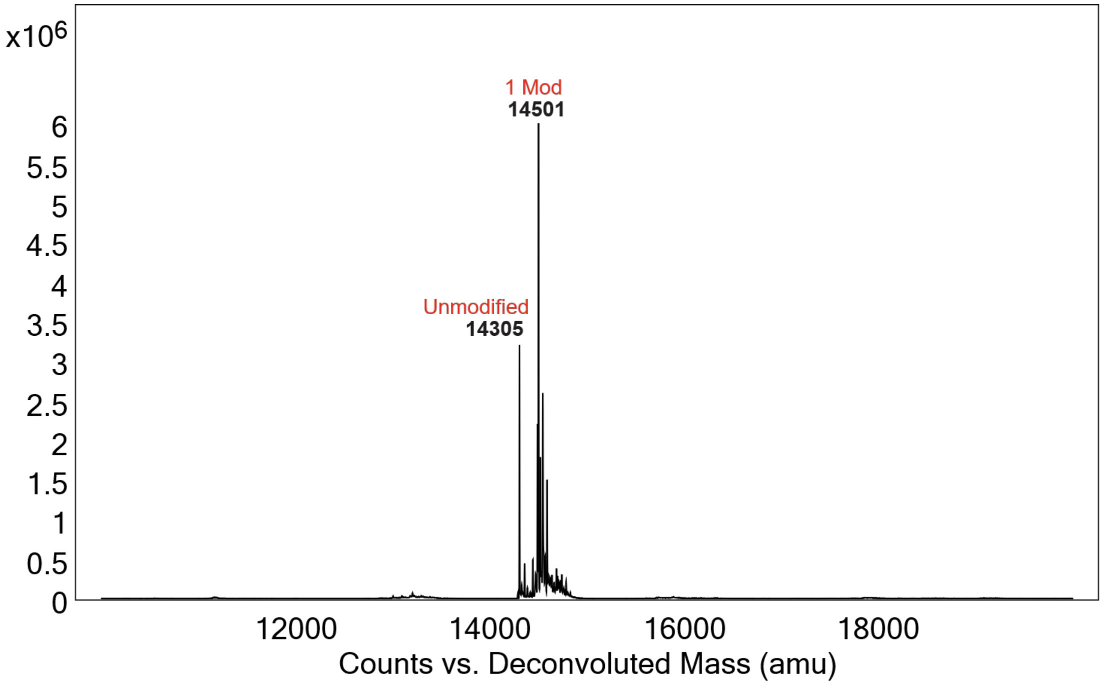

Intact MS Spectrum of Modified Lysozyme Chicken (150 mM NaCl)

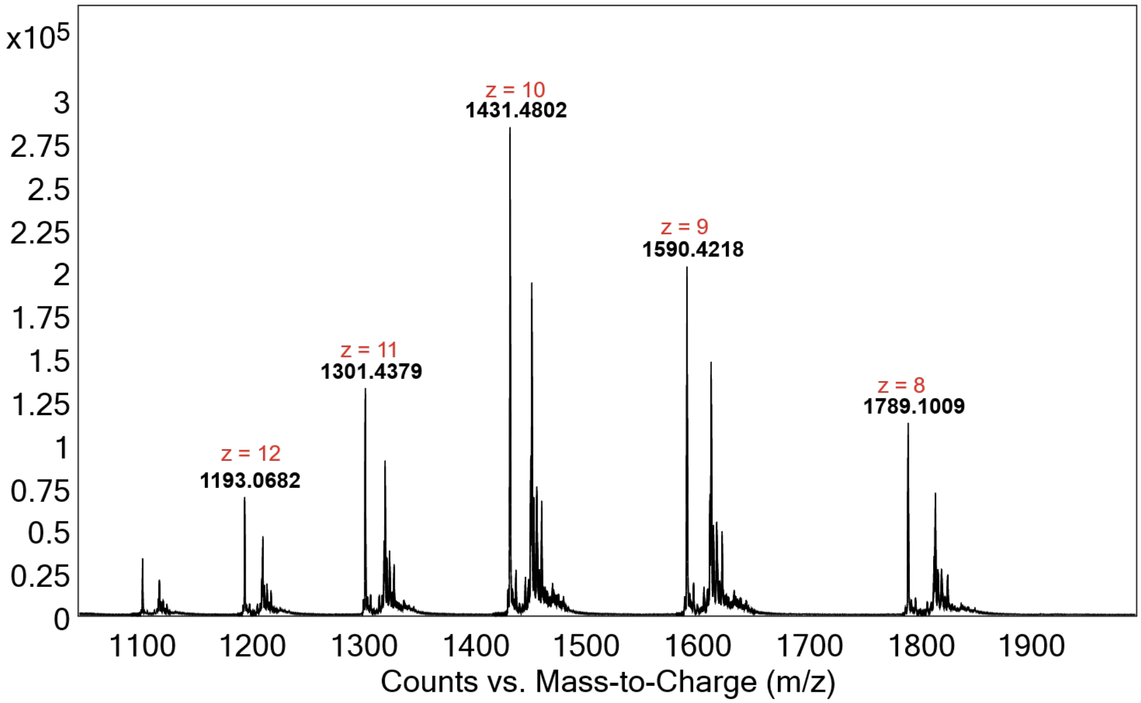

Deconvoluted MS Spectrum of Modified Lysozyme Chicken (150 mM NaCl)

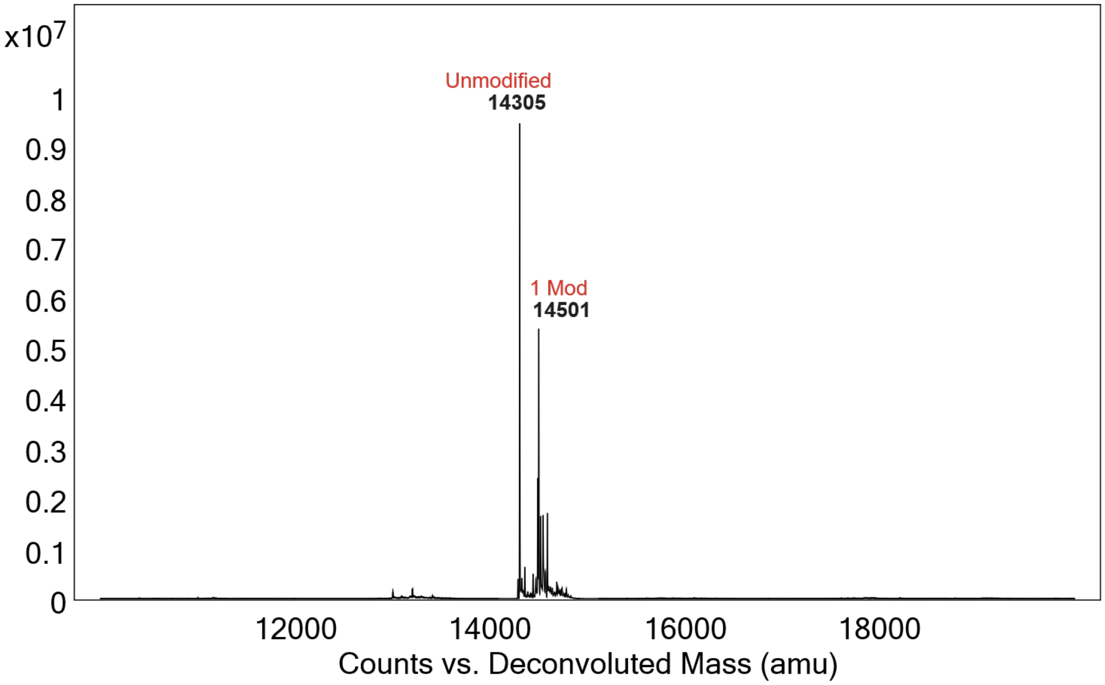

Intact MS Spectrum of Modified Lysozyme Chicken (500 mM NaCl)

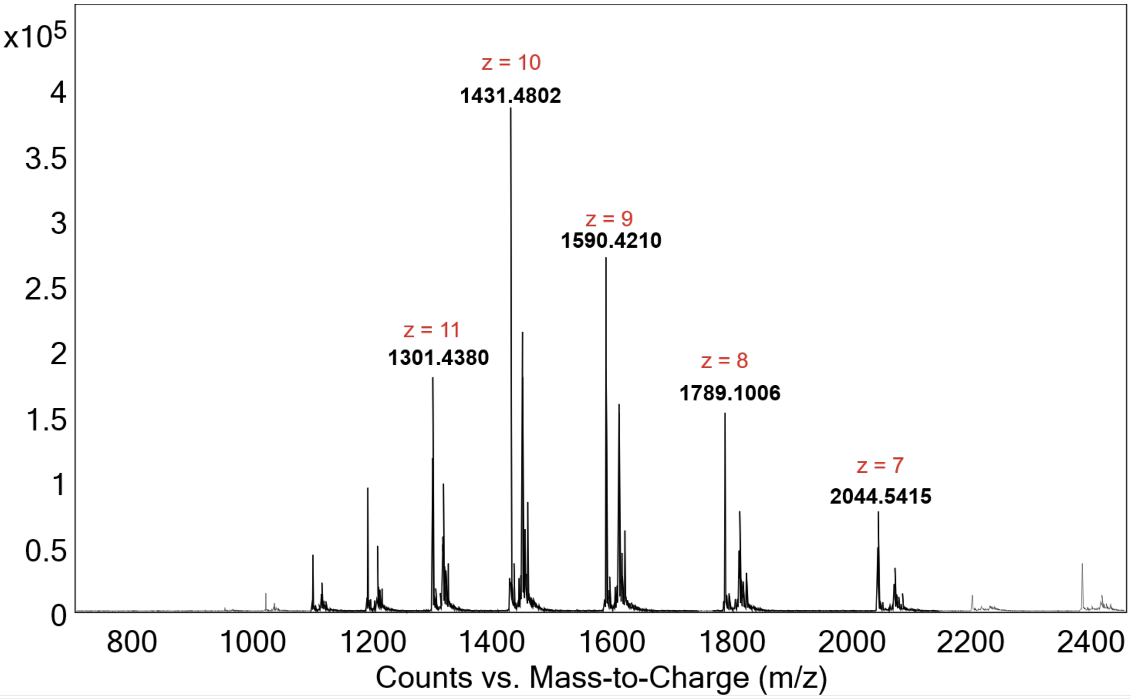

Deconvoluted MS Spectrum of Modified Lysozyme Chicken (500 mM NaCl)

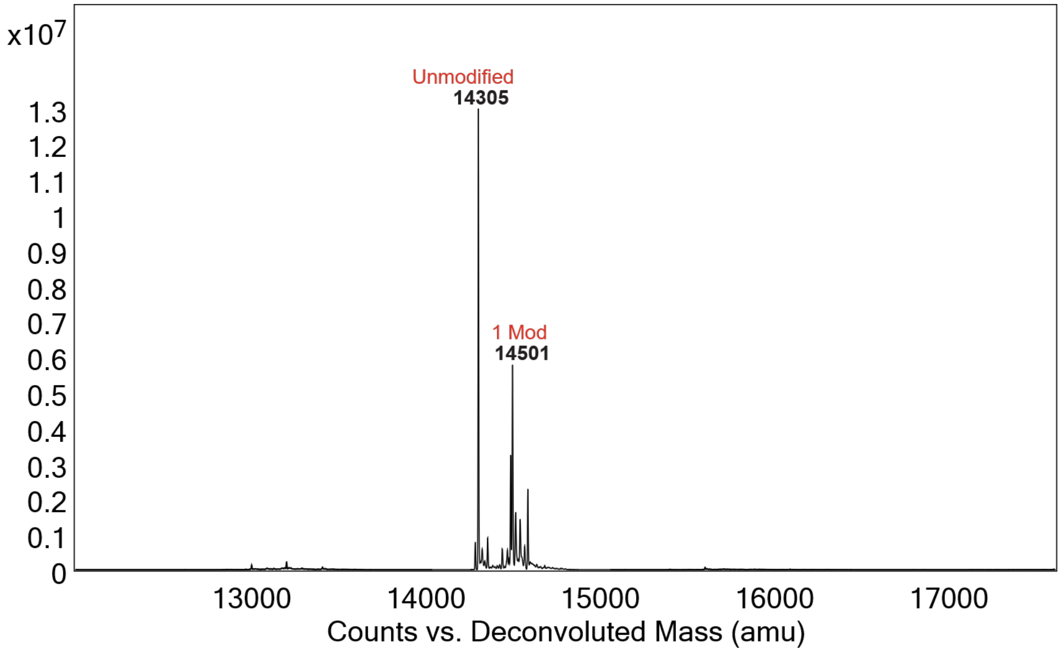

Intact MS Spectrum of Modified Lysozyme Chicken (1 M NaCl)

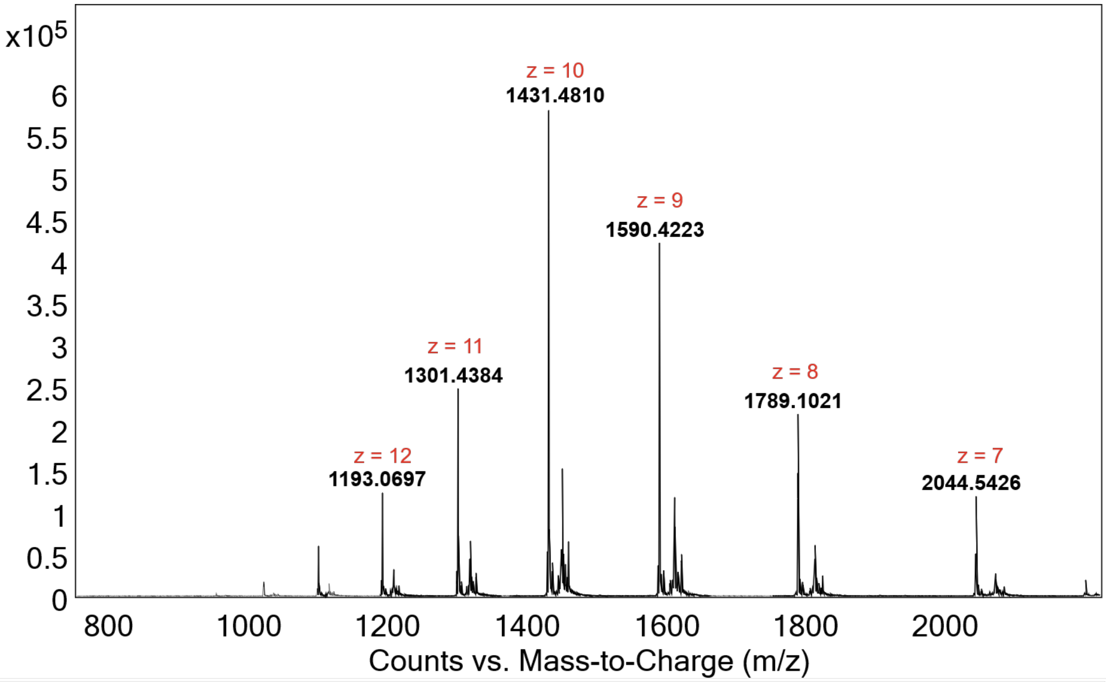

Deconvoluted MS Spectrum of Modified Lysozyme Chicken (1 M NaCl)

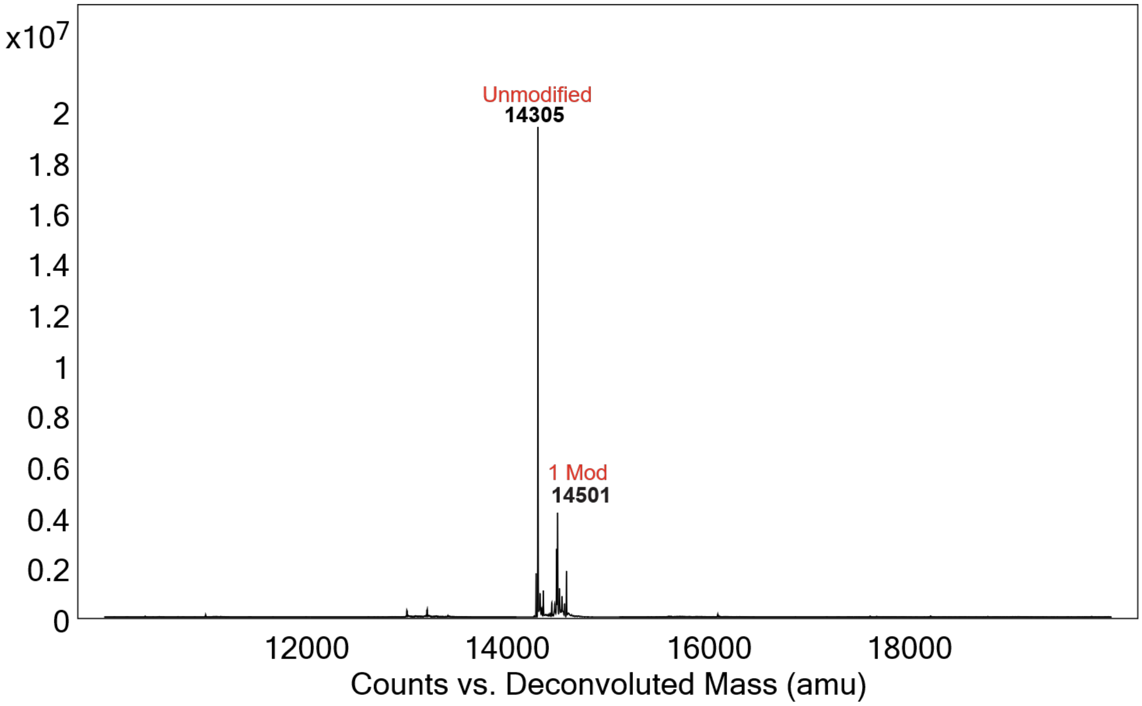

Intact MS Spectrum of Modified Lysozyme Chicken (0 mM Na_2SO_4)

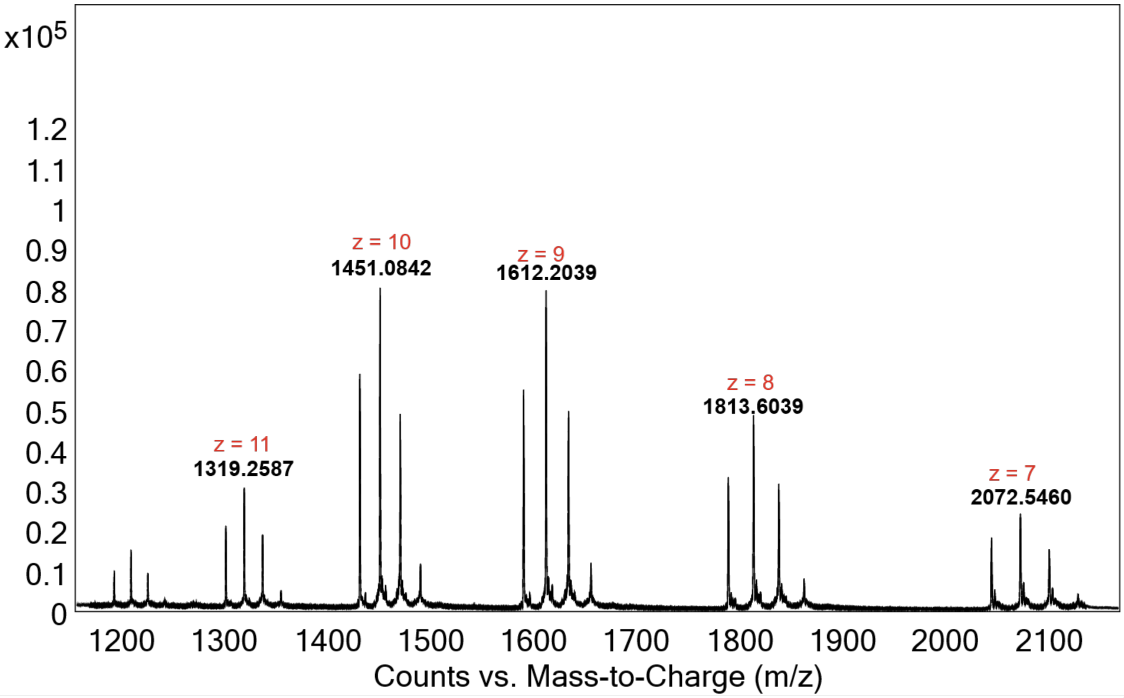

Deconvoluted MS Spectrum of Modified Lysozyme Chicken (0 mM Na_2SO_4)

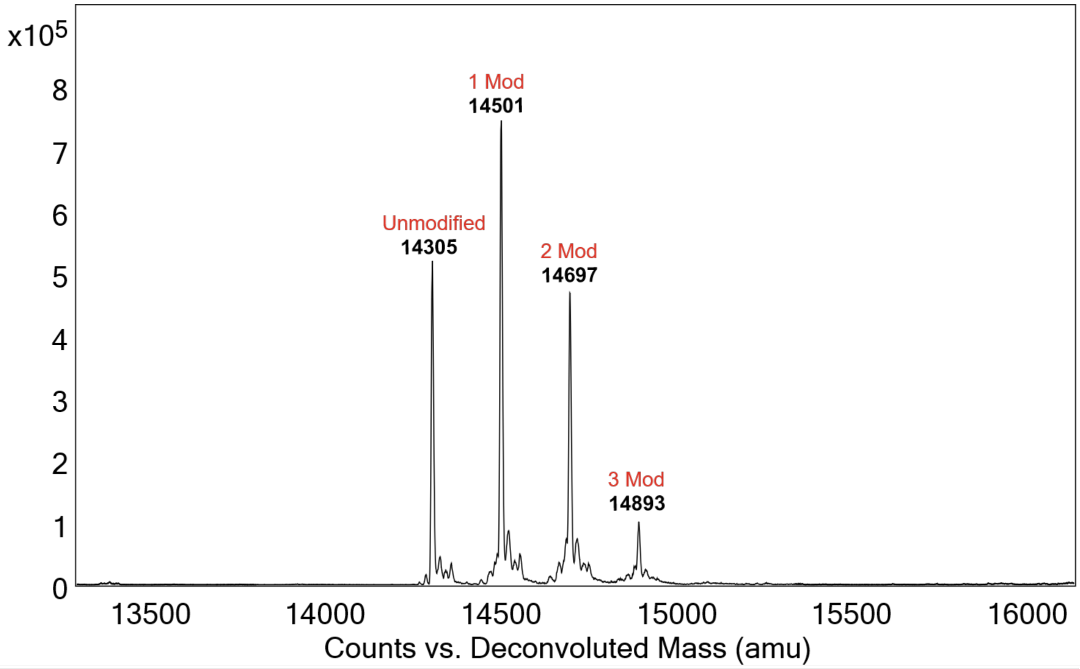

Intact MS Spectrum of Modified Lysozyme Chicken (150 mM Na_2SO_4)

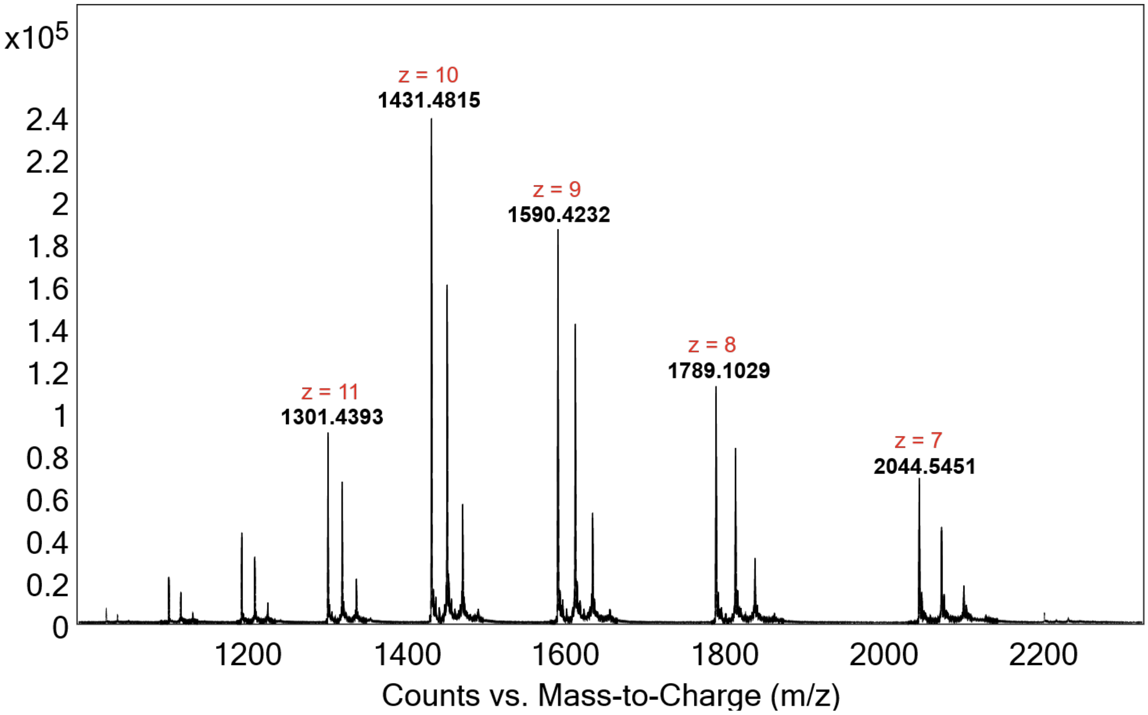

Deconvoluted MS Spectrum of Modified Lysozyme Chicken (150 mM Na_2SO_4)

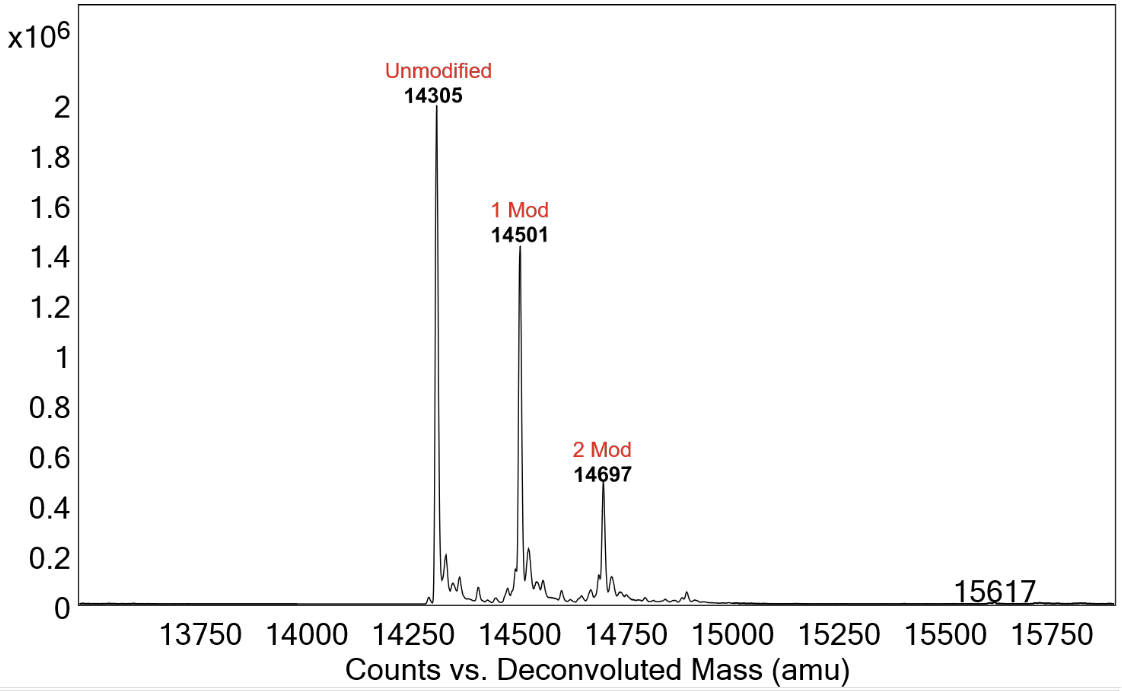

Intact MS Spectrum of Modified Lysozyme Chicken (500 mM Na_2SO_4)

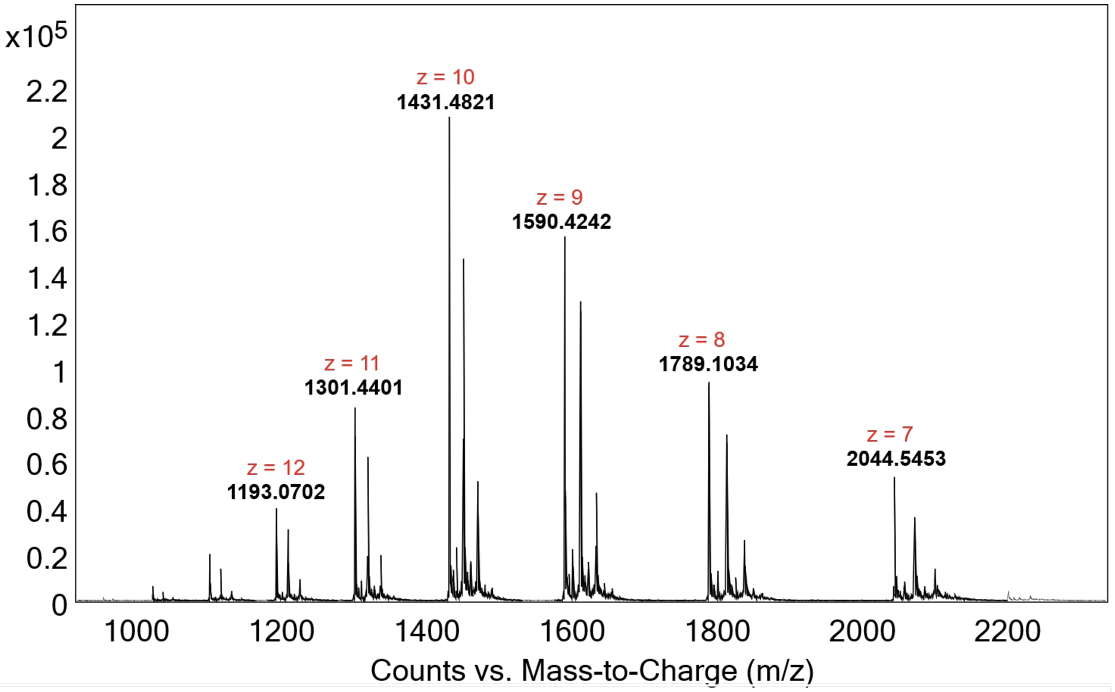

Deconvoluted MS Spectrum of Modified Lysozyme Chicken (500 mM Na_2SO_4)

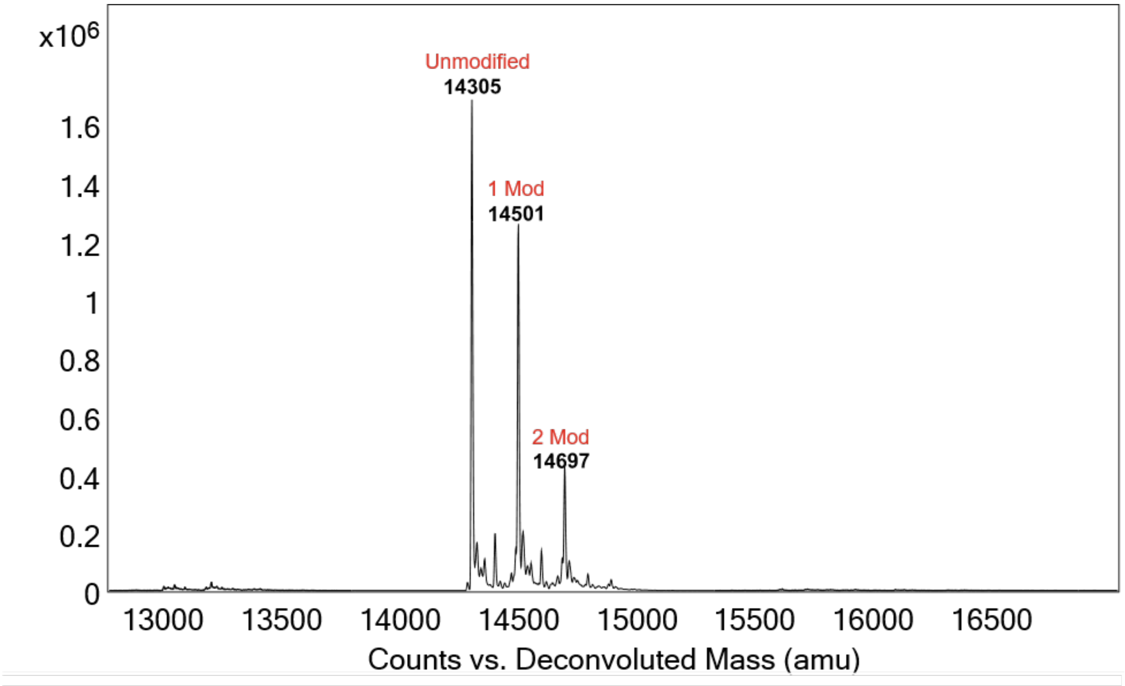

Intact MS Spectrum of Modified Lysozyme Chicken (1 M Na_2SO_4)

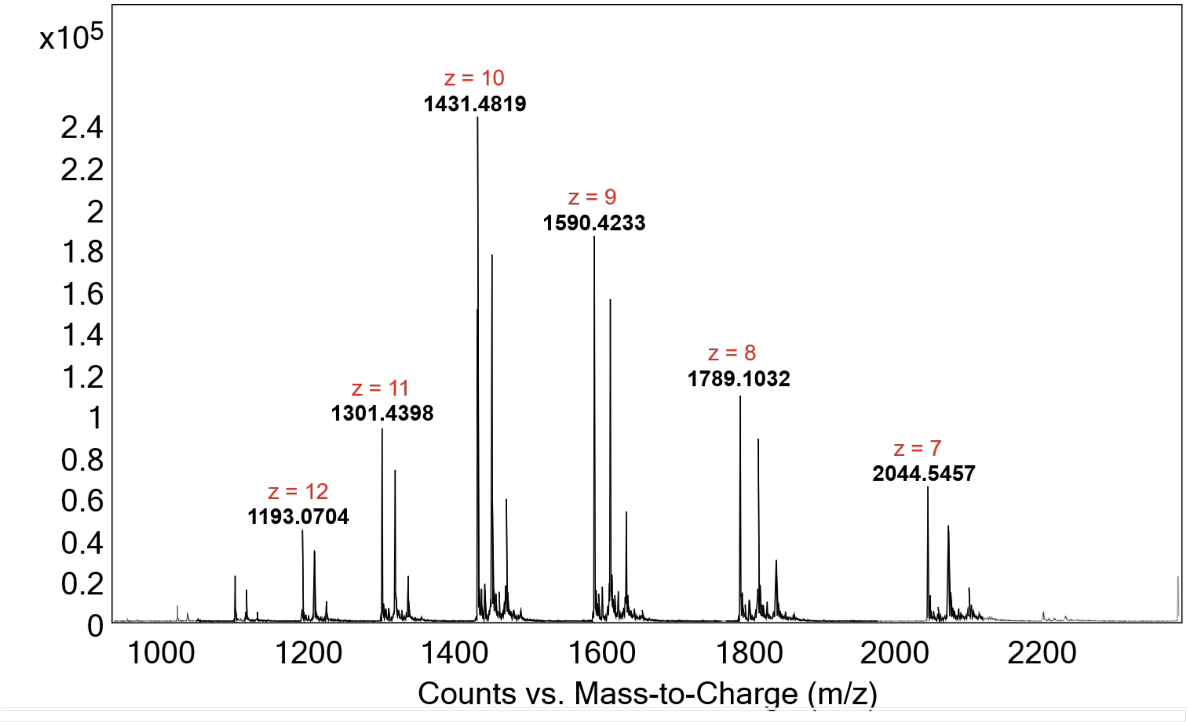

Deconvoluted MS Spectrum of Modified Lysozyme Chicken (1 M Na_2SO_4)

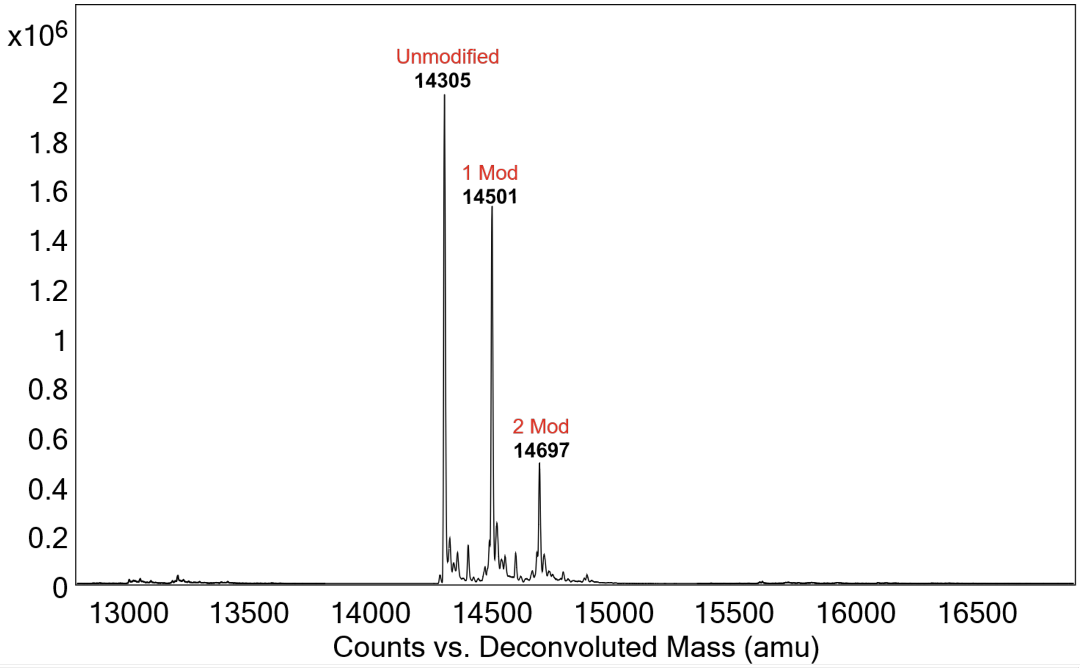

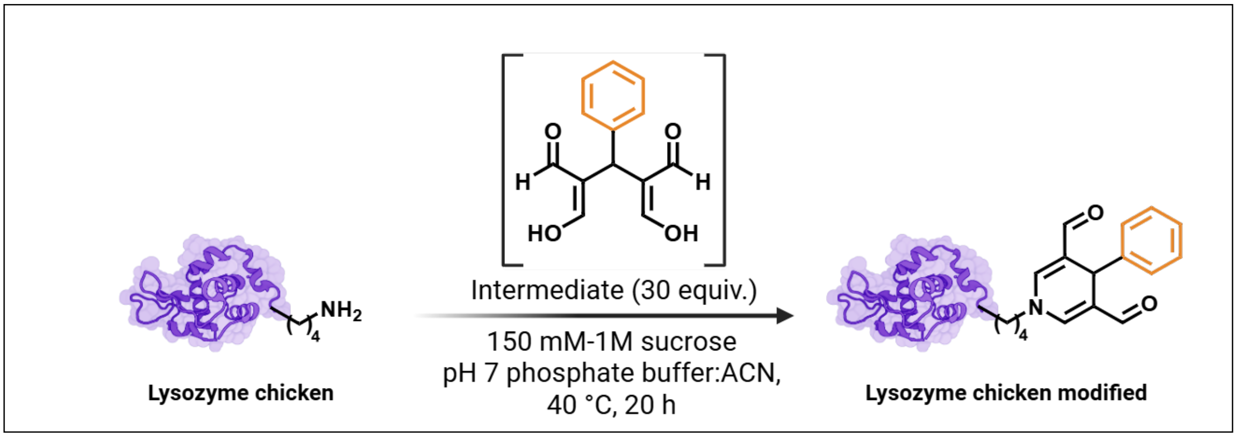

In a one-dram vial, lysozyme chicken (1 mg, 69.9 nmol) was dissolved in 100 µL of ACN and 300 µL of a sucrose solution made with sodium phosphate buffer (100 mM, pH 7) and varying concentrations of sucrose (150 mM - 1 M). The pH of the solution was monitored after addition of the sucrose solution. The protein was allowed to stir in the sucrose solution for 30 min after which the intermediate was added (30 equiv.) and left to stir at 37 °C for 20 hours. Subsequently, the reaction mixture was passed through Amicon Ultra 3 kDa spin-concentrator and washed with H_2O (5 x 500 µL) to remove the small molecule impurities. The labeled protein was redissolved in 0.1% formic acid in H_2O and analyzed using LC-MS. Modification of MDA-benzaldehyde is confirmed by a mass shift of +196 *m/z*. Using 0 mM sucrose resulted in 73% conversion (36.4% with 1 modification, 27.5% with 2 modifications, and 9.1% with 3 modifications), 150 mM sucrose resulted in 76% conversion (37.5% with 1 modification, and 29.2% with 2 modifications, and 12.5% with 3 modifications), 500 mM Na_2SO_4 resulted in 84% conversion (36.4% with 1 modification, 32.7% with 2 modifications, and 14.5% with 3 modifications), and 1 M sucrose

resulted in 81% conversion (37.5% with 1 modification, 31.3% with 2 modifications, and 12.5% with 3 modifications).

Intact MS Spectrum of Starting Lysozyme Chicken

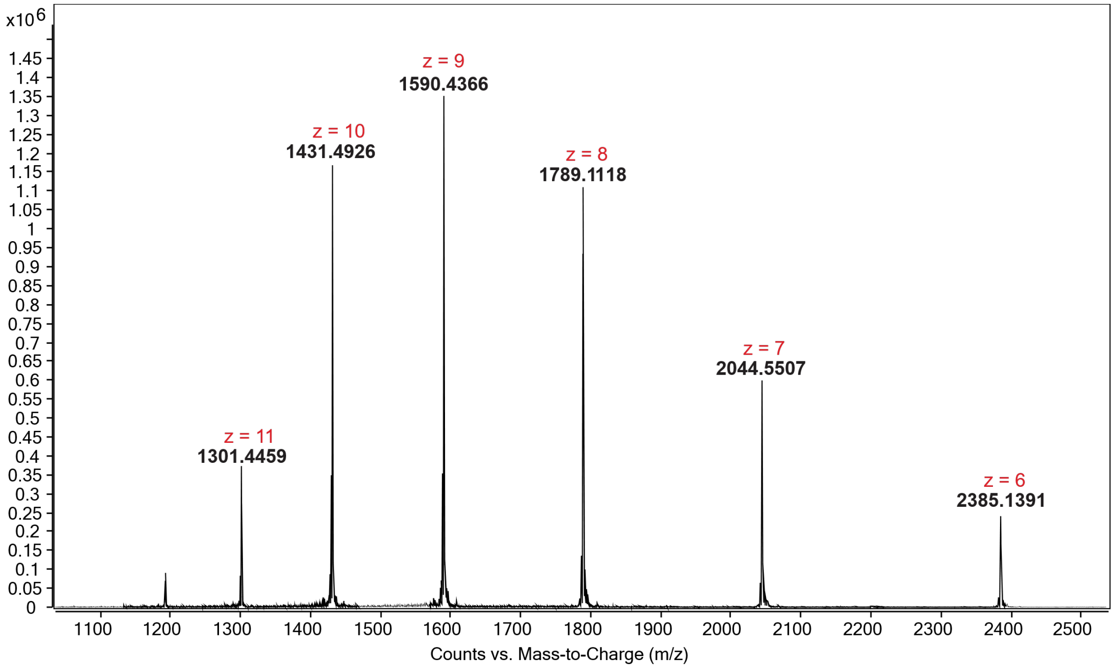

Deconvoluted MS Spectrum of Starting Lysozyme Chicken

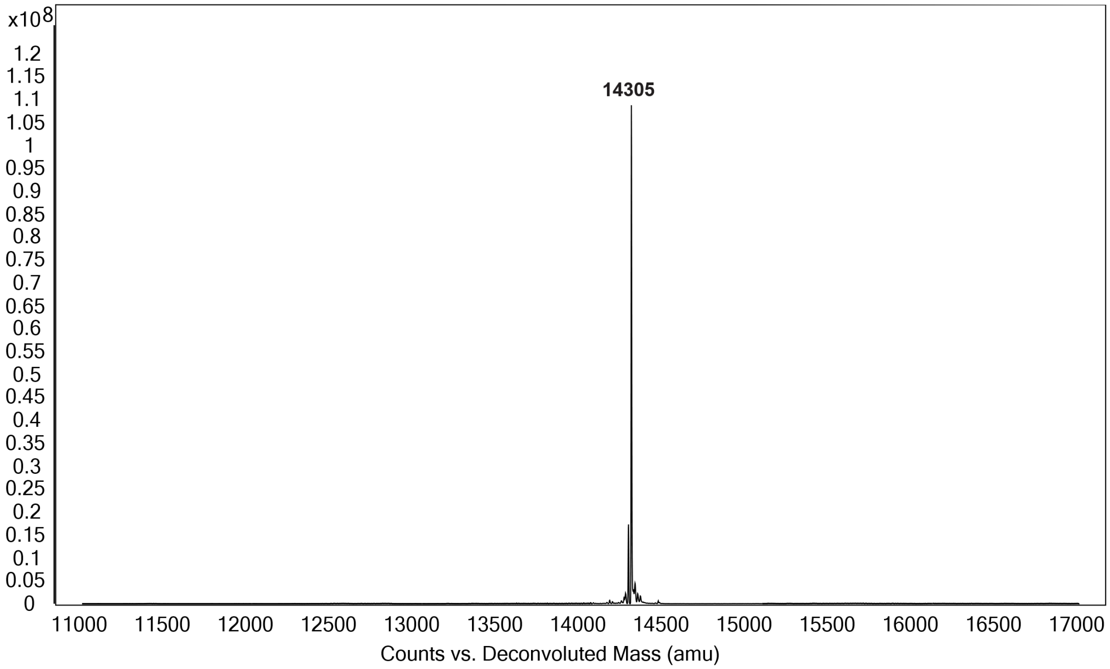

Intact MS Spectrum of Modified Lysozyme Chicken (0 mM sucrose)

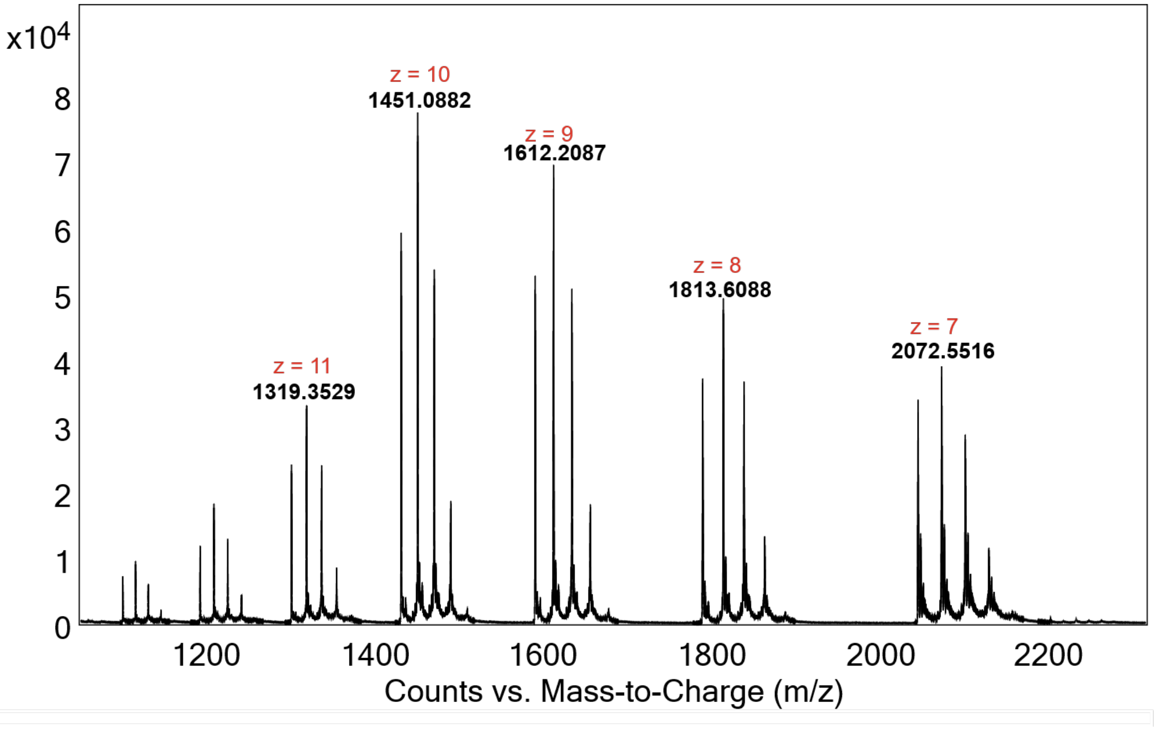

Deconvoluted MS Spectrum of Modified Lysozyme Chicken (0 mM sucrose)

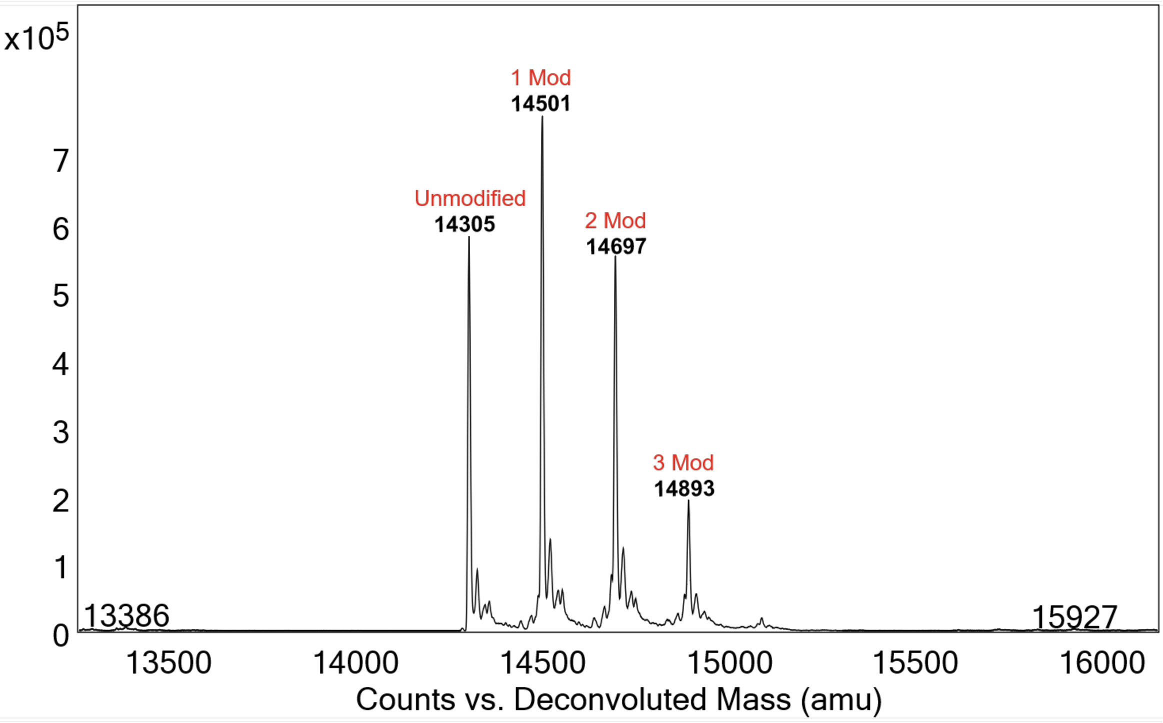

Intact MS Spectrum of Modified Lysozyme Chicken (150 mM sucrose)

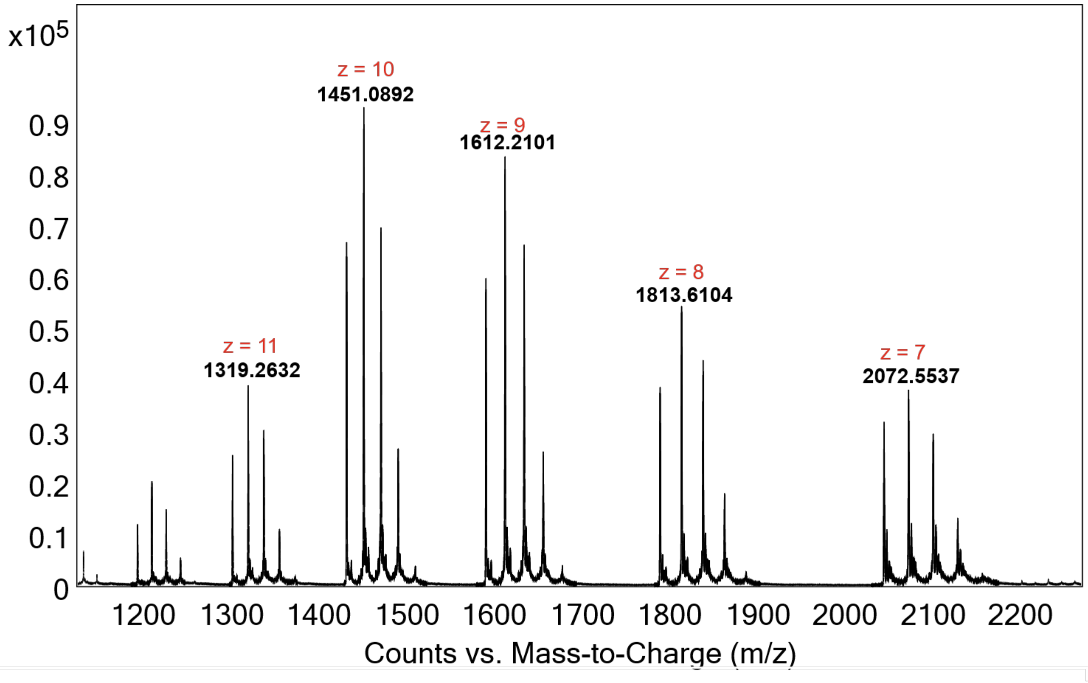

Deconvoluted MS Spectrum of Modified Lysozyme Chicken (150 mM sucrose)

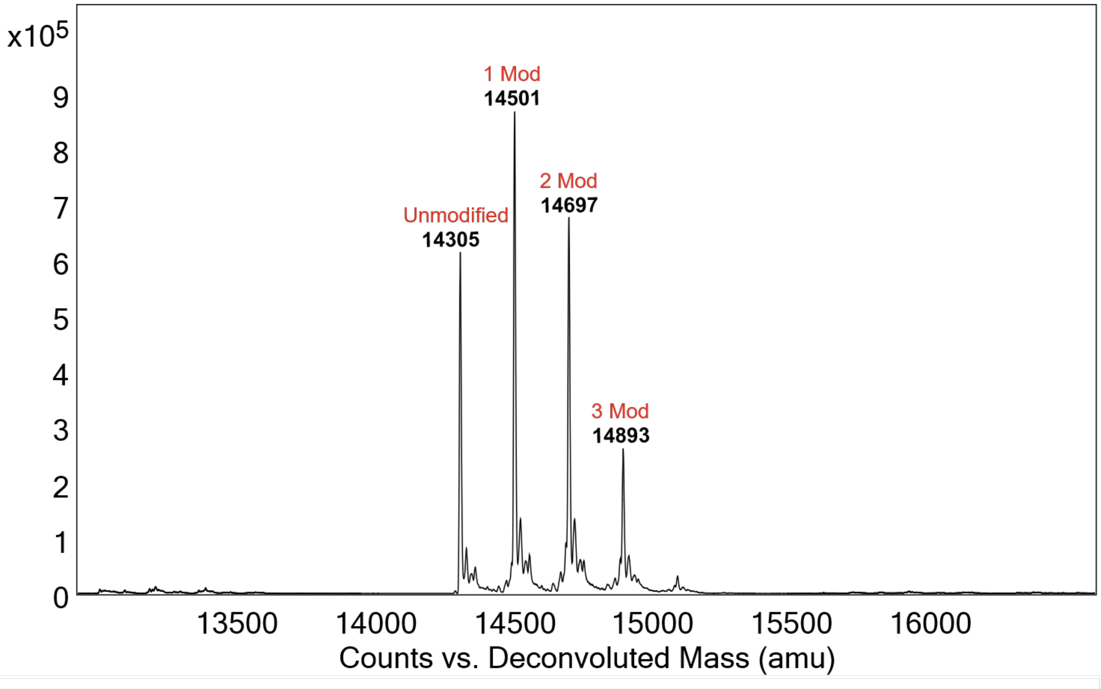

Intact MS Spectrum of Modified Lysozyme Chicken (500 mM sucrose)

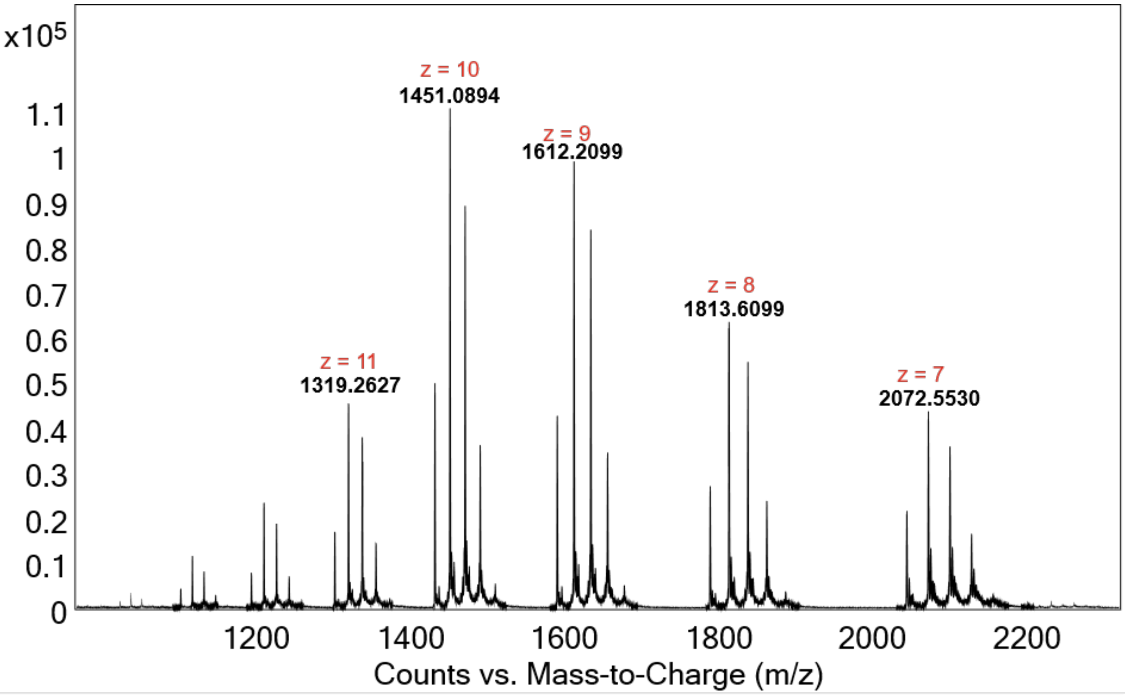

Deconvoluted MS Spectrum of Modified Lysozyme Chicken (500 mM sucrose)

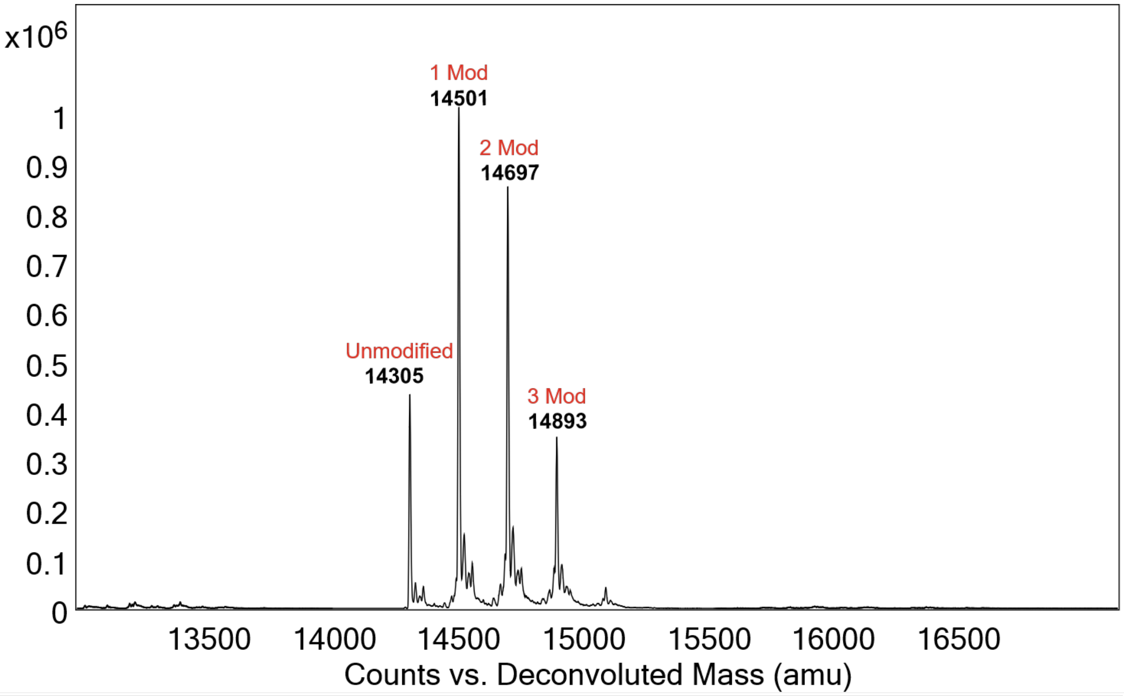

Intact MS Spectrum of Modified Lysozyme Chicken (1 M sucrose)

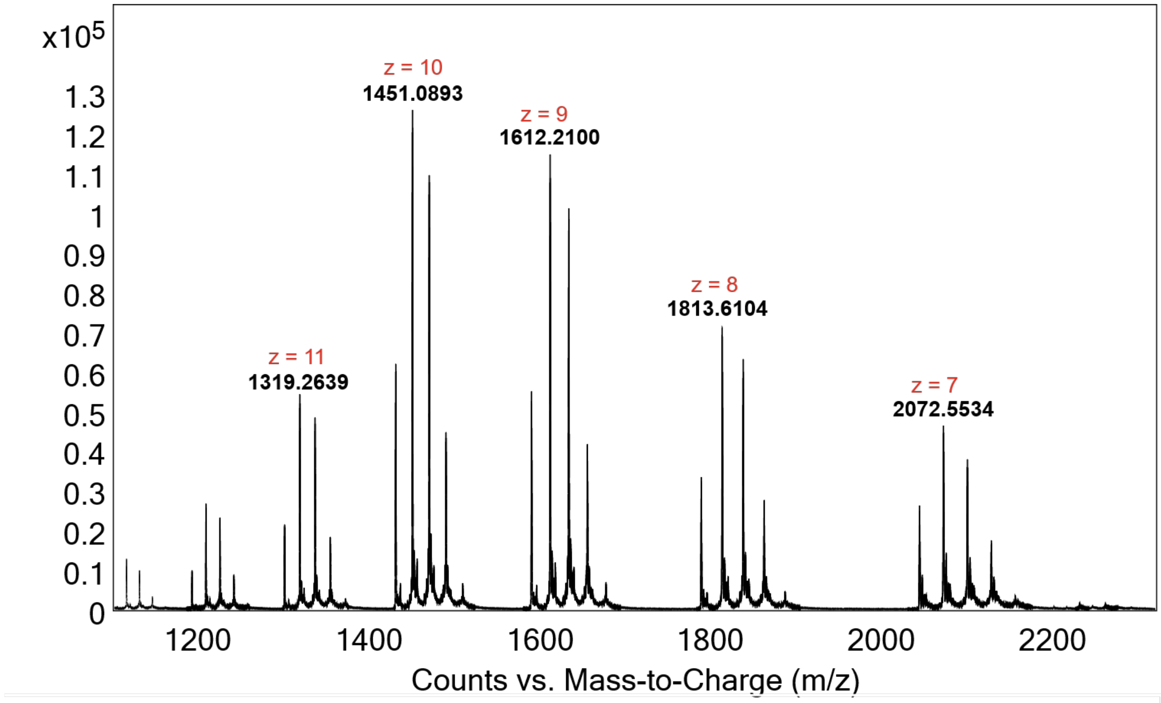

Deconvoluted MS Spectrum of Modified Lysozyme Chicken (1 M sucrose)

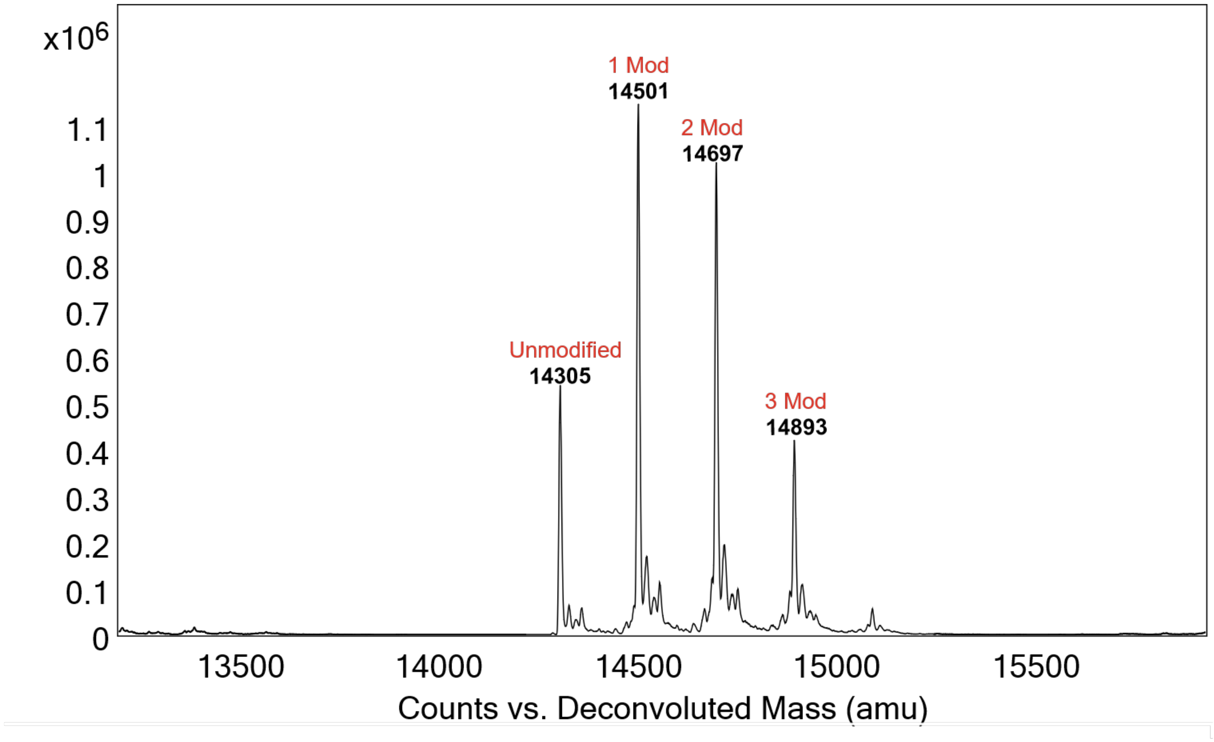

NHS-ester alkyne.

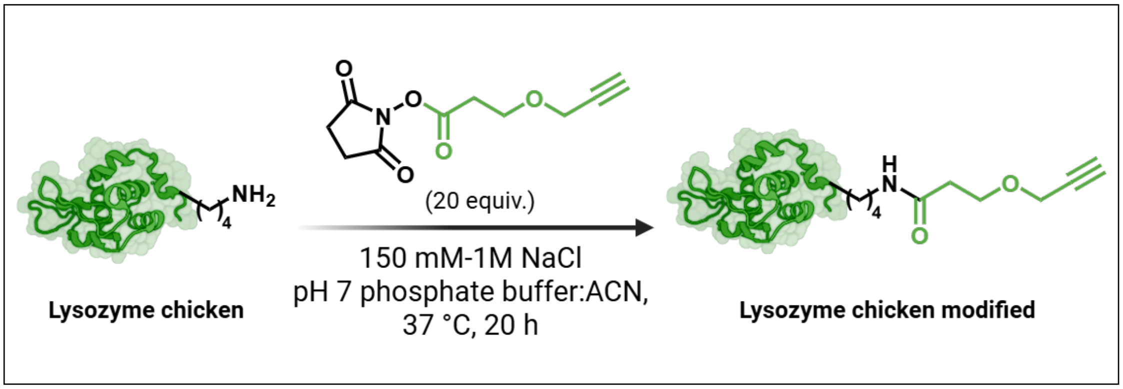

In a one-dram vial, lysozyme chicken (1 mg, 69.9 nmol) was dissolved in 300 µL of a NaCl solution made with sodium phosphate buffer (100 mM, pH 7) and varying concentrations of NaCl (150 mM – 1 M). The pH of the solution was monitored after addition of the NaCl solution. The protein was allowed to stir in the NaCl solution for 1 hour after which NHS-ester alkyne was added (20 equiv.) and left to stir at 37 °C for 20 hours. Subsequently, the reaction mixture was passed through Amicon Ultra 3 kDa spin-concentrator and washed with H_2O (5 x 500 µL) to remove the small molecule impurities. The labeled protein was redissolved in 0.1% formic acid in H_2O and analyzed using LC-MS. Modification of NHS-ester alkyne is confirmed by a mass shift of +110 *m/z*. Using 0 mM NaCl resulted in 99% conversion with 6 modifications, 150 mM NaCl resulted in 38% conversion with 5 modifications and 62% with 6 modifications, 500 mM NaCl resulted in 30% conversion with 4 modifications and 70% conversion with 5 modifications, and 1 M NaCl resulted in 6% conversion with 3 modifications, 38% conversion with 4 modifications, and 56% conversion with 5 modifications.

Intact MS Spectrum of Starting Lysozyme Chicken

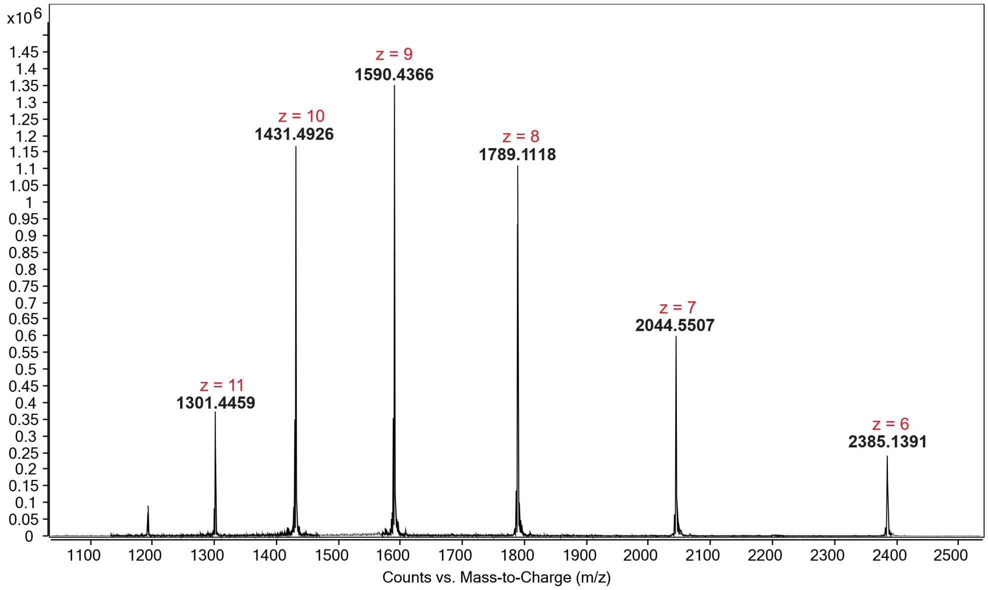

Deconvoluted MS Spectrum of Starting Lysozyme Chicken

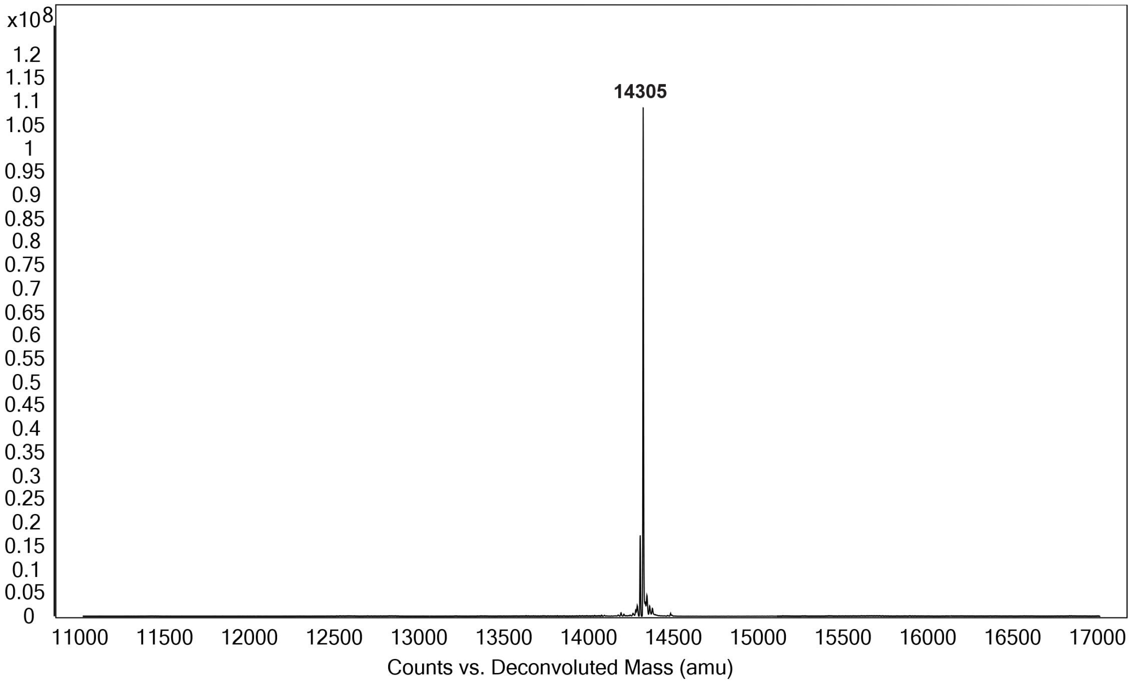

Intact MS Spectrum of Modified Lysozyme Chicken (0 mM NaCl)

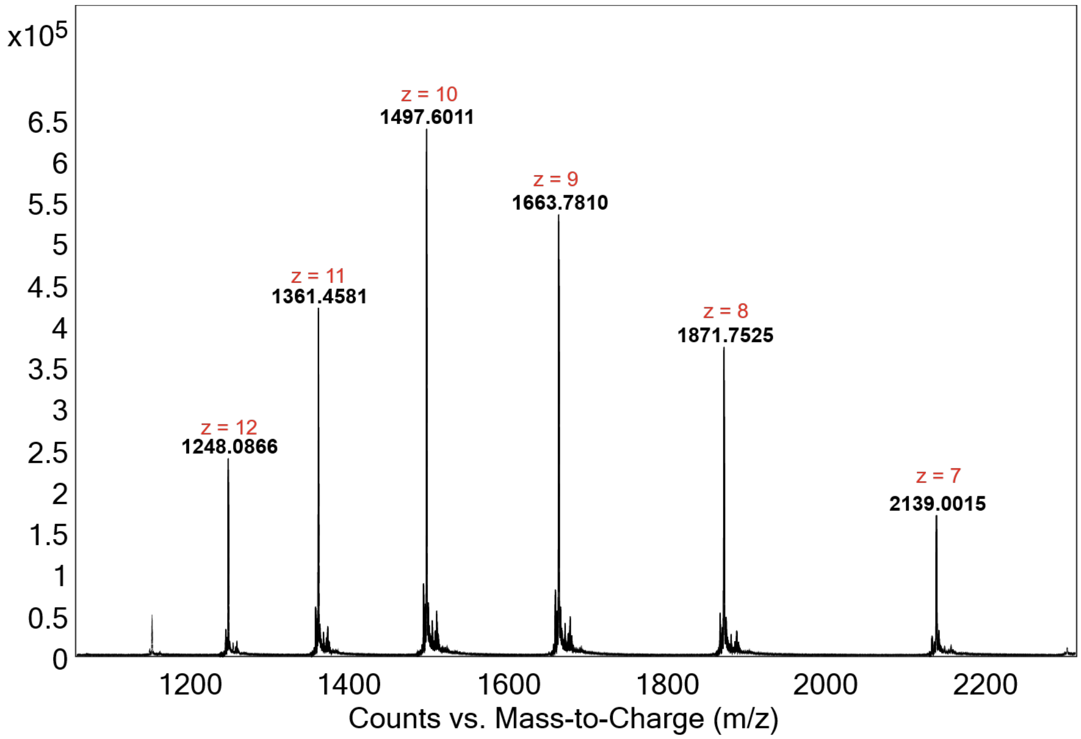

Deconvoluted MS Spectrum of Modified Lysozyme Chicken (0 mM NaCl)

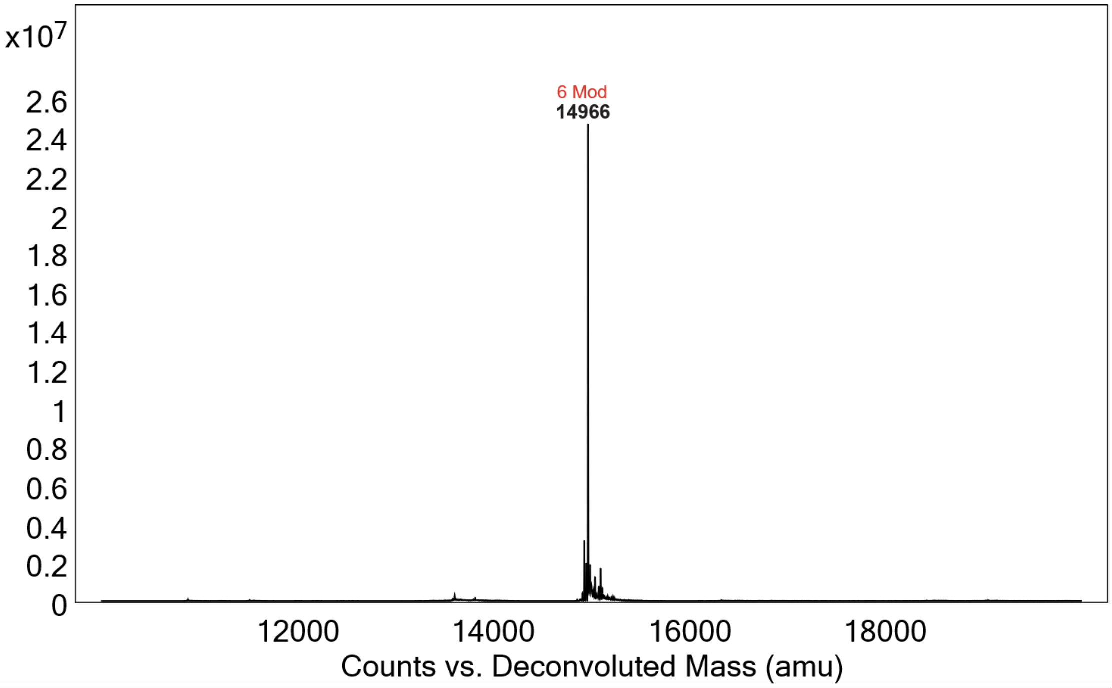

Intact MS Spectrum of Modified Lysozyme Chicken (150 mM NaCl)

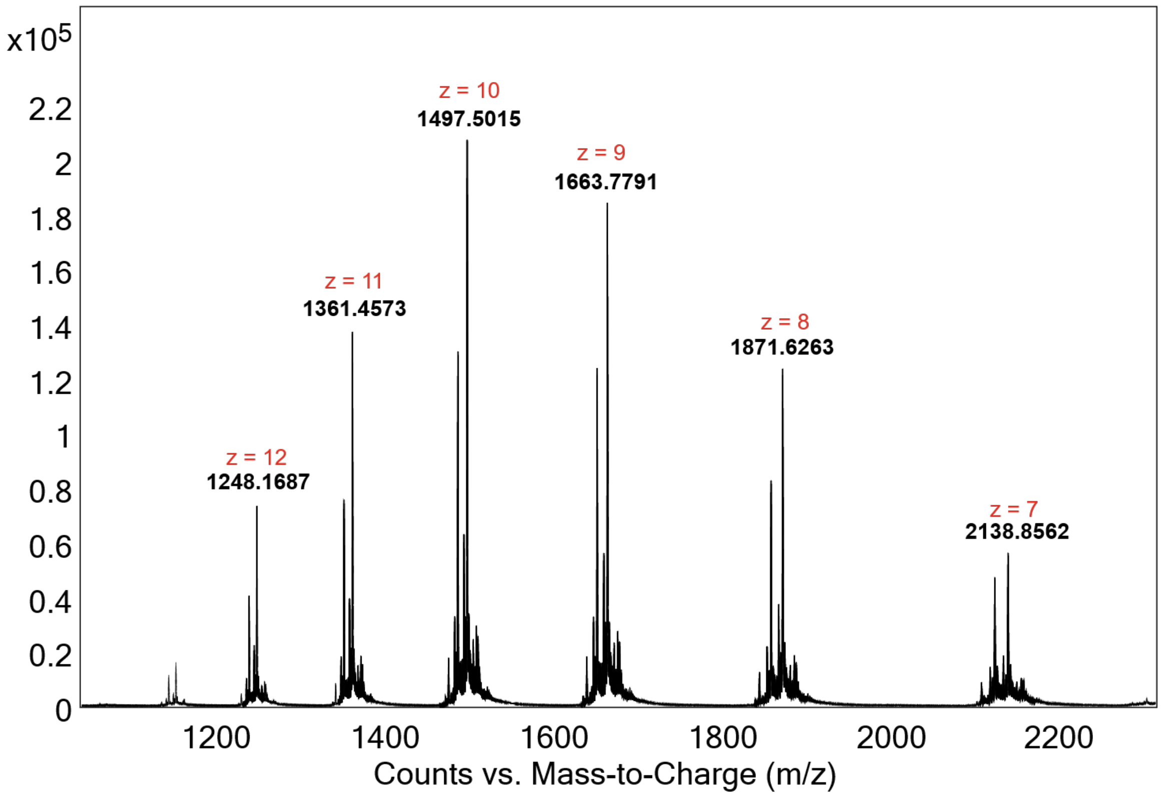

Deconvoluted MS Spectrum of Modified Lysozyme Chicken (150 mM NaCl)

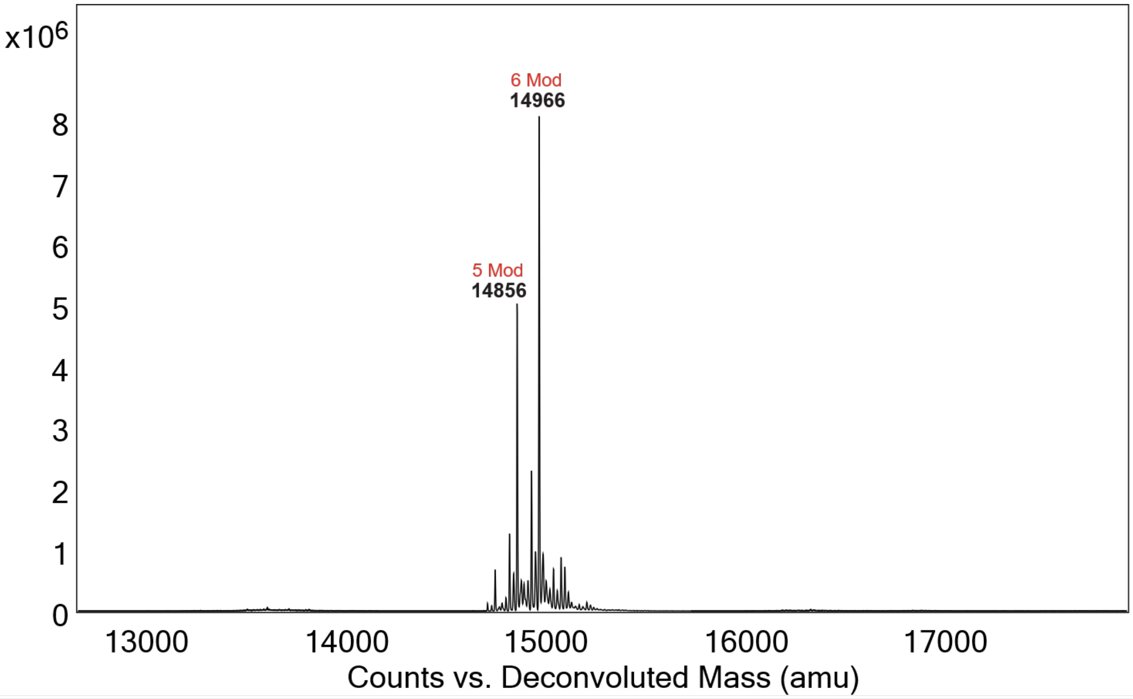

Intact MS Spectrum of Modified Lysozyme Chicken (500 mM NaCl)

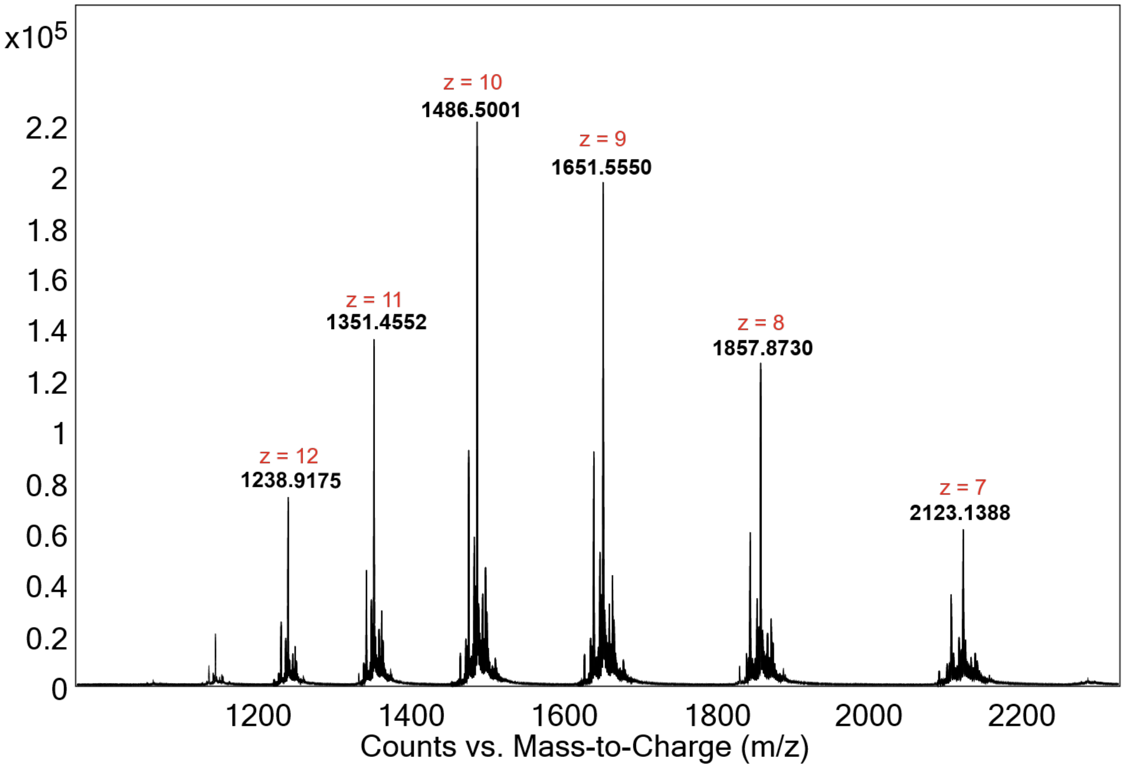

Deconvoluted MS Spectrum of Modified Lysozyme Chicken (500 mM NaCl)

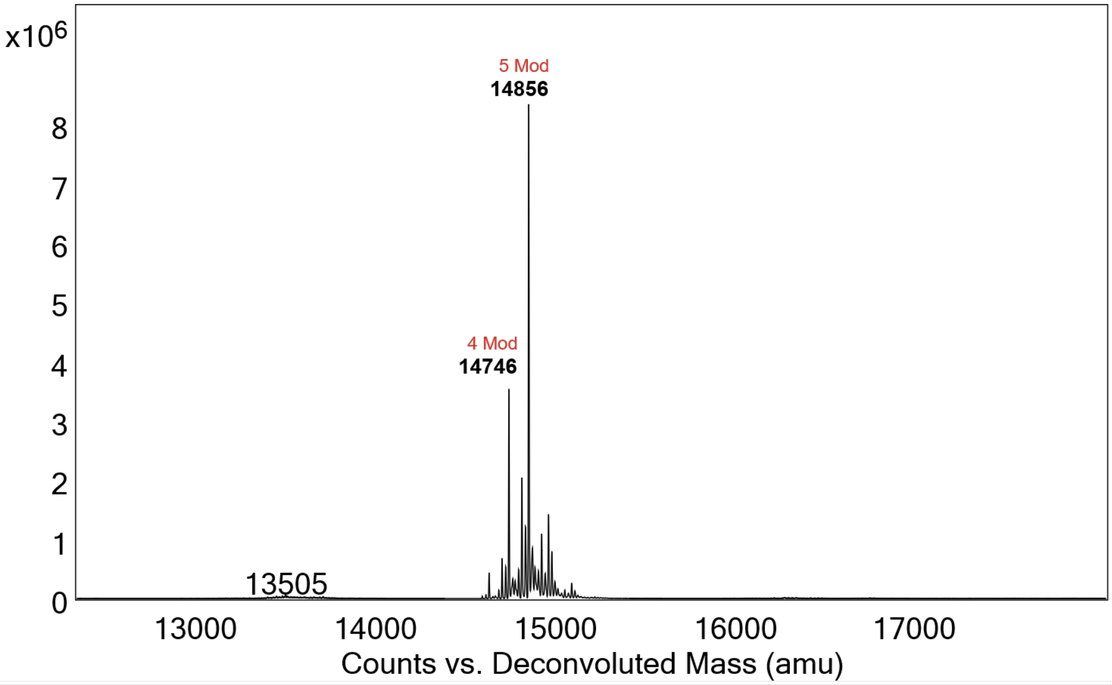

Intact MS Spectrum of Modified Lysozyme Chicken (1 M NaCl)

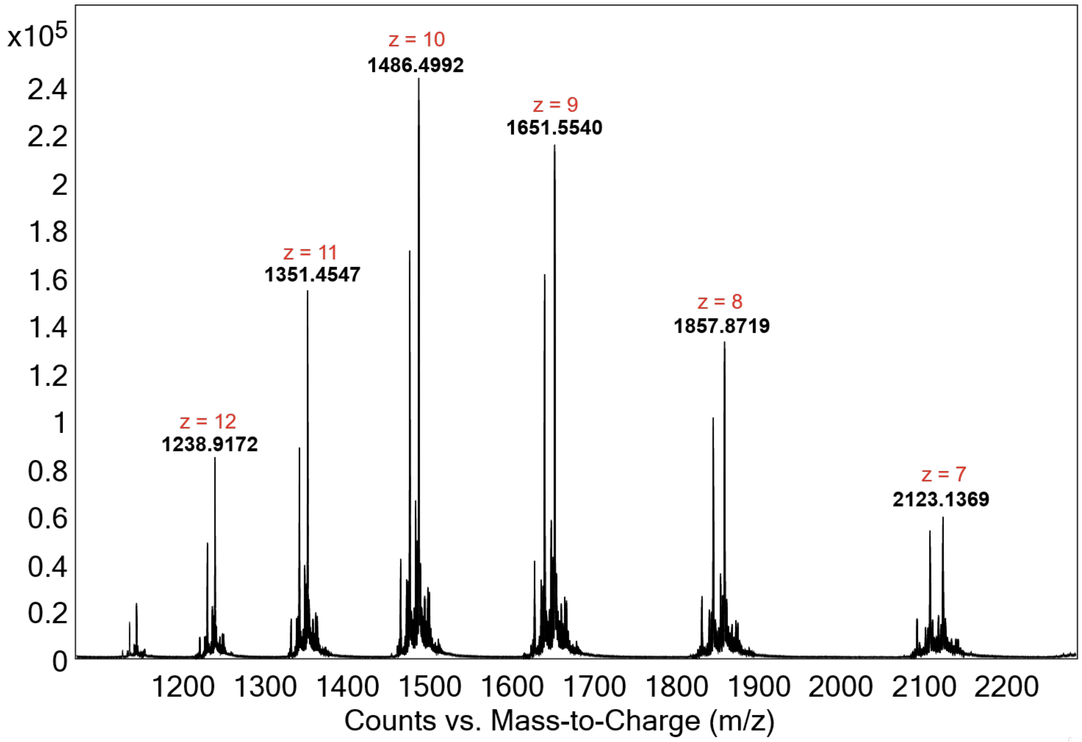

Deconvoluted MS Spectrum of Modified Lysozyme Chicken (1 M NaCl)

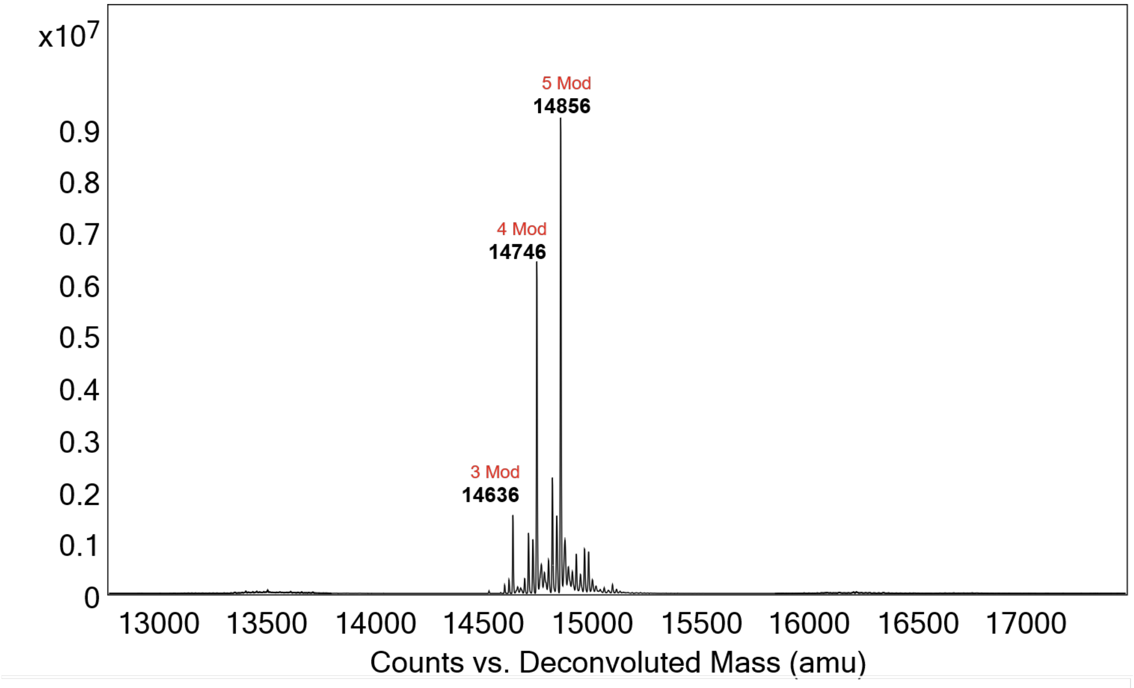

Supplemental Figure 21: Stability Studies

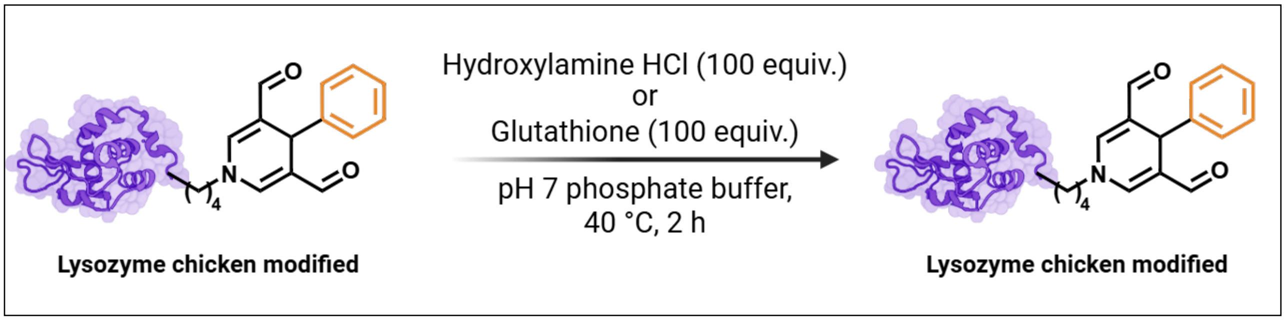

In a one-dram vial, lysozyme chicken (0.5 mg, 35.0 nmol) was dissolved in 300 µL of sodium phosphate buffer (100 mM, pH 7) and hydroxylamine hydrochloride (100 equiv.) or glutathione (100 equiv.) was added. The pH of the solution was monitored after addition of the hydroxylamine hydrochloride. The protein was left to stir at 37 °C for 2 hours. Subsequently, the reaction mixture was passed through Amicon Ultra 3 kDa spin-concentrator and washed with H_2O (5 x 500 µL) to remove the small molecule impurities. The labeled protein was redissolved in 0.1% formic acid in H_2O and analyzed using LC-MS. The undisturbed modification of MDA-benzaldehyde is confirmed by a mass shift of +196 *m/z*. The MDA-benzaldehyde modification remained in both reaction conditions.

Intact MS Spectrum of Starting Lysozyme Chicken

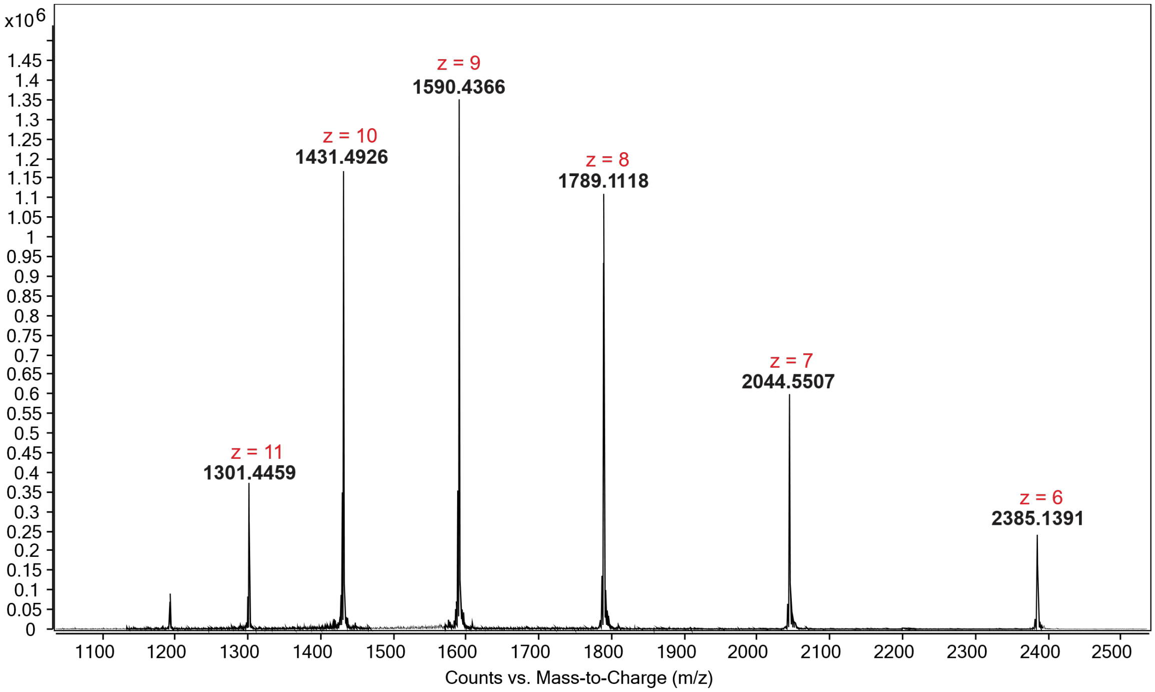

Deconvoluted MS Spectrum of Starting Lysozyme Chicken

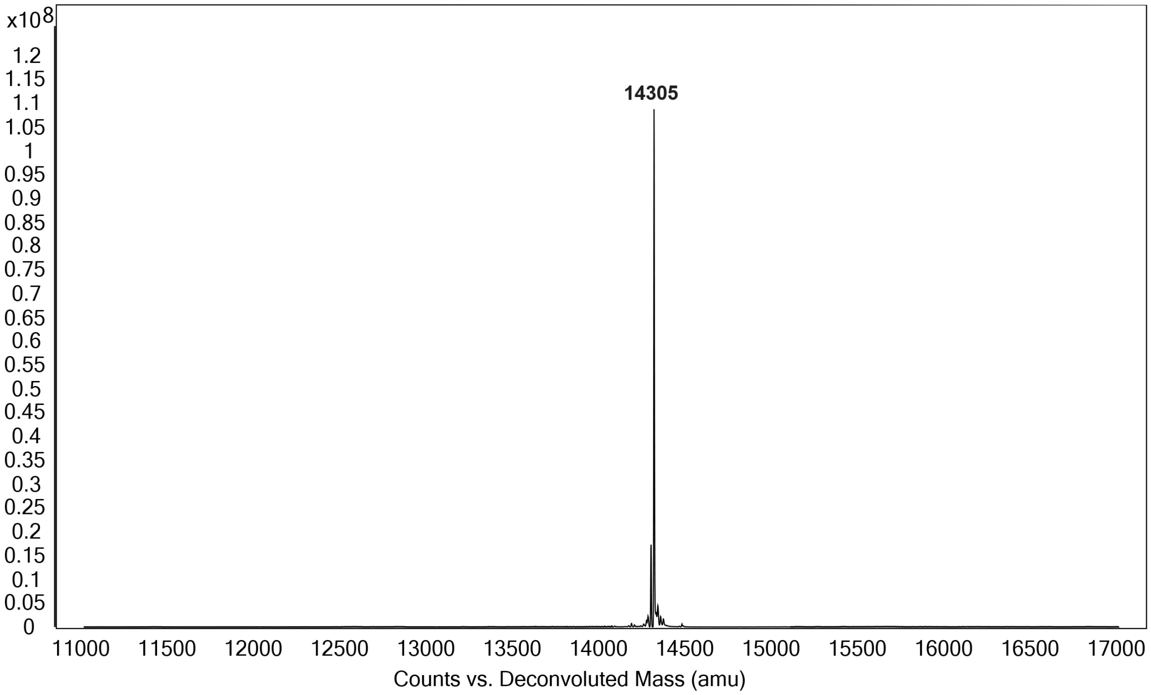

Intact MS Spectrum of Unmodified Lysozyme Chicken Following Hydroxylamine

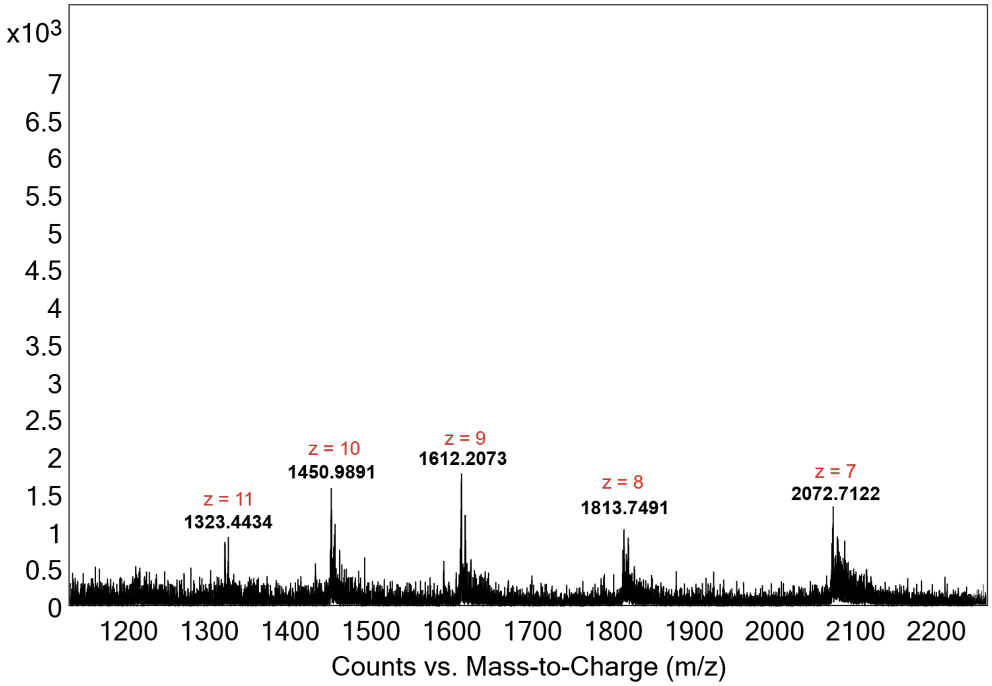

Deconvoluted MS Spectrum of Unmodified Lysozyme Chicken Following Hydroxylamine

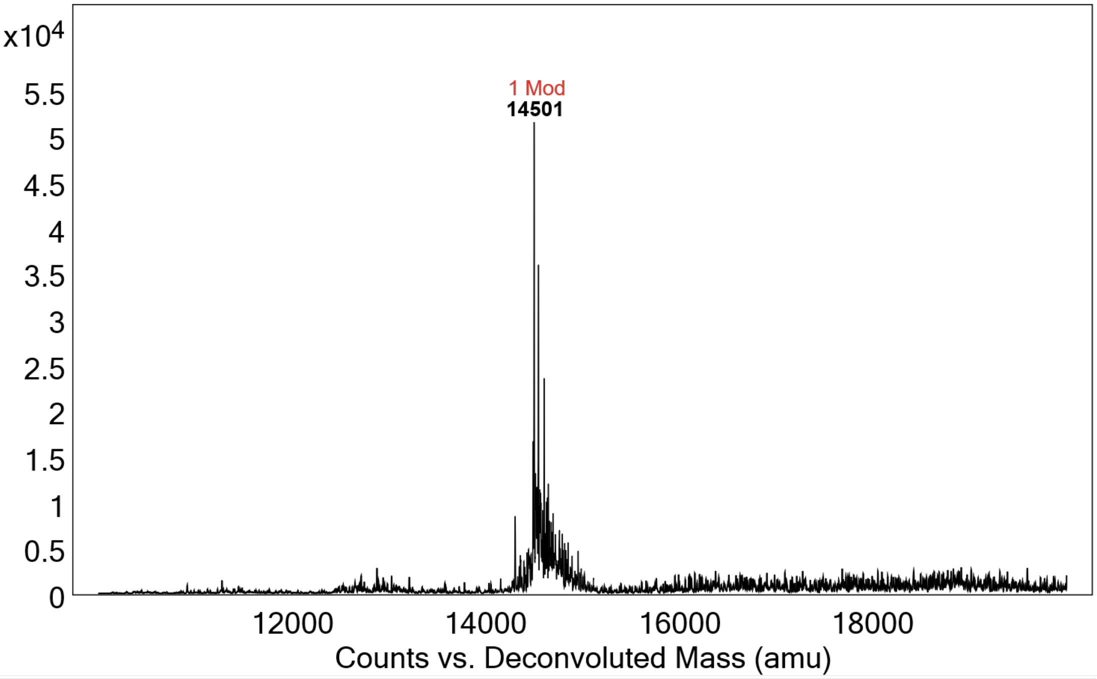

Intact MS Spectrum of Unmodified Lysozyme Chicken Following Glutathione

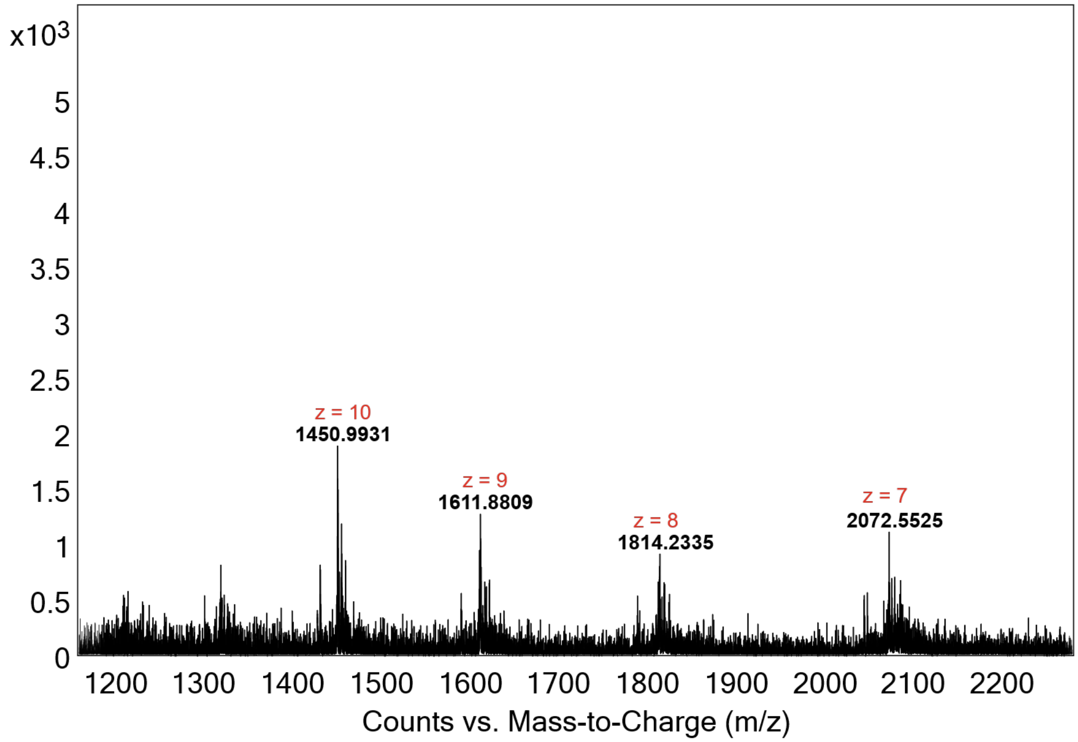

Deconvoluted MS Spectrum of Unmodified Lysozyme Chicken Following Glutathione

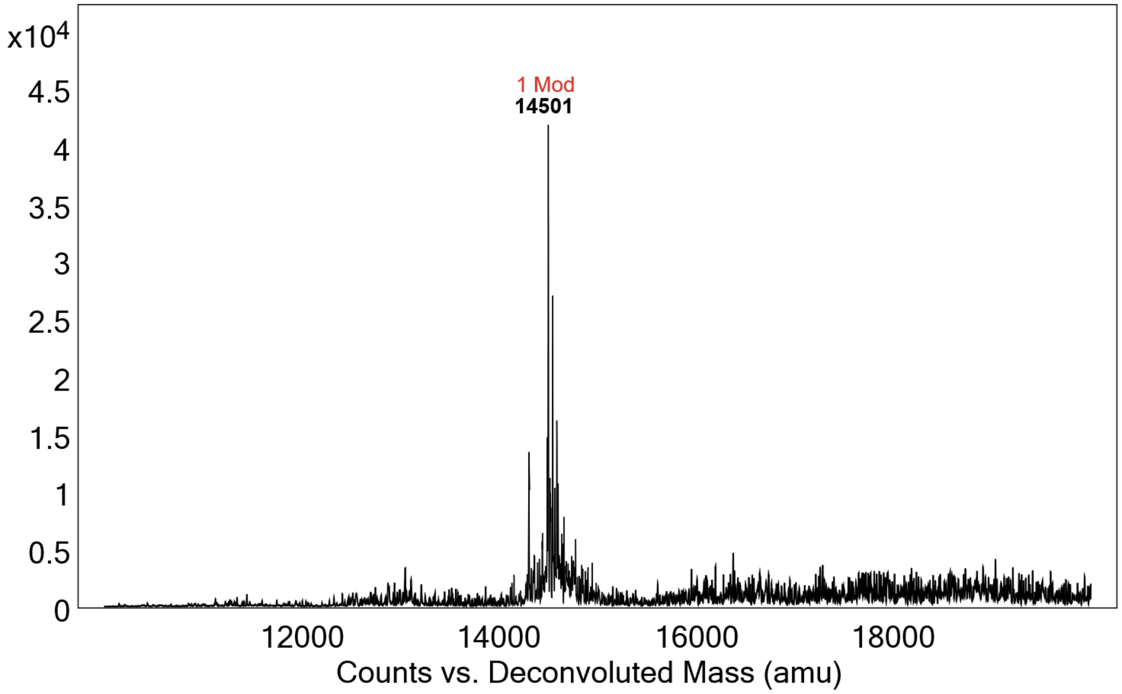

Supplemental Figure 22. GO MF of 167 Protein Cohort and Protein Abundance Distribution.

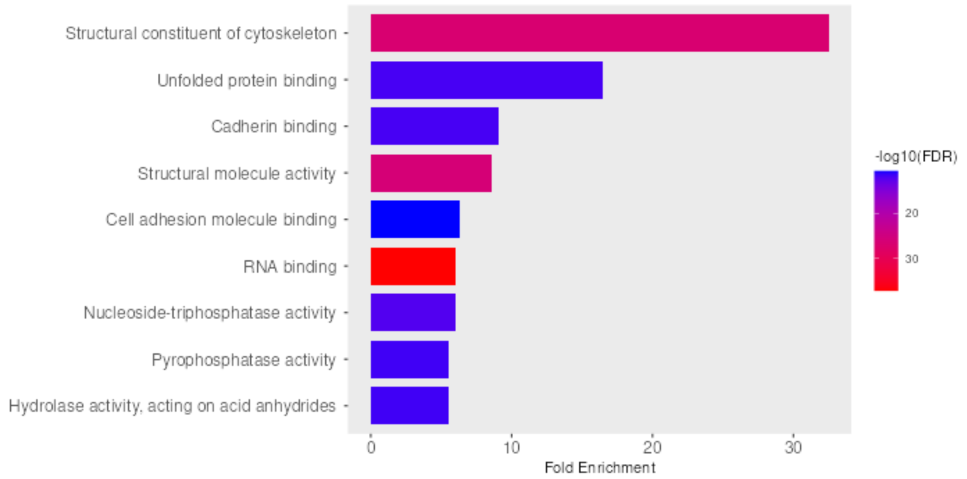

GO Molecular Function analysis of common proteins labeled with MDA-benzaldehyde intermediate. Analysis done with ShinyGO 0.77. Although some enriched proteins are highly abundant (e.g., cytoskeletal and RNA-binding proteins), many core modified proteins fall outside the highest abundance tier, suggesting that modification is not solely driven by protein abundance.

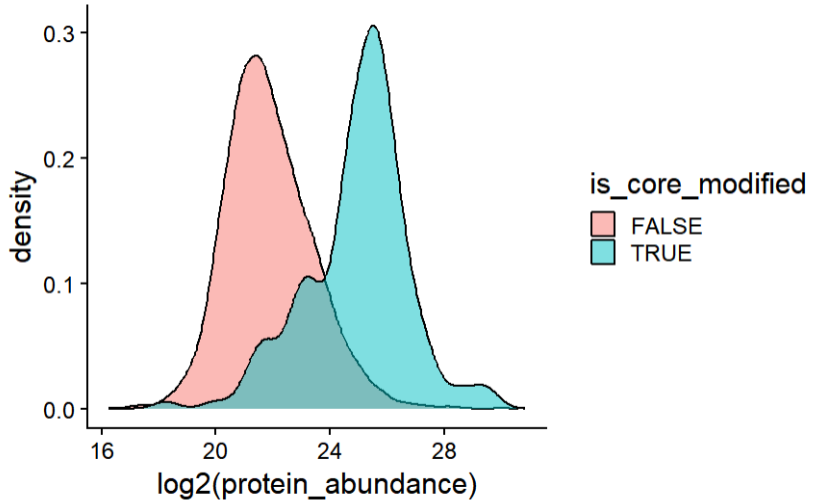

Distribution of baseline protein abundances in the control lysate. Density plot of log2-transformed protein abundance values comparing the full detected proteome (red) and the core modified protein cohort (blue; n = 167). Core modified proteins are enriched among higher-abundance species, yet numerous highly abundant proteins remain unmodified, indicating that modification is not solely driven by protein abundance.

Supplementary References:

1. Chan, W. C.; White, P. D. Fmoc solid phase peptide synthesis. *A practical approach (Oxford Univ. Press, New York)* **2000**.
2. Trivella, A.; Coussan, S.; Chiavassa, T. Malonaldehyde Synthesis. *Synthetic Communications*, **2008**, 3, 3285–3290.
